# Supplementary material for: Enabling nonconjugated polyesters emit full-spectrum fluorescence from blue to near-infrared
Source: Nat Commun. 2024 Jan 8;15:366. doi: 10.1038/s41467-023-44505-3 (PMC10774258; doi:10.1038/s41467-023-44505-3)
Supplement: Supplementary file 1 — Supplementary Information [file 41467_2023_44505_MOESM1_ESM.pdf]

Supplementary Information

Enabling nonconjugated polyesters emit full-spectrum fluorescence from blue to near-infrared

Bo Chu[1], Xiong Liu[1,2,3], Zuping Xiong[1,2,3], Ziteng Zhang[1,2,3], Bin Liu[4], Chengjian Zhang[1], Jing Zhi Sun[1,3], Qing Yang[5], Haoke Zhang[1,2,3]*, Ben Zhong Tang[1,6*], Xing-Hong Zhang[1]*

[1]National Key Laboratory of Biobased Transportation Fuel Technology, International Research Center for X Polymers, Department of Polymer Science and Engineering, Zhejiang University, Hangzhou 310058, China

[2]Zhejiang-Israel Joint Laboratory of Self-Assembling Functional Materials, ZJU-Hangzhou Global Scientific and Technological Innovation Center, Zhejiang University, Hangzhou 311215, China

[3]Centre of Healthcare Materials, Shaoxing Institute, Zhejiang University, Shaoxing 312000, China

[4]School of Energy and Power Engineering, North University of China, Taiyuan 030051, P. R. China

[5]State Key Laboratory of Silicon Materials, Zhejiang University, Hangzhou 310027, China

[6]School of Science and Engineering, Shenzhen Institute of Aggregate Science and Technology, The Chinese University of Hong Kong, Shenzhen (CUHK-Shenzhen), Guangdong 518172, China

*Corresponding emails: xhzhang@zju.edu.cn (Xing-Hong Zhang); tangbenz@cuhk.edu.cn (Ben Zhong Tang); zhanghaoke@zju.edu.cn (Haoke Zhang);

Supplementary Methods 9
Materials *9*
Measurement *9*
Synthesis of P1 .... *10*
Supplementary Figure 1. Synthetic routes to P1. 10
Synthesis of P2 .... *10*
Supplementary Figure 2. Synthetic routes to P2. 11
Synthesis of P1-aTEA (a% are from 0.5% to 5.0%) *11*
Supplementary Figure 3. Synthetic routes to P1-aTEA. 12
Synthesis of P1-2.0DBU, P1-2.0mTBD and P1P1-2.0TBD *12*
Supplementary Figure 4. Synthetic routes to P1-2.0DBU, P1-2.0mTBD and P1-2.0TBD. 13
Synthesis of P2-aTEA (a% are from 0.5% to 5.0%) *13*
Supplementary Figure 5. Synthetic routes to P2-aTEA. 14
Synthesis of P2-2.0DBU, P2-2.0mTBD and P2-2.0TBD *14*
Supplementary Figure 6. Synthetic routes to P2-2.0DBU, P2-2.0mTBD and P2-2.0TBD. 15
Synthesis of P1-5.0DBU *15*
Synthesis of P2-5.0DBU *15*
Synthesis of model molecules M1 .... *16*
Synthesis of model molecules M2 .... *16*
Computational details *16*
Dynamic NMR Spectra of Complexation *16*
Supplementary Discussion 19
Nuclear Magnetic Resonance Spectra *19*
Supplementary Figure 7. ^{1}H NMR spectra of polyester P1 (400 MHz, $CDCl_3$). 19
Supplementary Figure 8. ^{13}C NMR spectra of polyester P1 (100 MHz, $CDCl_3$). 19
Supplementary Figure 9. ^{1}H NMR spectra of polyester P2 (400 MHz, $CDCl_3$). 20
Supplementary Figure 10. ^{13}C NMR spectra of polyester P2 (100 MHz, $CDCl_3$). 20
Supplementary Figure 11. ^{1}H NMR spectra of polyester P1-0.5TEA (400 MHz, $CDCl_3$). 21
Supplementary Figure 12. ^{13}C NMR spectra of polyester P1-0.5TEA (100 MHz, $CDCl_3$). 21
Supplementary Figure 13. ^{1}H NMR spectra of polyester P1-1.0TEA (400 MHz, $CDCl_3$). 22
Supplementary Figure 14. ^{13}C NMR spectra of polyester P1-1.0TEA (100 MHz, $CDCl_3$). 22
Supplementary Figure 15. ^{1}H NMR spectra of polyester P1-1.5TEA (400 MHz, $CDCl_3$). 23
Supplementary Figure 16. ^{13}C NMR spectra of polyester P1-1.5TEA (100 MHz, $CDCl_3$). 23
Supplementary Figure 17. ^{1}H NMR spectra of polyester P1-2.0TEA (400 MHz, $CDCl_3$). 24
Supplementary Figure 18. ^{13}C NMR spectra of polyester P1-2.0TEA (100 MHz, $CDCl_3$). 24
Supplementary Figure 19. ^{1}H NMR spectra of polyester P1-3.0TEA (400 MHz, $CDCl_3$). 25
Supplementary Figure 20. ^{13}C NMR spectra of polyester P1-3.0TEA (100 MHz, $CDCl_3$). 25
Supplementary Figure 21. ^{1}H NMR spectra of polyester P1-4.0TEA (400 MHz, $CDCl_3$). 26
Supplementary Figure 22. ^{13}C NMR spectra of polyester P1-4.0TEA (100 MHz, $CDCl_3$). 26
Supplementary Figure 23. ^{1}H NMR spectra of polyester P1-5.0TEA (400 MHz, $CDCl_3$). 27
Supplementary Figure 24. ^{13}C NMR spectra of polyester P1-5.0TEA (100 MHz, $CDCl_3$). 27
Supplementary Figure 25. ^{1}H NMR spectra of polyester P1-2.0DBU (400 MHz, $CDCl_3$). 28
Supplementary Figure 26. ^{13}C NMR spectra of polyester P1-2.0DBU (100 MHz, $CDCl_3$). 28
Supplementary Figure 27. ^{1}H NMR spectra of polyester P1-2.0mTBD (400 MHz, $CDCl_3$). 29
Supplementary Figure 28. ^{13}C NMR spectra of polyester P1-2.0mTBD (100 MHz, $CDCl_3$). 29
Supplementary Figure 29. ^{1}H NMR spectra of polyester P1-2.0TBD (400 MHz, $CDCl_3$). 30
Supplementary Figure 30. ^{13}C NMR spectra of polyester P1-2.0TBD (100 MHz, $CDCl_3$). 30
Supplementary Figure 31. ^{1}H NMR spectra of polyester P2-0.5TEA (400 MHz, $CDCl_3$). 31
Supplementary Figure 32. ^{13}C NMR spectra of polyester P2-0.5TEA (100 MHz, $CDCl_3$). 31
Supplementary Figure 33. ^{1}H NMR spectra of polyester P2-1.0TEA (400 MHz, $CDCl_3$). 32
Supplementary Figure 34. ^{13}C NMR spectra of polyester P2-1.0TEA (100 MHz, $CDCl_3$). 32
Supplementary Figure 35. ^{1}H NMR spectra of polyester P2-1.5TEA (400 MHz, $CDCl_3$). 33
Supplementary Figure 36. ^{13}C NMR spectra of polyester P2-1.5TEA (100 MHz, $CDCl_3$). 33
Supplementary Figure 37. ^{1}H NMR spectra of polyester P2-2.0TEA (400 MHz, $CDCl_3$). 34
Supplementary Figure 38. ^{13}C NMR spectra of polyester P2-2.0TEA (100 MHz, $CDCl_3$). 34
Supplementary Figure 39. ^{1}H NMR spectra of polyester P2-3.0TEA (400 MHz, $CDCl_3$). 35
Supplementary Figure 40. ^{13}C NMR spectra of polyester P2-3.0TEA (100 MHz, $CDCl_3$). 35
Supplementary Figure 41. ^{1}H NMR spectra of polyester P2-4.0TEA (400 MHz, $CDCl_3$). 36
Supplementary Figure 42. ^{13}C NMR spectra of polyester P2-4.0TEA (100 MHz, $CDCl_3$). 36
Supplementary Figure 43. ^{1}H NMR spectra of polyester P2-5.0TEA (400 MHz, $CDCl_3$). 37

Supplementary Figure 44. ^{13}C NMR spectra of polyester P2-5.0TEA (100 MHz, $CDCl_3$). 37
Supplementary Figure 45. ^{1}H NMR spectra of polyester P2-2.0DBU (400 MHz, $CDCl_3$). 38
Supplementary Figure 46. ^{13}C NMR spectra of polyester P2-2.0DBU (100 MHz, $CDCl_3$). 38
Supplementary Figure 47. ^{1}H NMR spectra of polyester P2-2.0mTBD (400 MHz, $CDCl_3$). 39
Supplementary Figure 48. ^{13}C NMR spectra of polyester P2-2.0mTBD (100 MHz, $CDCl_3$). 39
Supplementary Figure 49. ^{1}H NMR spectra of polyester P2-2.0TBD (400 MHz, $CDCl_3$). 40
Supplementary Figure 50. ^{13}C NMR spectra of polyester P2-2.0TBD (100 MHz, $CDCl_3$). 40
Gel Permeation Chromatography **41**
Supplementary Figure 51. GPC curves of P1 and P2 were used as polystyrene the standard sample using chromatographic purity THF as an eluent at 40 °C. 41
Supplementary Figure 52. GPC curves of P1-aTEA (a% is from 0.5% to 5.0%) were used as polystyrene the standard sample using chromatographic purity THF as an eluent at 40 °C. 41
Supplementary Figure 53. GPC curves of P1-2.0DBU, P1-2.0mTBD and P1-2.0TBD were used as polystyrene the standard sample using chromatographic purity THF as an eluent at 40 °C. 42
Supplementary Figure 54. GPC curves of P2-aTEA (a% is from 0.5% to 5.0%) were used as polystyrene the standard sample using chromatographic purity THF as an eluent at 40 °C. 42
Supplementary Figure 55. GPC curves of P2-2.0DBU, P2-2.0mTBD and P2-2.0TBD were used as polystyrene the standard sample using chromatographic purity THF as an eluent at 40 °C. 43
Matrix Assisted Laser Desorption Ionization-Time of Flight (MALDI-TOF) Mass Spectra **44**
Supplementary Figure 56. MALDI-TOF mass spectrum of P1-2.0TEA. 44
Supplementary Figure 57. MALDI-TOF mass spectrum of P1-2.0DBU. 44
Supplementary Figure 58. MALDI-TOF mass spectrum of P1-2.0mTBD. 45
Supplementary Figure59. MALDI-TOF mass spectrum of P1-2.0TBD. 45
Supplementary Figure 60. MALDI-TOF mass spectrum of P2-2.0TEA. 46
Supplementary Figure 61. MALDI-TOF mass spectrum of P2-2.0DBU. 46
Supplementary Figure 62. MALDI-TOF mass spectrum of P2-2.0mTBD. 47
Supplementary Figure 63. MALDI-TOF mass spectrum of P2-2.0TBD. 47
Structural characterization of M1 and M2 **48**
Supplementary Figure 64. (A) ^{1}H NMR spectra of crude product M1 and (B) stacked ^{1}H NMR spectra of crude product M1 and TEA (400 MHz, $CDCl_3$). 48
Supplementary Figure 65. ESI-MS spectra of crude product M1 in THF of 5mg/mL. 48
Supplementary Figure 66. (A) ^{1}H NMR spectra of crude product M2 and (B) stacked ^{1}H NMR spectra of crude product M2 and TEA (400 MHz, $CDCl_3$). 49
Supplementary Figure 67. ESI-MS spectra of crude product M2 in THF of 5mg/mL. 49
Photophysical characterization of P1~P6 **50**
Supplementary Figure 68. Concentration-dependent UV-Vis absorption spectra of P1 in DCM solution. 50
Supplementary Figure 69. PL spectra of P1 in (A) solid and (B) DCM solution of 10^{-1}M. 50
Supplementary Figure 70. Concentration-dependent spectra of P1 in DCM solution, λ_{ex} = 380 nm. (A) PL spectra at 460 nm, (B) PL intensity plots. Concentration: from 10^{-5} to 10^{-1} M. 51
Influence of TEB on CL **51**
Supplementary Figure 71. PL spectra of P1 catalyzed by TEB-TEA pair (A) and TEA (B) in solid at different excitation wavelength. The molar ratios of PO, SA, TEB and TEA is 2:1:0.02:0.02. 51
Supplementary Figure 72. PL spectra of P1 catalyzed by TEB-TEA pair (A) and TEA (B) in DCM solution of 10^{-1}M. The molar ratios of PO, SA, TEB and TEA is 2:1:0.02:0.02. 52
Supplementary Figure 73. Excitation spectra of P1 catalyzed by TEB-TEA pair (A) and TEA (B) in DCM solution of 10^{-1}M. The molar ratios of PO, SA, TEB and TEA is 2:1:0.02:0.02. 52
Photophysical characterization of P1-aTEA (a% is from 0.5% to 5.0%) **53**
Supplementary Figure 74. UV-Vis absorption spectra of P1-aTEA in DCM solution of 10^{-3}M. 53
Supplementary Figure 75. Concentration-dependent UV-Vis absorption spectra of P1-0.5TEA in DCM solution. Concentration: from 10^{-5} to 10^{-1} M. 53
Supplementary Figure 76. Concentration-dependent UV-Vis absorption spectra of P1-1.0TEA in DCM solution. Concentration: from 10^{-5} to 10^{-1} M. 54
Supplementary Figure 77. Concentration-dependent UV-Vis absorption spectra of P1-1.5TEA in DCM solution. Concentration: from 10^{-5} to 10^{-1} M. 54
Supplementary Figure 78. Concentration-dependent UV-Vis absorption spectra of P1-2.0TEA in DCM solution. Concentration: from 10^{-5} to 10^{-1} M. 55
Supplementary Figure 79. Concentration-dependent UV-Vis absorption spectra of P1-3.0TEA in DCM solution. Concentration: from 10^{-5} to 10^{-1} M. 55
Supplementary Figure 80. Concentration-dependent UV-Vis absorption spectra of P1-4.0TEA in DCM solution. Concentration: from 10^{-5} to 10^{-1} M. 56

Supplementary Figure 81. Concentration-dependent UV-Vis absorption spectra of P1-5.0TEA in DCM solution. Concentration: from 10^{-5} to 10^{-1} M. 56
Supplementary Figure 82. PL spectra of P1-0.5TEA in solid under different excitation wavelengths. 57
Supplementary Figure 83. PL spectra of P1-1.0TEA in solid under different excitation wavelengths. 57
Supplementary Figure 84. PL spectra of P1-1.5TEA in solid under different excitation wavelengths. 58
Supplementary Figure 85. PL spectra of P1-2.0TEA in solid under different excitation wavelengths. 58
Supplementary Figure 86. PL spectra of P1-3.0TEA in solid under different excitation wavelengths. 59
Supplementary Figure 87. PL spectra of P1-4.0TEA in solid under different excitation wavelengths. 59
Supplementary Figure 88. PL spectra of P1-5.0TEA in solid under different excitation wavelengths. 60
Supplementary Figure 89. PL spectra (A) under different excitation wavelengths and excitation spectra (B) under different emission wavelengths of P1-0.5TEA in DCM solution of 10^{-1} M. 60
Supplementary Figure 90. PL spectra (A) under different excitation wavelengths and excitation spectra (B) under different emission wavelengths of P1-1.0TEA in DCM solution of 10^{-1} M. 61
Supplementary Figure 91. PL spectra (A) under different excitation wavelengths and excitation spectra (B) under different emission wavelengths of P1-1.5TEA in DCM solution of 10^{-1} M. 61
Supplementary Figure 92. PL spectra (A) under different excitation wavelengths and excitation spectra (B) under different emission wavelengths of P1-2.0TEA in DCM solution of 10^{-1} M. 62
Supplementary Figure 93. PL spectra (A) under different excitation wavelengths and excitation spectra (B) under different emission wavelengths of P1-3.0TEA in DCM solution of 10^{-1} M. 62
Supplementary Figure 94. PL spectra (A) under different excitation wavelengths and excitation spectra (B) under different emission wavelengths of P1-4.0TEA in DCM solution of 10^{-1} M. 63
Supplementary Figure 95. PL spectra (A) under different excitation wavelengths and excitation spectra (B) under different emission wavelengths of P1-5.0TEA in DCM solution of 10^{-1} M. 63
Supplementary Figure 96. Plots of ratios of intensities versuscontents of TEA for P1-aTEA in DCM solution of 10^{-1} M. The intensity ratios are of long-wavelength CL (λ_{ex} = 520 nm) to short-wavelength PL (λ_{ex} = 360 nm). 64
Supplementary Figure 97. Time-resolved PL decay curves of P1-0.5TEA measured at emission maximum of 480 nm in solid and DCM solution ($c = 10^{-3}$ M), λ_{ex} = 365 nm 64
Supplementary Figure 98. Time-resolved PL decay curves of P1-0.5TEA measured at emission maximum of 600 nm in solid and DCM solution ($c = 10^{-3}$ M), λ_{ex} = 365 nm 65
Supplementary Figure 99. Time-resolved PL decay curves of P1-1.0TEA measured at emission maximum of 480 nm in solid and DCM solution ($c = 10^{-3}$ M), λ_{ex} = 365 nm 65
Supplementary Figure 100. Time-resolved PL decay curves of P1-1.0TEA measured at emission maximum of 600 nm in solid and DCM solution ($c = 10^{-3}$ M), λ_{ex} = 365 nm. 66
Supplementary Figure 101. Time-resolved PL decay curves of P1-1.5TEA measured at emission maximum of 480 nm in solid and DCM solution ($c = 10^{-3}$ M), λ_{ex} = 365 nm. 66
Supplementary Figure 102. Time-resolved PL decay curves of P1-1.5TEA measured at emission maximum of 600 nm in solid and DCM solution ($c = 10^{-3}$ M), λ_{ex} = 365 nm. 67
Supplementary Figure 103. Time-resolved PL decay curves of P1-2.0TEA measured at emission maximum of 480 nm in solid and DCM solution ($c = 10^{-3}$ M), λ_{ex} = 365 nm. 67
Supplementary Figure 104. Time-resolved PL decay curves of P1-2.0TEA measured at emission maximum of 600 nm in solid and DCM solution ($c = 10^{-3}$ M), λ_{ex} = 365 nm. 68
Supplementary Figure 105. Time-resolved PL decay curves of P1-3.0TEA measured at emission maximum of 480 nm in solid and DCM solution ($c = 10^{-3}$ M), λ_{ex} = 365 nm. 68
Supplementary Figure 106. Time-resolved PL decay curves of P1-3.0TEA measured at emission maximum of 600 nm in solid and DCM solution ($c = 10^{-3}$ M), λ_{ex} = 365 nm. 69
Supplementary Figure 107. Time-resolved PL decay curves of P1-4.0TEA measured at emission maximum of 480 nm in solid and DCM solution ($c = 10^{-3}$ M), λ_{ex} = 365 nm. 69
Supplementary Figure 108. Time-resolved PL decay curves of P1-4.0TEA measured at emission maximum of 600 nm in solid and DCM solution ($c = 10^{-3}$ M), λ_{ex} = 365 nm. 70
Supplementary Figure 109. Time-resolved PL decay curves of P1-5.0TEA measured at emission maximum of 480 nm in solid and DCM solution ($c = 10^{-3}$ M), λ_{ex} = 365 nm. 70
Supplementary Figure 110. Time-resolved PL decay curves of P1-5.0TEA measured at emission maximum of 600 nm in solid and DCM solution ($c = 10^{-3}$ M), λ_{ex} = 365 nm. 71
Supplementary Figure 111. (A) Concentration-dependent PL spectra of P1-0.5TEA in DCM solution, λ_{ex} = 520 nm.(B) Plots of emission wavelength and PL intensity versusversus concentration. Concentration: from 10^{-5} to 10^{-1} M.71
Supplementary Figure 112. (A) Concentration-dependent spectra of P1-1.0TEA in DCM solution, λ_{ex} = 480 nm. (B) Plots of emission wavelength and PL intensity versus concentration.concentration Concentration: from 10^{-5} to 10^{-1} M.72
Supplementary Figure 113. (A) Concentration-dependent spectra of P1-1.5TEA in DCM solution, λ_{ex} = 480 nm. (B) Plots of emission wavelength and PL intensity and emission wavelength plots versus concentration.concentration Concentration: from 10^{-5} to 10^{-1} M. 72

Supplementary Figure 114. (A) Concentration-dependent spectra of P1-2.0TEA in DCM solution, λ_{ex} = 520 nm. (B) Plots of emission wavelength and PL intensity and emission wavelength plots versus concentration.concentration Concentration: from 10^{-5} to 10^{-1} M. 73
Supplementary Figure 115. (A) Concentration-dependent spectra of P1-3.0TEA in DCM solution, λ_{ex} = 540 nm. (B) Plots of emission wavelength and PL intensity and emission wavelength plots versus concentration.concentration Concentration: from 10^{-5} to 10^{-1} M. 73
Supplementary Figure 116. (A) Concentration-dependent spectra of P1-4.0TEA in DCM solution, λ_{ex} = 540 nm. (B) Plots of emission wavelength and PL intensity and emission wavelength plots versus concentration.concentration Concentration: from 10^{-5} to 10^{-1} M. 74
Supplementary Figure 117. (A) Concentration-dependent spectra of P1-5.0TEA in DCM solution, λ_{ex} = 520 nm. (B) Plots of emission wavelength and PL intensity and emission wavelength plots versus concentration.concentration Concentration: from 10^{-5} to 10^{-1} M. 74
Photophysical characterization of P2-aTEA (a% is from 0.5% to 5.0%)* *75
Supplementary Figure 118. Concentration-dependent UV-Vis absorption spectra of P2-0.5TEA in DCM solution. Concentration: from 10^{-5} to 10^{-1} M. 75
Supplementary Figure 119. Concentration-dependent UV-Vis absorption spectra of P2-1.0TEA in DCM solution. Concentration: from 10^{-5} to 10^{-1} M. 75
Supplementary Figure 120. Concentration-dependent UV-Vis absorption spectra of P2-1.5TEA in DCM solution. Concentration: from 10^{-5} to 10^{-1} M. 76
Supplementary Figure 121. Concentration-dependent UV-Vis absorption spectra of P2-2.0TEA in DCM solution. Concentration: from 10^{-5} to 10^{-1} M. 76
Supplementary Figure 122. Concentration-dependent UV-Vis absorption spectra of P2-3.0TEA in DCM solution. Concentration: from 10^{-5} to 10^{-1} M. 77
Supplementary Figure 123. Concentration-dependent UV-Vis absorption spectra of P2-4.0TEA in DCM solution. Concentration: from 10^{-5} to 10^{-1} M. 77
Supplementary Figure 124. Concentration-dependent UV-Vis absorption spectra of P2-5.0TEA in DCM solution. Concentration: from 10^{-5} to 10^{-1} M. 78
Supplementary Figure 125. PL spectra of P2-0.5TEA in solid under different excitation wavelengths. 78
Supplementary Figure 126. PL spectra of P2-1.0TEA in solid under different excitation wavelengths. 79
Supplementary Figure 127. PL spectra of P2-1.5TEA in solid under different excitation wavelengths. 79
Supplementary Figure 128. PL spectra of P2-2.0TEA in solid under different excitation wavelengths. 80
Supplementary Figure 129. PL spectra of P2-3.0TEA in solid under different excitation wavelengths. 80
Supplementary Figure 130. PL spectra of P2-4.0TEA in solid under different excitation wavelengths. 81
Supplementary Figure 131. PL spectra of P2-5.0TEA in solid under different excitation wavelengths. 81
Supplementary Figure 132. PL spectra (A) under different excitation wavelengths and excitation spectra (B) under different emission wavelengths of P2-0.5TEA in DCM solution of 10^{-1} M. 82
Supplementary Figure 133. PL spectra (A) under different excitation wavelengths and excitation spectra (B) under different emission wavelengths of P2-1.0TEA in DCM solution of 10^{-1} M. 82
Supplementary Figure 134. PL spectra (A) under different excitation wavelengths and excitation spectra (B) under different emission wavelengths of P2-1.5TEA in DCM solution of 10^{-1} M. 83
Supplementary Figure 135. PL spectra (A) under different excitation wavelengths and excitation spectra (B) under different emission wavelengths of P2-2.0TEA in DCM solution of 10^{-1} M. 83
Supplementary Figure 136. PL spectra (A) under different excitation wavelengths and excitation spectra (B) under different emission wavelengths of P2-3.0TEA in DCM solution of 10^{-1} M. 84
Supplementary Figure 137. PL spectra (A) under different excitation wavelengths and excitation spectra (B) under different emission wavelengths of P2-4.0TEA in DCM solution of 10^{-1} M. 84
Supplementary Figure 138. PL spectra (A) under different excitation wavelengths and excitation spectra (B) under different emission wavelengths of P2-5.0TEA in DCM solution of 10^{-1} M. 85
Supplementary Figure 139. Time-resolved PL decay curves of P2-0.5TEA measured at emission maximum of 480 nm in solid and DCM solution ($c = 10^{-3}$ M), λ_{ex} = 365 nm. 85
Supplementary Figure 140. Time-resolved PL decay curves of P2-0.5TEA measured at emission maximum of 680 nm in solid and DCM solution ($c = 10^{-3}$ M), λ_{ex} = 470 nm. 86
Supplementary Figure 141. Time-resolved PL decay curves of P2-1.0TEA measured at emission maximum of 480 nm in solid and DCM solution ($c = 10^{-3}$ M), λ_{ex} = 365 nm. 86
Supplementary Figure 142. Time-resolved PL decay curves of P2-1.0TEA measured at emission maximum of 680 nm in solid and DCM solution ($c = 10^{-3}$ M), λ_{ex} = 470 nm. 87
Supplementary Figure 143. Time-resolved PL decay curves of P2-1.5TEA measured at emission maximum of 480 nm in solid and DCM solution ($c = 10^{-3}$ M), λ_{ex} = 365 nm. 87
Supplementary Figure 144. Time-resolved PL decay curves of P2-1.5TEA measured at emission maximum of 680 nm in solid and DCM solution ($c = 10^{-3}$ M), λ_{ex} = 470 nm. 88

Supplementary Figure 145. Time-resolved PL decay curves of P2-2.0TEA measured at emission maximum of 480 nm in solid and DCM solution (c = 10^{-3} M), λ_{ex} = 365 nm. 88
Supplementary Figure 146. Time-resolved PL decay curves of P2-2.0TEA measured at emission maximum of 680 nm in solid and DCM solution (c = 10^{-3} M), λ_{ex} = 470 nm. 89
Supplementary Figure 147. Time-resolved PL decay curves of P2-3.0TEA measured at emission maximum of 480 nm in solid and DCM solution (c = 10^{-3} M), λ_{ex} = 365 nm. 89
Supplementary Figure 148. Time-resolved PL decay curves of P2-3.0TEA measured at emission maximum of 680 nm in solid and DCM solution (c = 10^{-3} M), λ_{ex} = 470 nm. 90
Supplementary Figure 149. Time-resolved PL decay curves of P2-4.0TEA measured at emission maximum of 480 nm in solid and DCM solution (c = 10^{-3} M), λ_{ex} = 365 nm. 90
Supplementary Figure 150. Time-resolved PL decay curves of P2-4.0TEA measured at emission maximum of 680 nm in solid and DCM solution (c = 10^{-3} M), λ_{ex} = 470 nm. 91
Supplementary Figure 151. Time-resolved PL decay curves of P2-5.0TEA measured at emission maximum of 480 nm in solid and DCM solution (c = 10^{-3} M), λ_{ex} = 365 nm. 91
Supplementary Figure 152. Time-resolved PL decay curves of P2-5.0TEA measured at emission maximum of 680 nm in solid and DCM solution (c = 10^{-3} M), λ_{ex} = 470 nm. 92
Supplementary Figure 153. Concentration-dependent PL spectra (A)~(I) of P2-0.5TEA in DCM solution under different excitation wavelengths. Concentration: from 10^{-5} to 10^{-1} M. 92
Supplementary Figure 154. Concentration-dependent PL spectra (A)~(I) of P2-1.0TEA in DCM solution under different excitation wavelengths. Concentration: from 10^{-5} to 10^{-1} M. 93
Supplementary Figure 155. Concentration-dependent PL spectra (A)~(I) of P2-1.5TEA in DCM solution under different excitation wavelengths. Concentration: from 10^{-5} to 10^{-1} M. 93
Supplementary Figure 156. Concentration-dependent PL spectra (A)~(I) of P2-2.0TEA in DCM solution under different excitation wavelengths. Concentration: from 10^{-5} to 10^{-1} M. 94
Supplementary Figure 157. Concentration-dependent PL spectra (A)~(I) of P2-3.0TEA in DCM solution under different excitation wavelengths. Concentration: from 10^{-5} to 10^{-1} M. 94
Supplementary Figure 158. Concentration-dependent PL spectra (A)~(I) of P2-4.0TEA in DCM solution under different excitation wavelengths. Concentration: from 10^{-5} to 10^{-1} M. 95
Supplementary Figure 159. Concentration-dependent PL spectra (A)~(I) of P2-5.0TEA in DCM solution under different excitation wavelengths. Concentration: from 10^{-5} to 10^{-1} M. 95
Photophysical characterization of P1-2.0amine and P2-2.0amine*.......... *96
Supplementary Figure 160. PL spectra of (A)P1-2.0TEA, (B) P1-2.0DBU, (C) P1-2.0mTBD and (D) P1-2.0TBD in solid under different excitation wavelengths. 96
Supplementary Figure 161. PL spectra of (A)P2-2.0TEA, (B) P2-2.0DBU, (C) P2-2.0mTBD and (D) P2-2.0TBD in solid under different excitation wavelengths. 97
Supplementary Figure 162. Normalzied PL spectra of P2-2.0TEA, P2-2.0DBU, P2-2.0mTBD and P2-2.0TBD in solid at λ_{ex} = 360 nm. Inset: photographs taken under 365 nm UV light. 98
Dynamic NMR spectra of complexation*.......... *98
Supplementary Figure 163. Time-dependent ^{1}H-NMR spectra of P2@DBU (400 MHz, $CDCl_3$). The molar ratio of structural units of P2 to DBU is 5:1. The h and d respectively represent hours and days. 98
Supplementary Figure 164. Time-dependent ^{13}C-NMR spectra of P2@DBU (100 MHz, $CDCl_3$). The molar ratio of structural units of P2 to DBU is 5:1. The h and d respectively represent hours and days. 99
Supplementary Figure 165. Plots of absolute value of chemical shift changes ($\Delta\delta\Delta\delta_H$ for hydrogen atoms, $\Delta\delta\Delta\delta_C$ for carbon atoms) versus atom positions on DBU for P2@DBU. 99
Supplementary Figure 166. Time-dependent ^{1}H-NMR spectra of DS@DBU (400 MHz, $CDCl_3$). The molar ratio of structural units of DS to DBU is 5:1. The h and d respectively represent hours and days. 100
Supplementary Figure 167. Time-dependent ^{13}C-NMR spectra of DS@DBU (100 MHz, $CDCl_3$). The molar ratio of structural units of DS to DBU is 5:1. The h and d respectively represent hours and days. 100
Supplementary Figure 168. Time-dependent ^{1}H-NMR spectra of DC@DBU (400 MHz, $CDCl_3$).. The molar ratio of structural units of DC to DBU is 5:1. The h and d respectively represent hours and days. 101
Supplementary Figure 169. Time-dependent ^{13}C-NMR spectra of DC@DBU (100 MHz, $CDCl_3$).. The molar ratio of structural units of DC to DBU is 5:1. The h and d respectively represent hours and days. 101
Supplementary Figure 170. Plots of absolute value of chemical shift changes ($\Delta\delta_H$ for hydrogen atoms, $\Delta\delta_C$ for carbon atoms) versus atom positions on DBU for DC@DBU. 102
Supplementary Figure 171. Stacked ^{1}H-NMR spectra of CA@DBU with molar ratio of structural units of CA to DBU is from 5:0 to 0:5 (400 MHz, $CDCl_3$). 102
Supplementary Figure 172. Stacked ^{13}C-NMR spectra of CA@DBU with molar ratio of structural units of CA to DBU is from 5:0 to 0:5 (100 MHz, $CDCl_3$). 103
Supplementary Figure 173. Plots of absolute value of chemical shift changes ($\Delta\delta_H$ for hydrogen atoms, $\Delta\delta_C$ for carbon atoms) versus atom positions on DBU for CA@DBU. 103

Supplementary Figure 174. Stacked ^{1}H-NMR spectra of SA@DBU with molar ratio of structural units of SA to DBU is from 5:0 to 0:5 (400 MHz, $CDCl_3$). 104
Supplementary Figure 175. Stacked ^{13}C-NMR spectra of SA@DBU with molar ratio of structural units of SA to DBU is from 5:0 to 0:5 (100 MHz, $CDCl_3$). 104
Supplementary Figure 176. Plots of absolute value of chemical shift changes ($\Delta\delta_H$ for hydrogen atoms, $\Delta\delta_C$ for carbon atoms) versus atom positions on DBU for SA@DBU.......... 105
Supplementary Figure 177. ^{1}H-NMR spectra of DBU (400 MHz, $CDCl_3$). 105
Supplementary Figure 178. ^{1}H-NMR spectra of SA (400 MHz, $CDCl_3$). 106
Supplementary Figure 179. ^{1}H-NMR spectra of SA@DBU with molar ratio of structural units of SA to DBU is 5:1 (400 MHz, $CDCl_3$). 106
Supplementary Figure 180. ^{1}H-NMR spectra of SA@DBU with molar ratio of structural units of SA to DBU is 4:1 (400 MHz, $CDCl_3$). 107
Supplementary Figure 181. ^{1}H-NMR spectra of SA@DBU with molar ratio of structural units of SA to DBU is 3:1 (400 MHz, $CDCl_3$). 107
Supplementary Figure 182. ^{1}H-NMR spectra of SA@DBU with molar ratio of structural units of SA to DBU is 2:1 (400 MHz, $CDCl_3$). 108
Supplementary Figure 183. ^{1}H-NMR spectra of SA@DBU with molar ratio of structural units of SA to DBU is 1:1 (400 MHz, $CDCl_3$). 108
Supplementary Figure 184. ^{1}H-NMR spectra of SA@DBU with molar ratio of structural units of SA to DBU is 1:2 (400 MHz, $CDCl_3$). 109
Supplementary Figure 185. ^{1}H-NMR spectra of SA@DBU with molar ratio of structural units of SA to DBU is 1:3 (400 MHz, $CDCl_3$). 109
Supplementary Figure 186. ^{1}H-NMR spectra of SA@DBU with molar ratio of structural units of SA to DBU is 1:4 (400 MHz, $CDCl_3$). 110
Supplementary Figure 187. ^{1}H-NMR spectra of SA@DBU with molar ratio of structural units of SA to DBU is 1:5 (400 MHz, $CDCl_3$). 110
Supplementary Figure 188. ^{13}C-NMR spectra of DBU (100 MHz, $CDCl_3$). 111
Supplementary Figure 189. ^{13}C-NMR spectra of SA@DBU with molar ratio of structural units of SAS to DBU is 5:1 (100 MHz, $CDCl_3$). 111
Supplementary Figure 190. ^{13}C-NMR spectra of SA@DBU with molar ratio of structural units of SA to DBU is 4:1 (100 MHz, $CDCl_3$). 112
Supplementary Figure 191. ^{13}C-NMR spectra of SA@DBU with molar ratio of structural units of SA to DBU is 3:1 (100 MHz, $CDCl_3$). 112
Supplementary Figure 192. ^{13}C-NMR spectra of SA@DBU with molar ratio of structural units of SA to DBU is 2:1 (100 MHz, $CDCl_3$). 113
Supplementary Figure 193. ^{13}C-NMR spectra of SA@DBU with molar ratio of structural units of SA to DBU is 1:1 (100 MHz, $CDCl_3$). 113
Supplementary Figure 194. ^{13}C-NMR spectra of SA@DBU with molar ratio of structural units of SA to DBU is 1:2 (100 MHz, $CDCl_3$). 114
Supplementary Figure 195. ^{13}C-NMR spectra of SA@DBU with molar ratio of structural units of SA to DBU is 1:3 (100 MHz, $CDCl_3$). 114
Supplementary Figure 196. ^{13}C-NMR spectra of SA@DBU with molar ratio of structural units of SA to DBU is 1:4 (100 MHz, $CDCl_3$). 115
Supplementary Figure 197. ^{13}C-NMR spectra of SA@DBU with molar ratio of structural units of SA to DBU is 1:5 (100 MHz, $CDCl_3$). 115
Supplementary Figure 198. Time-dependent ^{1}H-NMR spectra of P1@TEA (400 MHz, $CDCl_3$). The molar ratio of structural units of P1 to TEA is 5:1. The h and d respectively represent hours and days. 116
Supplementary Figure 199. Time-dependent ^{13}C-NMR spectra of P1@TEA (100 MHz, $CDCl_3$). The molar ratio of structural units of P1 to TEA is 5:1. The h and d respectively represent hours and days. 116
Supplementary Figure 200. Time-dependent ^{1}H-NMR spectra of P2@TEA (400 MHz, $CDCl_3$). The molar ratio of structural units of P2 to TEA is 5:1. The h and d respectively represent hours and days. 117
Supplementary Figure 201. Time-dependent ^{13}C-NMR spectra of P2@TEA (100 MHz, $CDCl_3$). The molar ratio of structural units of P2 to TEA is 5:1. The h and d respectively represent hours and days. 117
Supplementary Figure 202. Time-dependent ^{1}H-NMR spectra of DS@TEA (400 MHz, $CDCl_3$). The molar ratio of structural units of DS to TEA is 5:1. The h and d respectively represent hours and days. 118
Supplementary Figure 203. Time-dependent ^{13}C-NMR spectra of DS@TEA (100 MHz, $CDCl_3$). The molar ratio of structural units of DS to TEA is 5:1. The h and d respectively represent hours and days. 118
Supplementary Figure 204. Time-dependent ^{1}H-NMR spectra of DC@TEA (400 MHz, $CDCl_3$). The molar ratio of structural units of DC to TEA is 5:1. The h and d respectively represent hours and days. 119
Supplementary Figure 205. Time-dependent ^{13}C-NMR spectra of DC@TEA (100 MHz, $CDCl_3$). The molar ratio of

structural units of DC to TEA is 5:1. The h and d respectively represent hours and days... 119
Theoretical calculation on P2@DBU and P2-DBU ***120***
Supplementary Figure 206. Frontier molecular orbitals of optimized excited-state geometries of P2@DBU and P2-DBU calculated by TD-DFT method at B3LYP-D3/6-31G(d,p) level, Gaussian 09 program. HOMO: the highest occupied molecular orbital, LUMO: the lowest unoccupied molecular orbital... 120
Supplementary Figure 207. Hole (red color) and electron (blue color) of optimized excited-state geometries of P2@DBU and P2-DBU calculated by TD-DFT method at B3LYP-D3/6-31G(d,p) level, Gaussian 09 program. 120
Photophysical characterization of amine-ester complexes ***121***
Supplementary Figure 208. PL spectra of (A) DS and (B) DC in bulk under different excitation wavelengths....... 121
Supplementary Figure 209. (A)~(D) Time-dependent PL spectra of DS@TEA in bulk under different excitation wavelengths. The molar ratio of structural units of DS to TEA is 640:1.. 121
Supplementary Figure 210. (A)~(I) Time-dependent PL spectra of DS@DBU in bulk under different excitation wavelengths. The molar ratio of structural units of DS to DBU is 640:1.. 122
Supplementary Figure 211. (A)~(I) Time-dependent PL spectra of DC@TEA in bulk under different excitation wavelengths. The molar ratio of structural units of DC to TEA is 640:1. 122
Supplementary Figure 212. (A)~(I) Time-dependent PL spectra of DC@DBU in bulk under different excitation wavelengths. The molar ratio of structural units of DC to DBU is 640:1. 123
Supplementary Figure 213. Plots of optimal emission wavelength versus molar ratios of DS or DC to TEA or DBU for DS@TEA, DS@DBU, DC@TEA and DC@DBU... 123
Supplementary Figure 214. (A)~(F) Molar ratios-dependent PL spectra of DS@DBU in bulk under different excitation wavelengths. The molar ratios of DS to DBU are from 640/1 to 20/1. 124
Supplementary Figure 215. (A)~(F) Molar ratios-dependent PL spectra of DC@TEA in bulk under different excitation wavelengths. The molar ratios of DC to TEA are from 640/1 to 20/1... 124
Supplementary Figure 216. (A)~(F) Molar ratios-dependent PL spectra of DC@DBU in bulk under different excitation wavelengths. The molar ratios of DC to DBU are from 640/1 to 20/1.. 125
Supplementary Figure 217. (A)~(I) Temperature-dependent PL spectra of DC@DBU with temperature from 77K to 177K in bulk under different excitation wavelengths. The molar ratio of DC to DBU is 20/1.................................... 125
Supplementary Figure 218. (A)~(I) Temperature-dependent PL spectra of DC@DBU with temperature from 197K to 297K in bulk under different excitation wavelengths. The molar ratio of DC to DBU is 20/1.................................... 126
Supplementary Figure 219. PL spectra of (A) P1, (B) P1@TEA and (C) P1@DBU in DCM solution of 10^{-1} M with molar ratios of ester units to amines are 1/2 under different excitation wavelengths.. 126
Supplementary Figure 220. PL spectra of (A) P1, (B) P1@TEA and (C) P1@DBU in solid with molar ratios of ester units to amines are 1/2 under different excitation wavelengths.. 127
Supplementary Figure 221. PL spectra of (A) P2, (B) P2@TEA and (C) P2@DBU in DCM solution of 10^{-1} M with molar ratios of ester units to amines are 1/2 under different excitation wavelengths.. 127
Supplementary Figure 222. PL spectra of (A) P2, (B) P2@TEA and (C) P2@DBU in solid with molar ratios of ester units to amines are 1/2 under different excitation wavelengths.. 127
Photophysical characterizaion of amine-initiated polyesters after eluting terminal amines ***128***
Supplementary Figure 223. PL spectra of P1-5.0DBU in solid precipitated in ethanol under different excitation wavelengths. Treatment 0, 1, 2 respectively represent once precipitated treatment without hydrochloric acid, once and twice precipitated treatment under hydrochloric acid of 37 *wt*% (5 drops of hydrochloric acid for per precipitation). 128
Supplementary Figure 224. PL spectra of P2-5.0DBU in solid precipitated in ethanol under different excitation wavelengths. Treatment 0, 1, 2 respectively represent once precipitated treatment without hydrochloric acid, once and twice precipitated treatment under hydrochloric acid of 37 *wt*% (5 drops of hydrochloric acid for per precipitation). 128
Supplementary Figure 225. PL spectra of P1-5.0TEA in solid precipitated in ethanol under different excitation wavelengths. TreatmentTreatment 0, 1, 2 represent precipitated treatment without hydrochloric acid, once and twice precipitated treatment under hydrochloric acid of 37 *wt*% (5 drops of hydrochloric acid for per precipitation). 128
Supplementary Figure 226. PL spectra of P2-5.0TEA in solid precipitated in ethanol under different excitation wavelengths. Treatment 0, 1, 2 respectively represent once precipitated treatment without hydrochloric acid, once and twice precipitated treatment under hydrochloric acid of 37 wt% (5 drops of hydrochloric acid for per precipitation). 129
Supplementary Table 1. Summary of physical properties of P1 and P1-amines **130**
Supplementary Table 2. Summary of physical properties of P2 and P2-amines **131**
Supplementary Reference **131**

Supplementary Methods

Materials

All manipulations involving air- and/or water-sensitive compounds were carried out with the standard Schlenk and vacuum line techniques under an argon atmosphere or in a nitrogen-filled glovebox. Succinic anhydride (SA), citraconic anhydride (CA) were purchased from Macklin. Propylene epoxide (PO)was purchased from Aldrich. Chromatographic purity solvents including toluene, dichloromethane, tetrahydrofuran (THF), ethanol and flaky sodium hydroxide (CaH_2) were bought from SINOPHARM. Triethyl borane (TEB) in tetrahydrofuran solution (1.0 mol/L), Dimethyl succinate (DS) and dimethyl citraconate (DC) were purchased from Tokyo Chemical Industry (TCI). Triethylamine (TEA) was purchased from Aldrich, and 1,8-diazabicyclo[5.4.0]undec-7-ene (DBU), 7-methyl-1,5,7-triazabicyclo[4.4.0]dec-5-ene (mTBD) and 1,5,7-triazabicyclo[4.4.0]dec-5-ene (TBD) were purchased from TCI. PO, TEA, DBU, DS, DC and toluene were refluxed over CaH_2 for 24 h and vacuum-distilled prior to use. Especially, chloroform-*d* for dynamic NMR characterization of amine-ester complexes was refluxed over CaH_2 for 24 h and distilled prior to use. Both the anhydrides (SA and CA) were purified by vacuum sublimation for three times. The purified anhydrides were collected under inert atmosphere and stored in the glovebox for use. Other organic reagents were used without purification.

Measurement

The polyester samples were characterized by nuclear magnetic resonance (^{1}H NMR and ^{13}C NMR), gel permeation chromatograph (GPC), fluorescence spectrophotometer, and UV-vis spectrophotometer. NMR spectra were recorded on a Bruker 400 MHz instrument in $CDCl_3$ by using tetramethylsilane (TMS) as an internal reference. The ester unit content was calculated based on the integral area of the protons (^{1}H NMR) of the ester unit($A_{5.0\sim5.5}$+$A_{4.2\sim4.5}$) to all repeated units (ester and ether, $A_{5.0\sim5.5}$+$A_{4.2\sim4.5}$+$A_{3.5}$) of the polyesters, i.e., Ester (%) = ($A_{5.0\sim5.5}$+$A_{4.2\sim4.5}$)/($A_{5.0\sim5.5}$+$A_{4.2\sim4.5}$+$A_{3.5}$), A is the integral area of protons at certain chemical shift. The numbers of scan of ^{13}C NMR for **P2-amines** were set to four times (2048). The number-averaged molecular weight (M_n) and polydispersity index (PDI) were obtained using a PL-GPC 220 chromatograph (Polymer Laboratories, Ltd., Santa Clara/United States) equipped with an HP 1100 pump from Agilent Technologies (Shanghai, China) and differential refractive index (dRI) detectors. The columns (4 x PLgel 5 μm MIXED-C, 300 x 7.5 mm) were eluted using chromatographic purity THF with 1.0 mL/min at 40 °C. The standards weremonodispersed polystyrene covering the molecular weight from 200 to 2,000,000 Da. The standards were monodispersed polystyrene covering the molecular weight from 1000 to 10,000,000 Da. The copolymerizedcopolymeriz structureswere characterized by matrix-assisted laser desorption ionization-time of flight mass spectrometry (MALDI-TOF MS) from Bruker ultrafleXtreme. Sodium trifluoroacetatewasusedasthecationizationagent and trans-2-[3-(4-tert-Butylphenyl)-2-methyl-2-propenylidene] malononitrile (DCTB) was used as matrix. Copolymerizationwas characterized by electrospray ionization quadrupole time-of-flight mass spectrometry (ESI QTOF MS) recorded on G6545, Agilent. Photoluminescence spectra were collected from a FS5 transient fluorescence spectrometer (Edinburgh Instruments) with an excitation source of xenon lamp and variable temperature device (The temperature is set from 77K to 363K). Absolute quantum yields were obtained by additional integrating sphere. The fluorescence decay curves were recorded on an Instruments C11367 transient spectrometer. UV-Vis

absorption spectra were recorded on UV-vis spectrophotometer of Cary 100. The Photos were recorded by a Cannon EOS 90D.

Synthesis of P1

In a glovebox, Zn-Co (III) DMCC (2.0 mg), SA (800 mg, 8.0 mmol) and toluene (2.0 mL) were measured into one 10.0 mL vial equipped with a Teflon-coated stir bar. PO (0.28 mL, 4.0 mmol) was added via a syringe, washing all solids into one vial. The vial was sealed with a Teflon-lined cap, removed from the glovebox, and immersed into an oil-bath preheated to 80 °C. After 72 h reaction, the solution became viscous, colorless and transparent. The vial was then removed from the heat block and cooled to room temperature. The mixtures of the vial was dissolved in 2 mL DCM and then put into ca. 50 mL analytical ethanol (without PL response) to precipitate the products. The dissolve-precipitation process was repeated for four times to remove unreacted anhydrides and catalysts. The colorless polymers of **P1** were finally collected and dried in vacuum at 40 °C for 12 h.

For **P1**, the conversion of PO is 99% and the yield of ultimate white polymers is 94%. ^{1}H NMR ($CDCl_3$, 400MHz): δ/ppm: 5.13 (m, 1H), 3.97~4.20 (m, 2H), 2.63 (d, 4H), 1.23~1.25 (d, 3H). ^{13}C NMR ($CDCl_3$, 100MHz): δ/ppm: 172.08, 68.66, 66.29, 28.98,16.54. The M_n, M_w from GPC Curves are 10.8 *KDa* and 14.9 *KDa*. PDI is 1.38.

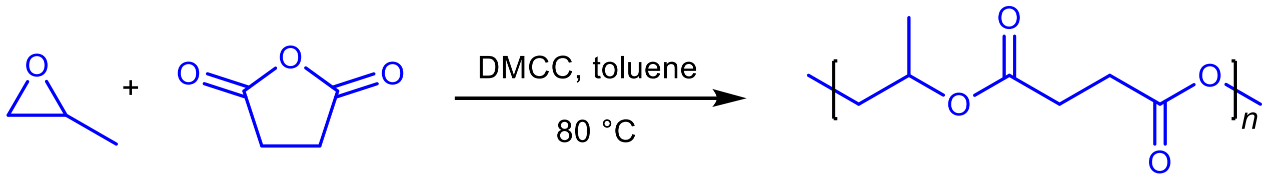

Supplementary Figure 1. Synthetic routes to **P1.**

Synthesis of P2

In a glovebox, Zn-Co (III) DMCC (2.0 mg), CA (0.74 mL, 8.0 moL) and toluene (2.0 mL) were measured into one 10.0 mL vial equipped with a Teflon-coated stir bar. PO (0.28 mL, 4.0 mmol) was added via a syringe, washing all solids into one vial. The vial was sealed with a Teflon-lined cap, removed from the glovebox, and immersed into an oil-bath preheated to 80 ℃. After 72 h reaction, the solution became viscous, light-yellow and transparent. The vial was then removed from the heat block and cooled to room temperature. The mixtures of the vial was dissolved in 2 mL DCM and then put into ca. 50 mL analytical ethanol (without PL response) to precipitate the products. The dissolve-precipitation process was repeated for four times to remove unreacted anhydrides and catalysts. The yellow polymers of **P2** were finally collected and dried in vacuum at 40 ℃ for 12 h.

For **P2**, the conversion of PO is 98% and the yield of ultimate yellow polymers is 96%. ^{1}H NMR ($CDCl_3$, 400MHz): δ/ppm: 5.85 (s, 1H), 5.13~5.33 (m, 1H), 4.14~4.34 (m, 2H), 2.04 (s, 3H), 1.24~1.36 (m, 3H). ^{13}C NMR ($CDCl_3$, 100MHz): δ/ppm: 168.27, 164.10, 146.03, 121.78, 119.61, 69.00, 66.48, 20.33, 16.17. The M_n, M_w from GPC Curves are 10.4 *KDa* and 16.8 *KDa*. PDI is 1.62.

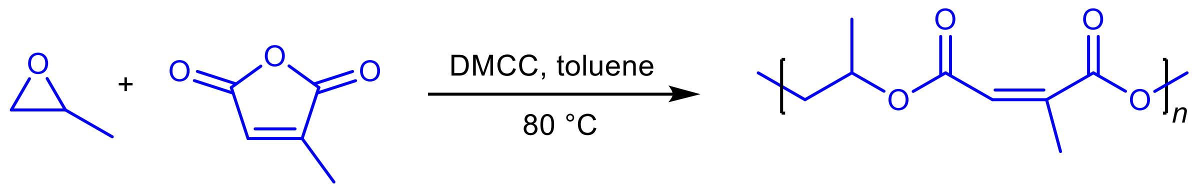

Supplementary Figure 2. Synthetic routes to **P2.**

Synthesis of P1-aTEA (a% are from 0.5% to 5.0%)

In a glovebox, PO (1.0 mL, 14.0 mmol), SA (400 mg, 4.0 mmol) were mixed into seven 10.0 mL vials equipped with Teflon-coated stir bars. Molar contents of TEB and triethylamine (TEA) to SA were from 0.5% to 5.0%, keeping molar ratios of TEB to TEA always at 1, and different-content TEB-TEA mixtures were measured into the seven vials after PO has completely dissolved SA, respectively. The seven vials were sealed with Teflon-lined caps, removed from the glovebox, and immersed into an oil-bath preheated to 60 °C. After 24 h reaction, the solution became viscous and brown-to-red. The seven vials were then removed from the heat block and cooled to room temperature. The mixtures of the vials were dissolved in 2 mL DCM and then put into ca. 50 mL analytical ethanol (without PL response) to precipitate the products. The dissolve-precipitation process was repeated for twice times to remove unreacted monomers. The brown-to-red polymers of **P1-aTEA** were finally collected and dried in vacuum at 40 °C for 12 h.

For **P1-0.5TEA**, the conversion of SA is ~99% and the yield of ultimate white polymers is ~98%. ^{1}H NMR ($CDCl_3$, 400MHz): δ/ppm: 5.10 (m, 1H), 4.01~4.20 (m, 2H), 2.59 (d, 4H), 1.18~1.25 (d, 3H). ^{13}C NMR ($CDCl_3$, 100MHz): δ/ppm: 171.97, 68.60, 66.22, 28.92,16.45. The M_n, M_w from GPC Curves are 5.2 *KDa* and 7.3 *KDa*. PDI is 1.38.

For **P1-1.0TEA**, the conversion of SA is ~99% and the yield of ultimate white polymers is ~98%. ^{1}H NMR ($CDCl_3$, 400MHz): δ/ppm: 5.14 (m, 1H), 4.05~4.19 (m, 2H), 2.62 (d, 4H), 1.14~1.29 (d, 3H). ^{13}C NMR ($CDCl_3$, 100MHz): δ/ppm: 172.00, 68.93, 66.54, 29.50,16.77. The M_n, M_w from GPC Curves are 6.9 *KDa* and 11.6 *KDa*. PDI is 1.69.

For **P1-1.5TEA**, the conversion of SA is ~99% and the yield of ultimate white polymers is ~96%. ^{1}H NMR ($CDCl_3$, 400MHz): δ/ppm: 5.14 (m, 1H), 4.08~4.20 (m, 2H), 2.63~2.65 (m, 4H), 1.14~1.26 (d, 3H). ^{13}C NMR ($CDCl_3$, 100MHz): δ/ppm: 172.05, 68.64, 66.27, 29.22,16.49. The M_n, M_w from GPC Curves are 2.9 *KDa* and 5.9 *KDa*. PDI is 1.90.

For **P1-2.0TEA**, the conversion of SA is ~99% and the yield of ultimate white polymers is ~95%. ^{1}H NMR ($CDCl_3$, 400MHz): δ/ppm: 5.13 (m, 1H), 4.04~4.17 (m, 2H), 2.62 (d, 4H), 1.14~1.23 (d, 3H). ^{13}C NMR ($CDCl_3$, 100MHz): δ/ppm: 172.03, 68.62, 66.26, 29.21,16.48. The M_n, M_w from GPC Curves are 6.9 *KDa* and 10.9 *KDa*. PDI is 1.58.

For **P1-3.0TEA**, the conversion of SA is ~99% and the yield of ultimate white polymers is ~98%. ^{1}H NMR ($CDCl_3$, 400MHz): δ/ppm: 5.14 (m, 1H), 4.07~4.20 (m, 2H), 2.62 (m, 4H), 1.14~1.26 (m, 3H). ^{13}C NMR ($CDCl_3$, 100MHz): δ/ppm: 172.04, 73.61, 69.78, 68.62, 66.27, 29.22,16.49. The M_n, M_w from GPC Curves are 6.7 *KDa* and 10.0 *KDa*. PDI is 1.51.

For **P1-4.0TEA**, the conversion of SA is ~99% and the yield of ultimate white polymers is ~94%. ^{1}H NMR ($CDCl_3$, 400MHz): δ/ppm: 5.04~5.12 (d, 1H), 3.48~4.19 (m, 2H), 2.61 (d, 4H), 1.11~1.26 (m, 3H). ^{13}C NMR

$(CDCl_3$, 100MHz): δ/ppm: 172.37, 73.90, 70.06, 68.87, 66.54, 29.66,16.93. The M_n, M_w from GPC Curves are 7.4 *KDa* and 11.9 *KDa*. PDI is 1.60.

For **P1-5.0TEA**, the conversion of SA is ~99% and the yield of ultimate white polymers is ~92%. ^{1}H NMR ($CDCl_3$, 400MHz): δ/ppm: 5.06 (m, 1H), 3.44~3.52 (m, 2H), 2.61 (d, 4H), 1.21 (m, 3H). ^{13}C NMR ($CDCl_3$, 100MHz): δ/ppm: 171.87, 73.66, 69.75, 29.47,16.70. The M_n, M_w from GPC Curves are 18.2 *KDa* and 28.2 *KDa*. PDI is 1.55.

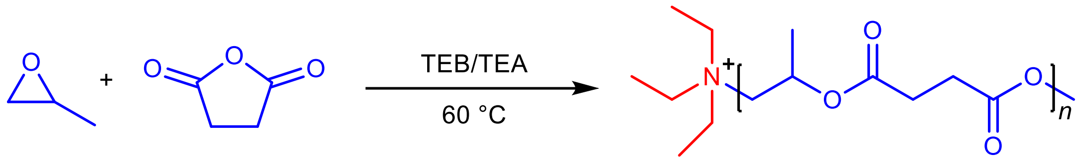

Supplementary Figure 3. Synthetic routes to **P1-aTEA.**

Synthesis of P1-2.0DBU, P1-2.0mTBD and P1P1-2.0TBD

In a glovebox, PO (1.0 mL, 14.0 mmol), SA (400 mg, 4.0 mmol) were mixed into three 10.0 mL vials equipped with Teflon-coated stir bars. Molar contents of TEB and amines (DBU, mTBD and TBD) to SA were 2.0%, keeping molar ratios of TEB to the amines always at 1, and different-content TEB-amine mixtures were measured into the three vials after PO has completely dissolved SA, respectively. The three vials were sealed with Teflon-lined caps, removed from the glovebox, and immersed into an oil-bath preheated to 60 °C. After 24 h reaction, the solution became viscous and brown-to-red. The three vials were then removed from the heat block and cooled to room temperature. The mixtures of the vials were dissolved in 2 mL DCM and then put into ca. 50 mL analytical ethanol (without PL response) to precipitate the products. The dissolve-precipitation process was repeated for twice times to remove unreacted monomers. The brown-to-red polymers of **P1-2.0DBU, P1-2.0mTBD and P1P1-2.0TBD** were finally collected and dried in vacuum at 40 °C for 12 h, respectively.

For **P1-2.0DBU**, the conversion of SA is ~99% and the yield of ultimate white polymers isis ~95%. ^{1}H NMR ($CDCl_3$, 400MHz): δ/ppm: 5.13 (m, 1H), 4.04~4.17 (m, 2H), 2.59~2.62 (m, 4H), 1.18~1.23 (d, 3H). ^{13}C NMR ($CDCl_3$, 100MHz): δ/ppm: 172.03, 68.62, 66.26, 28.95,16.48. The M_n, M_w from GPC Curves are 4.2 *KDa* and 7.8 *KDa*. PDI is 1.84.

For **P1-2.0mTBD**, the conversion of SA is ~99% and the yield of ultimate white polymers isis ~96%. ^{1}H NMR ($CDCl_3$, 400MHz): δ/ppm: 5.16 (m, 1H), 4.07~4.20 (m, 2H), 2.64 (d, 4H), 1.20~1.27 (d, 3H). ^{13}C NMR ($CDCl_3$, 100MHz): δ/ppm: 172.06, 68.66, 66.28, 29.24,16.52. The M_n, M_w from GPC Curves are 6.1 *KDa* and 13.6 *KDa*. PDI is 2.21.

For **P1-2.0TBD**, the conversion of SA are ~99% and the yield of ultimate white polymers isis ~92%. ^{1}H NMR ($CDCl_3$, 400MHz): δ/ppm: 5.11 (m, 1H), 4.08~4.17 (m, 2H), 2.59~2.67 (m, 4H), 1.22~1.24 (d, 3H). ^{13}C NMR ($CDCl_3$, 100MHz): δ/ppm: 172.05, 68.65, 66.28, 29.24,16.51. The M_n, M_w from GPC Curves are 4.1 *KDa* and 8.2 *KDa*. PDI is 2.01.

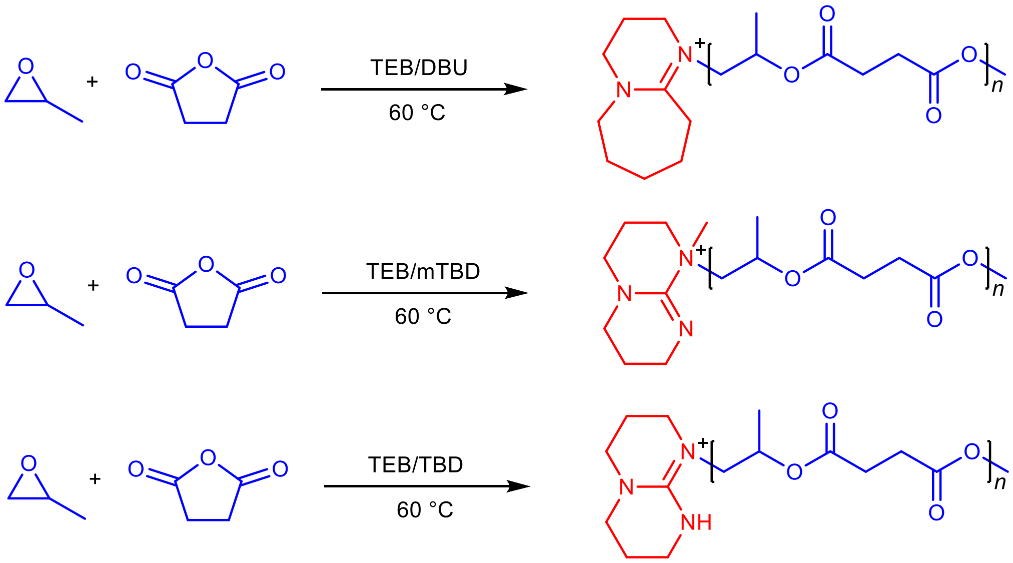

Supplementary Figure 4. Synthetic routes to **P1-2.0DBU, P1-2.0mTBD** and **P1-2.0TBD.**

Synthesis of P2-aTEA (a% are from 0.5% to 5.0%)

In a glovebox, PO (0.70 mL, 10.0 mmol), CA (0.81 mL, 9.0 mmoL) and toluene (1.0 mL) were measured into seven 10.0 mL vials equipped with Teflon-coated stir bars. Molar contents of TEB and triethylamine (TEA) to CA were from 0.5% to 5.0%, keeping molar ratios of TEB to TEA always at 1, and different-content TEB-TEA mixtures were measured into the seven vials, respectively. The seven vials were sealed with Teflon-lined caps, removed from the glovebox, and immersed into an oil-bath preheated to 60 °C. After 18 h reaction, the solution became viscous and purple-red. The seven vials were then removed from the heat block and cooled to room temperature. The mixtures of the vials were dissolved in 2 mL DCM and then put into ca. 50 mL analytical ethanol (without PL response) to precipitate the products. The dissolve-precipitation process was repeated for twice times to remove unreacted monomers. The brownbrown-to-red polymers of **P2-aTEA** were finally collected and dried in vacuum at 40 °C for 12 h.

For **P2-0.5TEA**, the conversion of CA isis ~99% and the yield of ultimate purple-red polymers isis ~96%. ^{1}H NMR ($CDCl_3$, 400MHz): δ/ppm: 5.82 (s, 1H), 5.09~5.23 (m, 1H), 4.02~4.17 (m, 2H), 2.04 (m, 3H), 1.15~1.27 (m, 3H). ^{13}C NMR ($CDCl_3$, 100MHz): δ/ppm: 168.77, 164.37, 129.98, 120.22, 70.99, 69.58, 66.91, 65.79, 20.84, 18.13, 16.41, 10.08. The M_n, M_w from GPC Curves are 3.9 *KDa* and 11.5 *KDa*. PDI is 2.91.

For **P2-1.0TEA**, the conversion of CA isis ~99% and the yield of ultimate purple-red polymers isis ~95%. ^{1}H NMR ($CDCl_3$, 400MHz): δ/ppm: 5.83 (s, 1H), 5.11~5.28 (m, 1H), 4.06~4.22 (m, 2H), 2.01~2.06 (m, 3H), 1.16~1.28 (m, 3H). ^{13}C NMR ($CDCl_3$, 100MHz): δ/ppm: 168.41, 164.33, 129.62, 119.93, 70.64, 68.55, 66.66, 65.42, 20.80, 18.49, 16.35, 9.74. The M_n, M_w from GPC Curves are 3.2 KDa and 6.5 KDa. PDI is 2.00.

For **P2-1.5TEA**, the conversion of CA isis ~99% and the yield of ultimate purple-red polymers isis ~93%. ^{1}H NMR ($CDCl_3$, 400MHz): δ/ppm: 5.83 (s, 1H), 5.10~5.20 (m, 1H), 4.03~4.22 (m, 2H), 2.01~2.05 (m, 3H), 1.15~1.23 (m, 3H). ^{13}C NMR (CDCl3, 100MHz): δ/ppm: 168.47, 164.40, 129.67, 120.10, 70.69, 69.30, 66.63, 65.46, 20.84, 18.51, 16.05, 9.78. The Mn, Mw from GPC Curves are 2.7 KDa and 7.5 KDa. PDI is 2.78.

For **P2-2.0TEA**, the conversion of CA isis ~99% and the yield of ultimate purple-red polymers isis ~94%. ^{1}H NMR ($CDCl_3$, 400MHz): δ/ppm: 5.85 (s, 1H), 5.12~5.24 (m, 1H), 4.10~4.24 (m, 2H), 2.03~2.07 (m, 3H),

1.17~1.23 (m, 3H). ^{13}C NMR (CDCl3, 100MHz): δ/ppm: 168.32, 163.76, 129.47, 125.16, 70.51, 69.15, 66.46, 65.30, 20.38, 16.20, 11.34, 8.41. The Mn, Mw from GPC Curves are 2.9 KDa and 8.6 KDa. PDI is 2.95.

For **P2-3.0TEA**, the conversion of CA isis ~99% and the yield of ultimate purple-red polymers isis ~96%. ^{1}H NMR ($CDCl_3$, 400MHz): δ/ppm: 5.87 (s, 1H), 5.10~5.28 (m, 1H), 4.01~4.20 (m, 2H), 2.01~2.06 (m, 3H), 1.16~1.21 (m, 3H). ^{13}C NMR (CDCl3, 100MHz): δ/ppm: 169.18, 164.70~166.06, 130.00, 120.37, 70.99, 68.96, 66.06, 65.83, 21.18, 18.85, 16.34, 10.12. The Mn, Mw from GPC Curves are 2.4 *KDa* and 6.7 *KDa*. PDI is 2.79.

For **P2-4.0TEA**, the conversion of CA isis ~99% and the yield of ultimate purple-red polymers isis ~97%. ^{1}H NMR ($CDCl_3$, 400MHz): δ/ppm: 5.84 (s, 1H), 5.11~5.28 (m, 1H), 4.04~4.23 (m, 2H), 2.02~2.06 (m, 3H), 1.16~1.29 (m, 3H). ^{13}C NMR (CDCl3, 100MHz): δ/ppm: 167.96, 163.72~165.45, 129.37, 121.05, 69.63, 68.65, 66.18, 65.19, 20.26, 18.06, 15.88, 9.50. The Mn, Mw from GPC Curves are 1.7 KDa and 4.6 KDa. PDI is 2.78.

For **P2-5.0TEA**, the conversion of CA isis ~99% and the yield of ultimate purple-red polymers isis ~96%. 1H NMR (CDCl3, 400MHz): δ/ppm: 5.84 (s, 1H), 5.07~5.26 (m, 1H), 4.05~4.18 (m, 2H), 1.99~2.03 (m, 3H), 1.16~1.21 (m, 3H). ^{13}C NMR (CDCl3, 100MHz): δ/ppm: 168.09, 163.98~164.32, 129.64, 120.91, 69.05~70.61, 65.44~66.54, 26.22, 20.48, 16.32, 14.11, 9.73. The Mn, Mw from GPC Curves are 1.6 KDa and 3.90 KDa. PDI is 2.37.

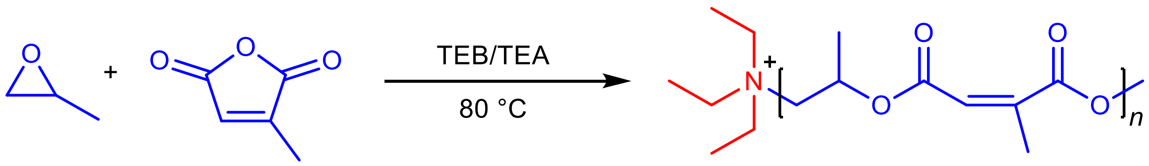

Supplementary Figure 5. Synthetic routes to **P2-aTEA.**

Synthesis of P2-2.0DBU, P2-2.0mTBD and P2-2.0TBD

In a glovebox, PO (0.70 mL, 10.0 mmol), CA (0.81 mL, 9.0 mmoL) and toluene (1.0 mL) were mixed into three 10.0 mL vials equipped with Teflon-coated stir bars. Molar contents of TEB and amines (DBU, mTBD and TBD) to CA were 2.0%, keeping molar ratios of TEB to the amines always at 1, and different-content TEB-amine mixtures were measured into the three vials, respectively. The three vials were sealed with Teflon-lined caps, removed from the glovebox, and immersed into an oil-bath preheated to 60 °C. After 18 h reaction, the solution became viscous and brown-to-red. The three vials were then removed from the heat block and cooled to room temperature. The mixtures of the vials were dissolved in 2 mL DCM and then put into ca. 50 mL analytical ethanol (without PL response) to precipitate the products. The dissolve-precipitation process was repeated for twice times to remove unreacted monomers. The brown-to-red polymers of **P2-2.0DBU, P2-2.0mTBD and P2-2.0TBD** were finally collected and dried in vacuum at 40 °C for 12 h, respectively.

For **P2-2.0DBU**, the conversion of CA isis ~99% and the yield of ultimate purple-red polymers isis ~95%. ^{1}H NMR ($CDCl_3$, 400MHz): δ/ppm: 5.86 (s, 1H), 5.09~5.27 (m, 1H), 4.03~4.21 (m, 2H), 2.00~2.04 (m, 3H), 1.14~1.22 (m, 3H). ^{13}C NMR (CDCl3, 100MHz): δ/ppm: 168.24, 164.34~165.86, 129.67, 120.13~121.06, 69.22~70.63, 65.44~66.62, 20.51, 18.49, 16.17, 9.78, 7.57. The Mn, Mw from GPC Curves are 4.8 KDa and 11.5 KDa. PDI is 2.40.

For **P2-2.0mTBD**, the conversion of CA isis ~99% and the yield of ultimate purple-red polymers isis ~94%. ^{1}H NMR ($CDCl_3$, 400MHz): δ/ppm: 5.81 (s, 1H), 5.07~5.21 (m, 1H), 4.04~4.16 (m, 2H), 1.99~2.03 (m, 3H), 1.09~1.23 (m, 3H). ^{13}C NMR ($CDCl_3$, 100MHz): δ/ppm: 168.11~168.76, 163.96~165.74, 146.73~149.24, 129.63, 68.97~70.55, 65.42~66.56, 18.14, 16.31, 13.91, 9.71. The M_n, M_w from GPC Curves are 8.4 KDa and 12.9 KDa. PDI is 1.53.

For **P2-2.0TBD**, the conversion of CA isis ~99% and the yield of ultimate purple-red polymers isis ~95%. ^{1}H NMR ($CDCl_3$, 400MHz): δ/ppm: 5.81 (s, 1H), 5.08~5.26 (m, 1H), 4.10~4.16 (m, 2H), 1.99~2.14 (m, 3H), 1.14~1.20 (m, 3H). ^{13}C NMR ($CDCl_3$, 100MHz): δ/ppm: 168.32, 163.76, 125.16~129.47, 69.15~70.51, 65.30~66.46, 20.38, 16.20, 11.34, 8.41. The M_n, M_w from GPC Curves are 6.9 KDa and 12.0 KDa. PDI is 1.74.

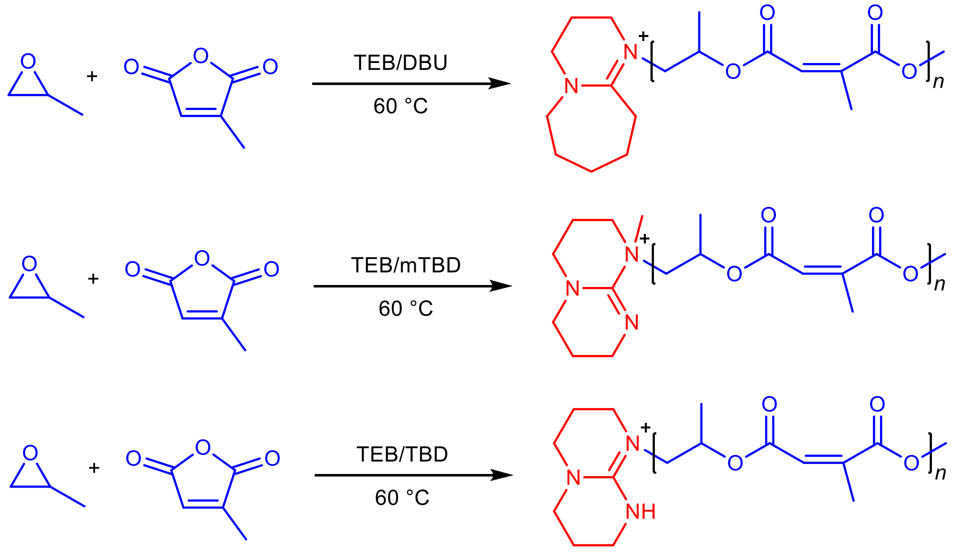

Supplementary Figure 6. Synthetic routes to **P2-2.0DBU, P2-2.0mTBD and P2-2.0TBD.**

Synthesis of P1-5.0DBU

In a glovebox, PO (1.0 mL, 14.0 mmol), SA (400 mg, 4.0 mmol) were mixed into one 10.0 mL vials equipped with a Teflon-coated stir bar. Molar content of TEB and DBU to SA was 5.0%, keeping molar ratios of TEB to the amines always at 1, and the TEB-DBU mixtures were measured into the vial after PO has completely dissolved SA, respectively. The vial was sealed and removed from the glovebox, and immersed into an oil-bath preheated to 60 °C. After 24 h reaction, the solution became viscous and brown-to-red. The vial was then removed from the heat block and cooled to room temperature. The mixtures of the vial were dissolved in 2 mL DCM and then put into ca. 50 mL analytical ethanol (without PL response) to precipitate the product. The dissolve-precipitation process was repeated for twice times to remove unreacted monomers. The red polymers of **P1-5.0DBU** were finally collected and dried in vacuum at 40 °C for 12 h, respectively. The conversion of SA is ~99% and the yield of ultimate red polymers is ~97%.

Synthesis of P2-5.0DBU

In a glovebox, PO (1.0 mL, 14.0 mmol), CA (0.81 mL, 9.0 mmoL) and toluene (1.0 mL) were mixed into one 10.0 mL vials equipped with a Teflon-coated stir bar. Molar content of TEB and DBU to SA was 5.0%, keeping molar ratios of TEB to the amines always at 1, and the TEB-DBU mixtures were measured into the vial,

respectively. The vial was sealed and removed from the glovebox, and immersed into an oil-bath preheated to 60 ℃. After 24 h reaction, the solution became viscous and brown-to-red. The vial was then removed from the heat block and cooled to room temperature. The mixtures of the vial were dissolved in 2 mL DCM and then put into ca. 50 mL analytical ethanol (without PL response) to precipitate the product. The dissolve-precipitation process was repeated for twice times to remove unreacted monomers. The red polymers of **P2-5.0DBU** were finally collected and dried in vacuum at 40 ℃ for 12 h, respectively. The conversion of CA is ~99% and the yield of ultimate red polymers is ~99%.

Synthesis of model molecules M1

In a glovebox, PO (0.14 mL, 2.0 mmol), SA (200mg, 2.0 mmoL) was mixed into one 4.0 mL vials equipped with a Teflon-coated stir bar. Molar contents of triethylborane (TEB) and TEA to SA were 100%, keeping molar ratios of TEB to the amines always at 1, and the TEB-DBU mixtures were measured into the vial, respectively. The vial was sealed and removed from the glovebox, and stirred at room temperature for 12h. Finally, the mixtures in the vial were directly dried in vacuum at room temperature for 2h and **M1** were obtained.

Synthesis of model molecules M2

In a glovebox, PO (0.14 mL, 2.0 mmol), CA (0.18 mL, 2.0 mmoL) was mixed into one 4.0 mL vials equipped with a Teflon-coated stir bar. Molar contents of triethylborane (TEB) and TEA to SA were 100%, keeping molar ratios of TEB to the amines always at 1, and the TEB-DBU mixtures were measured into the vial, respectively. The vial was sealed and removed from the glovebox, and stirred at room temperature for 12h. Finally, the mixtures in the vial were directly dried in vacuum at room temperature for 2h and **M2** were obtained.

Computational details

All the calculations were performed with the TD-DFT method at B3LYP/6-31(d) level using Gaussian 09 program[1]. The polymer-related calculations were replaced by the oligomers with three repeating units. The frontier molecular orbitals (FMO) of **P1@DBU**, **P1-DBU**, **P2@DBU** and **P2-DBU** were displayed using IQmol molecular viewer package (Isovalue: 0.05). Hole-electron analyses were calculated by Multiwfn 3.8[2,3], and were displayed by using Visual Molecular Dynamics (VMD) (Isovalue: 0.0005)[4].

Dynamic NMR Spectra of Complexation

P2@DBU: **P2** (17 mg, 0.10 mmol) and **DBU** (3 μL, 0.02mmol) were dissolved by 0.5 mL of anhydrous chloroform-d in clean glass bottles, then the solution in bottle was transferred into one NMR tube for ^{1}H- and ^{13}C-NMR characterization on a Bruker 400 MHz instrument by using tetramethylsilane (TMS) as an internal reference. The measurement times were at 1hour, 1day, 2days, 3days, 4days and 5days after dissolution, respectively. $\Delta\delta_H$ and $\Delta\delta_C$ represent the absolute value of chemical shift changes for hydrogen and carbon atoms on DBU.

DS@DBU: **DS** (13 μL, 0.1 mmol) and **DBU** (3 μL, 0.02mmol) were dissolved by 0.5 mL of anhydrous chloroform-d in clean glass bottles, then the solution in bottle was transferred into one NMR tube for

^{1}H- and ^{13}C-NMR characterization on a Bruker 400 MHz instrument by using tetramethylsilane (TMS) as an internal reference. The measurement times were at 1 hour, 1 day, 2 days, 3 days and 4 days after dissolution, respectively. $\Delta\delta_H$ and $\Delta\delta_C$ represent the absolute value of chemical shift changes for hydrogen and carbon atoms on DBU.

DC@DBU: **DC** (14 μL, 0.1 mmol) and **DBU** (3 μL, 0.02mmol) were dissolved by 0.5 mL of anhydrous chloroform-d in clean glass bottles, then the solution in bottle was transferred into one NMR tube for ^{1}H- and ^{13}C-NMR characterization on a Bruker 400 MHz instrument by using tetramethylsilane (TMS) as an internal reference. The measurement times were at 1 hour, 1 day, 2 days, 3 days and 4 days after dissolution, respectively. $\Delta\delta_H$ and $\Delta\delta_C$ represent the absolute value of chemical shift changes for hydrogen and carbon atoms on DBU.

CA@DBU: The different mixtures with various molar ratios of **CA** to **DBU** were dissolved by 0.5 mL of anhydrous chloroform-d in clean glass bottles, then the solution in bottle was transferred into NMR tubes for ^{1}H- and ^{13}C-NMR characterization on a Bruker 400 MHz instrument by using tetramethylsilane (TMS) as an internal reference. The molar ratios of CA to DBU were 5:0, 1:1, 1:5 and 0:5, respectively. $\Delta\delta_H$ and $\Delta\delta_C$ represent the absolute value of chemical shift changes for hydrogen and carbon atoms on DBU.

SA@DBU: The different mixtures with various molar ratios of **SA** to **DBU** were dissolved by 0.5 mL of anhydrous chloroform-d in clean glass bottles, then the solution in bottle was transferred into NMR tubes for ^{1}H- and ^{13}C-NMR characterization on a Bruker 400 MHz instrument by using tetramethylsilane (TMS) as an internal reference. The molar ratios of CA to DBU were 5:0, 5:1, 4:1, 3:1, 2:1, 1:1, 1:2, 1:3, 1:4, 1:5 and 0:5, respectively. $\Delta\delta_H$ and $\Delta\delta_C$ represent the absolute value of chemical shift changes for hydrogen and carbon atoms on DBU.

P1@TEA: **P1** (15.8 mg, 0.10 mmol) and **TEA** (2.8 μL, 0.02mmol) were dissolved by 0.5 mL of anhydrous chloroform-d in clean glass bottles, then the solution in bottle was transferred into one NMR tube for ^{1}H- and ^{13}C-NMR characterization on a Bruker 400 MHz instrument by using tetramethylsilane (TMS) as an internal reference. The measurement times were at 1hour, 1day, 2days, 3days, 4days and 5days after dissolution, respectively.

P2@TEA: **P2** (17 mg, 0.10 mmol) and **TEA** (2.8 μL, 0.02mmol) were dissolved by 0.5 mL of anhydrous chloroform-d in clean glass bottles, then the solution in bottles bottle was transferred into one NMR tube for ^{1}H- and ^{13}C-NMR characterization on a Bruker 400 MHz instrument by using tetramethylsilane (TMS) as an internal reference. The measurement times were at 1hour, 1day, 2days, 3days, 4days and 5days after dissolution, respectively.

DS@TEA: **DS** (13 μL, 0.10 mmol) and **TEA** (2.8 μL, 0.02mmol) were dissolved by 0.5 mL of anhydrous chloroform-d in clean glass bottles, then the solution in bottles bottle was transferred into one NMR tube for ^{1}H- and ^{13}C-NMR characterization on a Bruker 400 MHz instrument by using tetramethylsilane (TMS) as an internal reference. The measurement times were at 1 hour, 1 day, 2 days, 3 days and 4 days after dissolution, respectively.

DC@TEA: **DC** (14 μL, 0.10 mmol) and **TEA** (2.8 μL, 0.02mmol) were dissolved by 0.5 mL of anhydrous chloroform-d in clean glass bottles, then the solution in bottle was transferred into one NMR tube for ^{1}H- and ^{13}C-NMR characterization on a Bruker 400 MHz instrument by using tetramethylsilane (TMS) as an internal reference. The measurement times were at 1 hour, 1 day, 2 days, 3 days and 4 days after dissolution, respectively.

Supplementary Discussion

Nuclear Magnetic Resonance Spectra

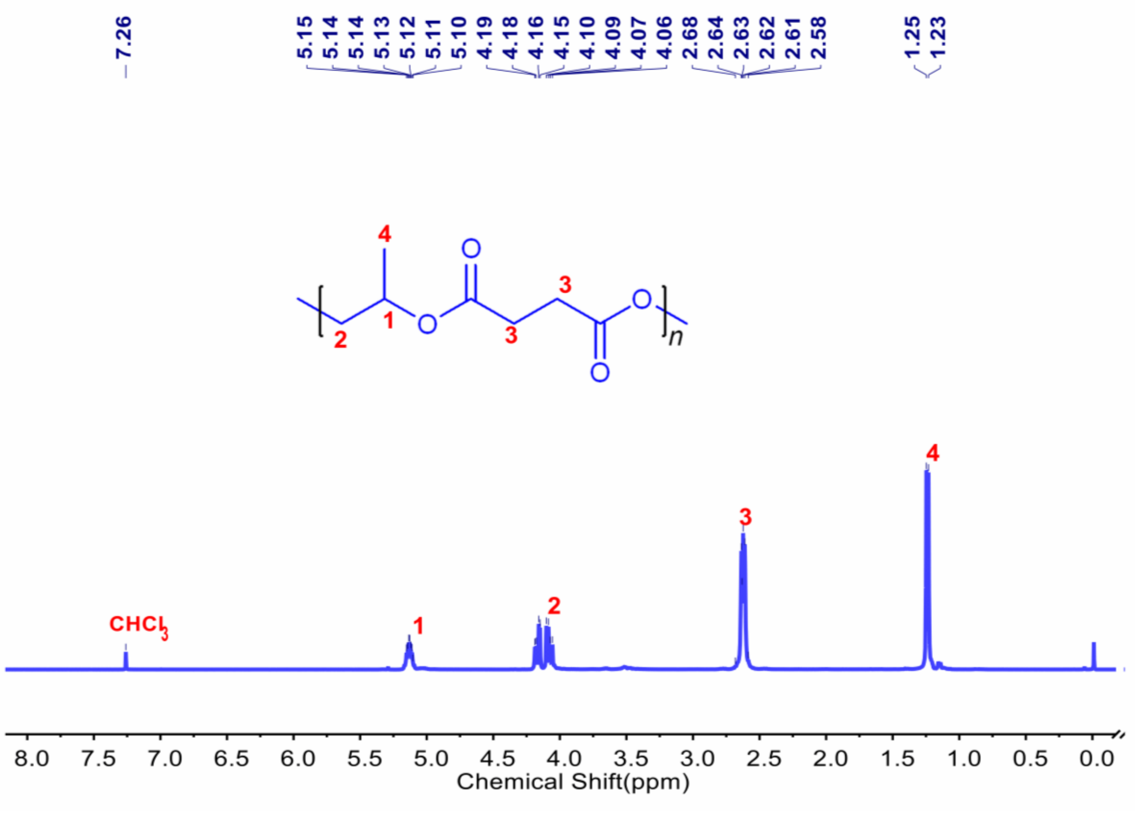

Supplementary Figure 7. ^{1}H NMR spectra of polyester **P1** (400 MHz, $CDCl_3$).

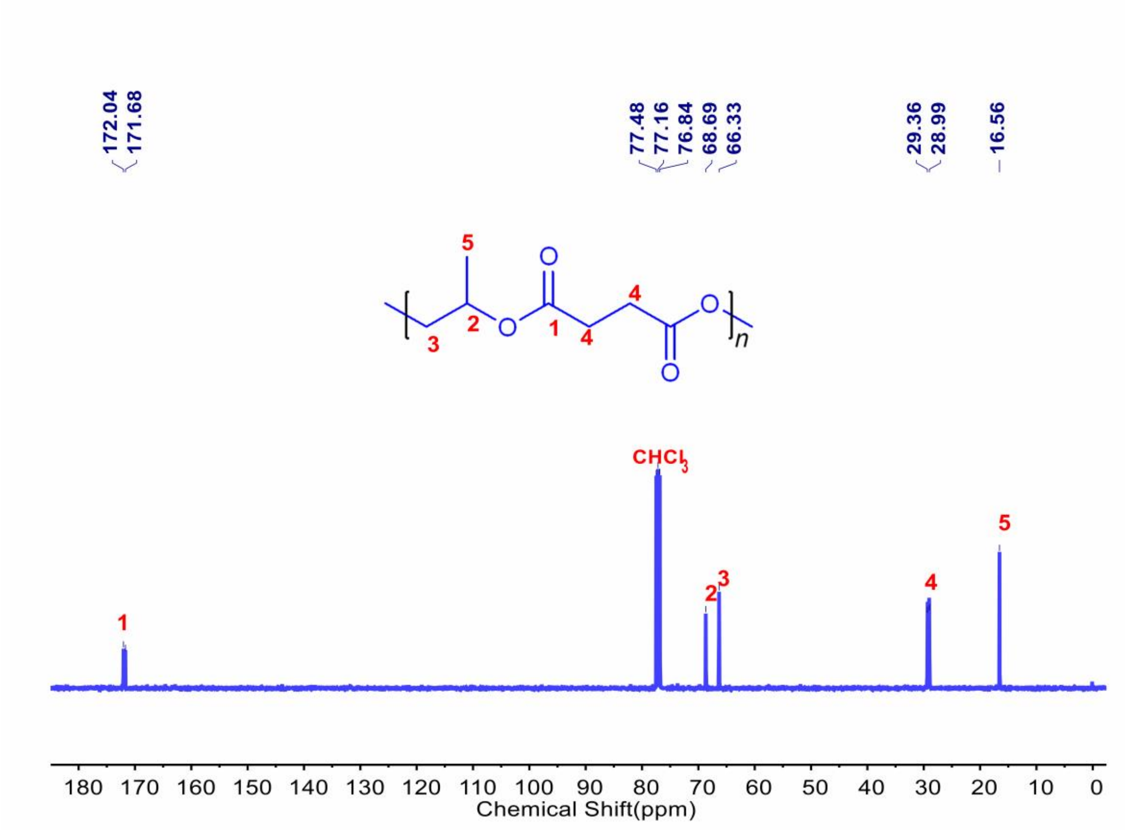

Supplementary Figure 8. ^{13}C NMR spectra of polyester **P1** (100 MHz, $CDCl_3$).

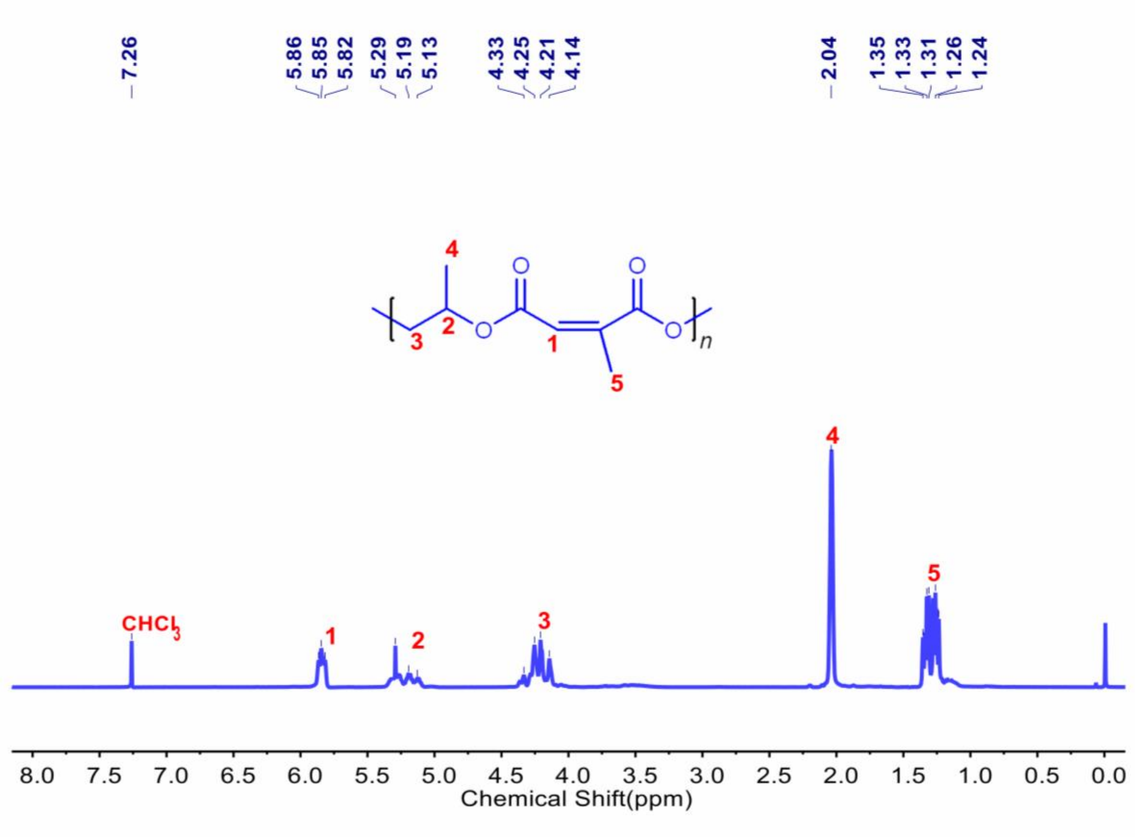

Supplementary Figure 9. ^{1}H NMR spectra of polyester **P2** (400 MHz, $CDCl_3$).

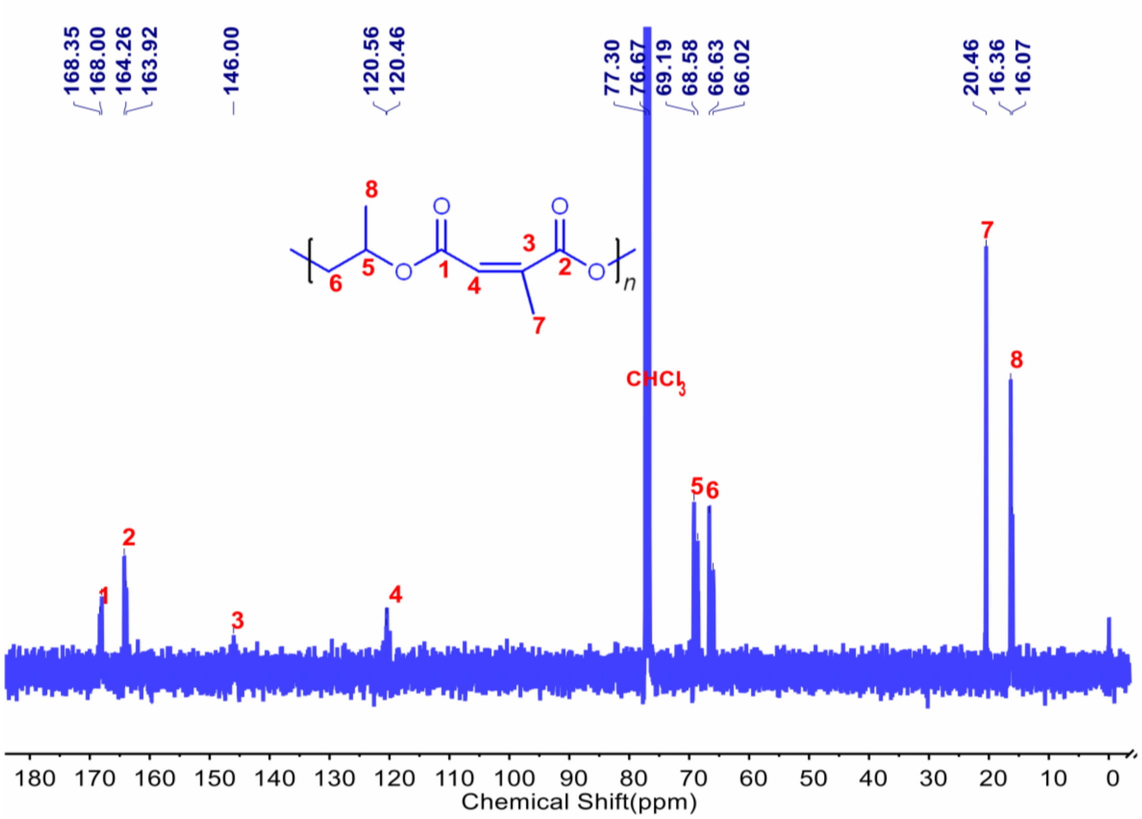

Supplementary Figure 10. ^{13}C NMR spectra of polyester **P2** (100 MHz, $CDCl_3$).

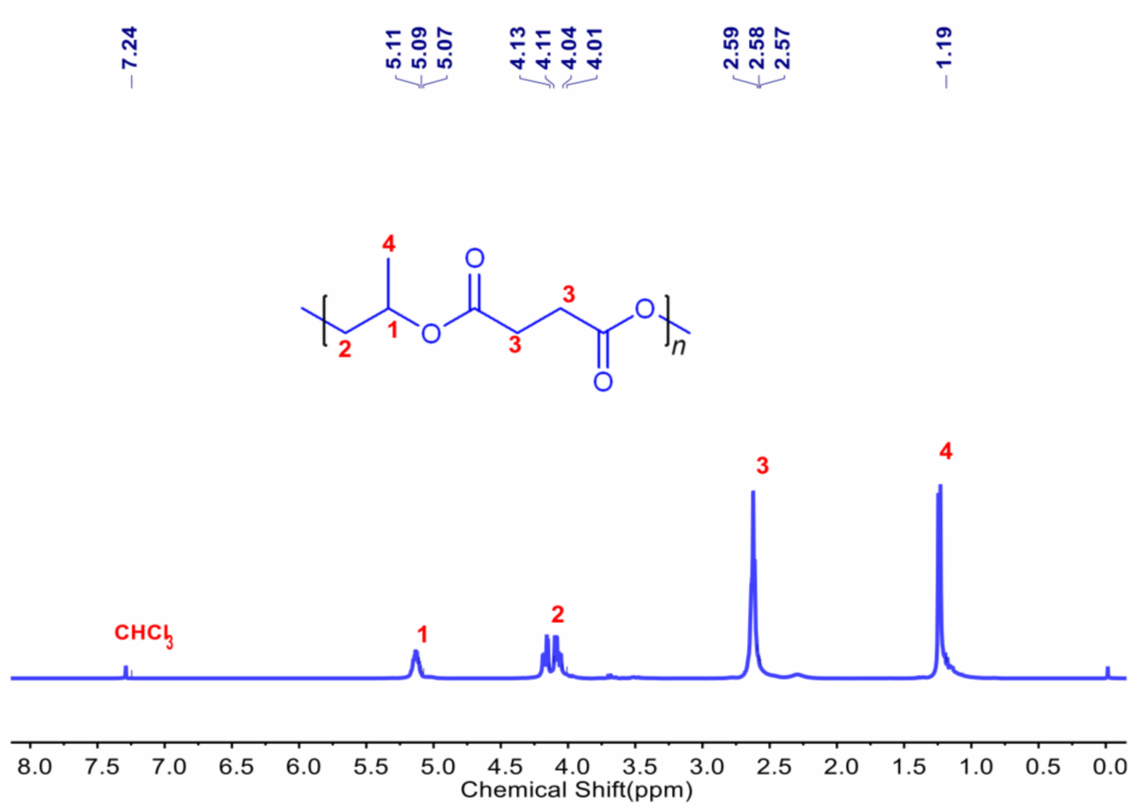

Supplementary Figure 11. ^{1}H NMR spectra of polyester **P1-0.5TEA** (400 MHz, $CDCl_3$).

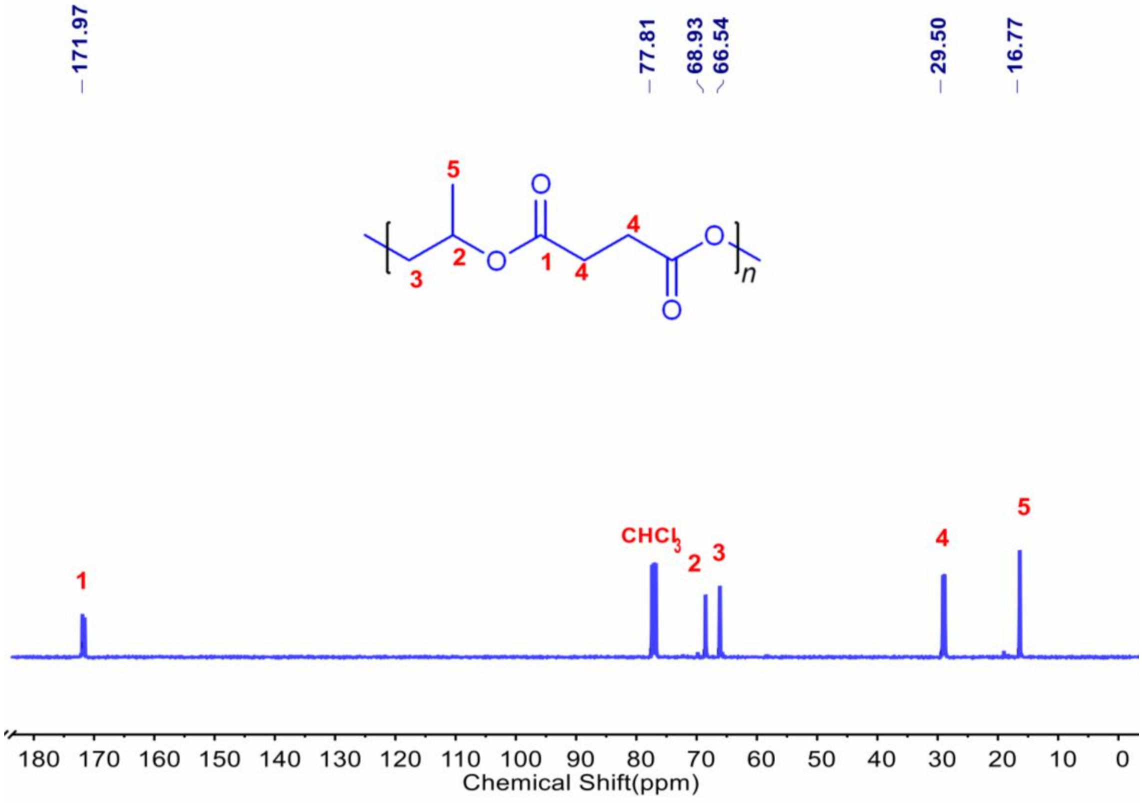

Supplementary Figure 12. ^{13}C NMR spectra of polyester **P1-0.5TEA** (100 MHz, $CDCl_3$).

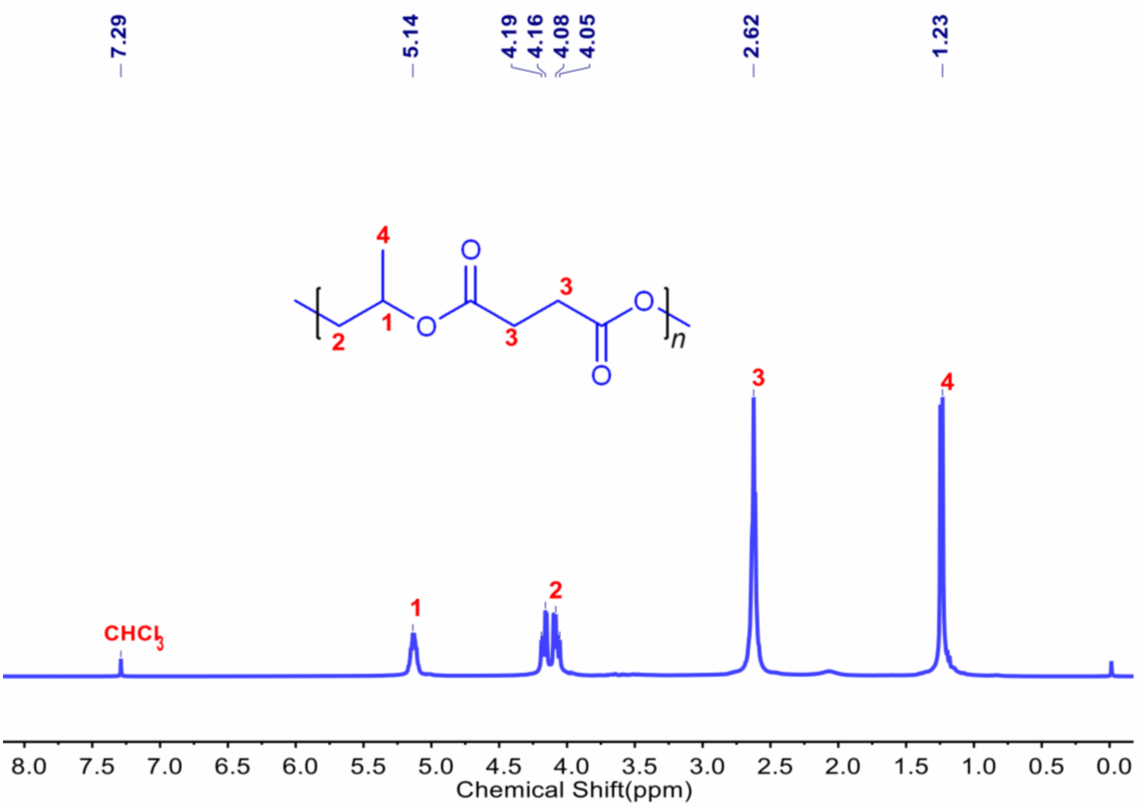

Supplementary Figure 13. ^{1}H NMR spectra of polyester **P1-1.0TEA** (400 MHz, $CDCl_3$).

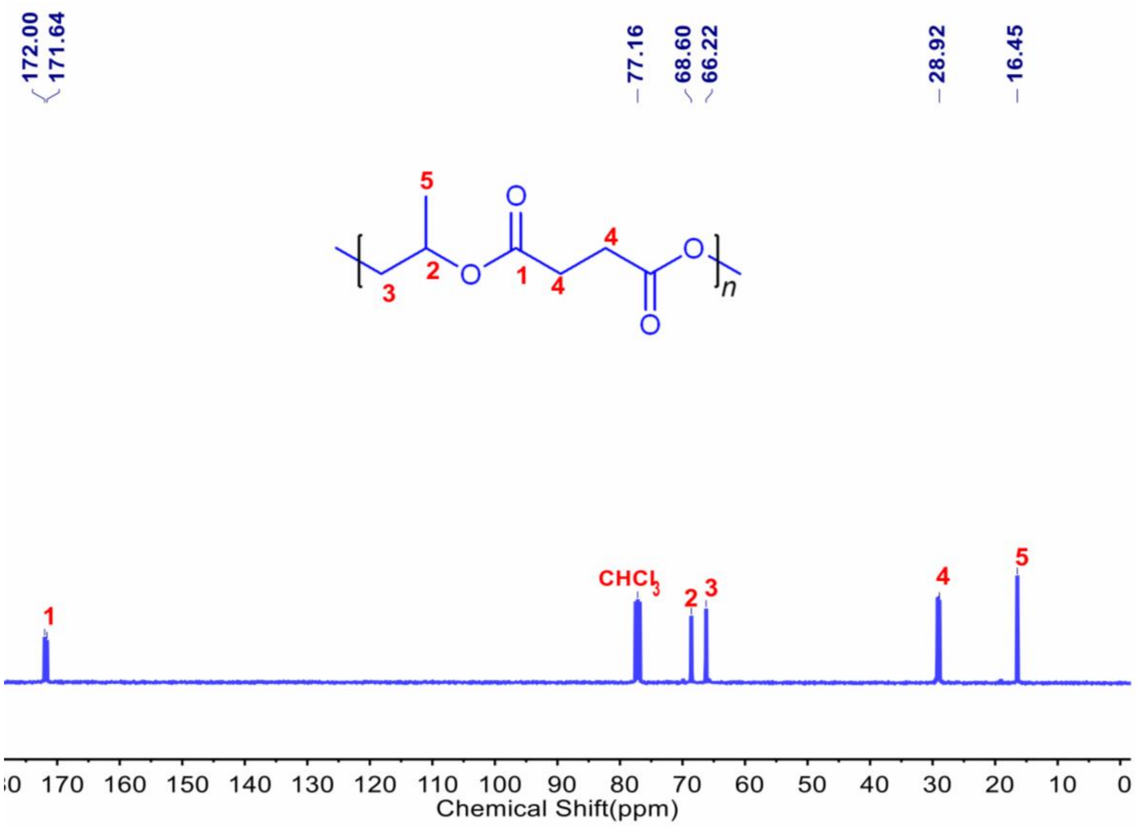

Supplementary Figure 14. ^{13}C NMR spectra of polyester **P1-1.0TEA** (100 MHz, $CDCl_3$).

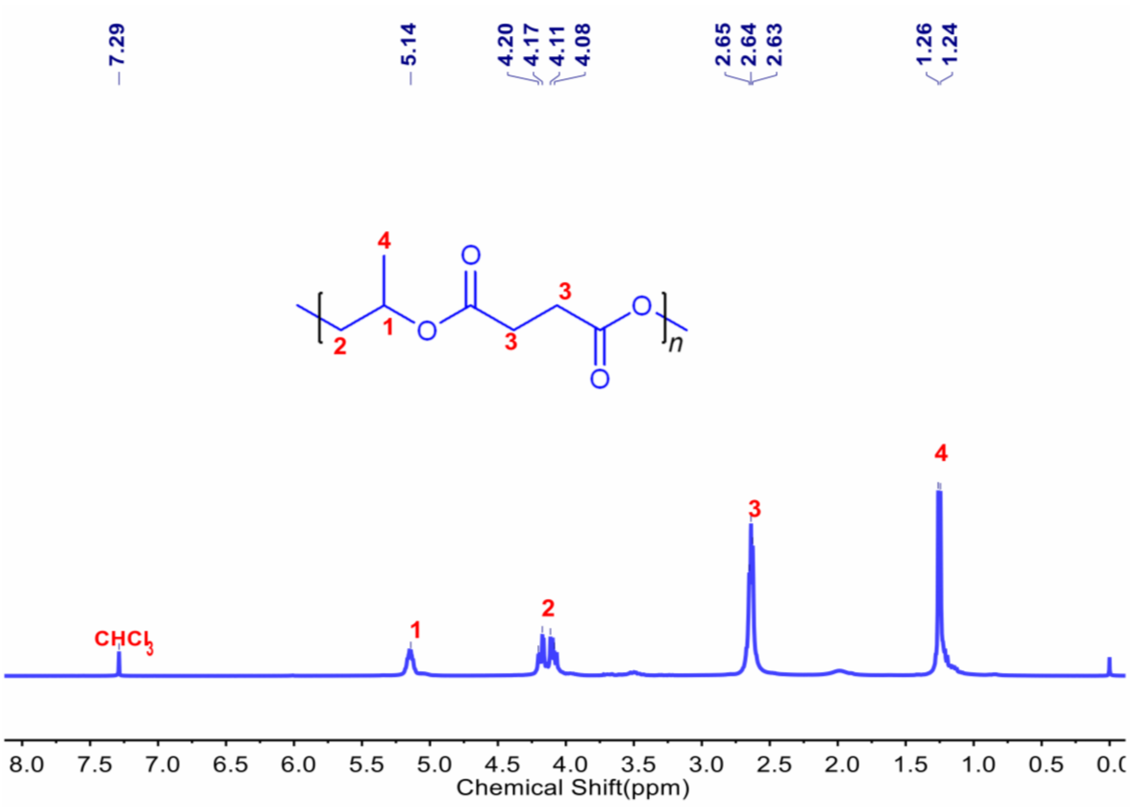

Supplementary Figure 15. ^{1}H NMR spectra of polyester **P1-1.5TEA** (400 MHz, $CDCl_3$).

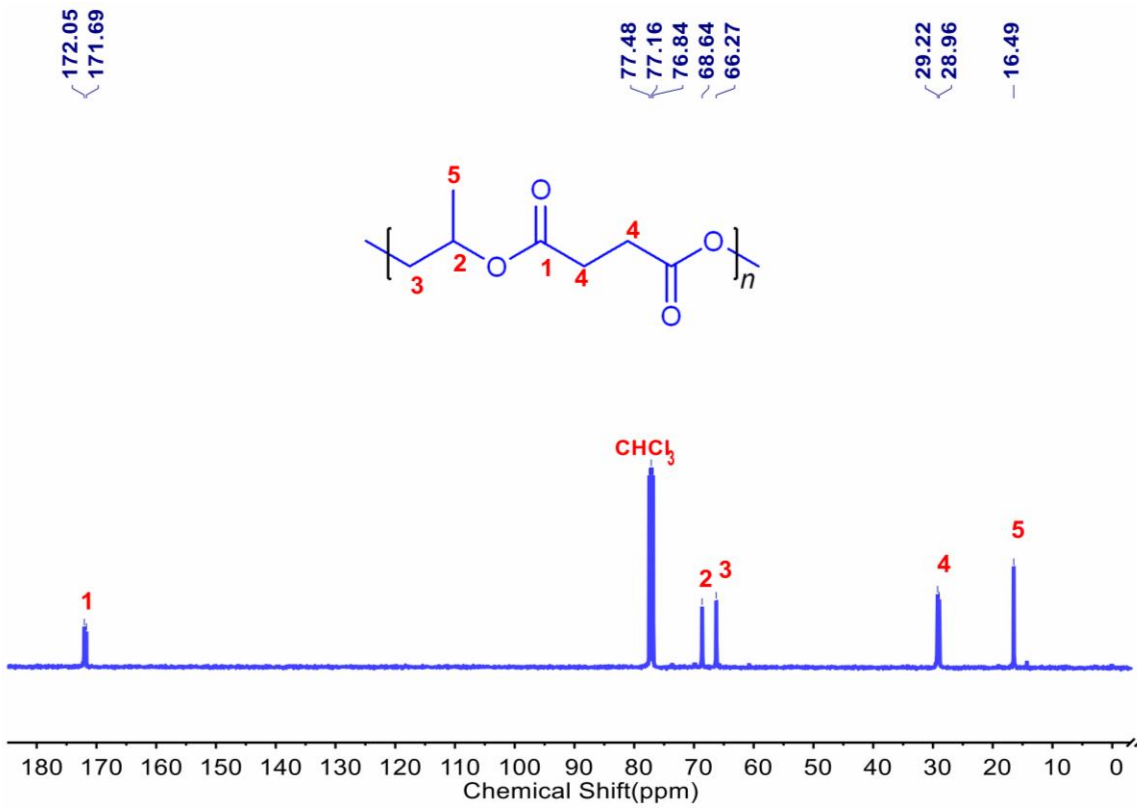

Supplementary Figure 16. ^{13}C NMR spectra of polyester **P1-1.5TEA** (100 MHz, $CDCl_3$).

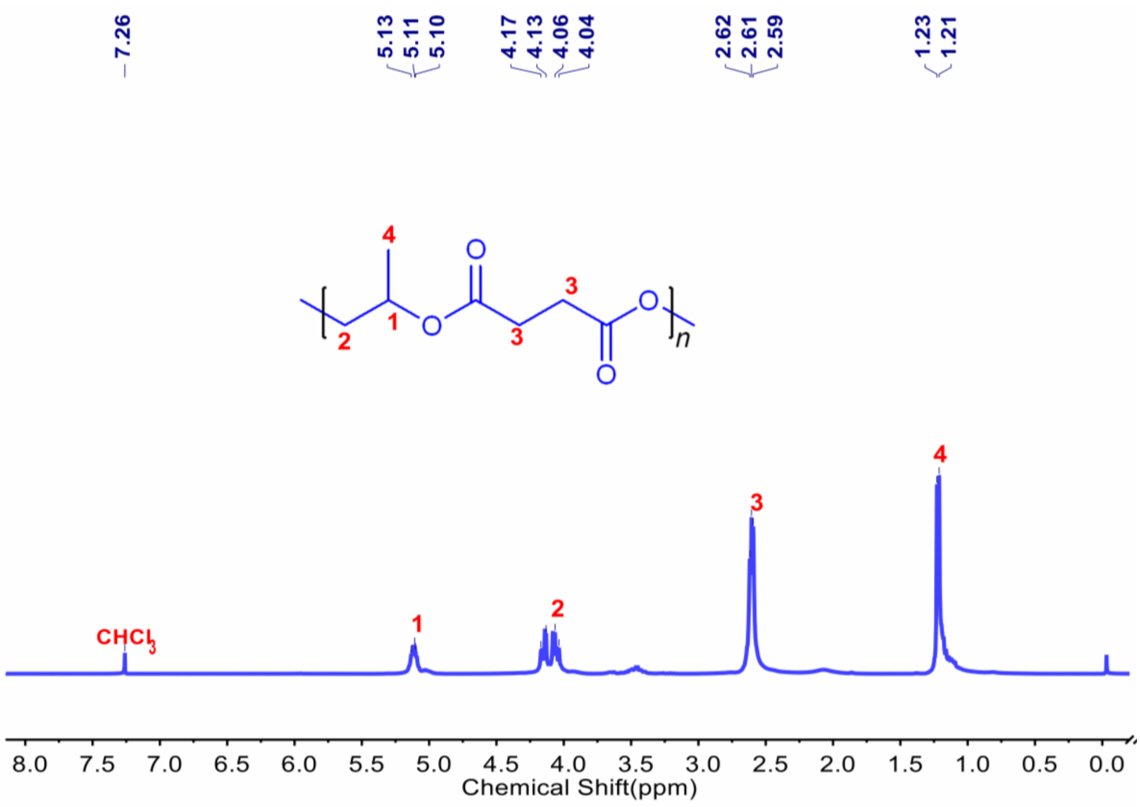

Supplementary Figure 17. ^{1}H NMR spectra of polyester **P1-2.0TEA** (400 MHz, $CDCl_3$).

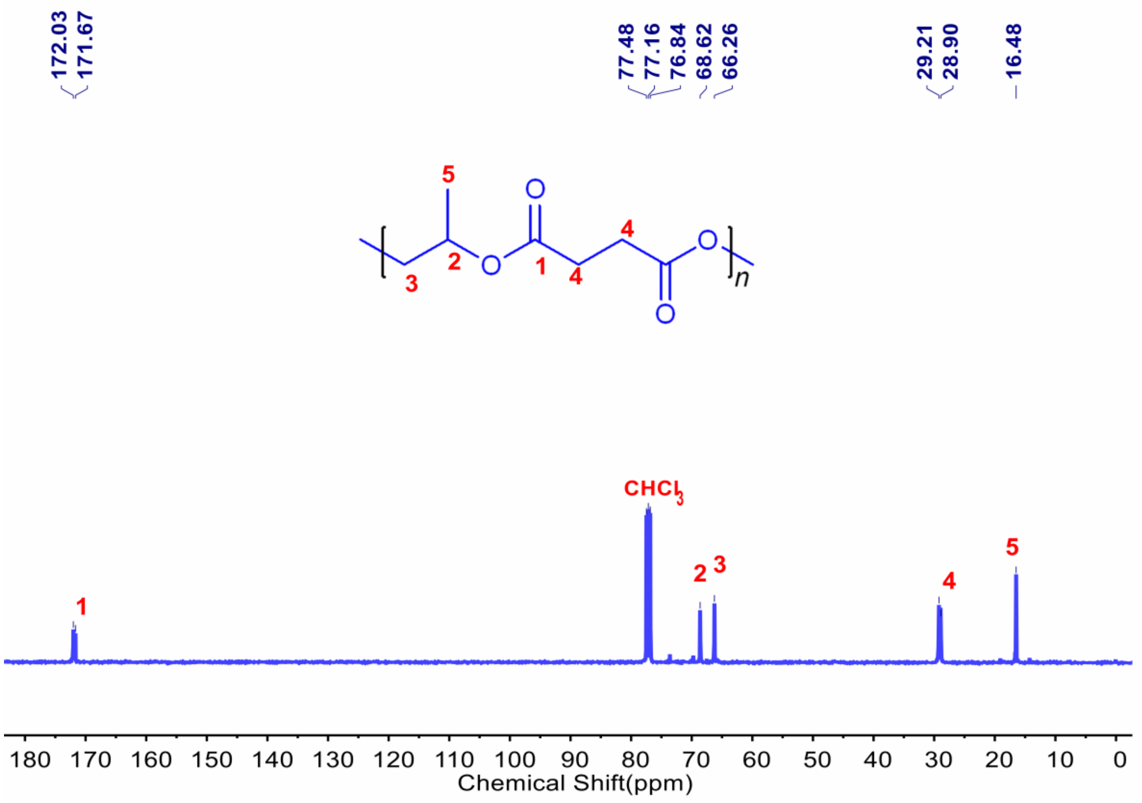

Supplementary Figure 18. ^{13}C NMR spectra of polyester **P1-2.0TEA** (100 MHz, $CDCl_3$).

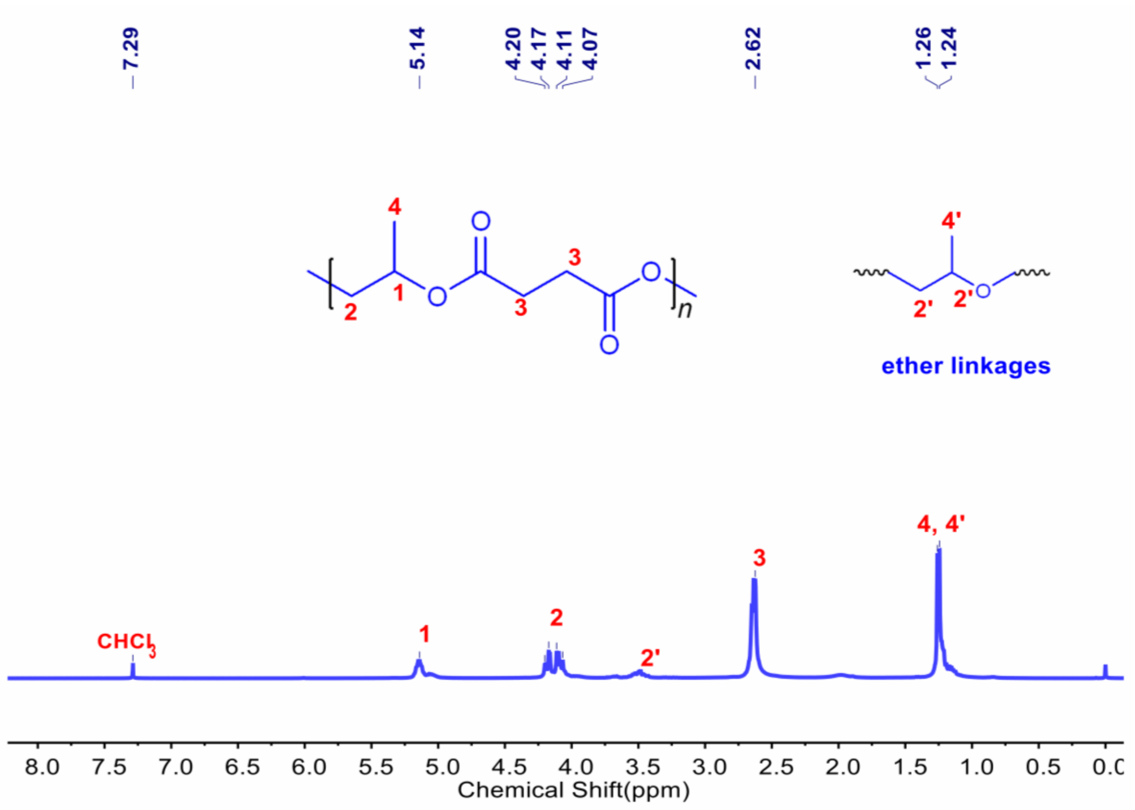

Supplementary Figure 19. ^{1}H NMR spectra of polyester **P1-3.0TEA** (400 MHz, $CDCl_3$).

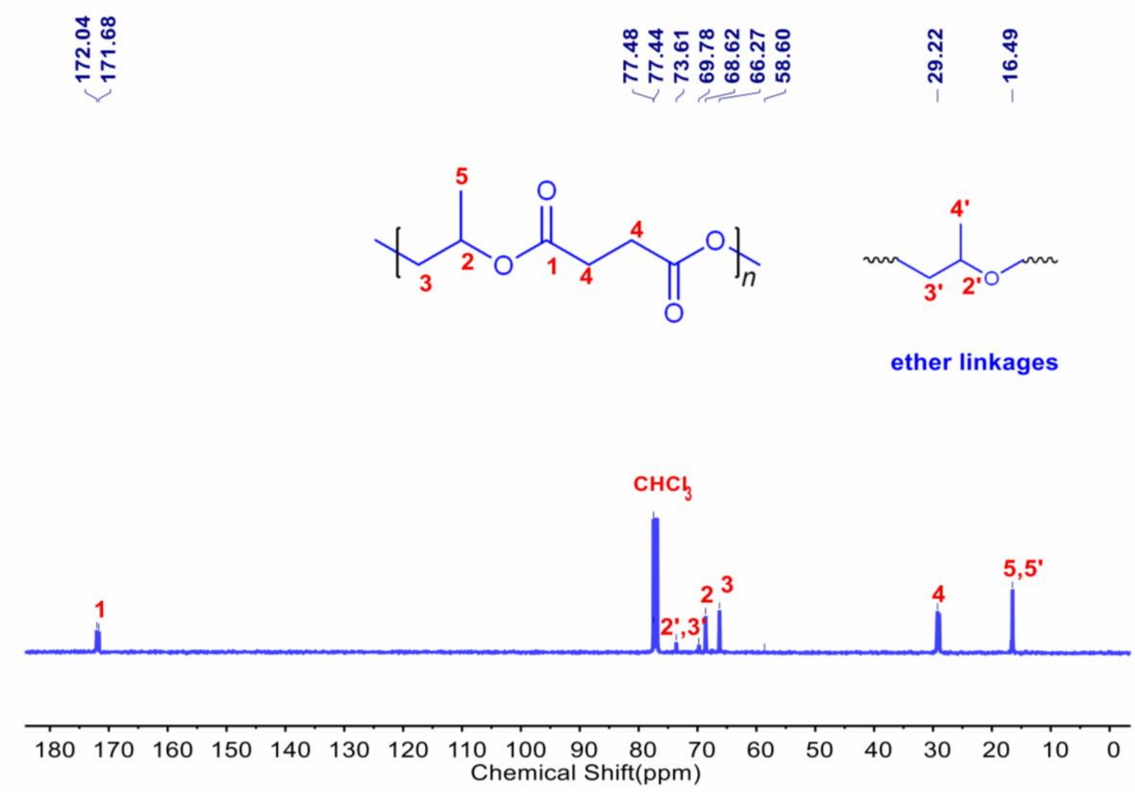

Supplementary Figure 20. ^{13}C NMR spectra of polyester **P1-3.0TEA** (100 MHz, $CDCl_3$).

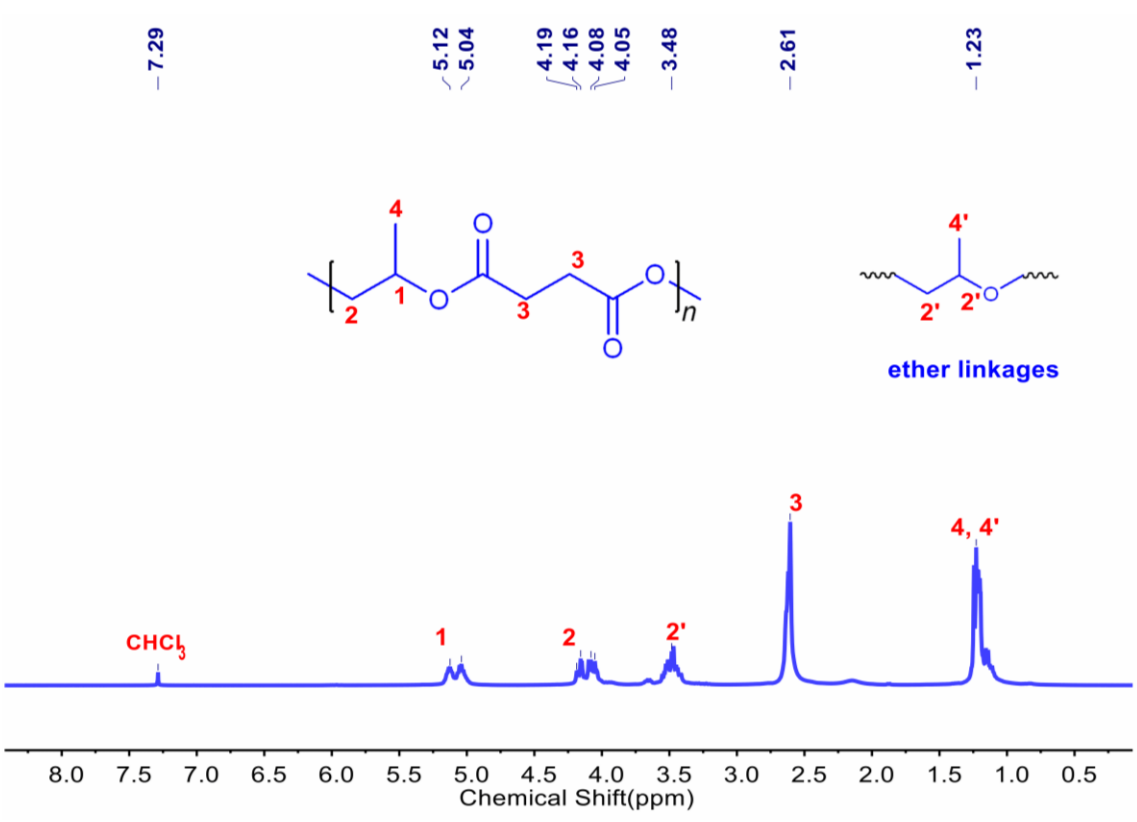

Supplementary Figure 21. ^{1}H NMR spectra of polyester **P1-4.0TEA** (400 MHz, $CDCl_3$).

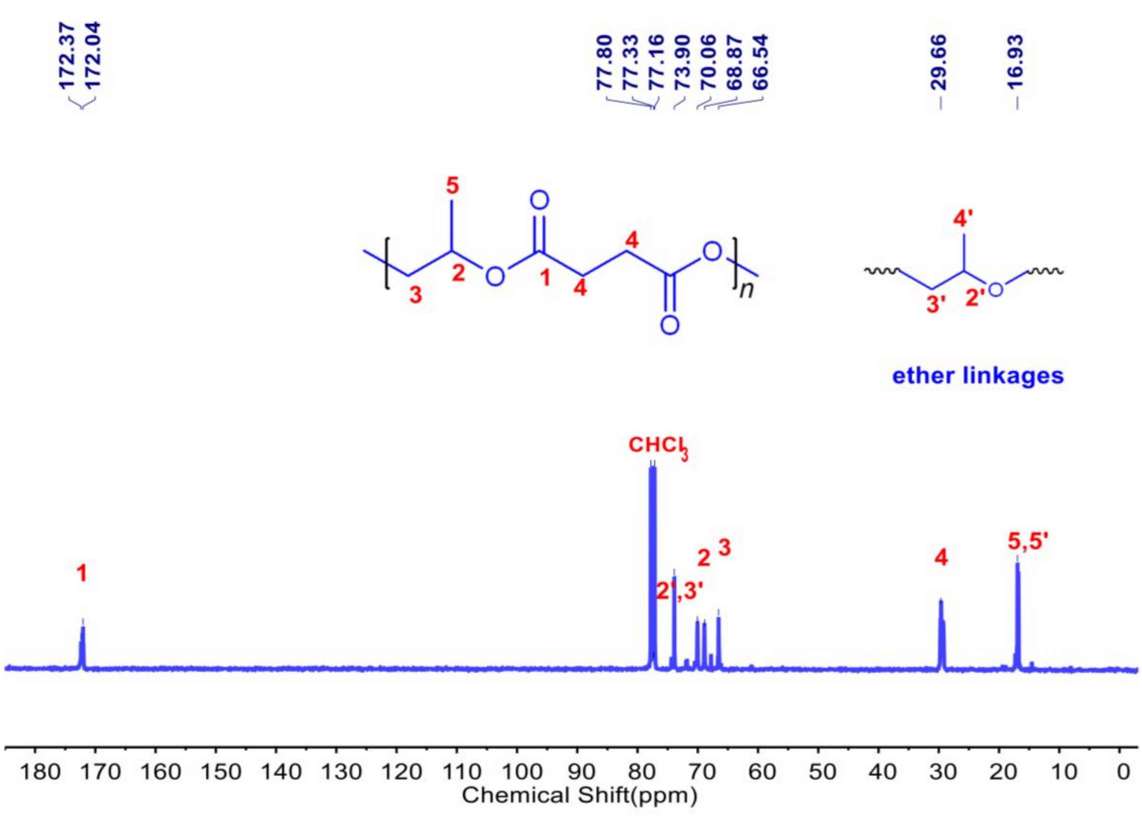

Supplementary Figure 22. ^{13}C NMR spectra of polyester **P1-4.0TEA** (100 MHz, $CDCl_3$).

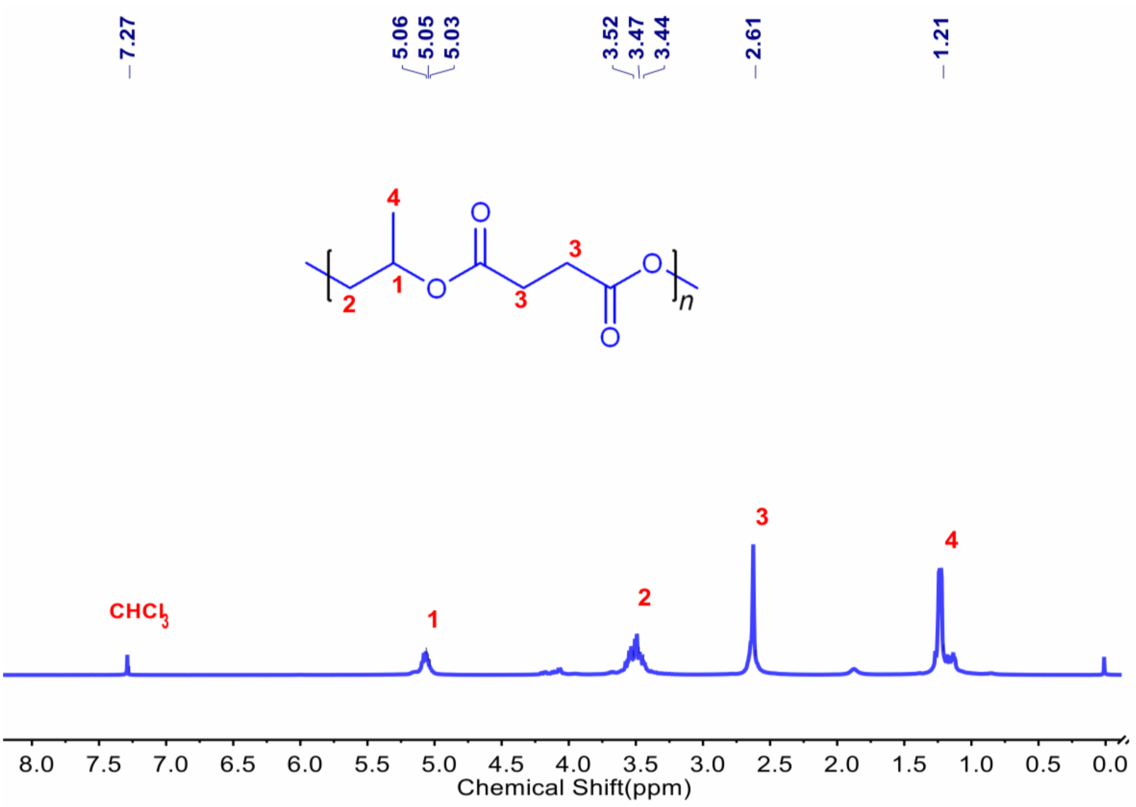

Supplementary Figure 23. ^{1}H NMR spectra of polyester **P1-5.0TEA** (400 MHz, $CDCl_3$).

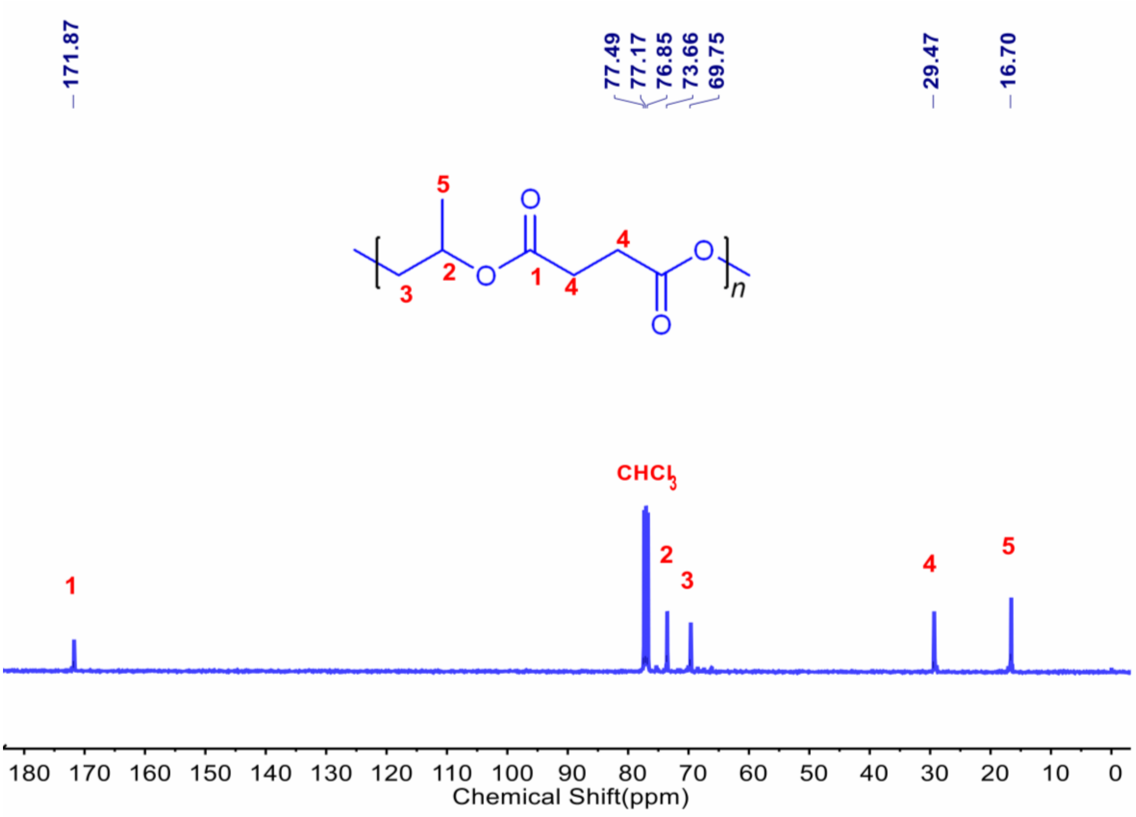

Supplementary Figure 24. ^{13}C NMR spectra of polyester **P1-5.0TEA** (100 MHz, $CDCl_3$).

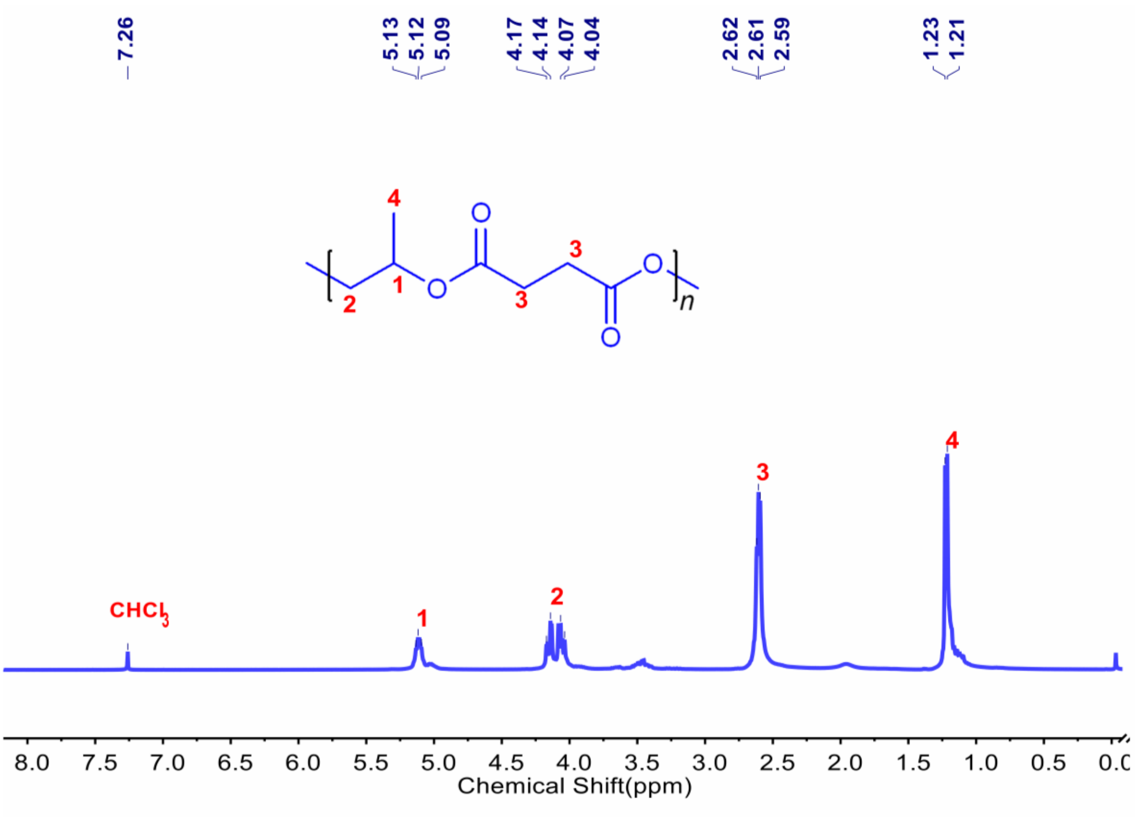

Supplementary Figure 25. ^{1}H NMR spectra of polyester **P1-2.0DBU** (400 MHz, $CDCl_3$).

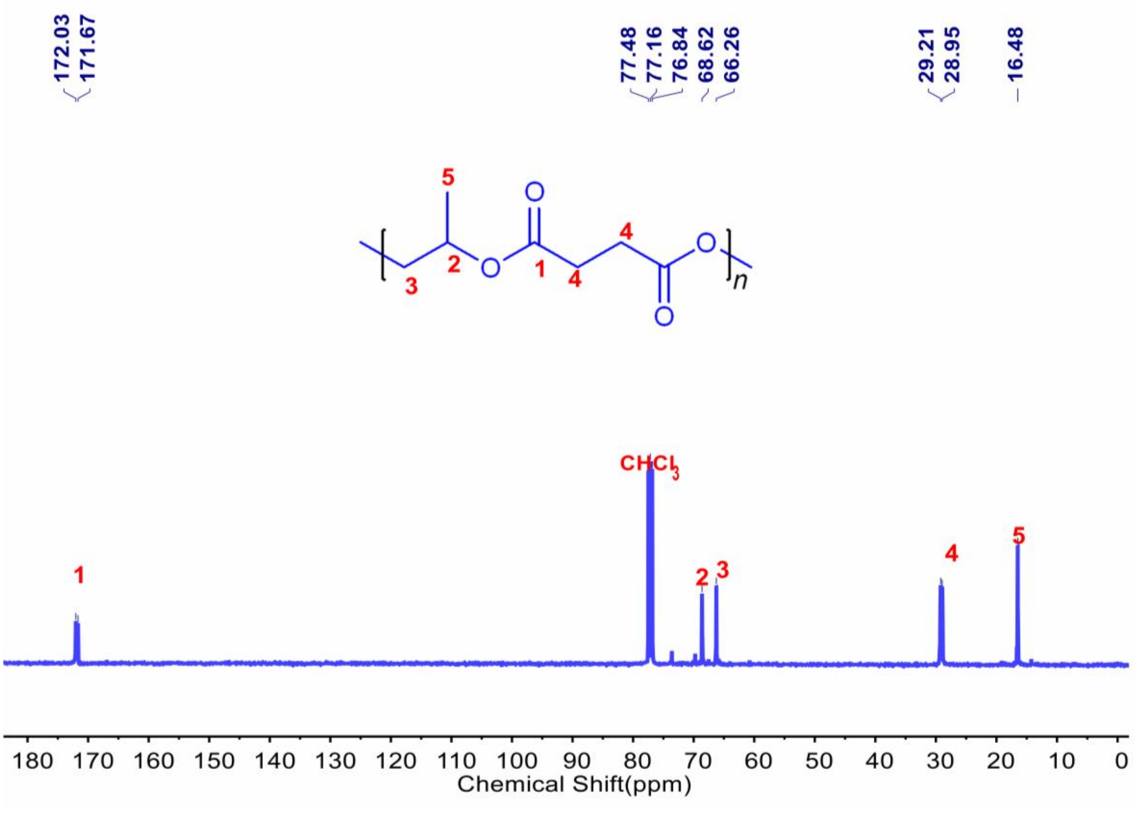

Supplementary Figure 26. ^{13}C NMR spectra of polyester **P1-2.0DBU** (100 MHz, $CDCl_3$).

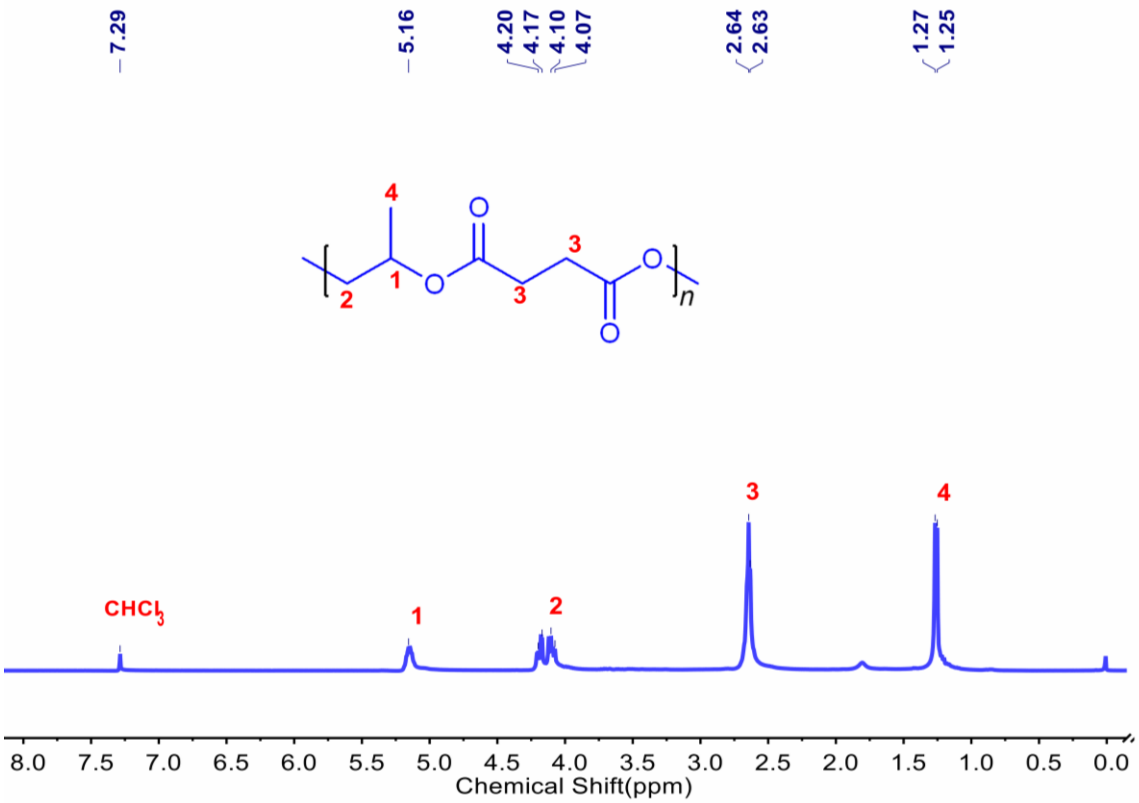

Supplementary Figure 27. ^{1}H NMR spectra of polyester **P1-2.0mTBD** (400 MHz, $CDCl_3$).

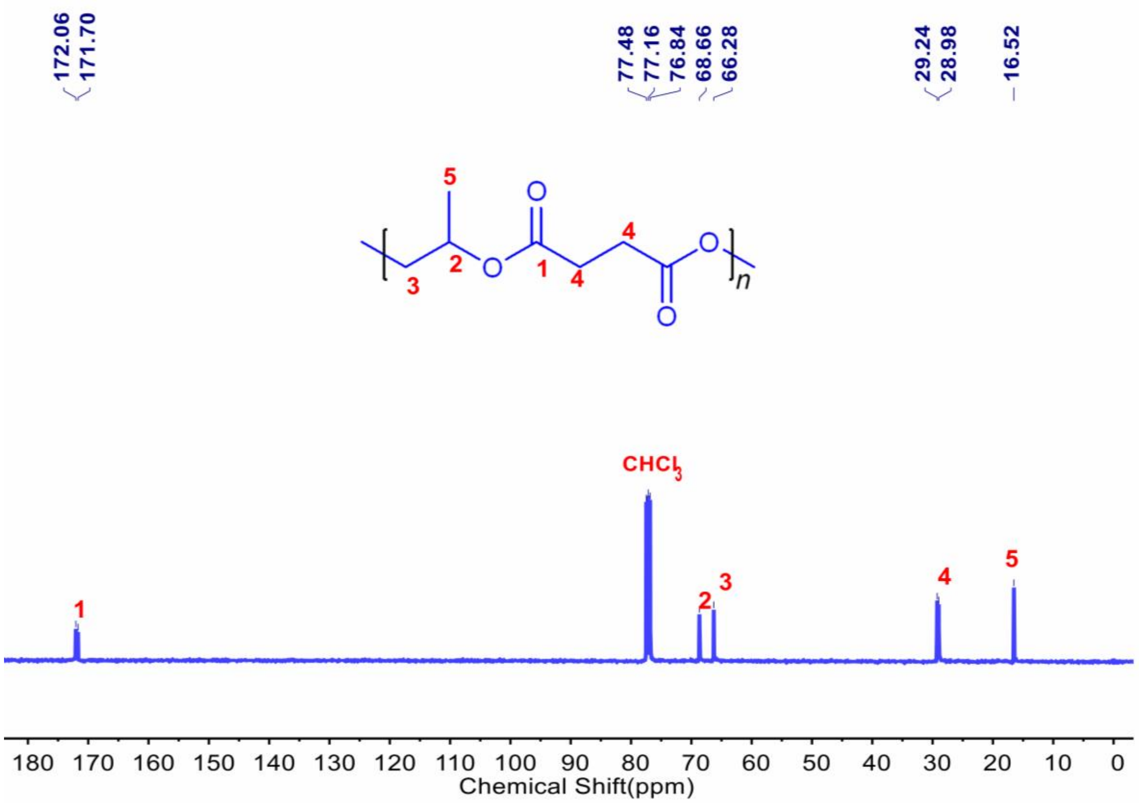

Supplementary Figure 28. ^{13}C NMR spectra of polyester **P1-2.0mTBD** (100 MHz, $CDCl_3$).

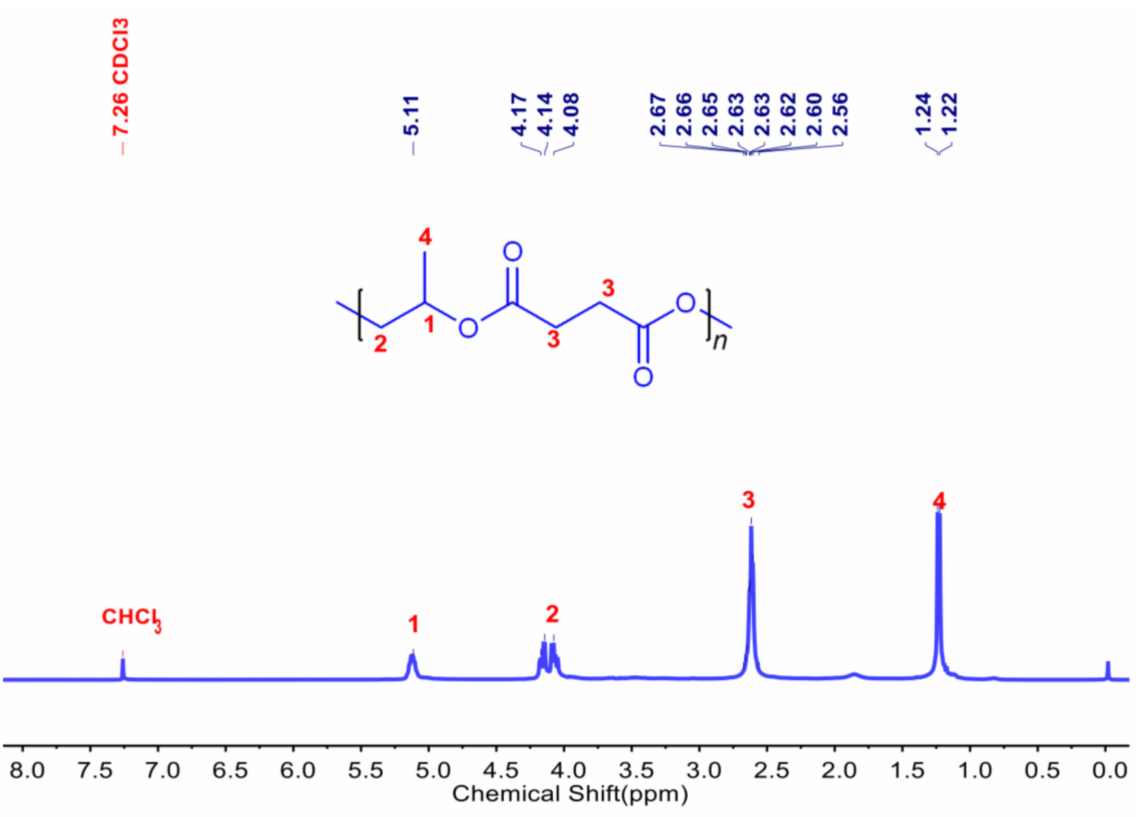

Supplementary Figure 29. ^{1}H NMR spectra of polyester **P1-2.0TBD** (400 MHz, $CDCl_3$).

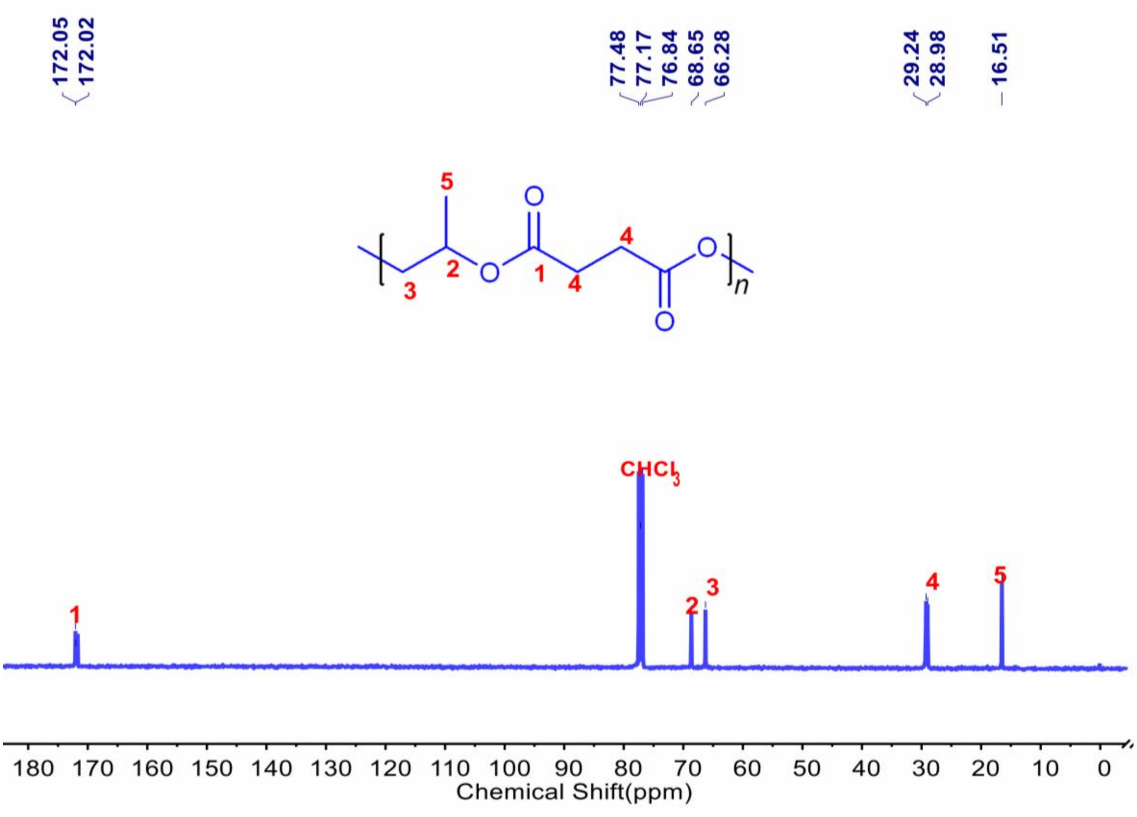

Supplementary Figure 30. ^{13}C NMR spectra of polyester **P1-2.0TBD** (100 MHz, $CDCl_3$).

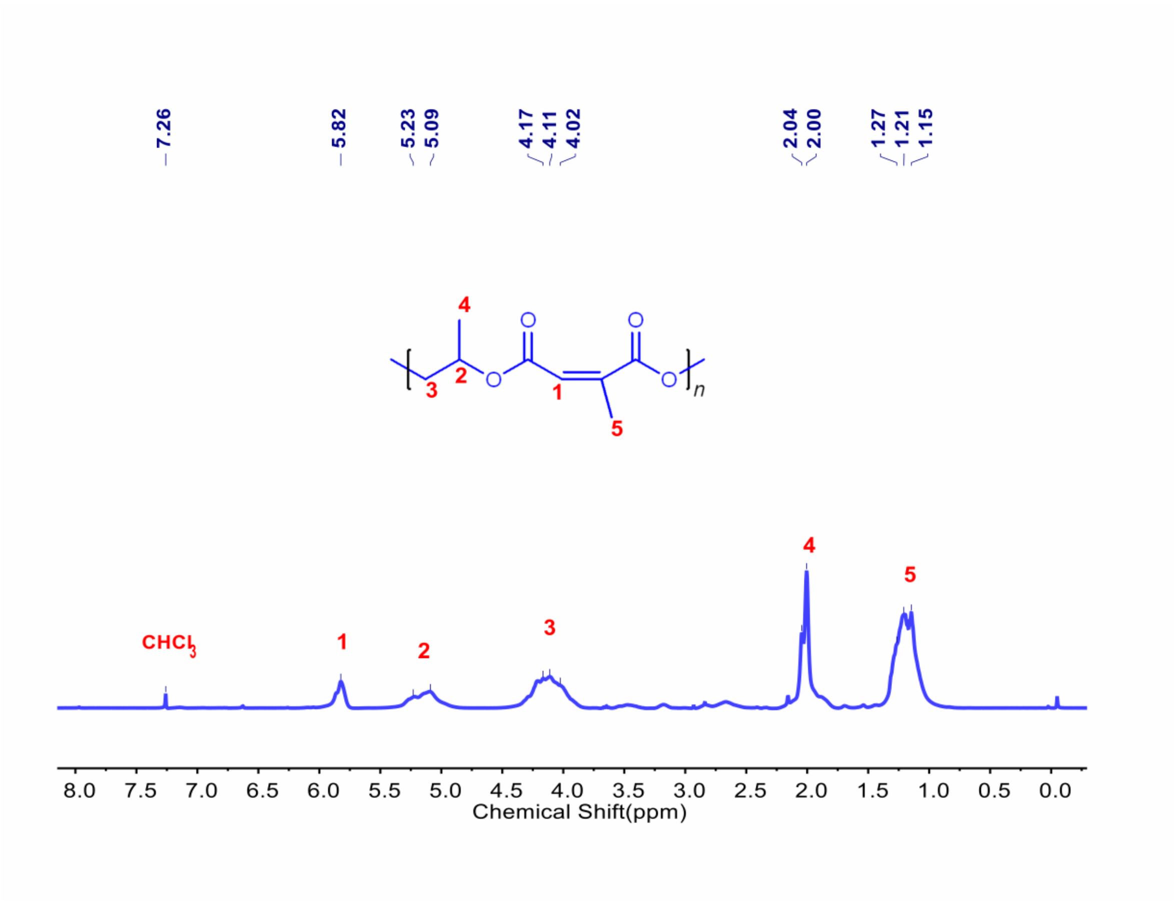

Supplementary Figure 31. ^{1}H NMR spectra of polyester **P2-0.5TEA** (400 MHz, $CDCl_3$).

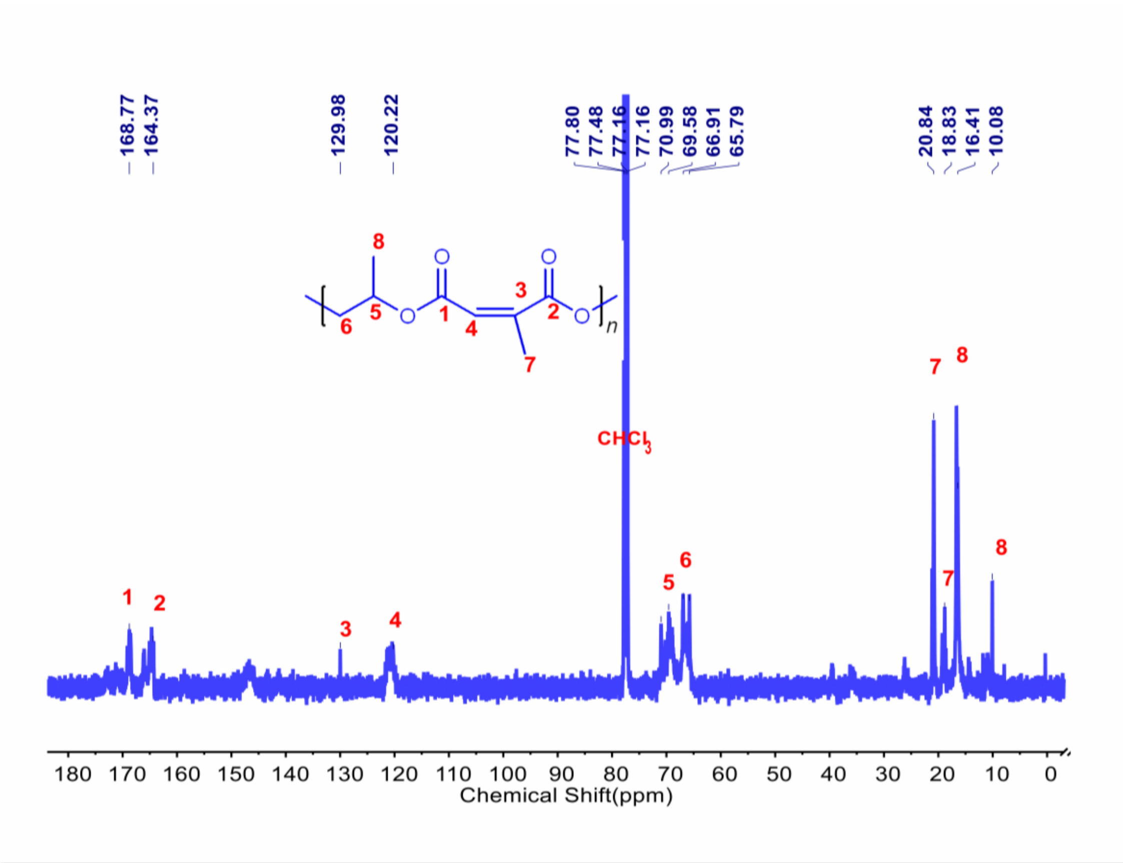

Supplementary Figure 32. ^{13}C NMR spectra of polyester **P2-0.5TEA** (100 MHz, $CDCl_3$).

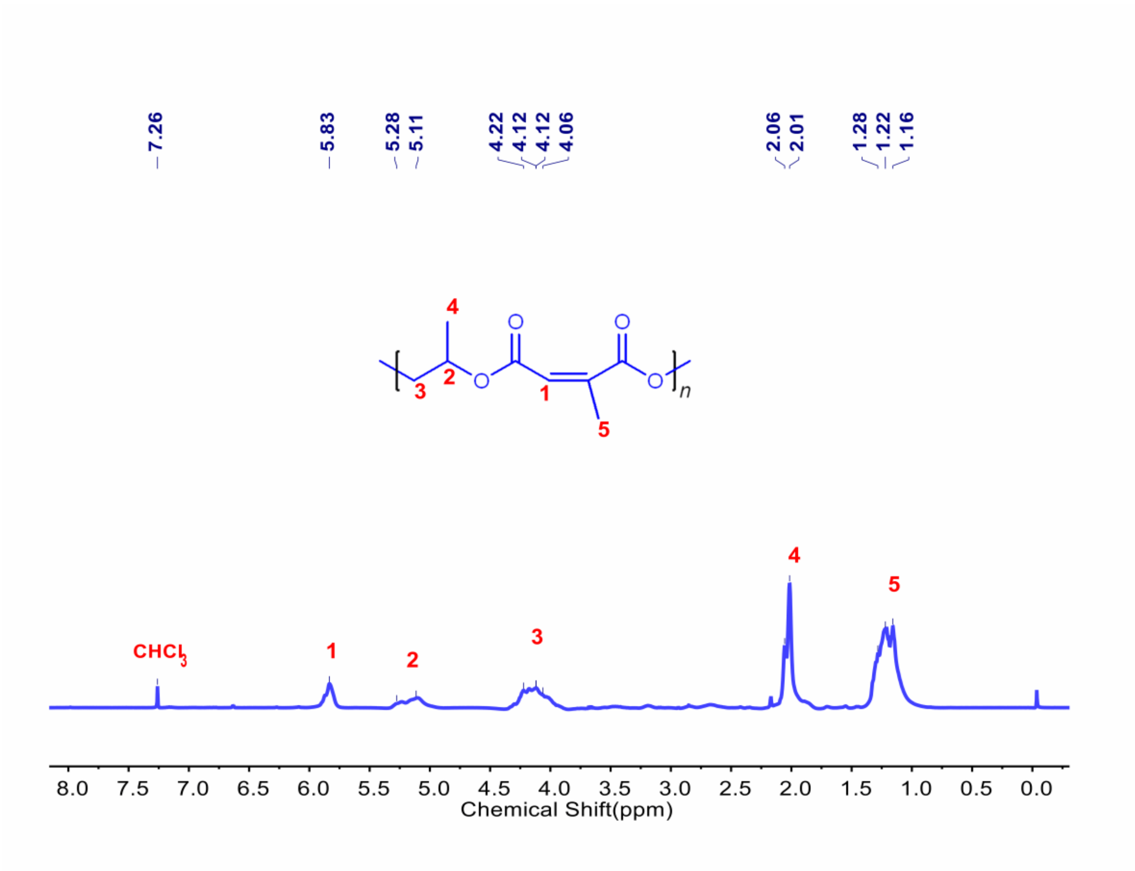

Supplementary Figure 33. ^{1}H NMR spectra of polyester **P2-1.0TEA** (400 MHz, $CDCl_3$).

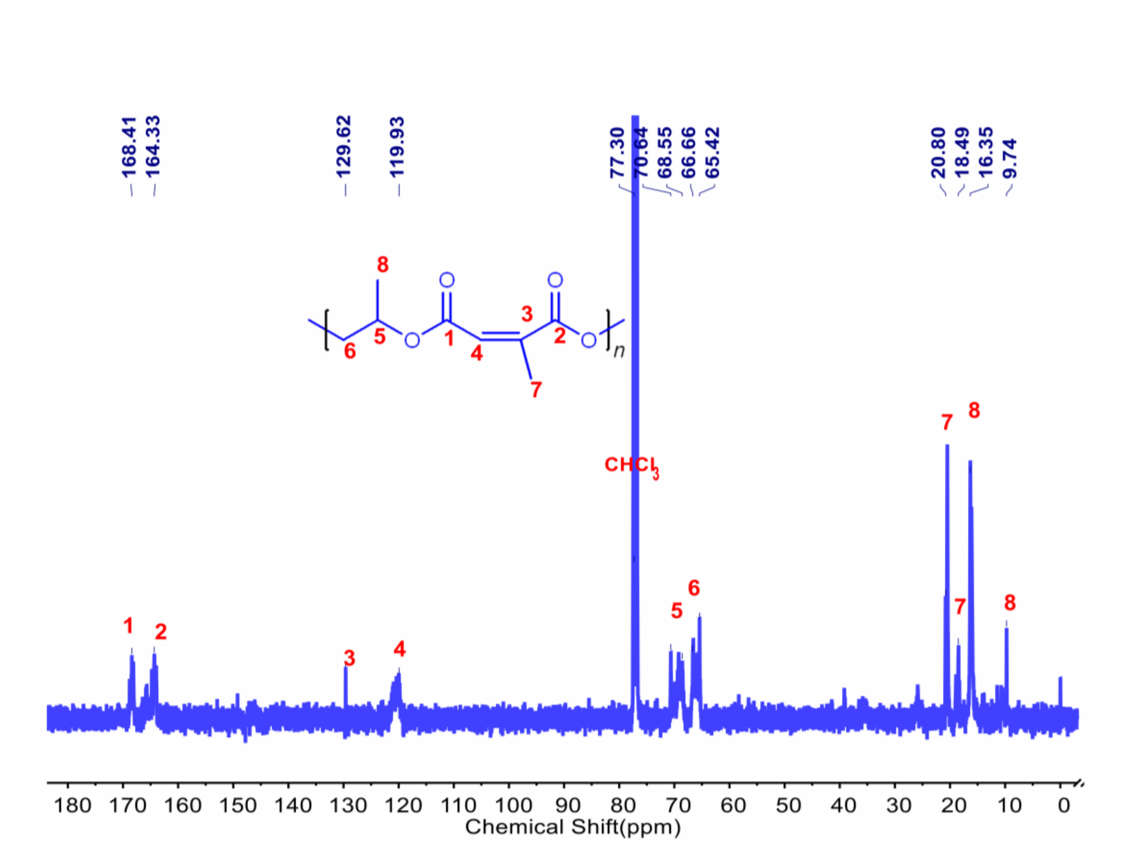

Supplementary Figure 34. ^{13}C NMR spectra of polyester **P2-1.0TEA** (100 MHz, $CDCl_3$).

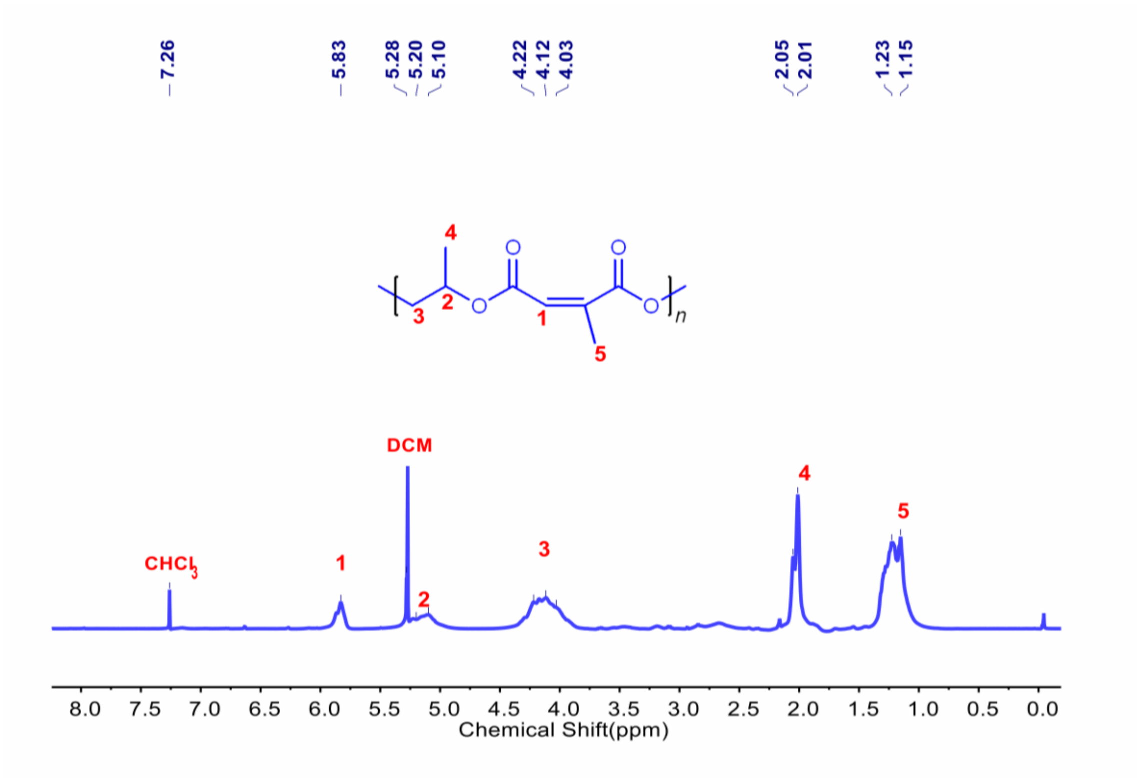

Supplementary Figure 35. ^{1}H NMR spectra of polyester **P2-1.5TEA** (400 MHz, $CDCl_3$).

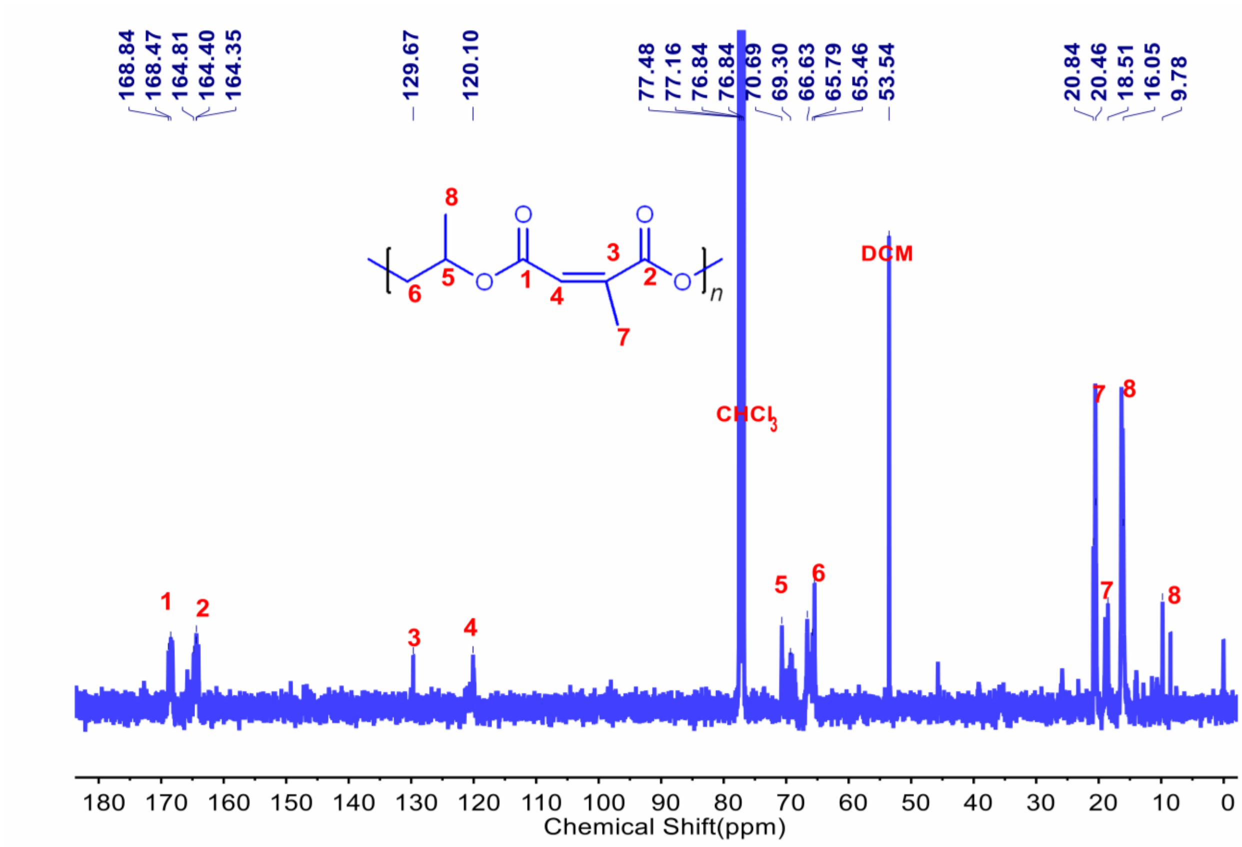

Supplementary Figure 36. ^{13}C NMR spectra of polyester **P2-1.5TEA** (100 MHz, $CDCl_3$).

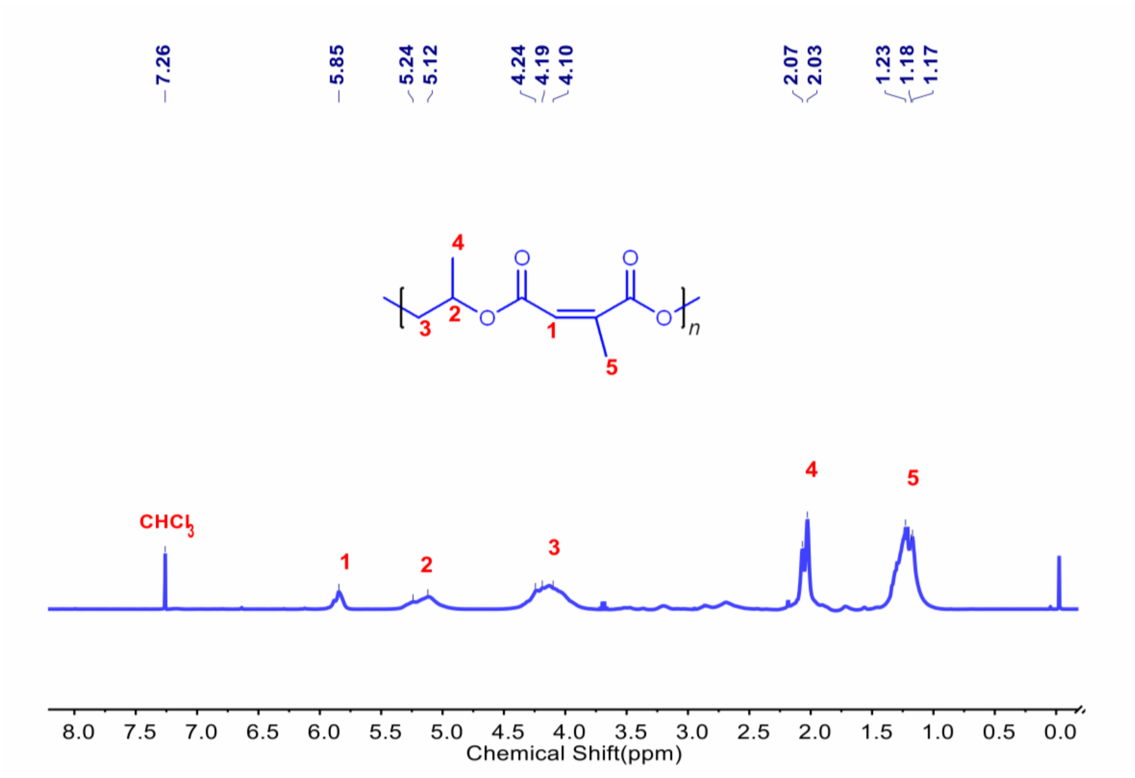

Supplementary Figure 37. ^{1}H NMR spectra of polyester **P2-2.0TEA** (400 MHz, $CDCl_3$).

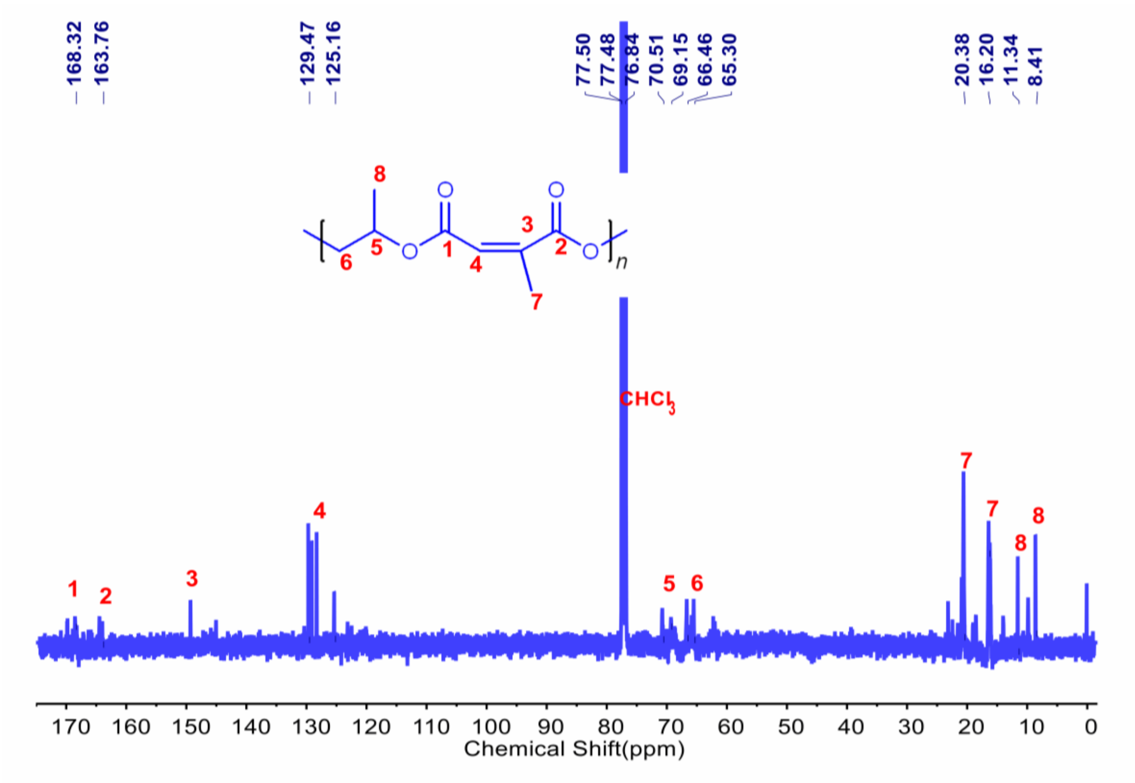

Supplementary Figure 38. ^{13}C NMR spectra of polyester **P2-2.0TEA** (100 MHz, $CDCl_3$).

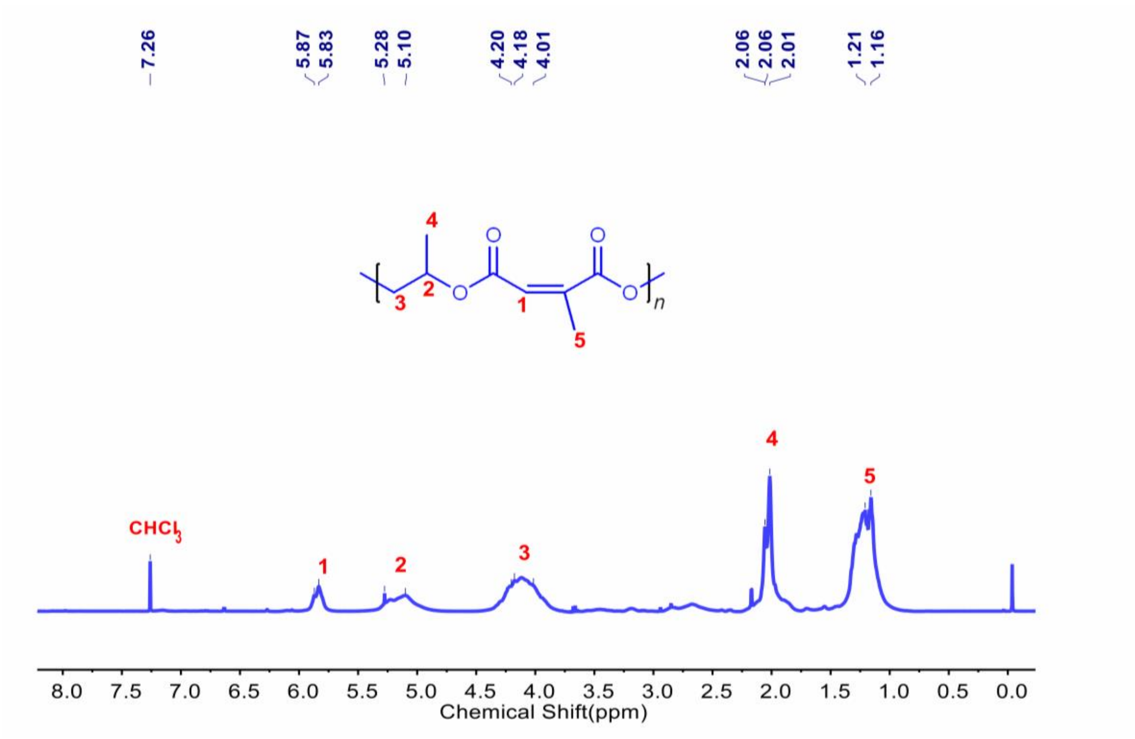

Supplementary Figure 39. ^{1}H NMR spectra of polyester **P2-3.0TEA** (400 MHz, $CDCl_3$).

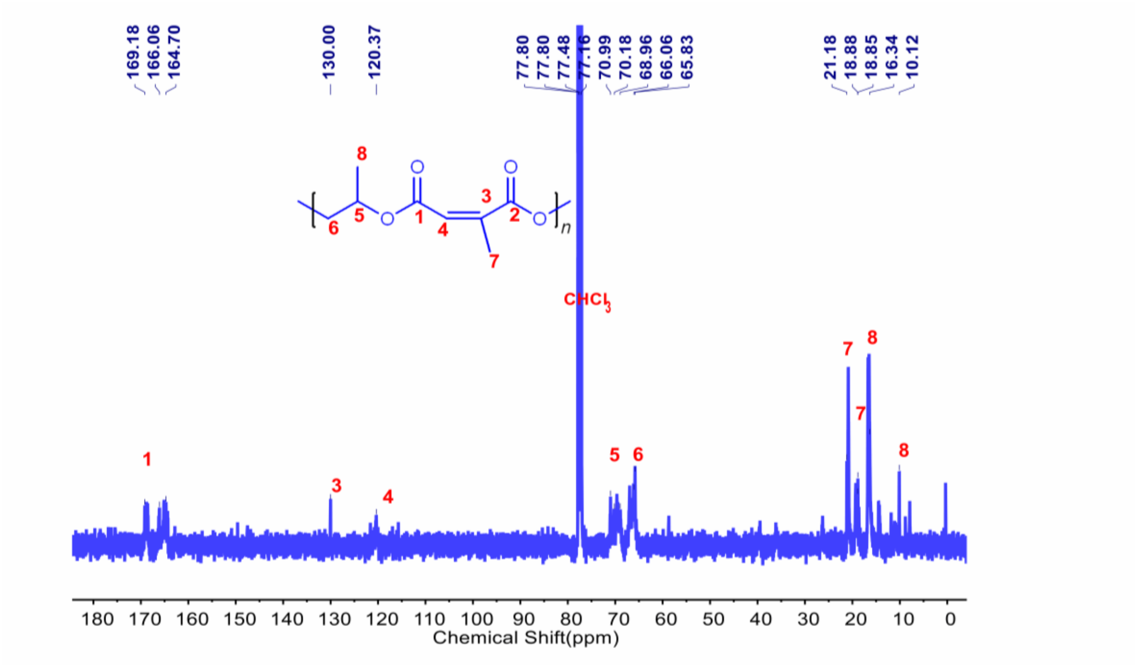

Supplementary Figure 40. ^{13}C NMR spectra of polyester **P2-3.0TEA** (100 MHz, $CDCl_3$).

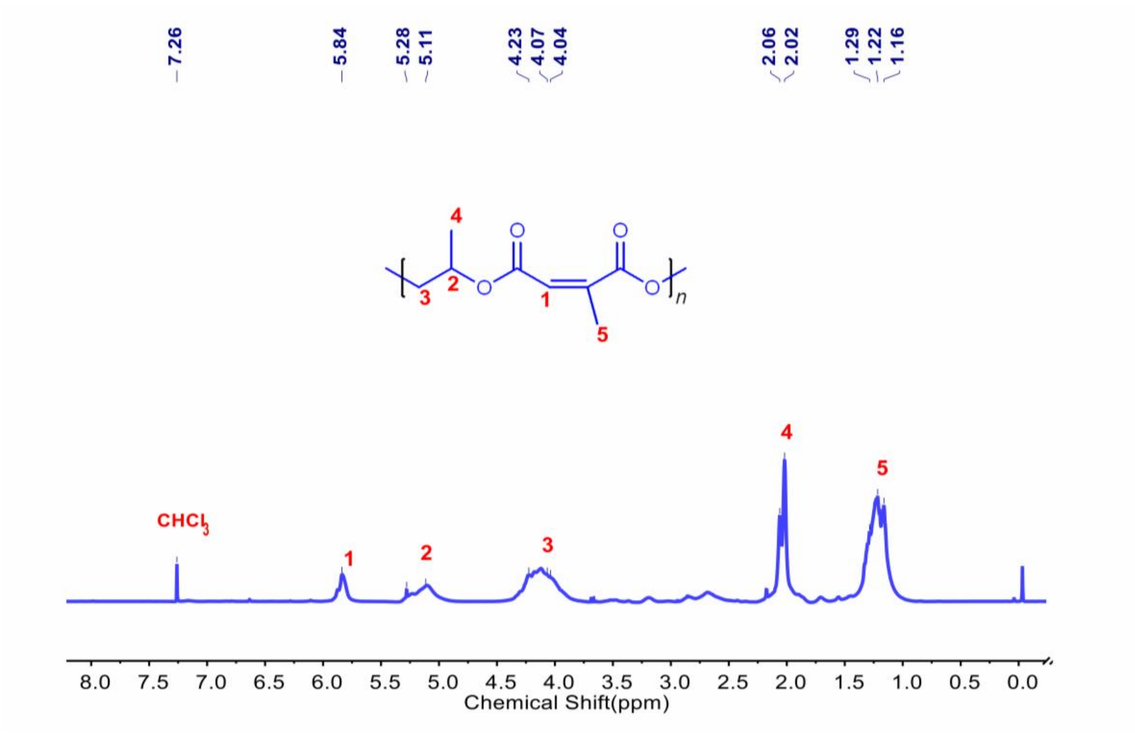

Supplementary Figure 41. ^{1}H NMR spectra of polyester **P2-4.0TEA** (400 MHz, $CDCl_3$).

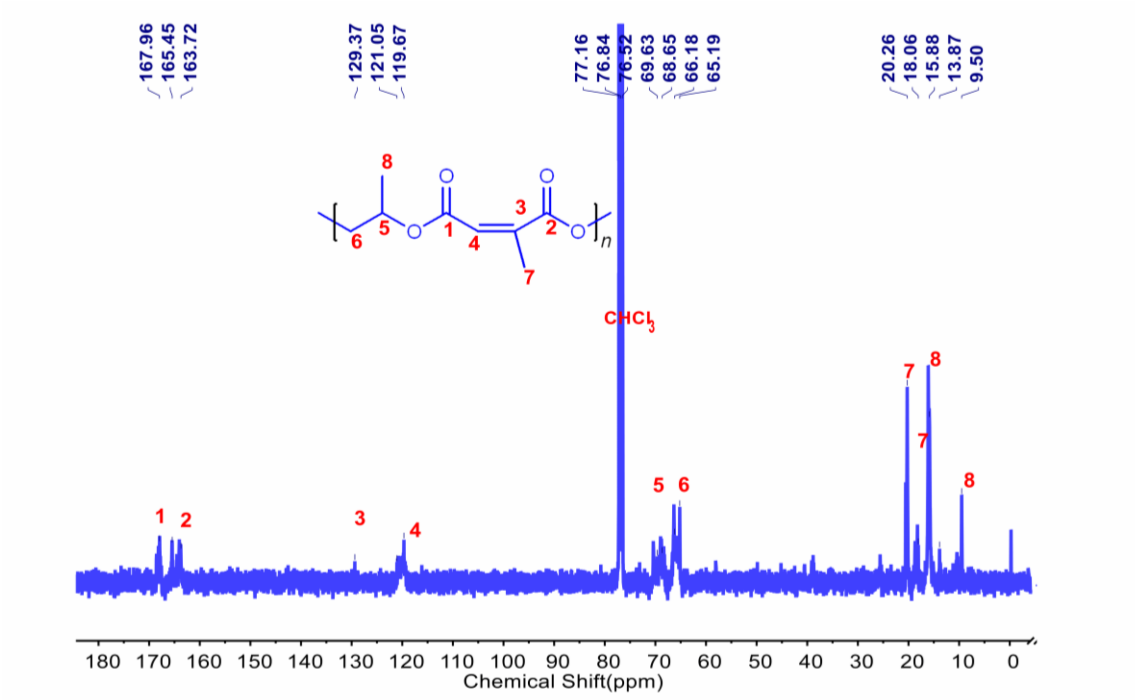

Supplementary Figure 42. ^{13}C NMR spectra of polyester **P2-4.0TEA** (100 MHz, $CDCl_3$).

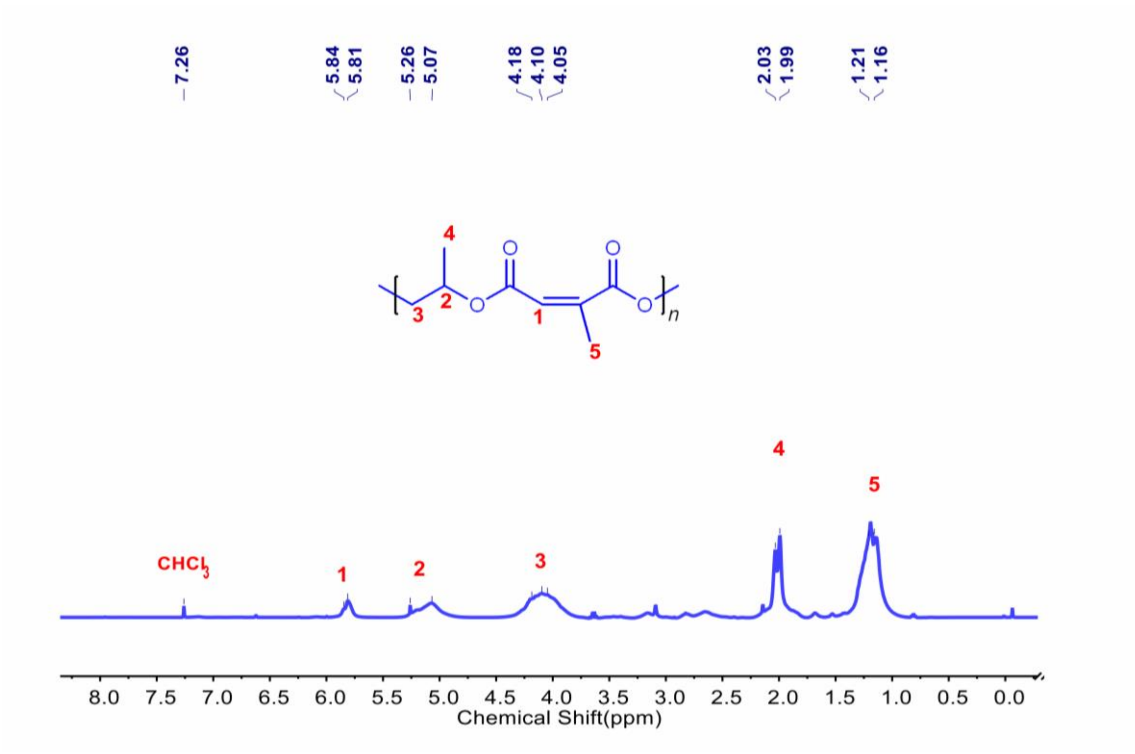

Supplementary Figure 43. ^{1}H NMR spectra of polyester **P2-5.0TEA** (400 MHz, $CDCl_3$).

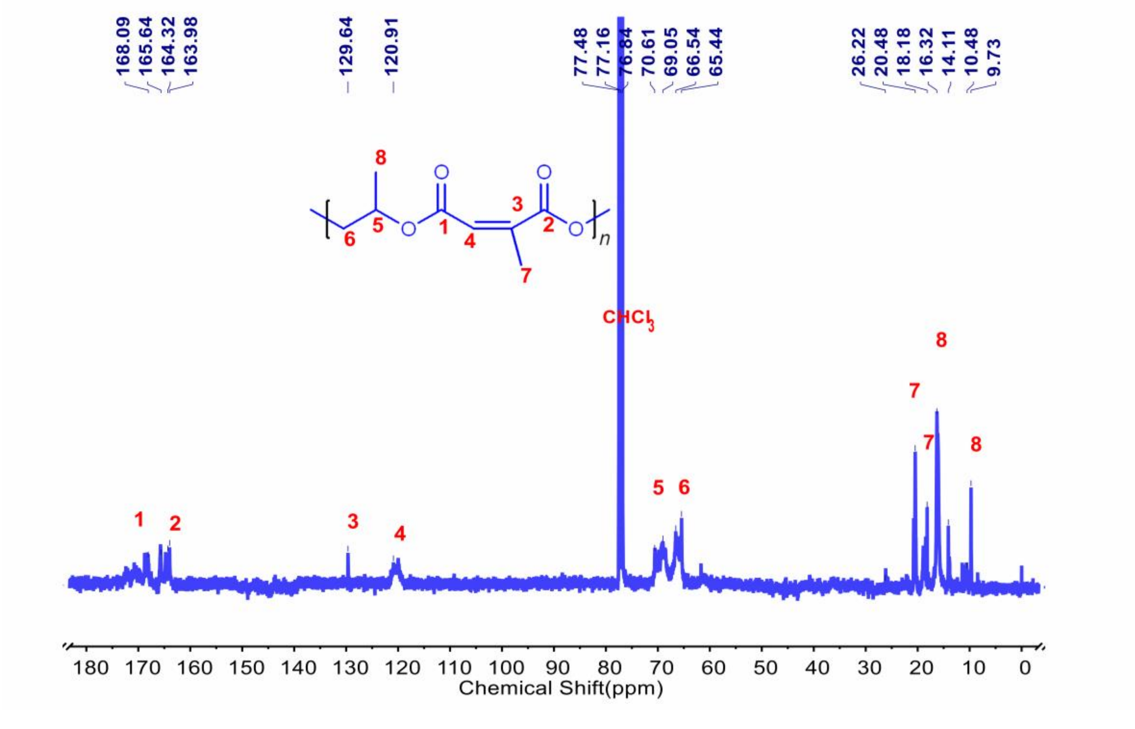

Supplementary Figure 44. ^{13}C NMR spectra of polyester **P2-5.0TEA** (100 MHz, $CDCl_3$).

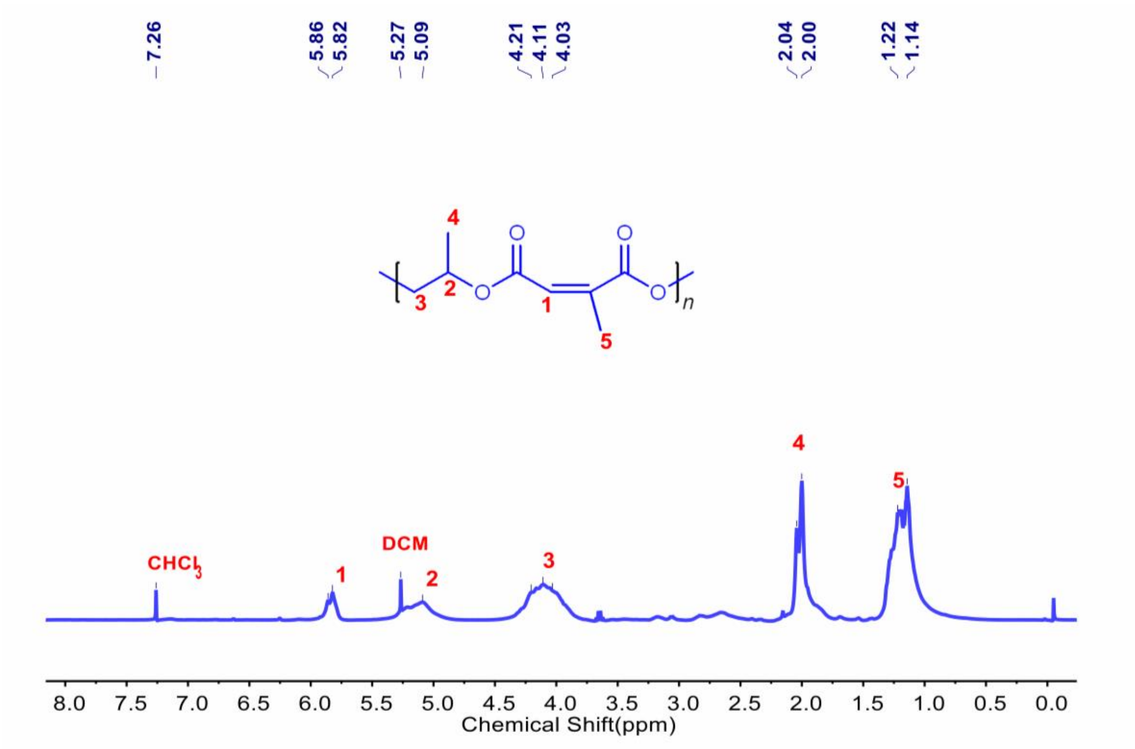

Supplementary Figure 45. ^{1}H NMR spectra of polyester **P2-2.0DBU** (400 MHz, $CDCl_3$).

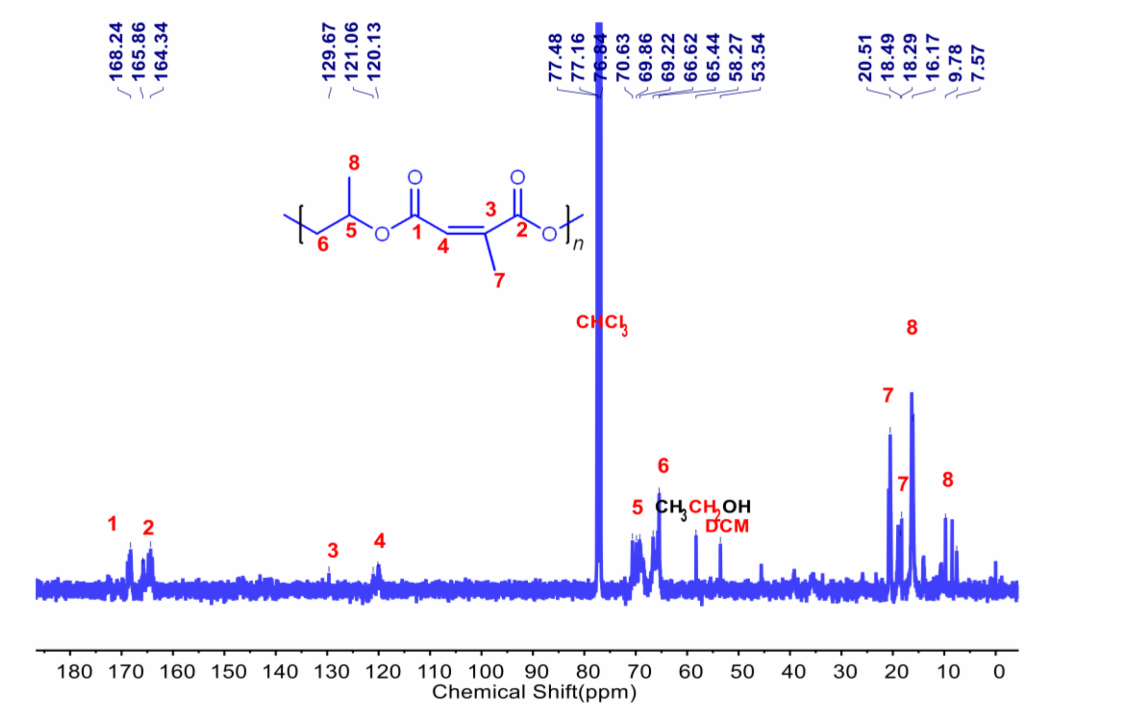

Supplementary Figure 46. ^{13}C NMR spectra of polyester **P2-2.0DBU** (100 MHz, $CDCl_3$).

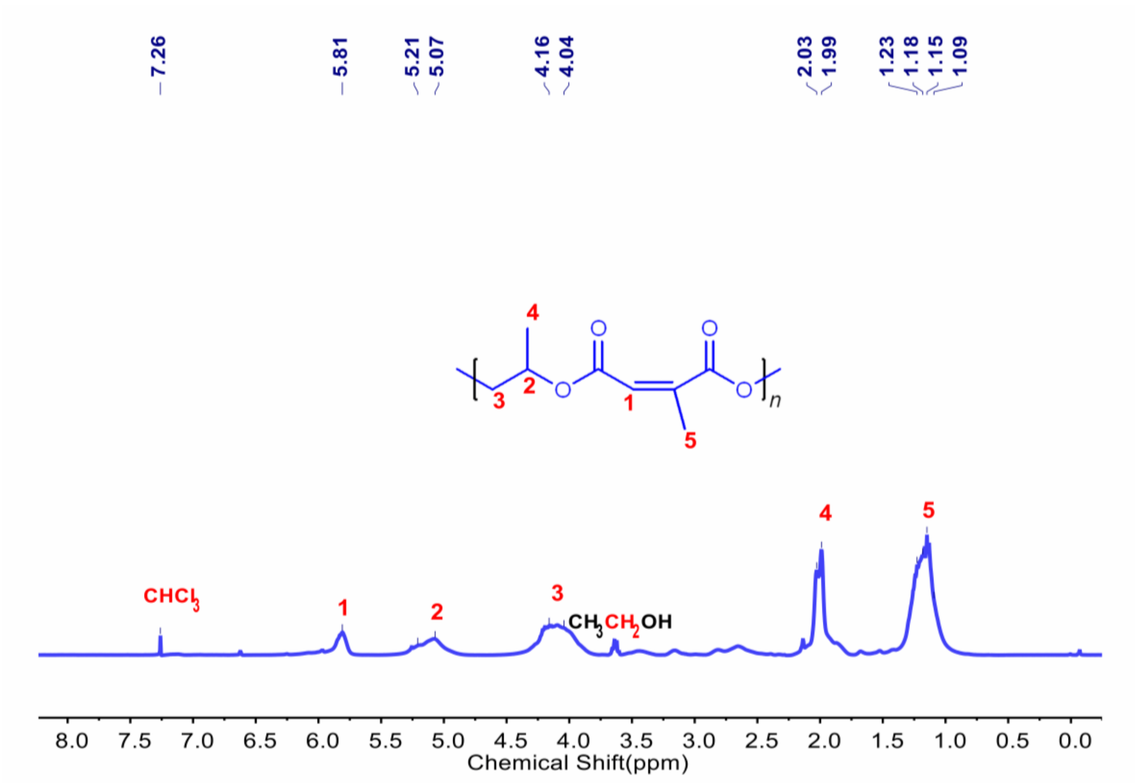

Supplementary Figure 47. ¹H NMR spectra of polyester **P2-2.0mTBD** (400 MHz, $CDCl_3$).

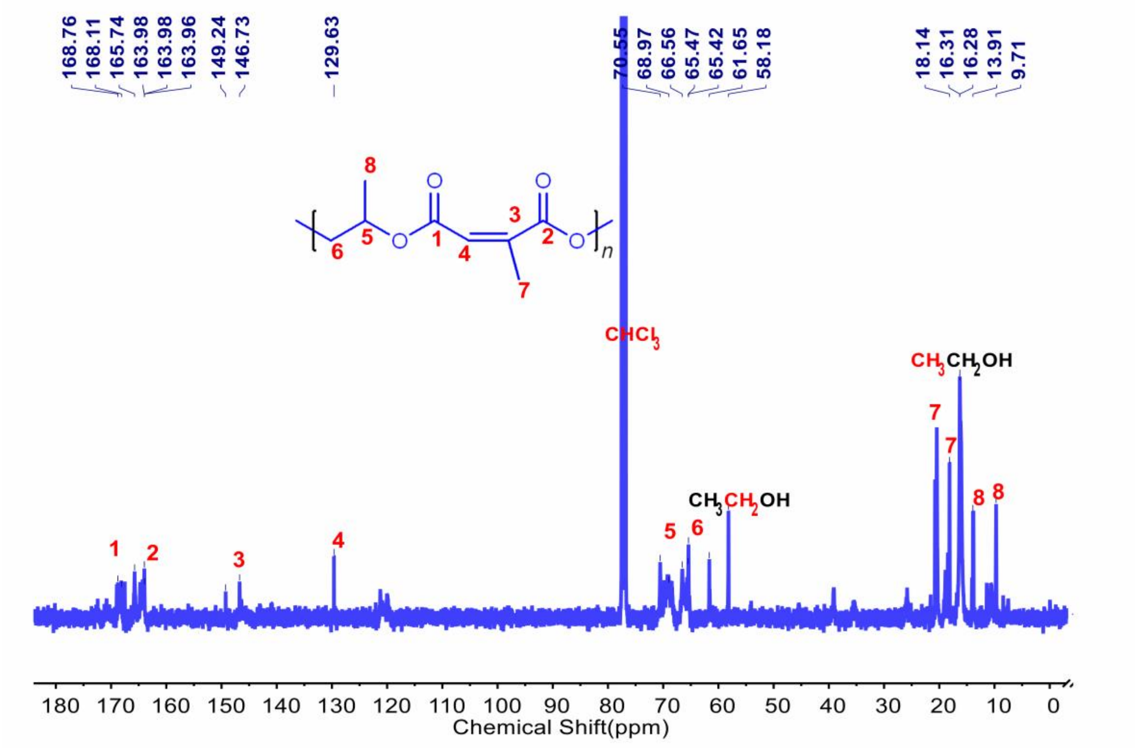

Supplementary Figure 48. ¹³C NMR spectra of polyester **P2-2.0mTBD** (100 MHz, $CDCl_3$).

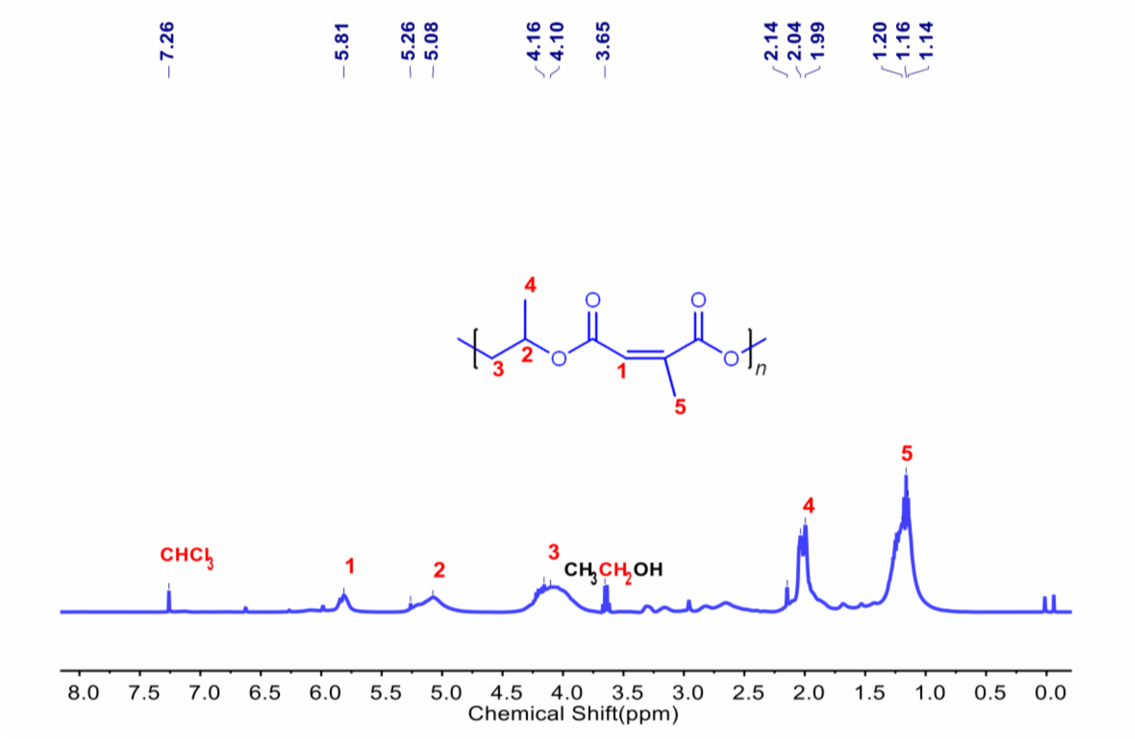

Supplementary Figure 49. ^{1}H NMR spectra of polyester **P2-2.0TBD** (400 MHz, $CDCl_3$).

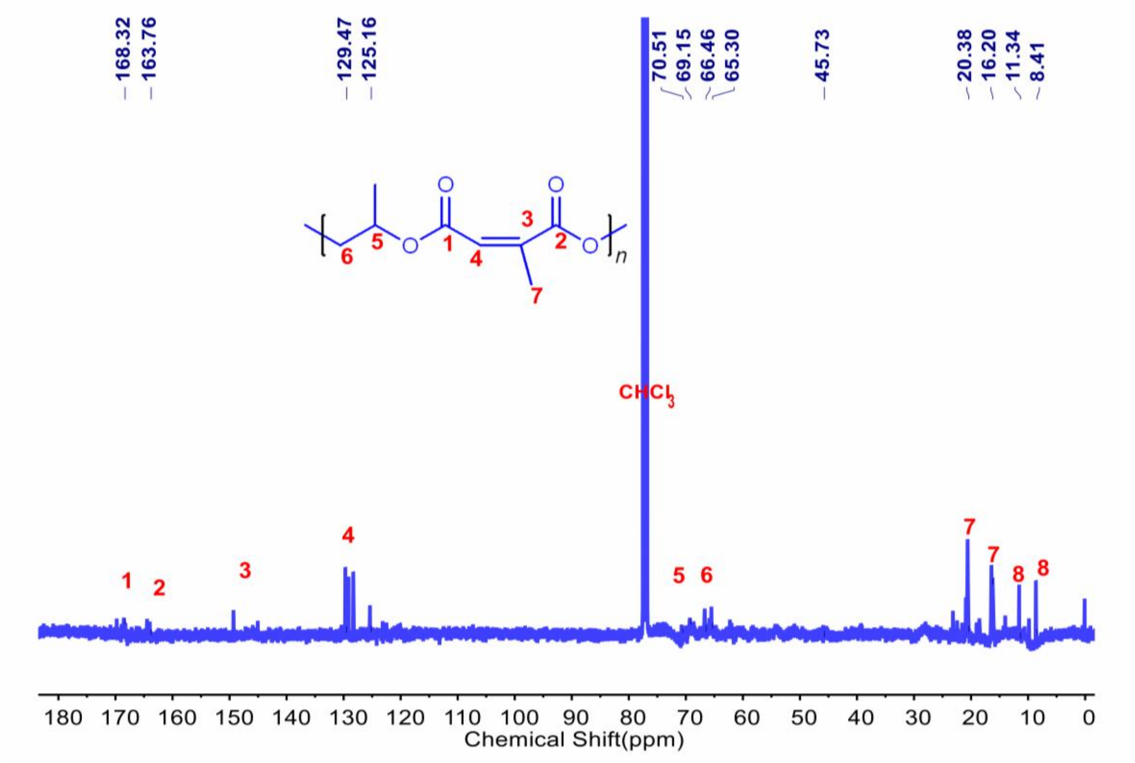

Supplementary Figure 50. ^{13}C NMR spectra of polyester **P2-2.0TBD** (100 MHz, $CDCl_3$).

Gel Permeation Chromatography

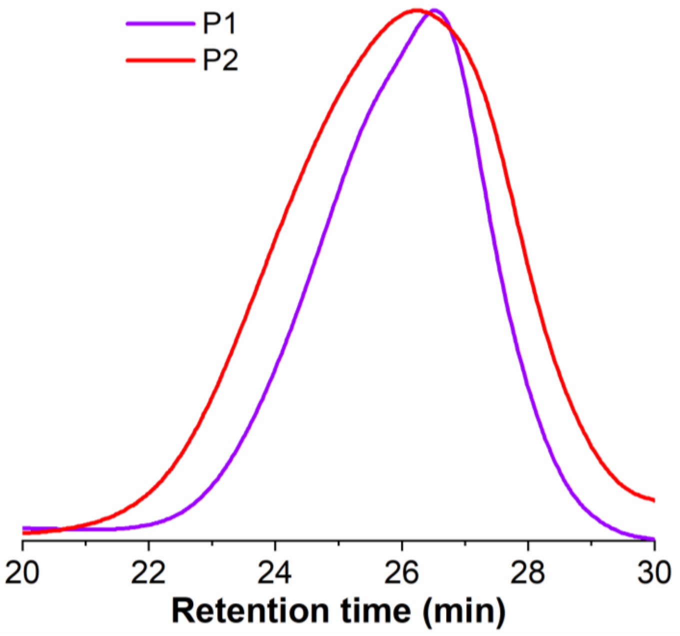

Supplementary Figure 51. GPC curves of **P1** and **P2** were used as polystyrene the standard sample using chromatographic purity THF as an eluent at 40 °C.

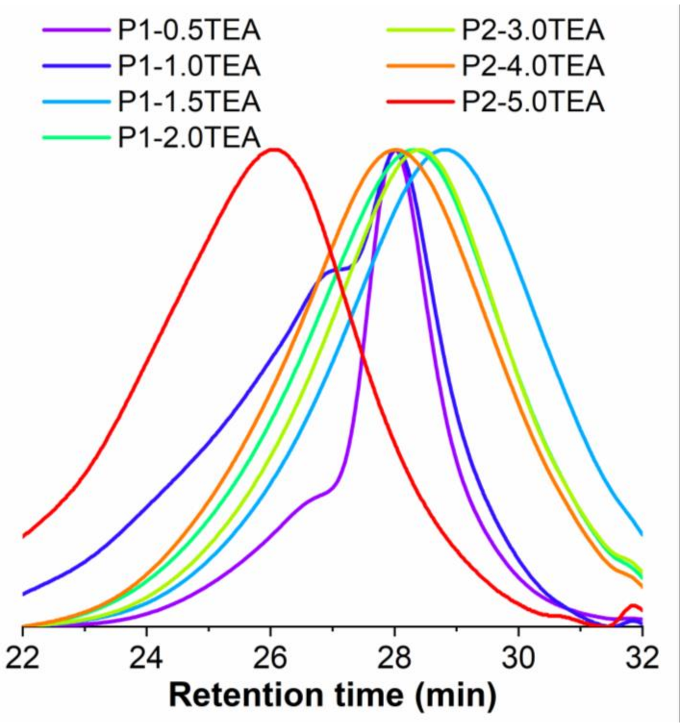

Supplementary Figure 52. GPC curves of **P1-aTEA** (a% is from 0.5% to 5.0%) were used as polystyrene the standard sample using chromatographic purity THF as an eluent at 40 °C.

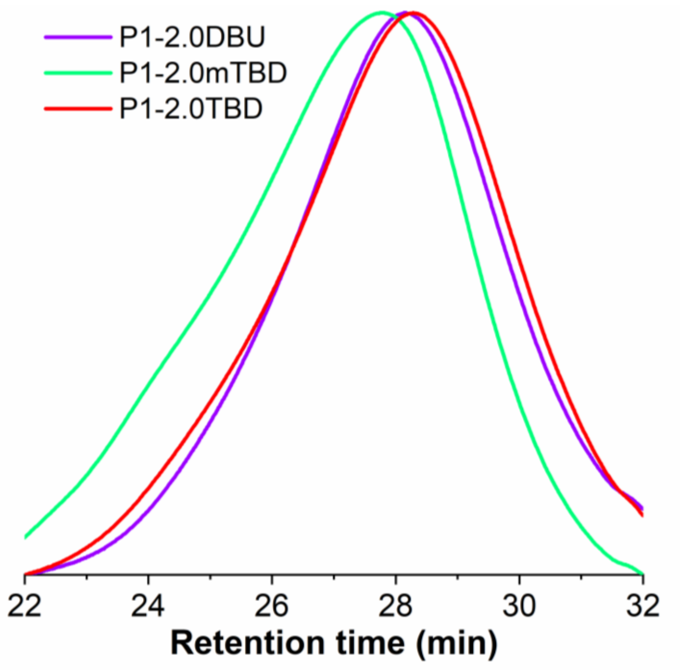

Supplementary Figure 53. GPC curves of **P1-2.0DBU**, **P1-2.0mTBD** and **P1-2.0TBD** were used as polystyrene the standard sample using chromatographic purity THF as an eluent at 40 °C.

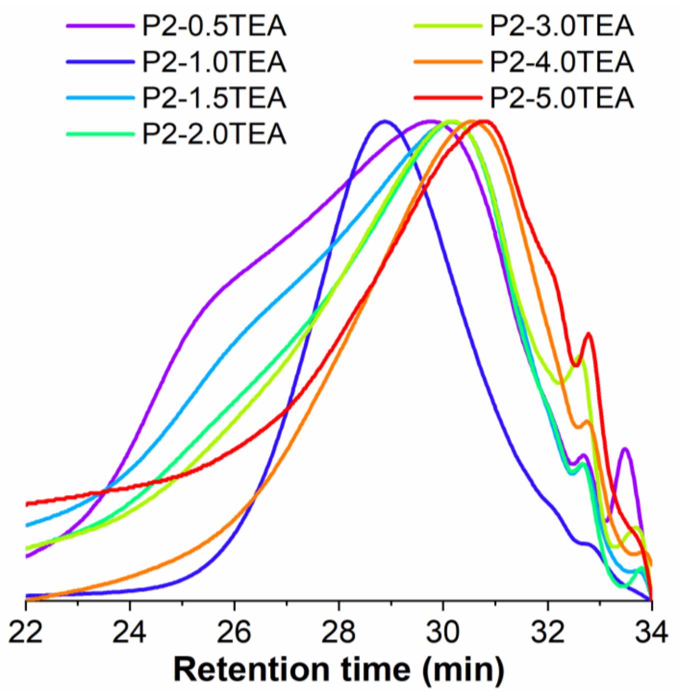

Supplementary Figure 54. GPC curves of **P2-aTEA** (a% is from 0.5% to 5.0%) were used as polystyrene the standard sample using chromatographic purity THF as an eluent at 40 °C.

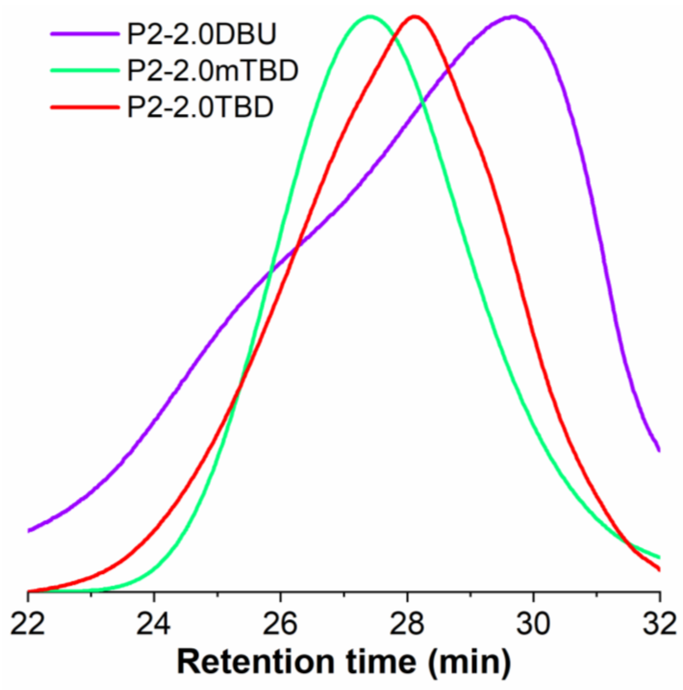

Supplementary Figure 55. GPC curves of **P2-2.0DBU**, **P2-2.0mTBD** and **P2-2.0TBD** were used as polystyrene the standard sample using chromatographic purity THF as an eluent at 40 °C.

Matrix Assisted Laser Desorption Ionization-Time of Flight (MALDI-TOF) Mass Spectra

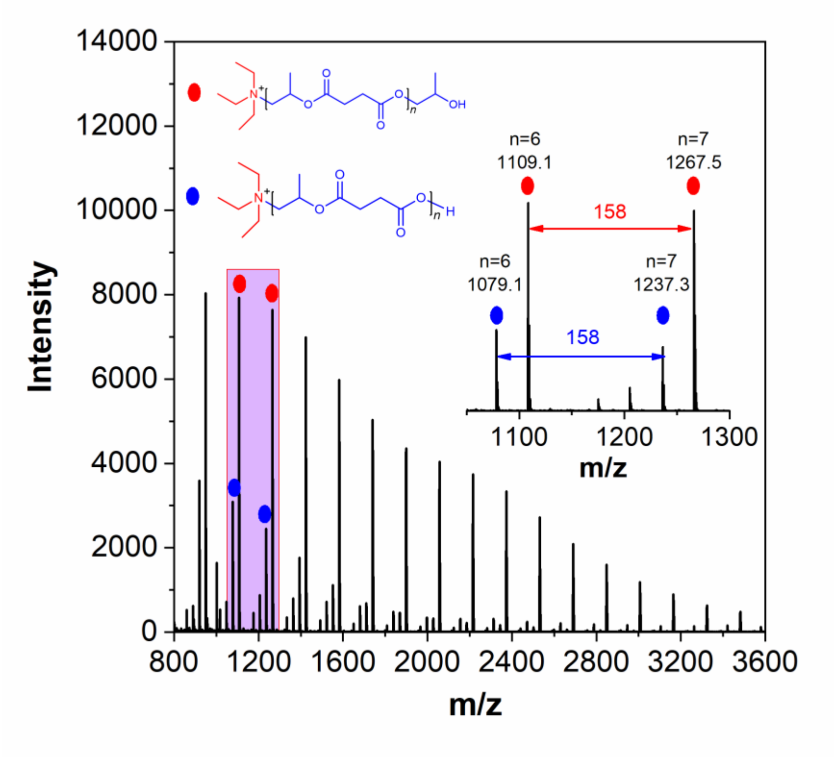

Supplementary Figure 56. MALDI-TOF mass spectrum of **P1-2.0TEA**.

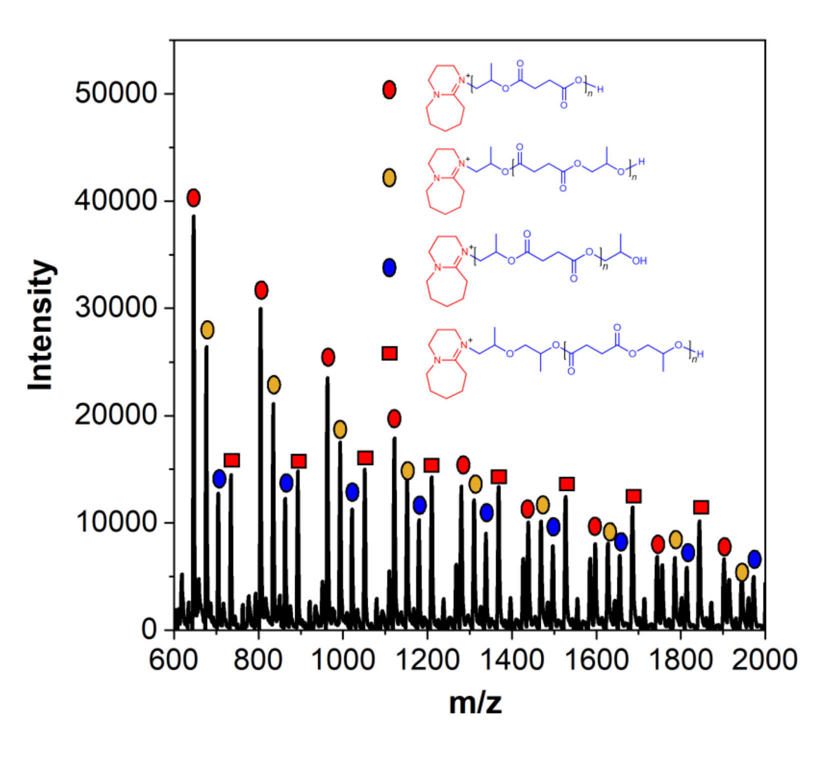

Supplementary Figure 57. MALDI-TOF mass spectrum of **P1-2.0DBU**.

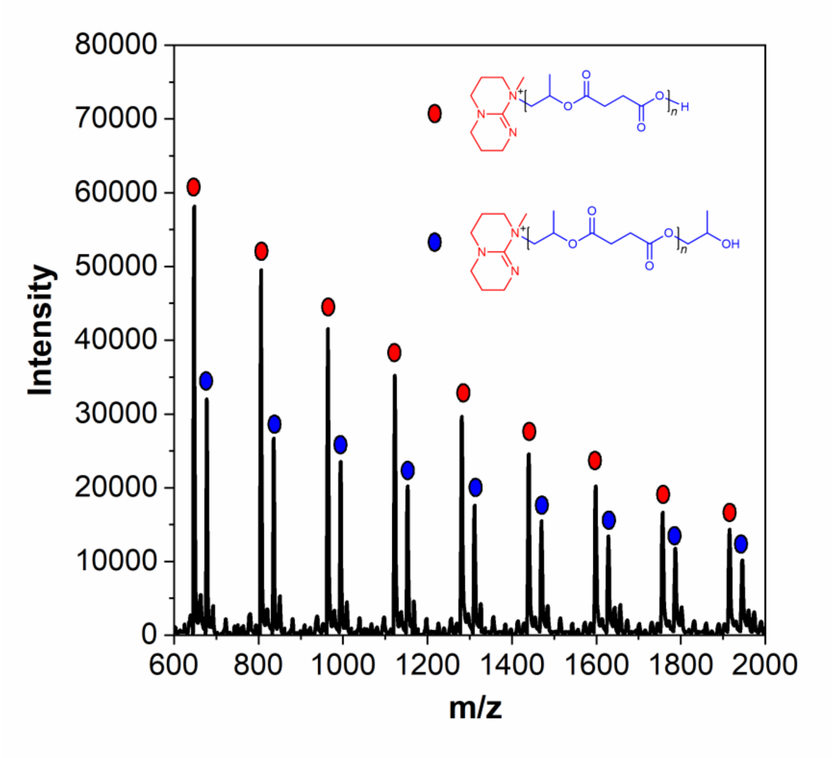

Supplementary Figure 58. MALDI-TOF mass spectrum of **P1-2.0mTBD**.

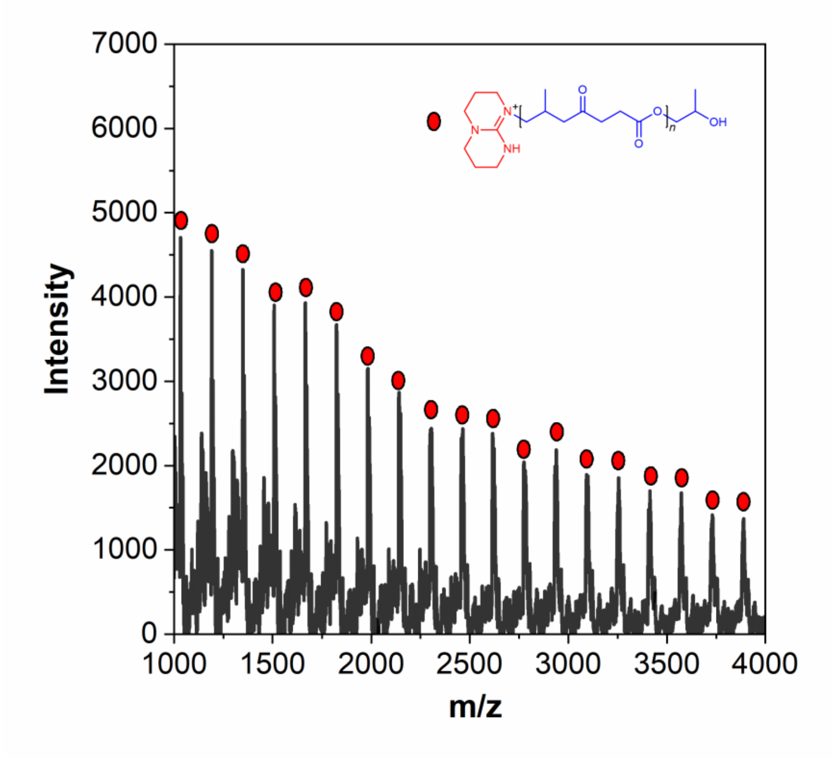

Supplementary Figure59. MALDI-TOF mass spectrum of **P1-2.0TBD**.

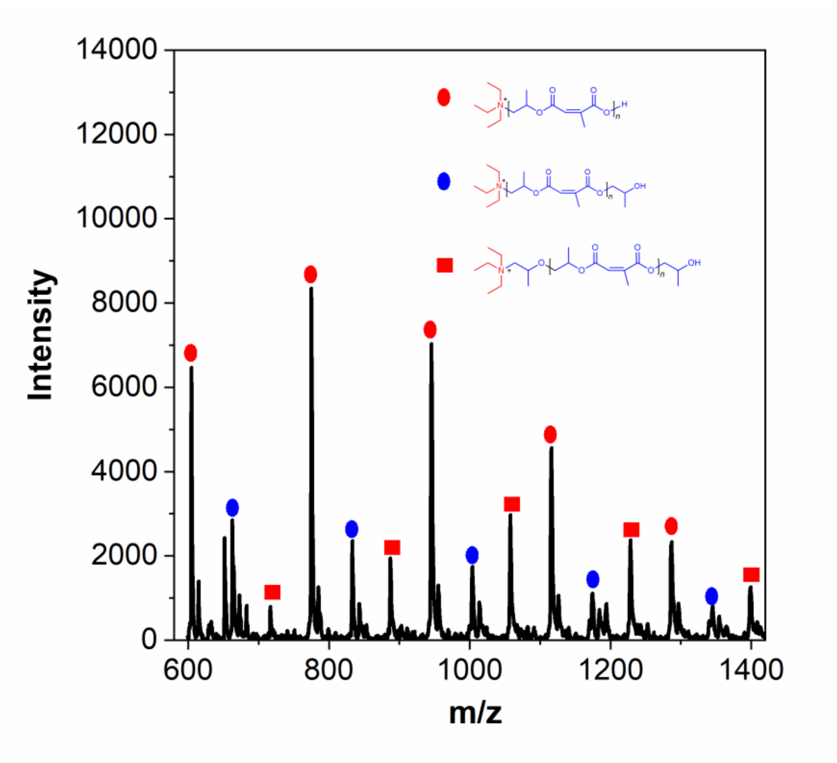

Supplementary Figure 60. MALDI-TOF mass spectrum of **P2-2.0TEA**.

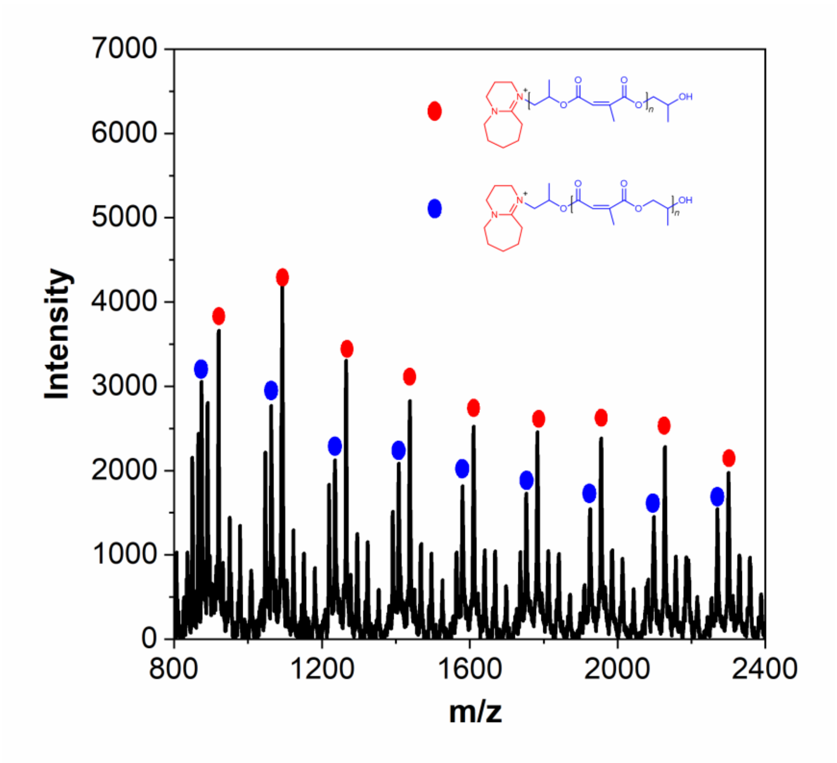

Supplementary Figure 61. MALDI-TOF mass spectrum of **P2-2.0DBU**.

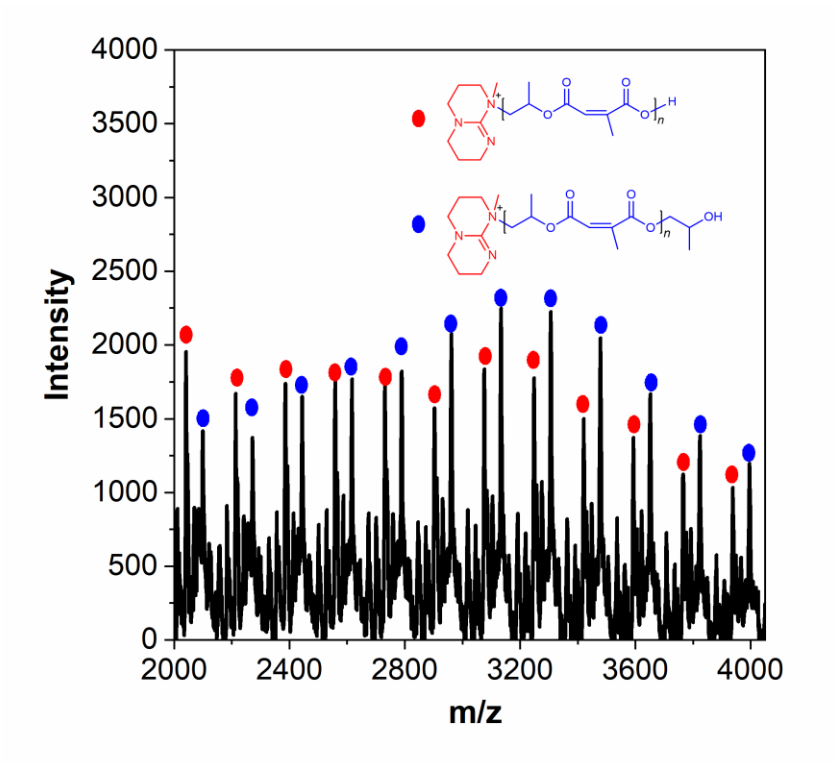

Supplementary Figure 62. MALDI-TOF mass spectrum of **P2-2.0mTBD**.

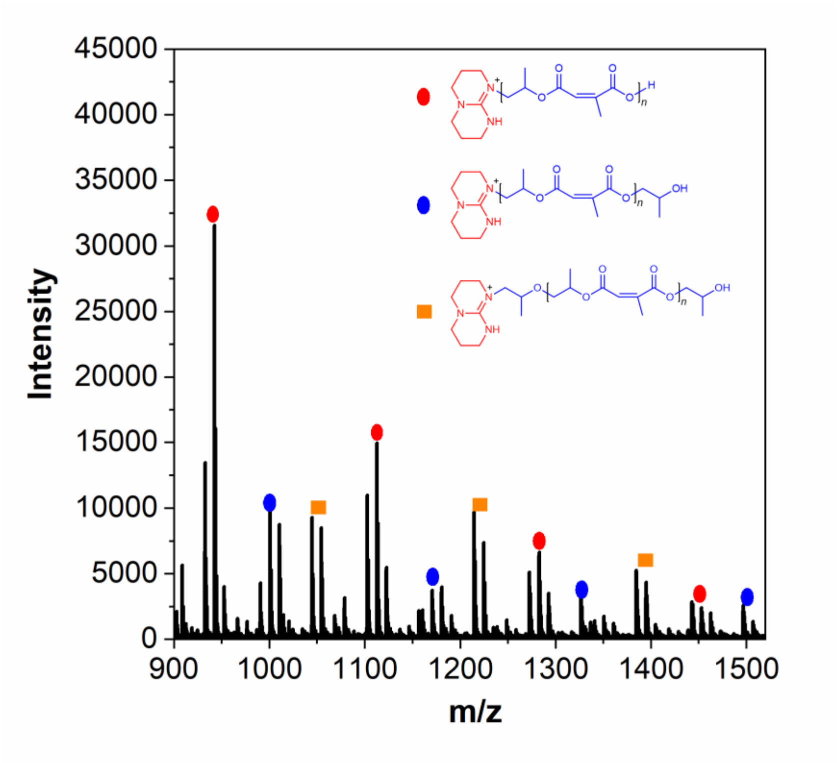

Supplementary Figure 63. MALDI-TOF mass spectrum of **P2-2.0TBD**.

Structural characterization of M1 and M2

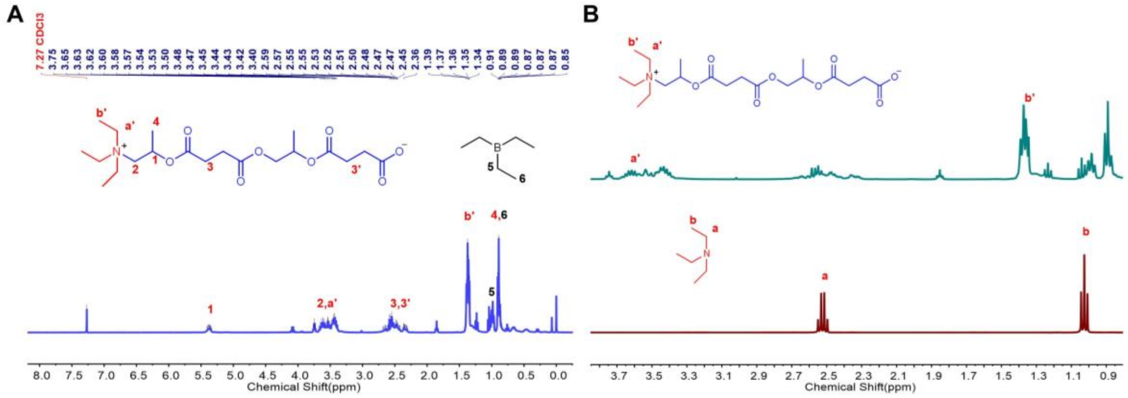

Supplementary Figure 64. (A) ^{1}H NMR spectra of crude product **M1** and (B) stacked ^{1}H NMR spectra of crude product **M1** and TEA (400 MHz, $CDCl_3$).

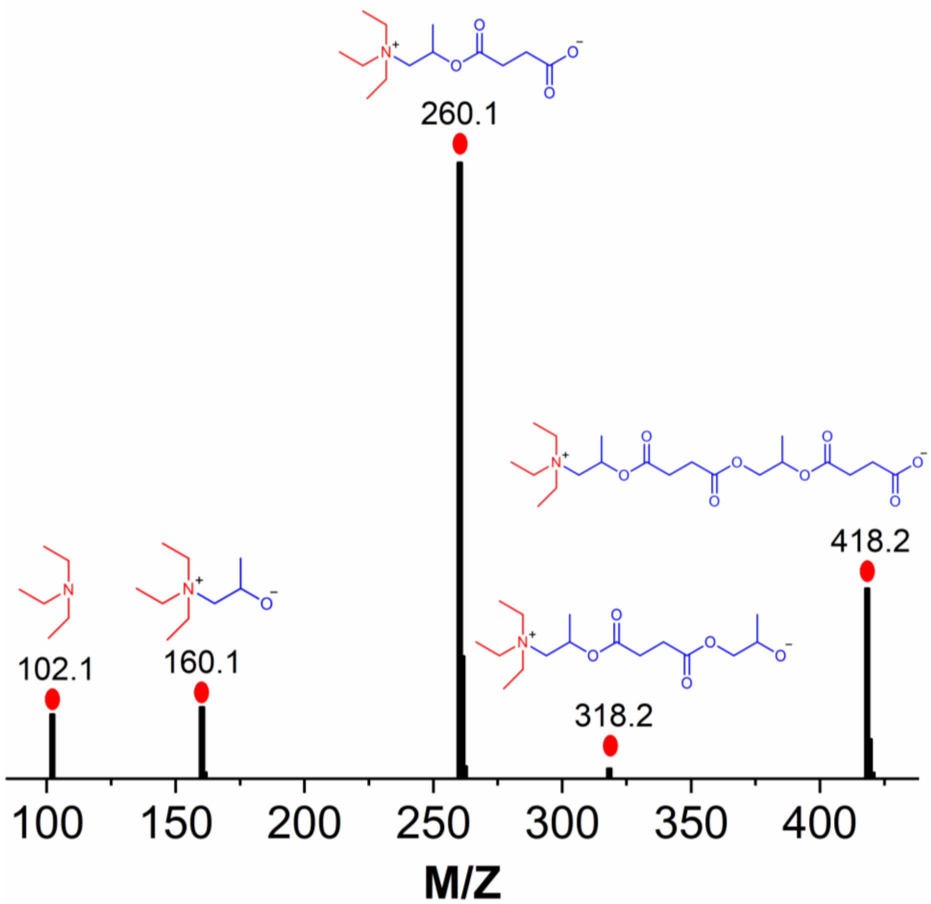

Supplementary Figure 65. ESI-MS spectra of crude product **M1** in THF of 5mg/mL.

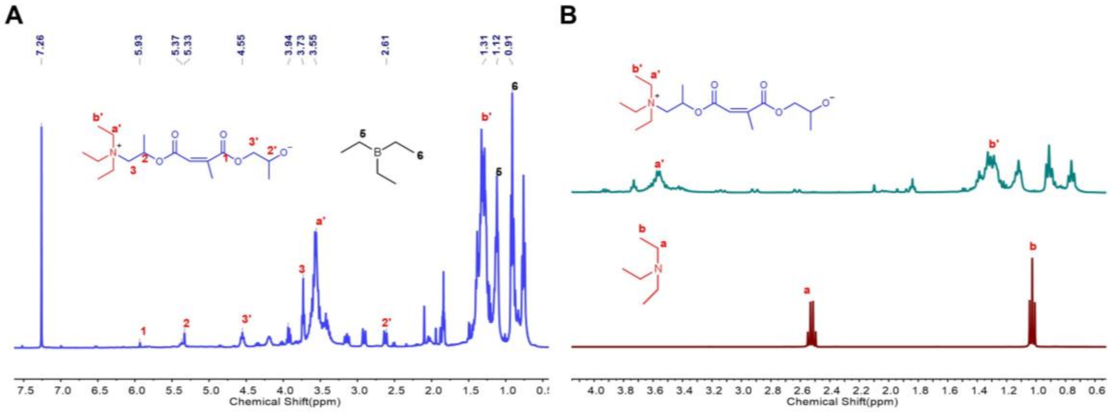

Supplementary Figure 66. (A) ^{1}H NMR spectra of crude product **M2** and (B) stacked ^{1}H NMR spectra of crude product **M2** and TEA (400 MHz, $CDCl_3$).

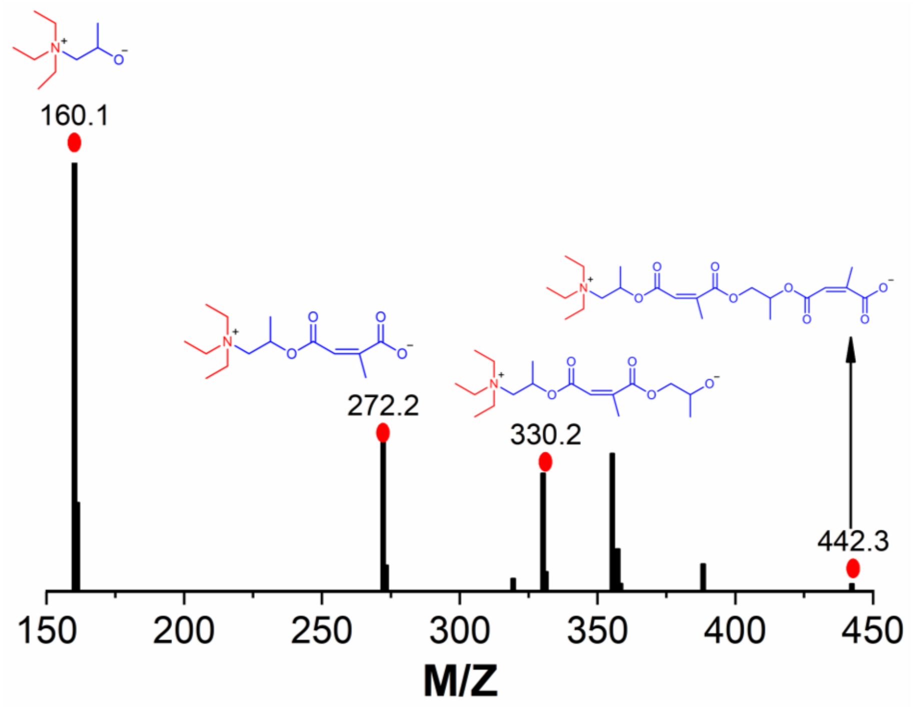

Supplementary Figure 67. ESI-MS spectra of crude product **M2** in THF of 5mg/mL.

Photophysical characterization of P1~P6

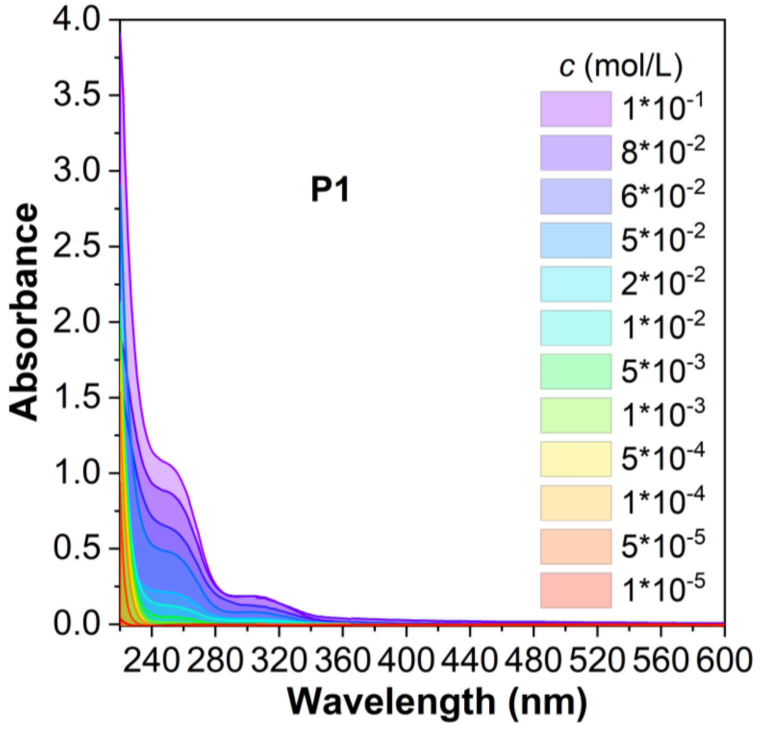

Supplementary Figure 68. Concentration-dependent UV-Vis absorption spectra of **P1** in DCM solution.

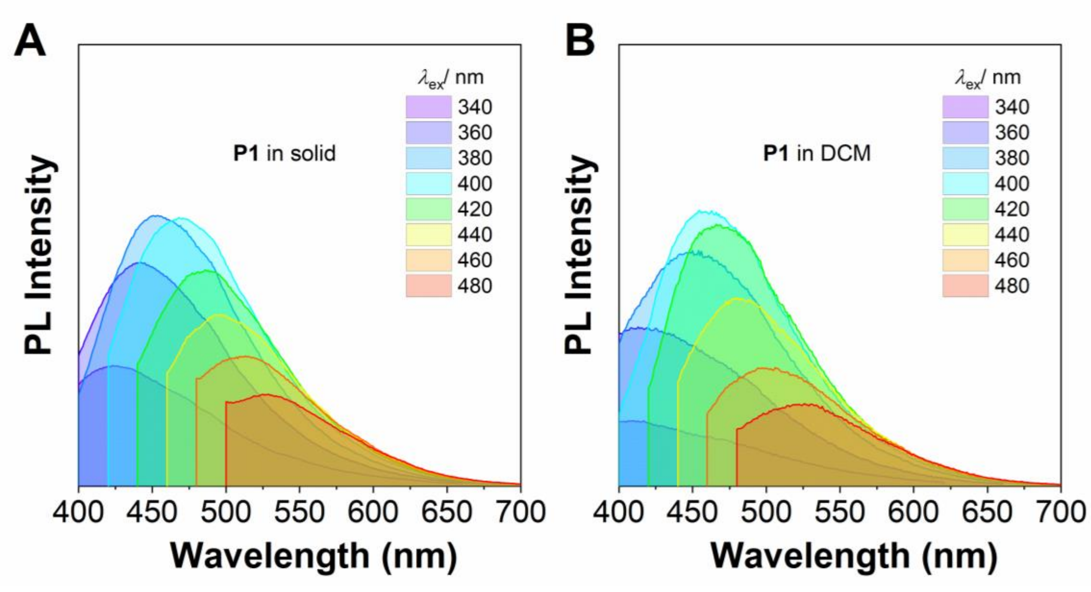

Supplementary Figure 69. PL spectra of **P1** in (A) solid and (B) DCM solution of 10^{-1}M.

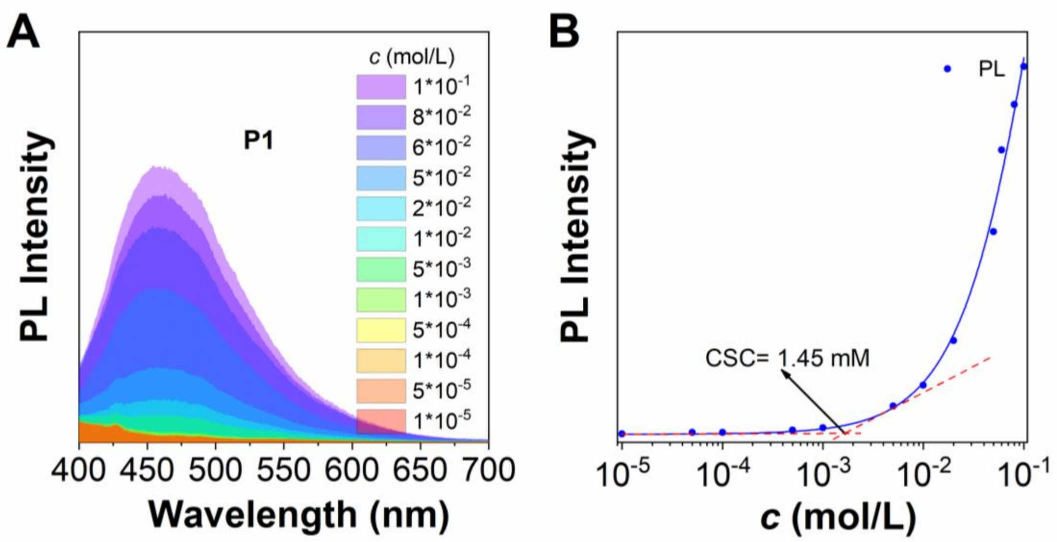

Supplementary Figure 70. Concentration-dependent spectra of P1 in DCM solution, λ_{ex} = 380 nm. (A) PL spectra at 460 nm, (B) PL intensity plots. Concentration: from 10^{-5} to 10^{-1} M.

Influence of TEB on CL

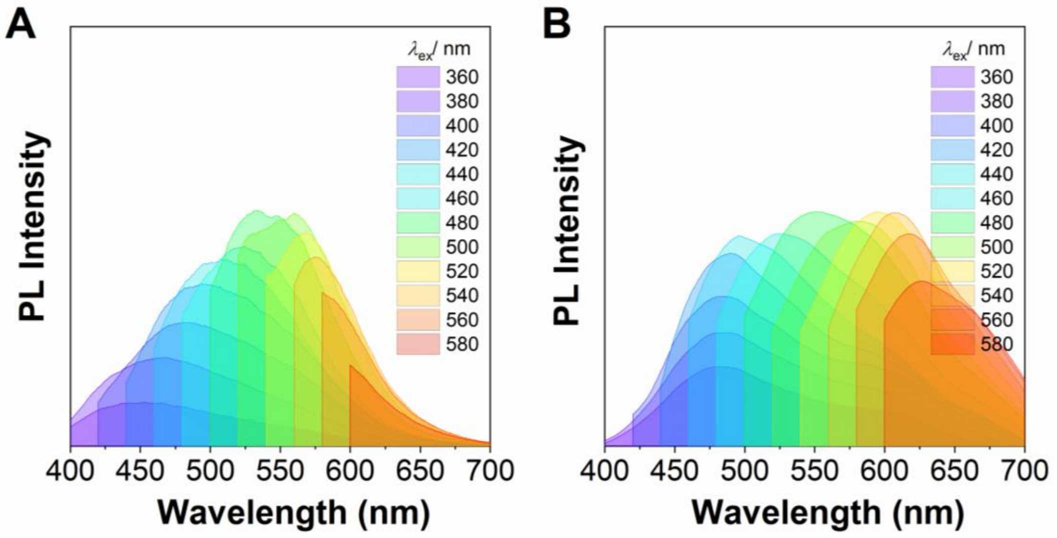

Supplementary Figure 71. PL spectra of **P1** catalyzed by TEB-TEA pair (A) and TEA (B) in solid at different excitation wavelength. The molar ratios of PO, SA, TEB and TEA is 2:1:0.02:0.02.

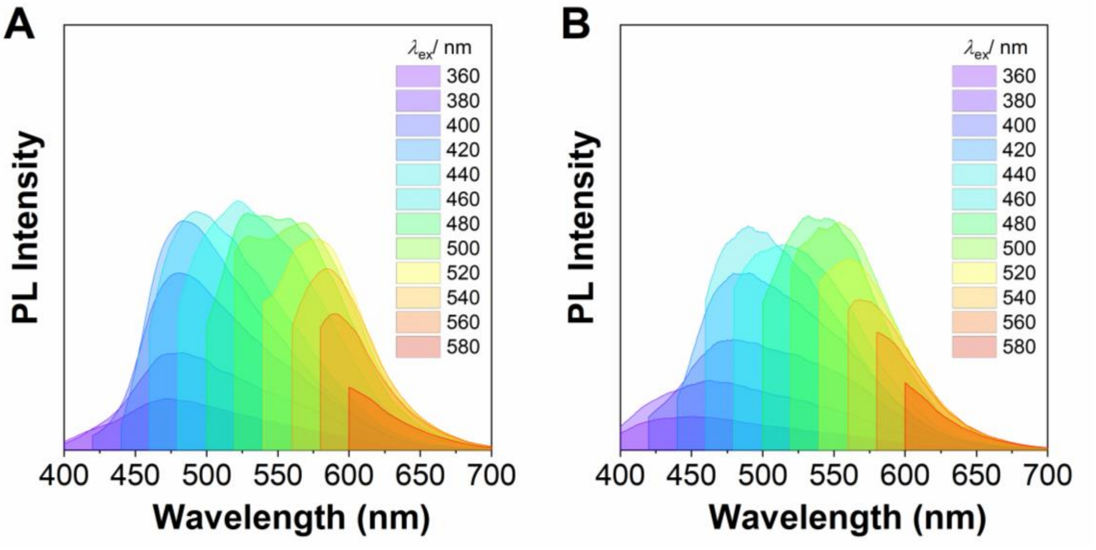

Supplementary Figure 72. PL spectra of **P1** catalyzed by TEB-TEA pair (A) and TEA (B) in DCM solution of 10^{-1}M. The molar ratios of PO, SA, TEB and TEA is 2:1:0.02:0.02.

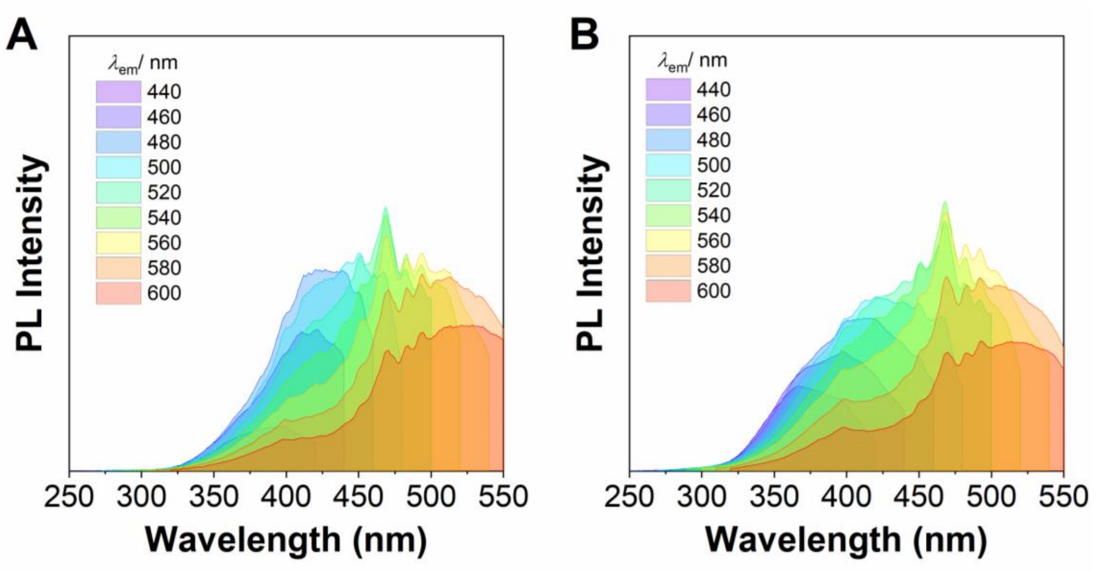

Supplementary Figure 73. Excitation spectra of **P1** catalyzed by TEB-TEA pair (A) and TEA (B) in DCM solution of 10^{-1}M. The molar ratios of PO, SA, TEB and TEA is 2:1:0.02:0.02.

Photophysical characterization of P1-aTEA (a% is from 0.5% to 5.0%)

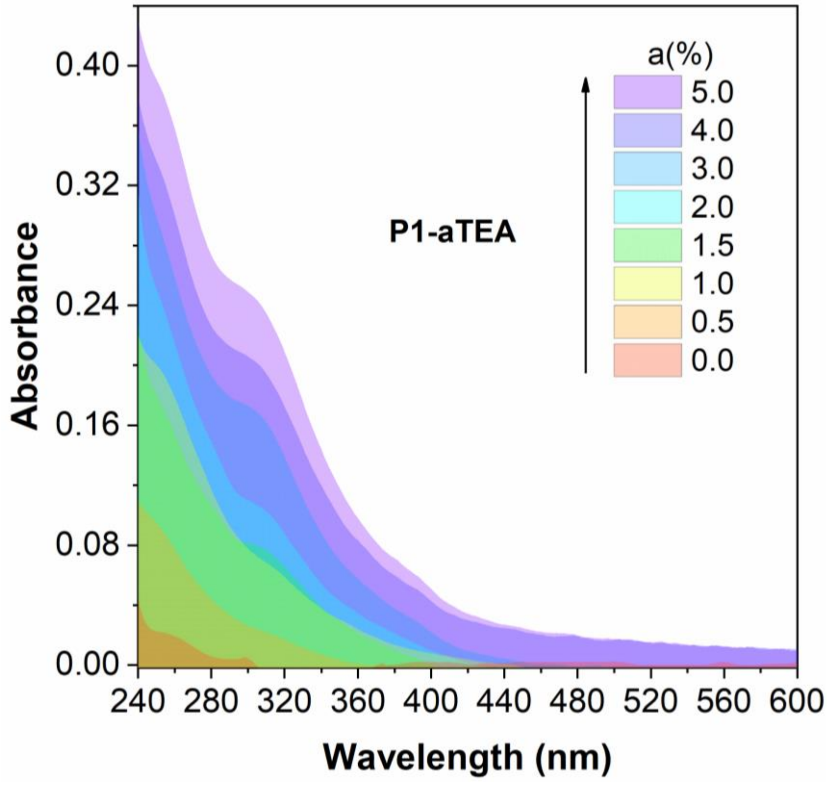

Supplementary Figure 74. UV-Vis absorption spectra of **P1-aTEA** in DCM solution of 10^{-3}M.

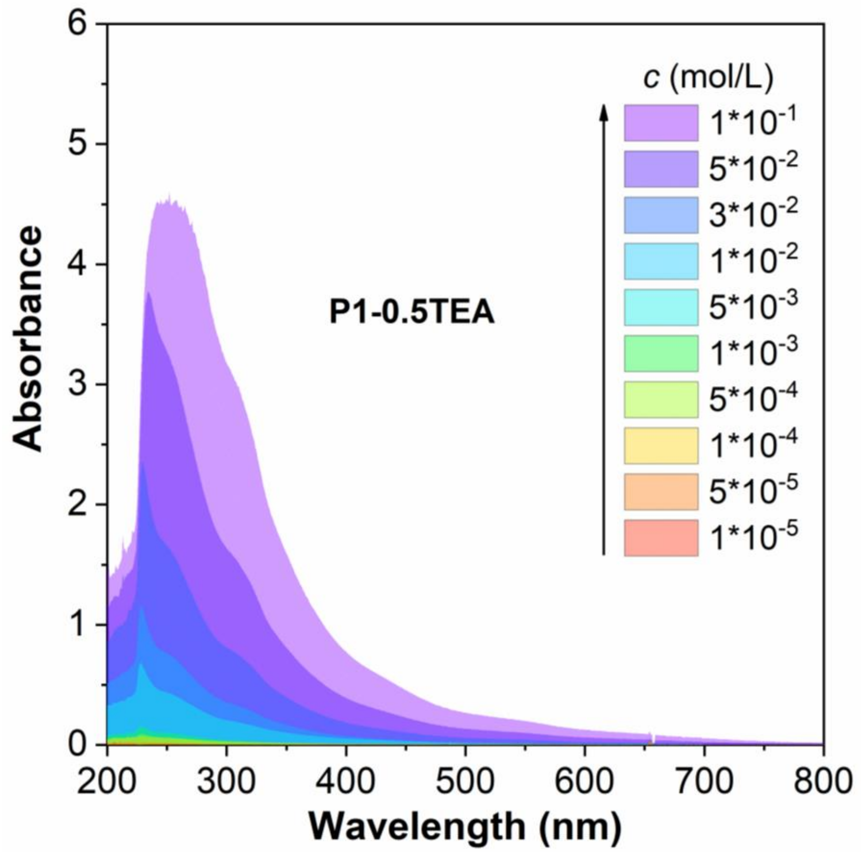

Supplementary Figure 75. Concentration-dependent UV-Vis absorption spectra of **P1-0.5TEA** in DCM solution. Concentration: from 10^{-5} to 10^{-1} M.

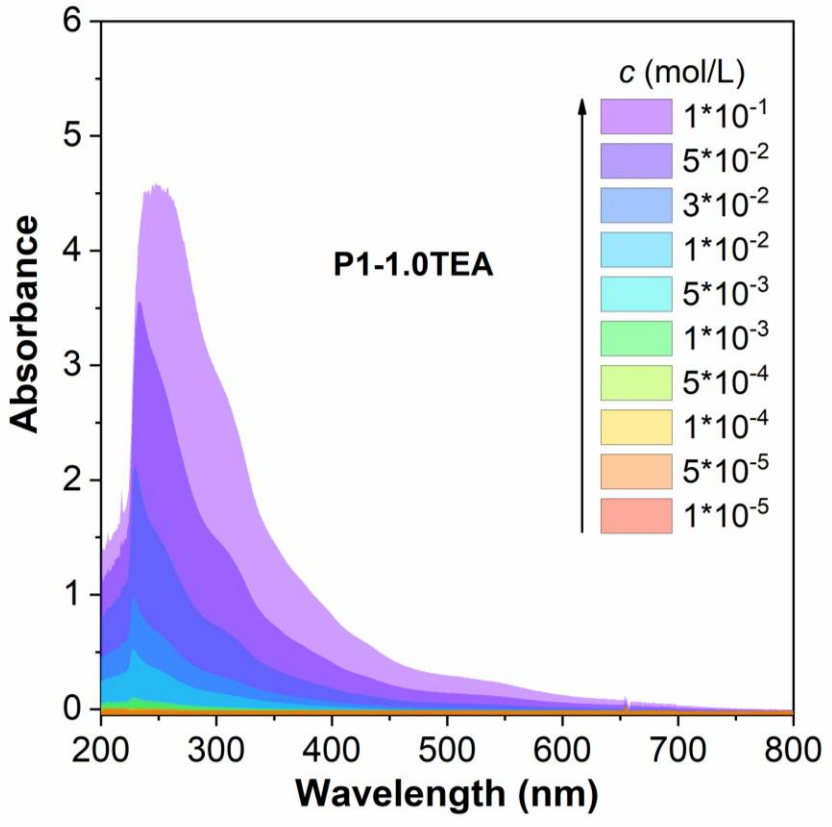

Supplementary Figure 76. Concentration-dependent UV-Vis absorption spectra of **P1-1.0TEA** in DCM solution. Concentration: from 10^{-5} to 10^{-1} M.

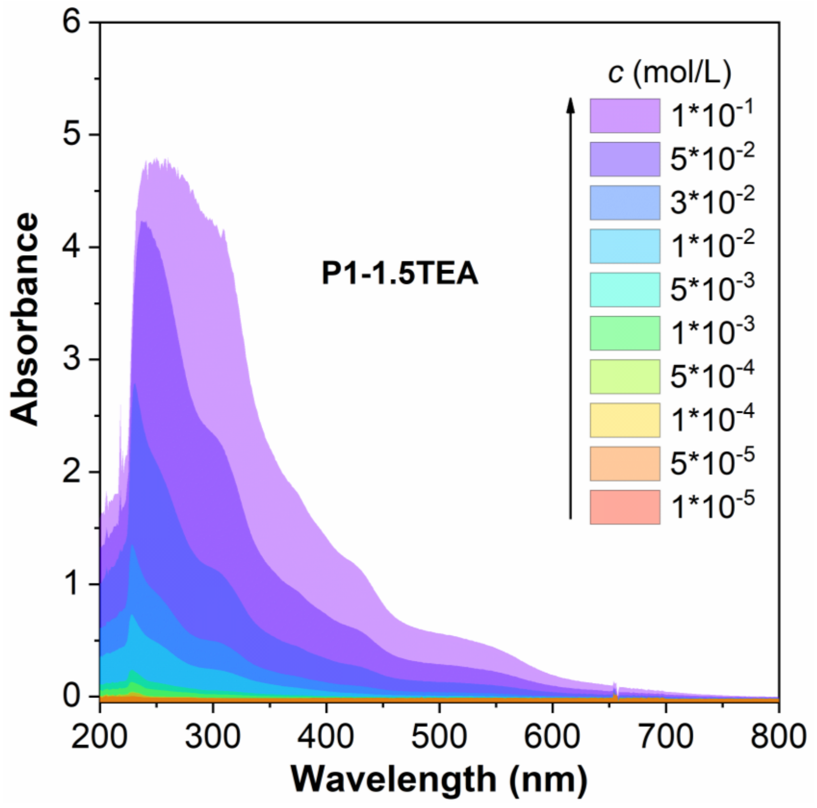

Supplementary Figure 77. Concentration-dependent UV-Vis absorption spectra of **P1-1.5TEA** in DCM solution. Concentration: from 10^{-5} to 10^{-1} M.

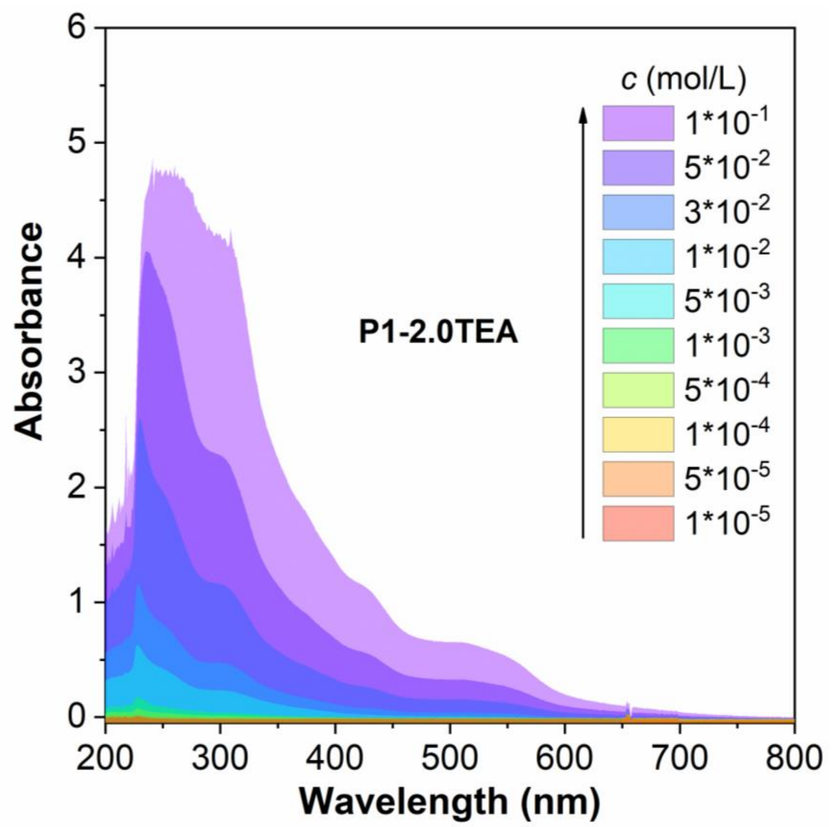

Supplementary Figure 78. Concentration-dependent UV-Vis absorption spectra of **P1-2.0TEA** in DCM solution. Concentration: from 10^{-5} to 10^{-1} M.

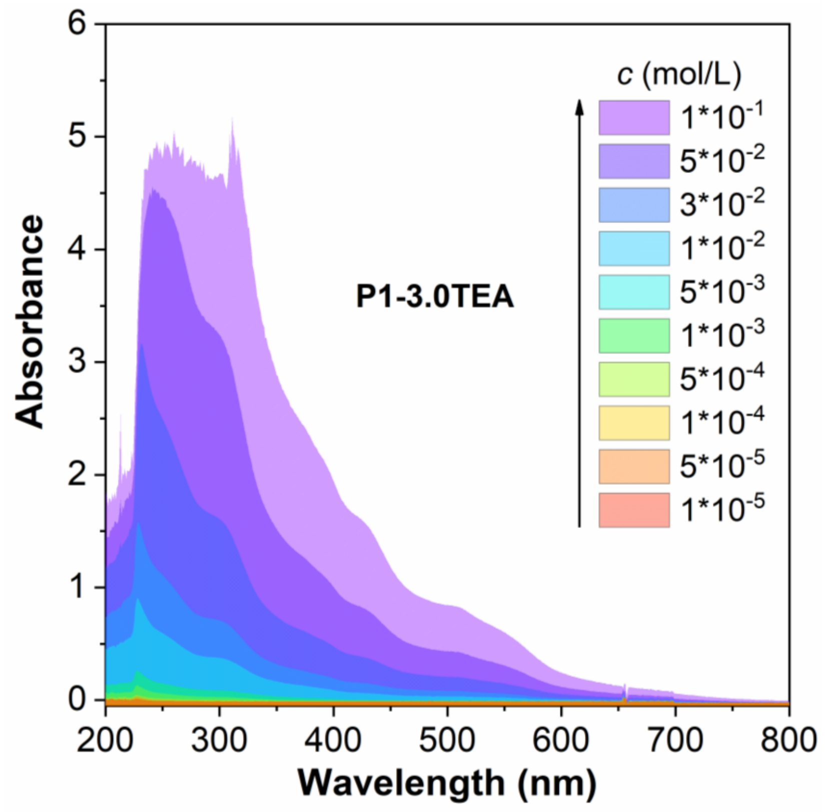

Supplementary Figure 79. Concentration-dependent UV-Vis absorption spectra of **P1-3.0TEA** in DCM solution. Concentration: from 10^{-5} to 10^{-1} M.

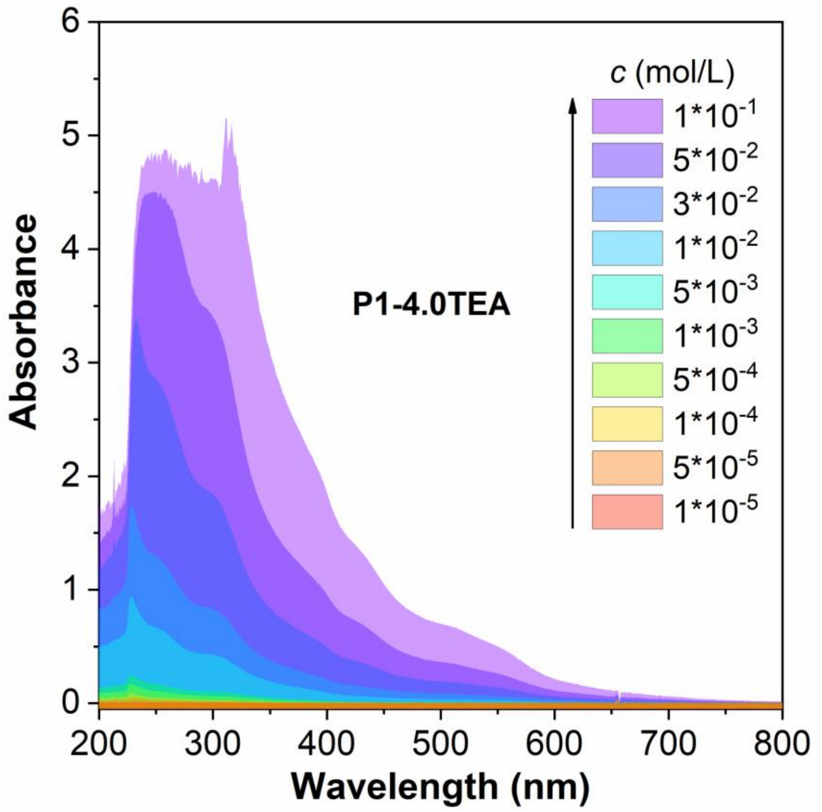

Supplementary Figure 80. Concentration-dependent UV-Vis absorption spectra of **P1-4.0TEA** in DCM solution. Concentration: from 10^{-5} to 10^{-1} M.

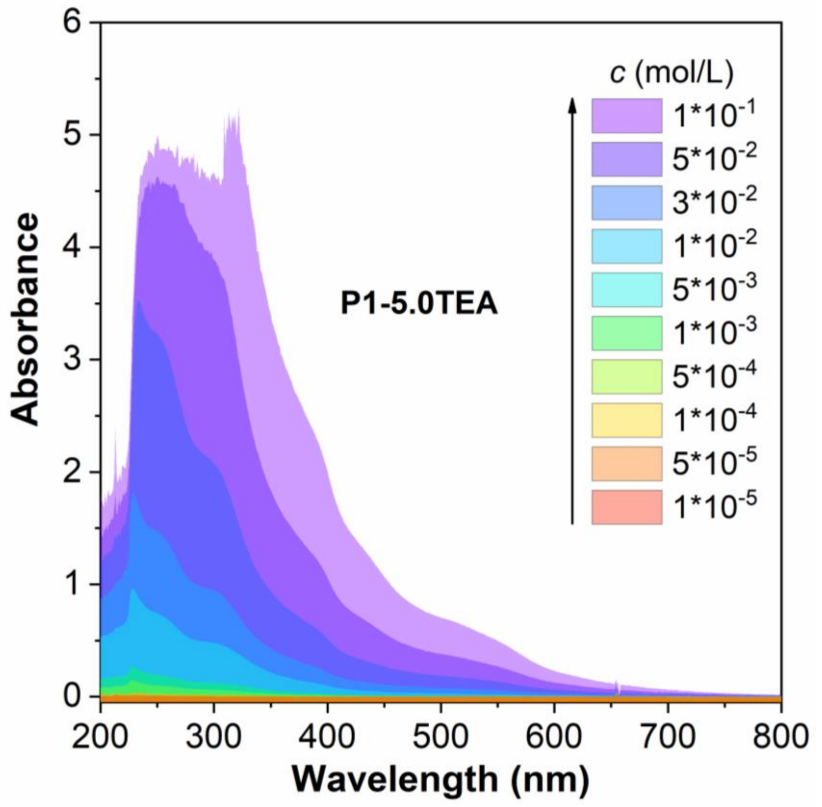

Supplementary Figure 81. Concentration-dependent UV-Vis absorption spectra of **P1-5.0TEA** in DCM solution. Concentration: from 10^{-5} to 10^{-1} M.

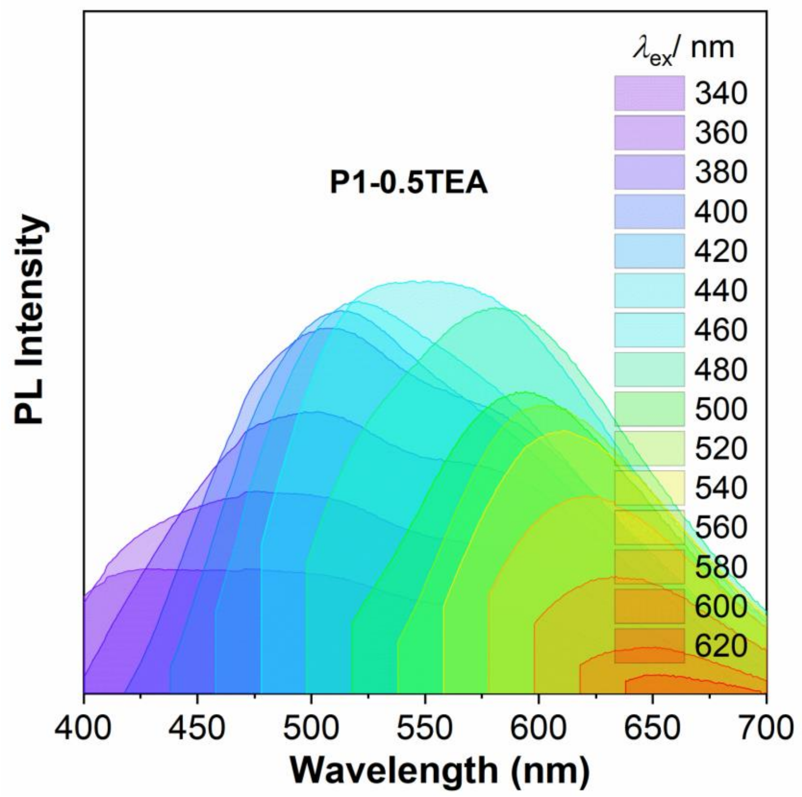

Supplementary Figure 82. PL spectra of **P1-0.5TEA** in solid under different excitation wavelengths.

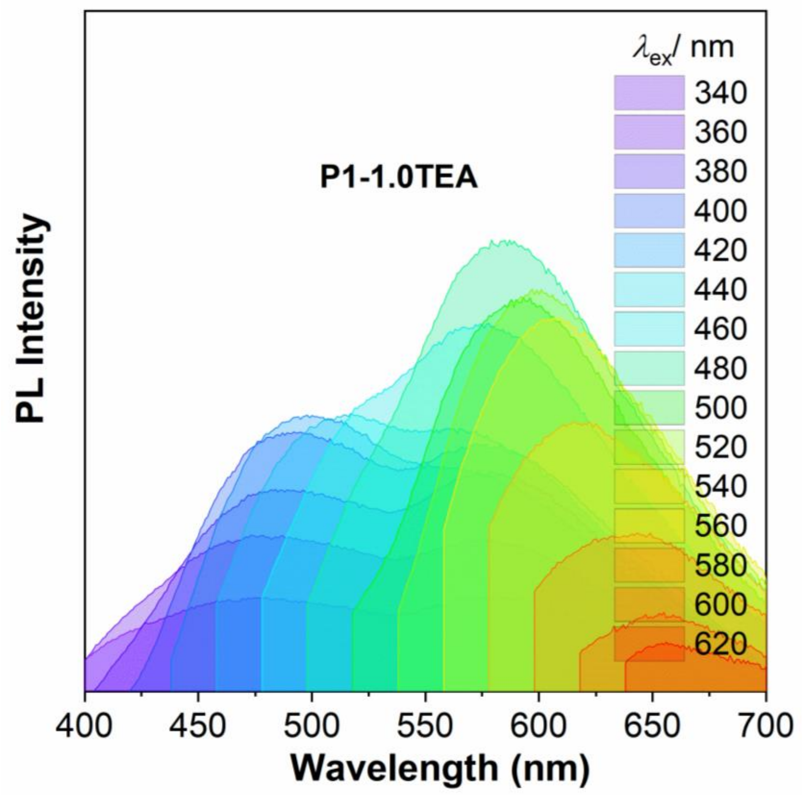

Supplementary Figure 83. PL spectra of **P1-1.0TEA** in solid under different excitation wavelengths.

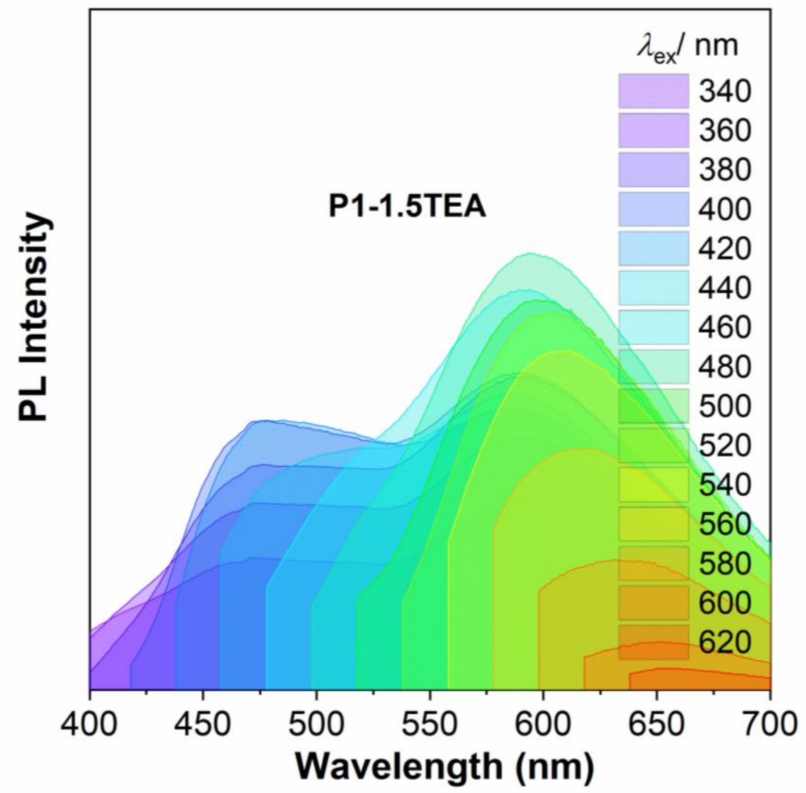

Supplementary Figure 84. PL spectra of **P1-1.5TEA** in solid under different excitation wavelengths.

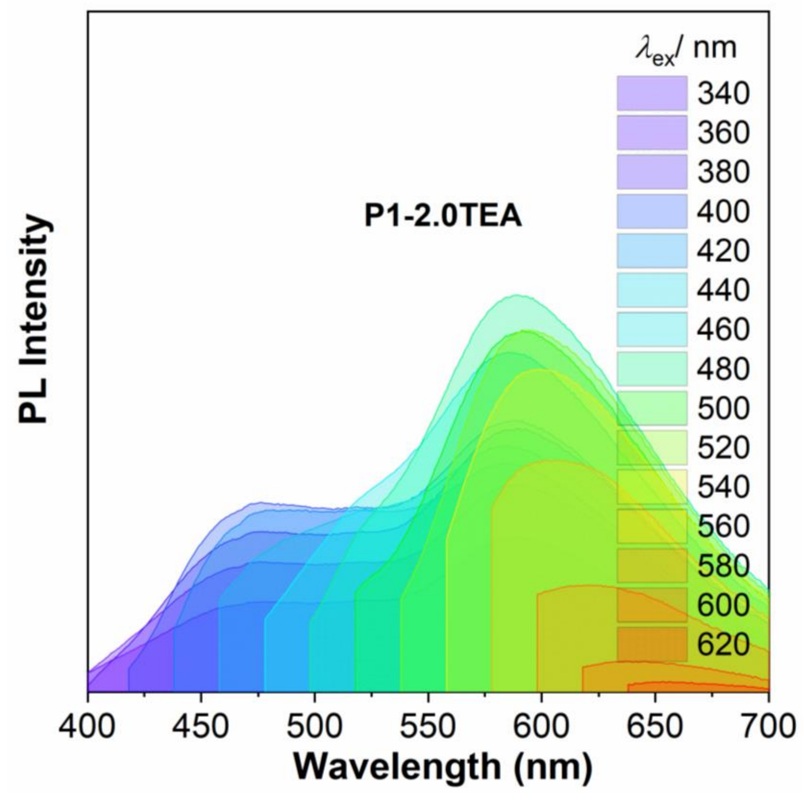

Supplementary Figure 85. PL spectra of **P1-2.0TEA** in solid under different excitation wavelengths.

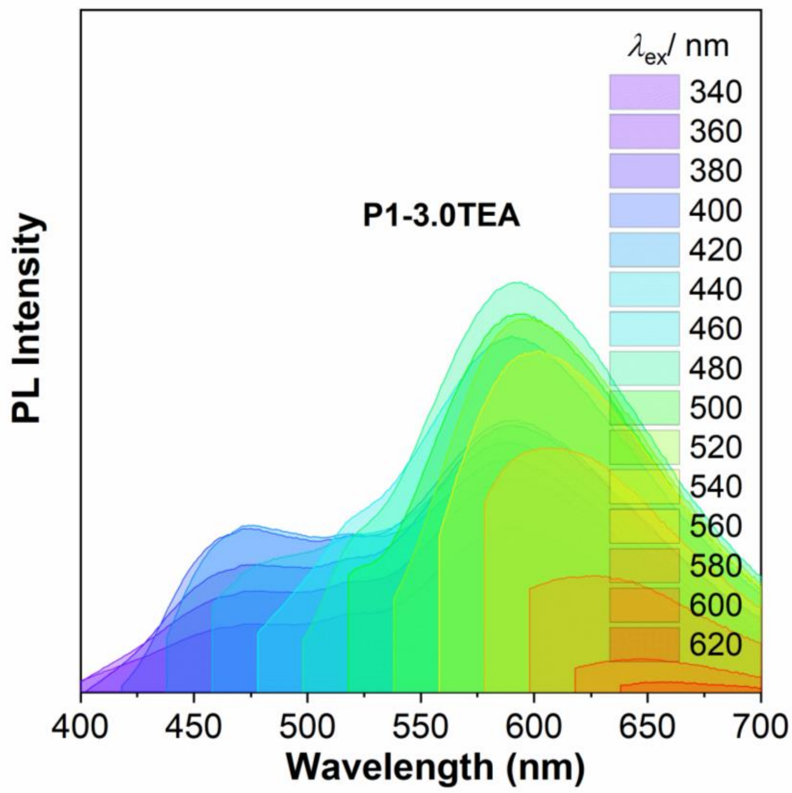

Supplementary Figure 86. PL spectra of **P1-3.0TEA** in solid under different excitation wavelengths.

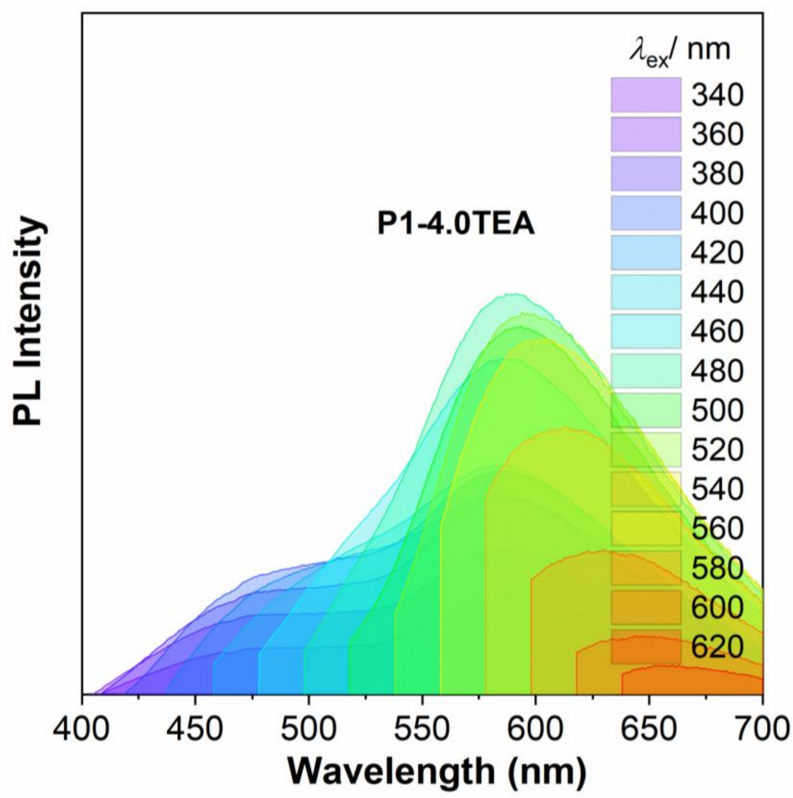

Supplementary Figure 87. PL spectra of **P1-4.0TEA** in solid under different excitation wavelengths.

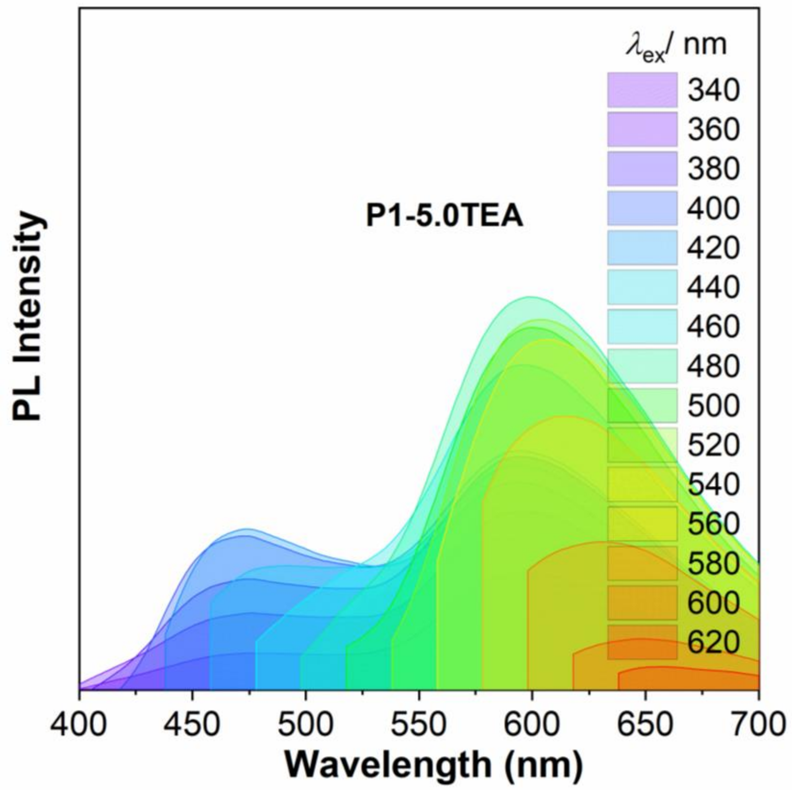

Supplementary Figure 88. PL spectra of **P1-5.0TEA** in solid under different excitation wavelengths.

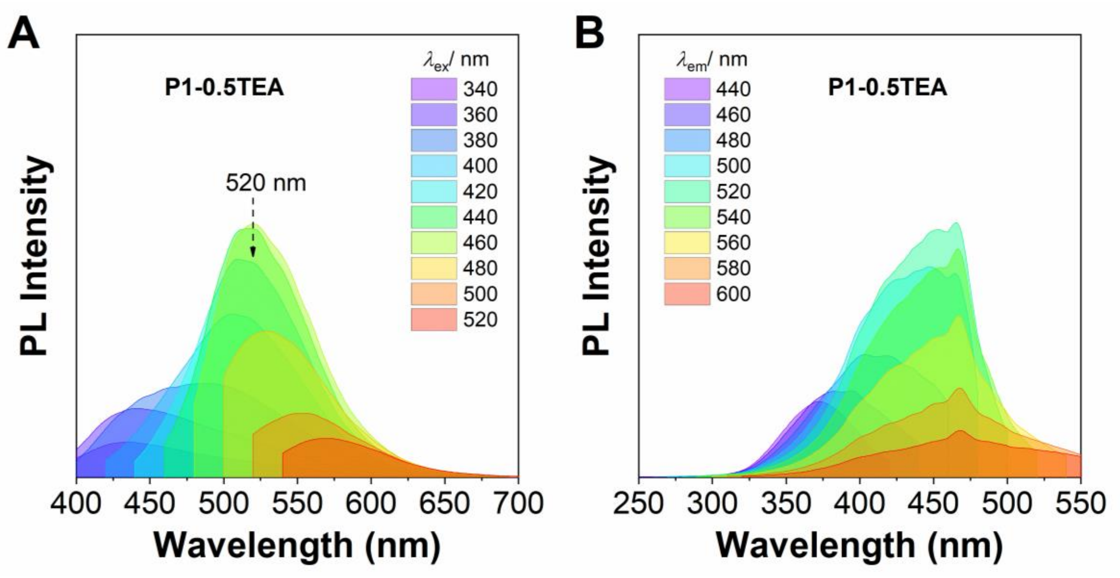

Supplementary Figure 89. PL spectra (A) under different excitation wavelengths and excitation spectra (B) under different emission wavelengths of **P1-0.5TEA** in DCM solution of 10^{-1} M.

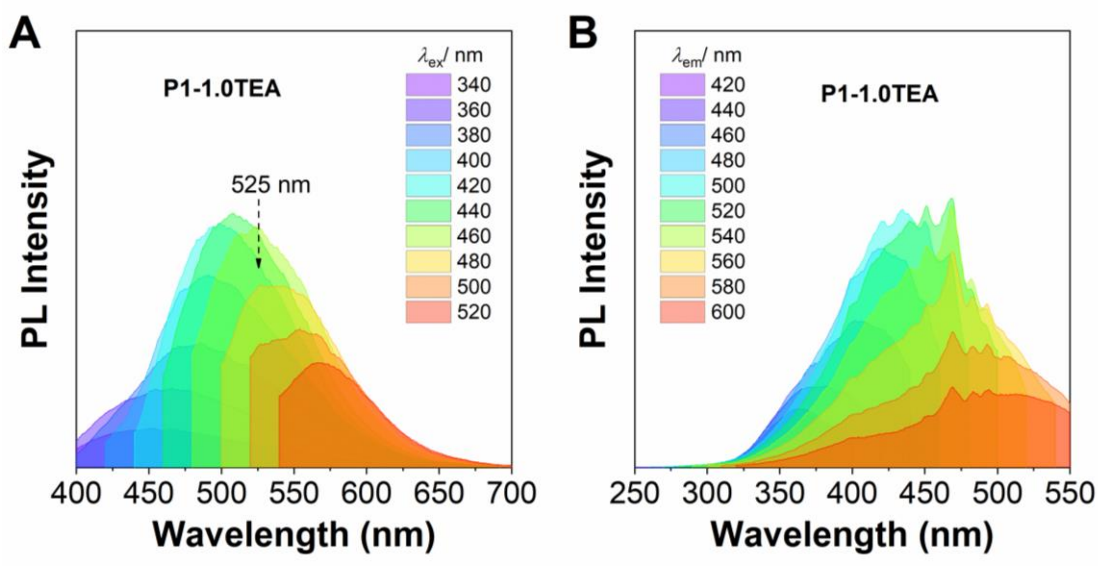

Supplementary Figure 90. PL spectra (A) under different excitation wavelengths and excitation spectra (B) under different emission wavelengths of **P1-1.0TEA** in DCM solution of 10^{-1} M.

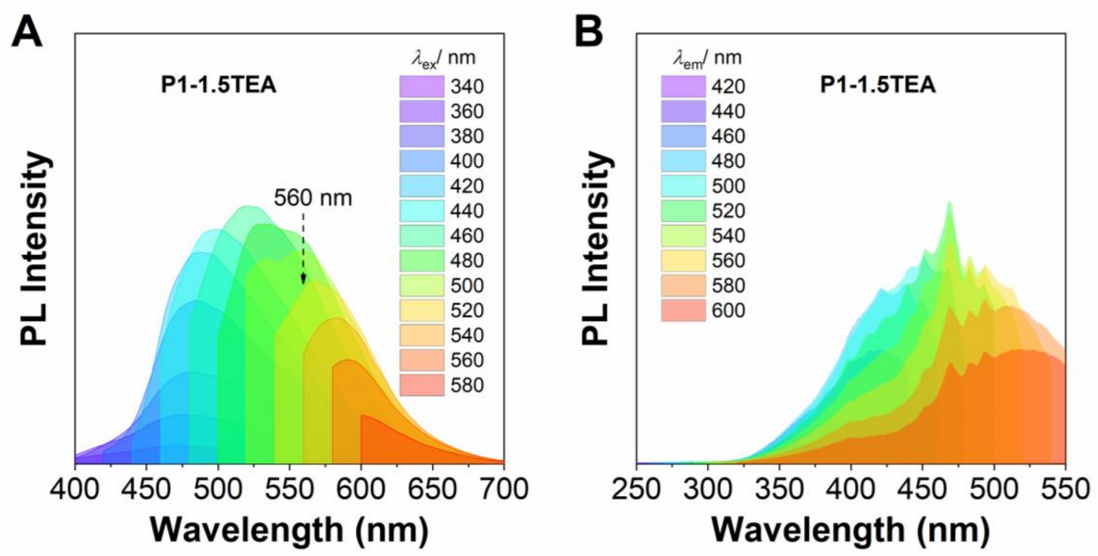

Supplementary Figure 91. PL spectra (A) under different excitation wavelengths and excitation spectra (B) under different emission wavelengths of **P1-1.5TEA** in DCM solution of 10^{-1} M.

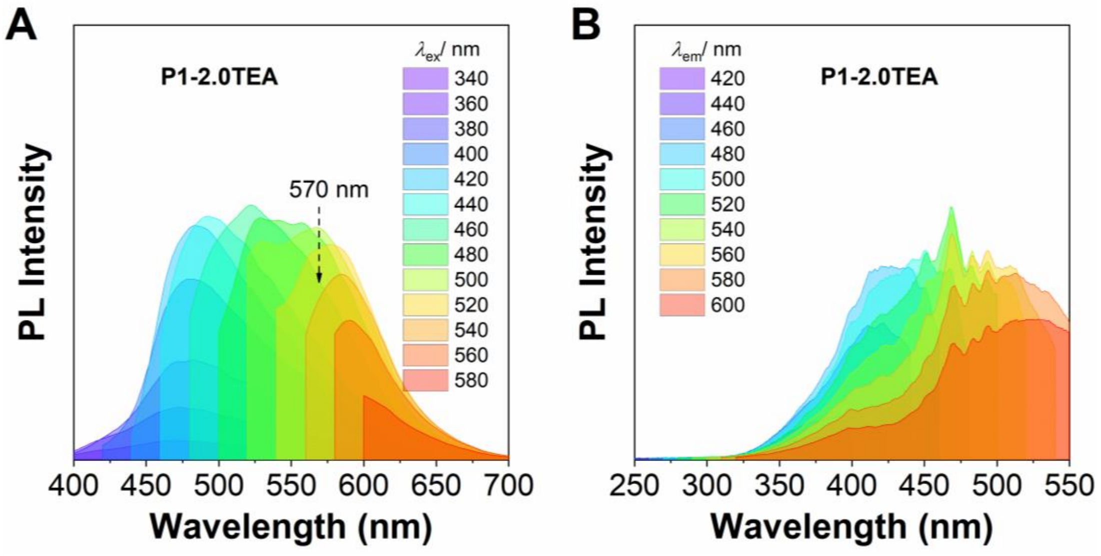

Supplementary Figure 92. PL spectra (A) under different excitation wavelengths and excitation spectra (B) under different emission wavelengths of **P1-2.0TEA** in DCM solution of 10^{-1} M.

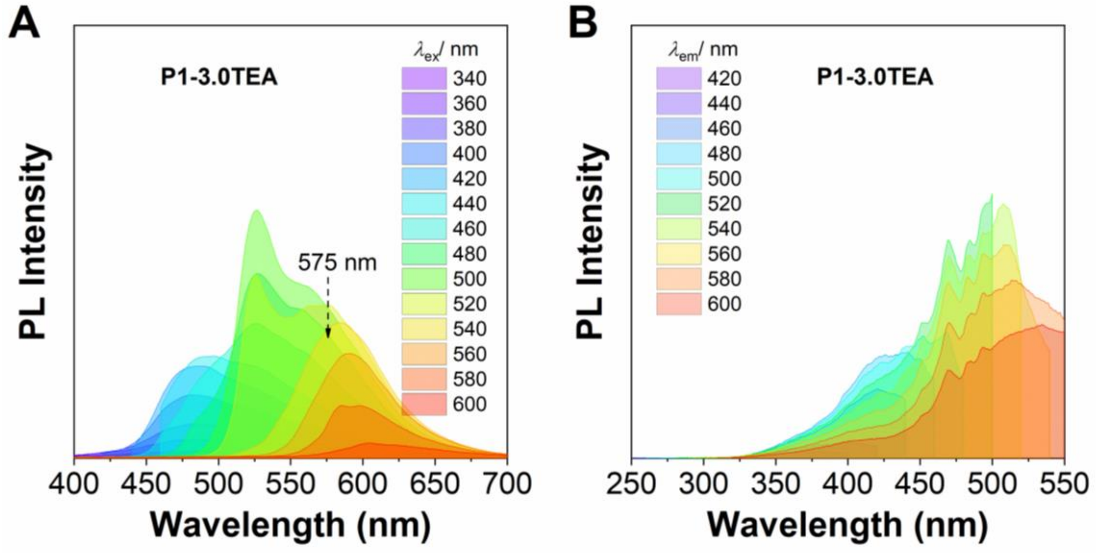

Supplementary Figure 93. PL spectra (A) under different excitation wavelengths and excitation spectra (B) under different emission wavelengths of **P1-3.0TEA** in DCM solution of 10^{-1} M.

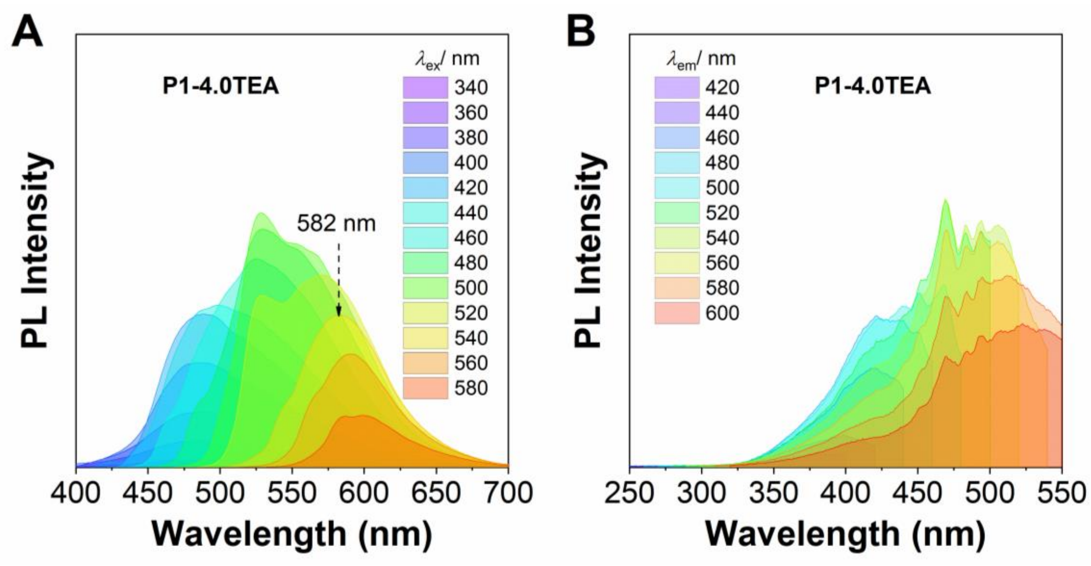

Supplementary Figure 94. PL spectra (A) under different excitation wavelengths and excitation spectra (B) under different emission wavelengths of **P1-4.0TEA** in DCM solution of 10^{-1} M.

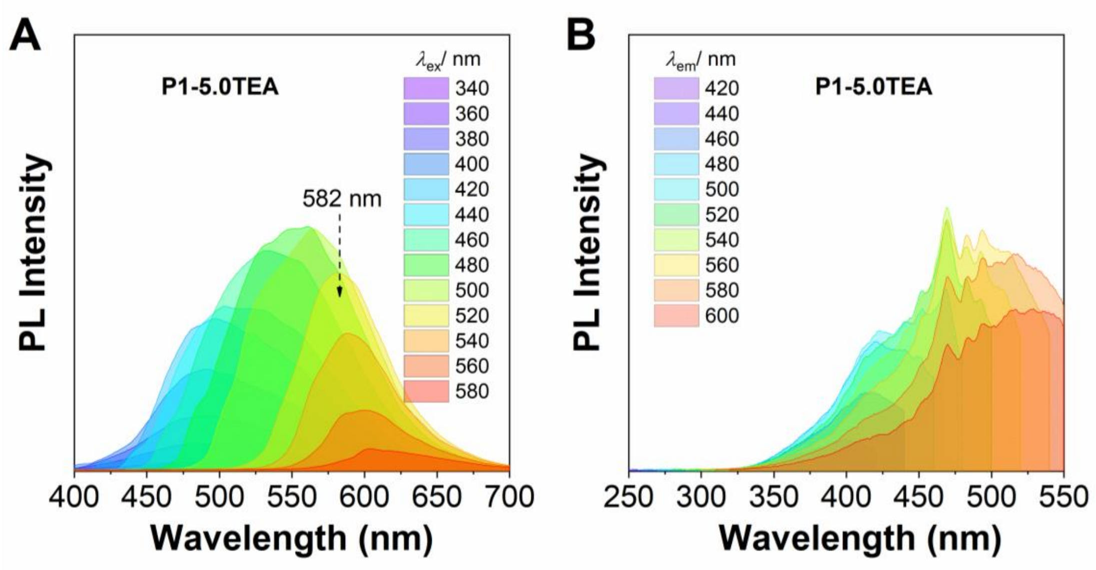

Supplementary Figure 95. PL spectra (A) under different excitation wavelengths and excitation spectra (B) under different emission wavelengths of **P1-5.0TEA** in DCM solution of 10^{-1} M.

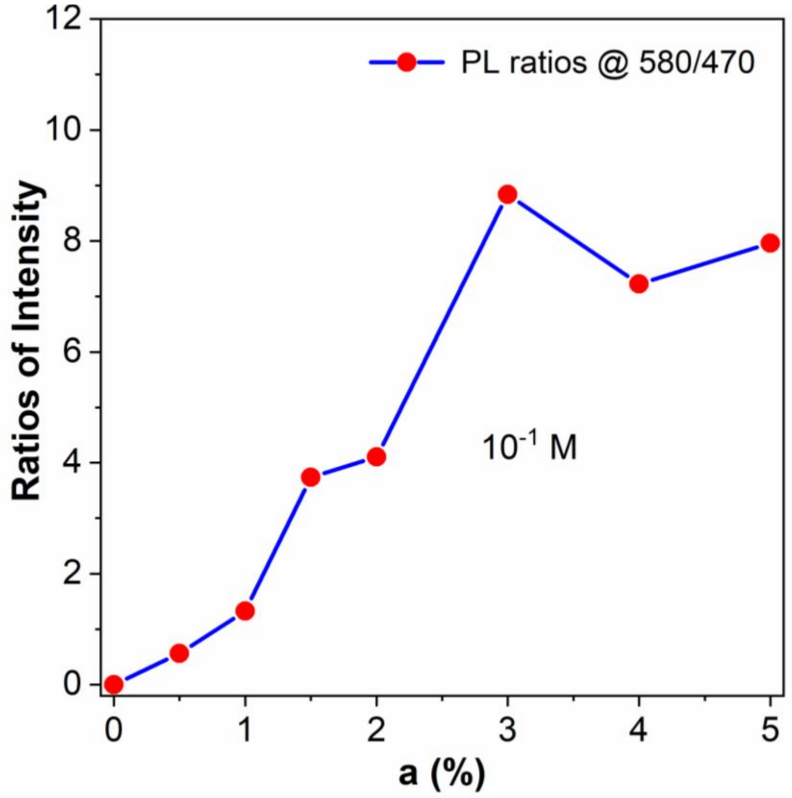

Supplementary Figure 96. Plots of ratios of intensities versuscontents of TEA for **P1-aTEA** in DCM solution of 10^{-1} M. The intensity ratios are of long-wavelength CL (λ_{ex} = 520 nm) to short-wavelength PL (λ_{ex} = 360 nm).

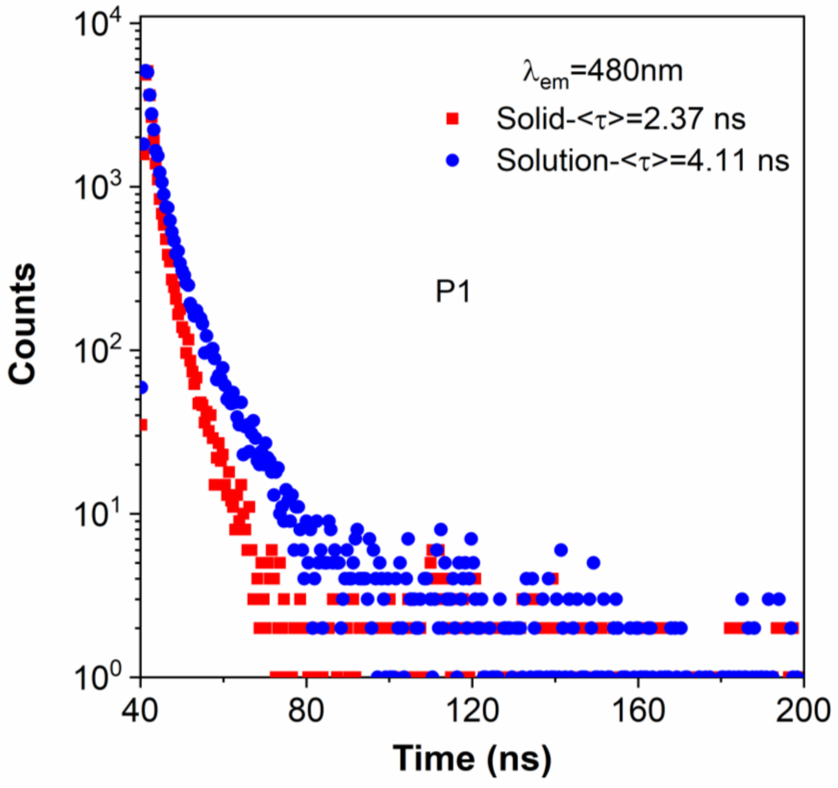

Supplementary Figure 97. Time-resolved PL decay curves of **P1-0.5TEA** measured at emission maximum of 480 nm in solid and DCM solution (c = 10^{-3} M), λ_{ex} = 365 nm.

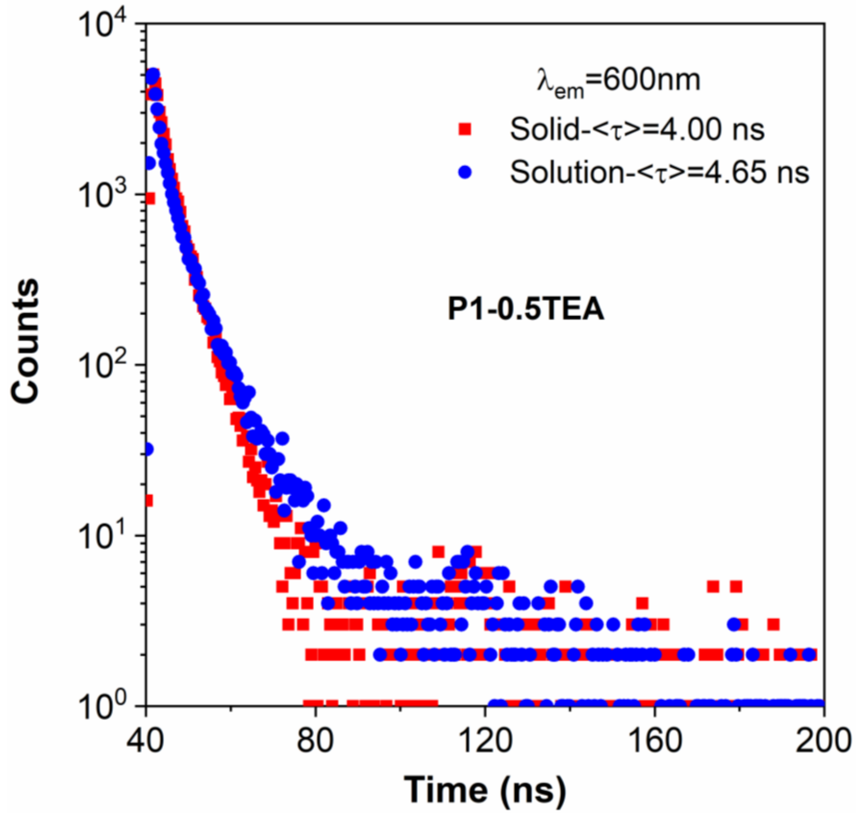

Supplementary Figure 98. Time-resolved PL decay curves of **P1-0.5TEA** measured at emission maximum of 600 nm in solid and DCM solution (c = 10^{-3} M), λ_{ex} = 365 nm.

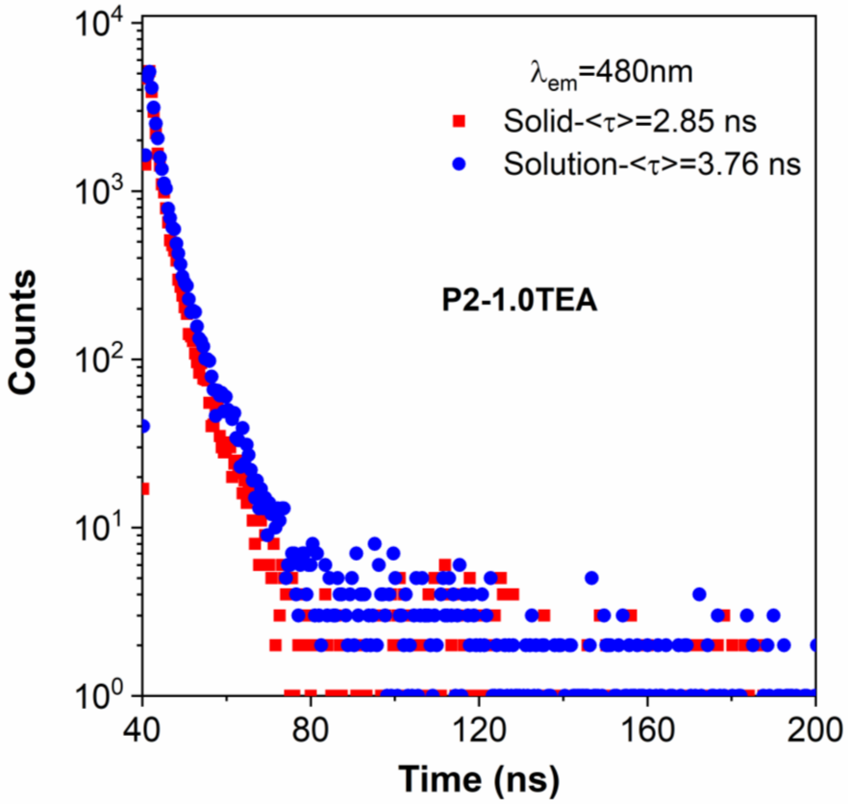

Supplementary Figure 99. Time-resolved PL decay curves of **P1-1.0TEA** measured at emission maximum of 480 nm in solid and DCM solution (c = 10^{-3} M), λ_{ex} = 365 nm.

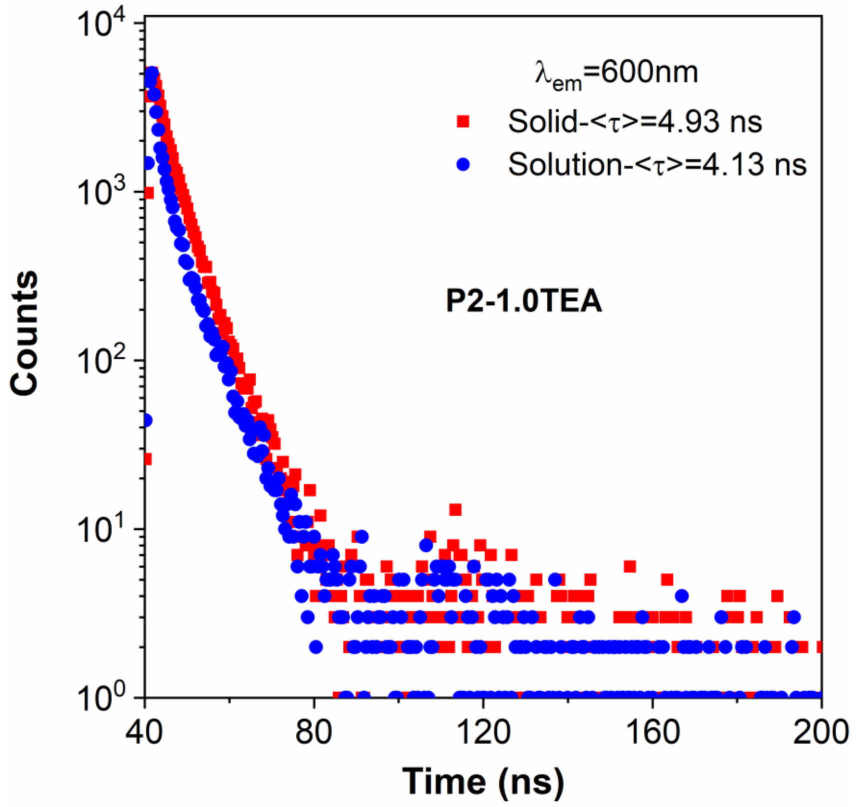

Supplementary Figure 100. Time-resolved PL decay curves of **P1-1.0TEA** measured at emission maximum of 600 nm in solid and DCM solution (c = 10^{-3} M), λ_{ex} = 365 nm.

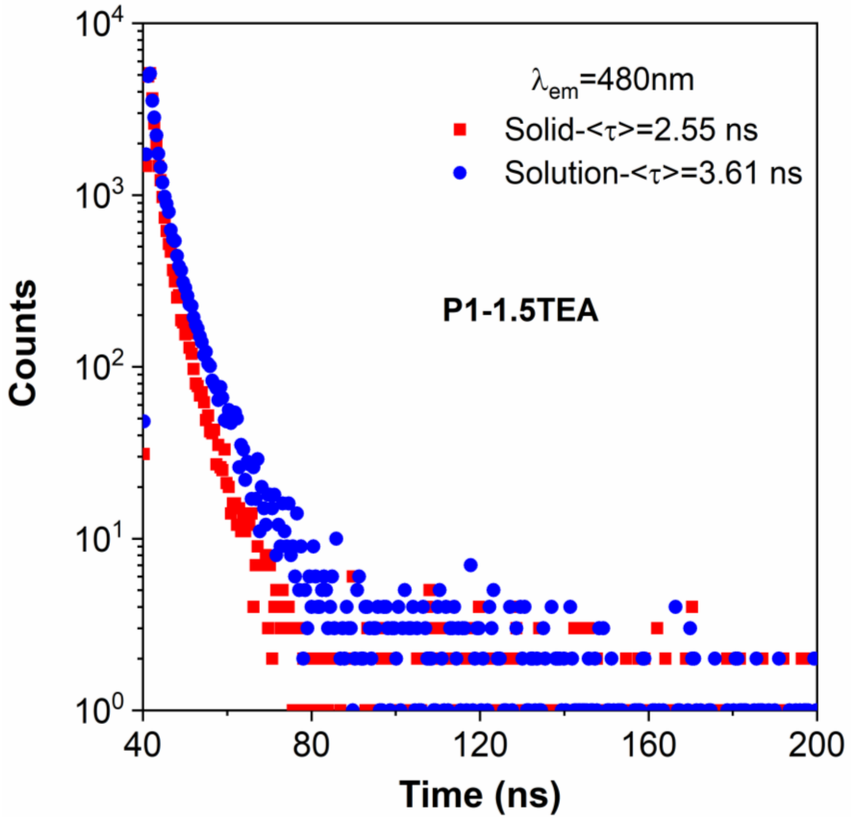

Supplementary Figure 101. Time-resolved PL decay curves of **P1-1.5TEA** measured at emission maximum of 480 nm in solid and DCM solution (c = 10^{-3} M), λ_{ex} = 365 nm.

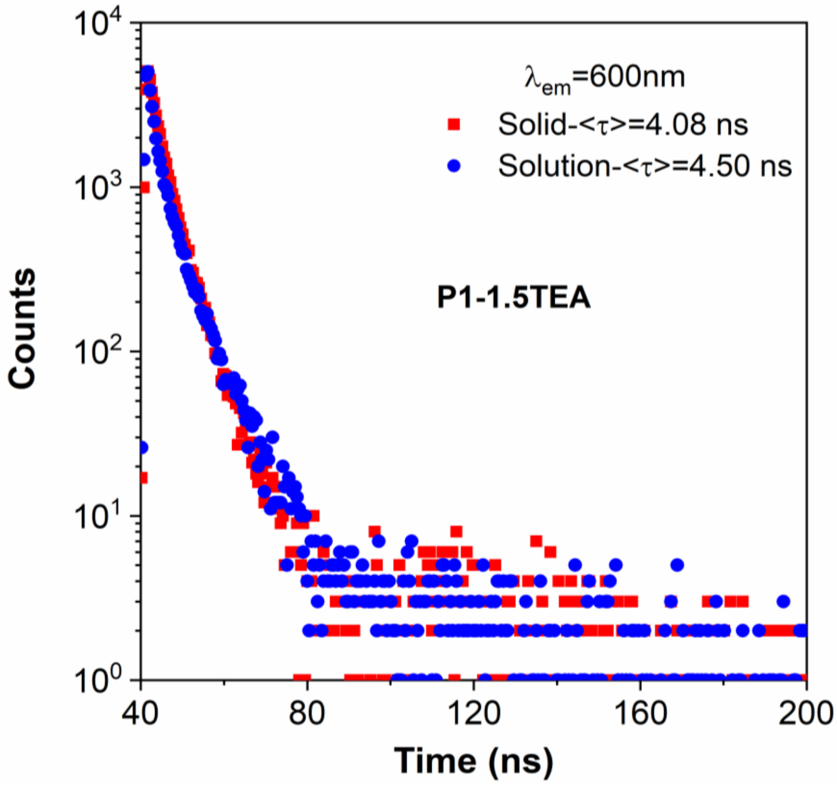

Supplementary Figure 102. Time-resolved PL decay curves of **P1-1.5TEA** measured at emission maximum of 600 nm in solid and DCM solution (c = 10^{-3} M), λ_{ex} = 365 nm.

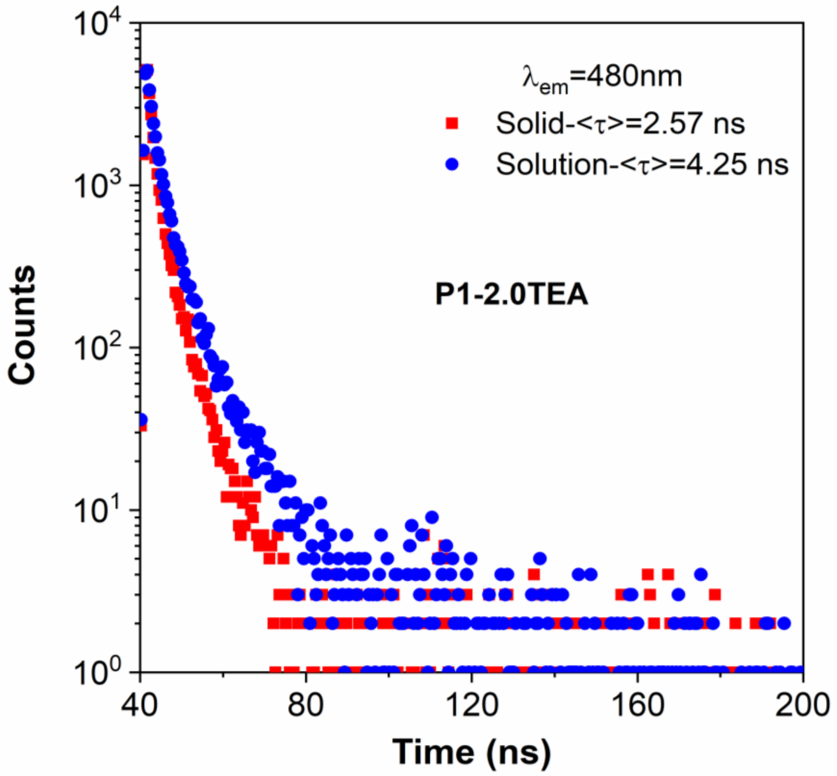

Supplementary Figure 103. Time-resolved PL decay curves of **P1-2.0TEA** measured at emission maximum of 480 nm in solid and DCM solution (c = 10^{-3} M), λ_{ex} = 365 nm.

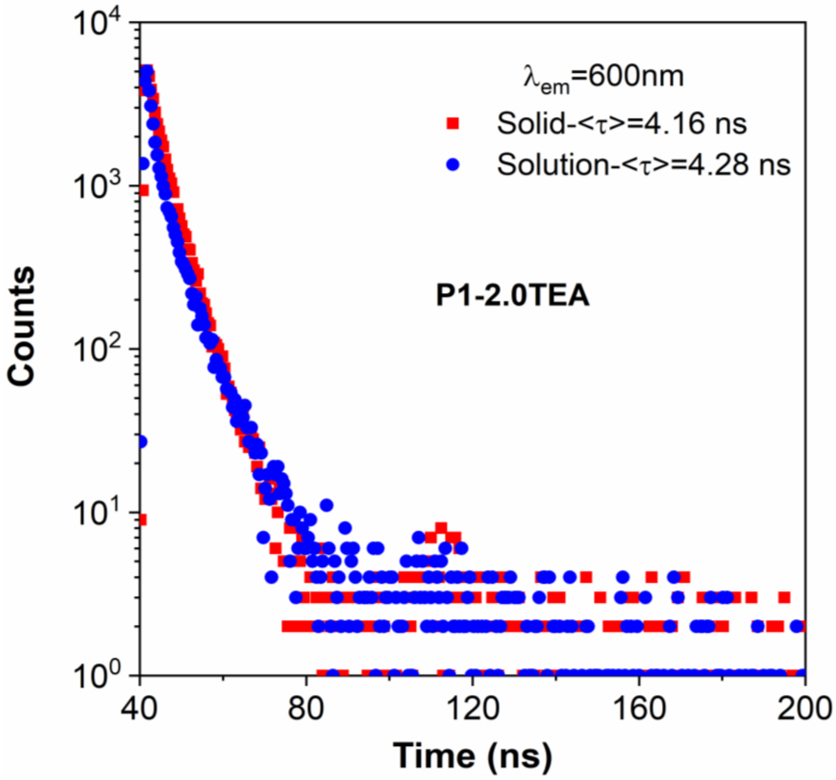

Supplementary Figure 104. Time-resolved PL decay curves of **P1-2.0TEA** measured at emission maximum of 600 nm in solid and DCM solution (c = 10^{-3} M), λ_{ex} = 365 nm.

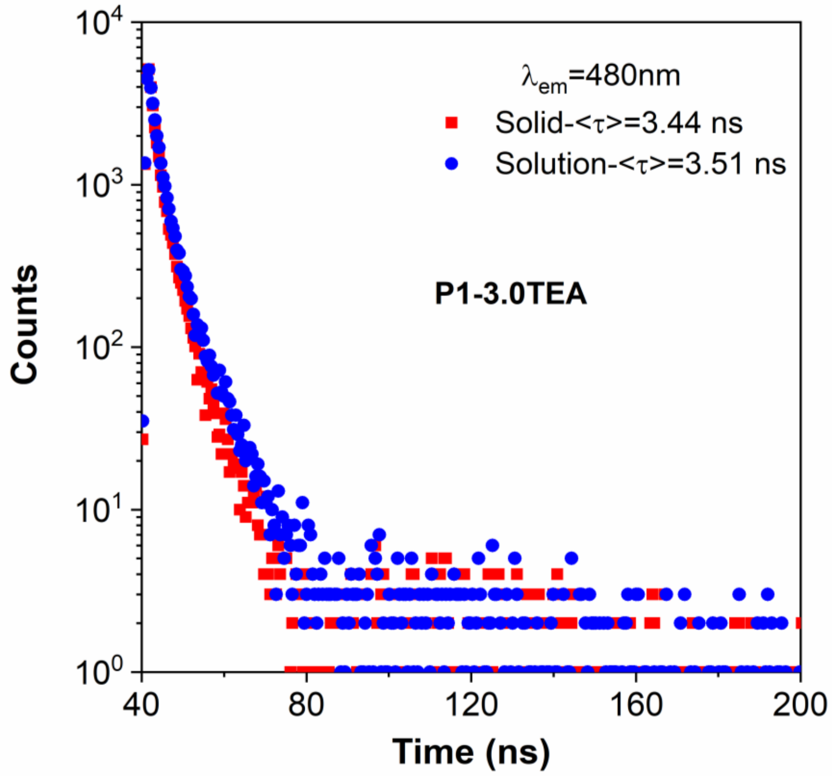

Supplementary Figure 105. Time-resolved PL decay curves of **P1-3.0TEA** measured at emission maximum of 480 nm in solid and DCM solution (c = 10^{-3} M), λ_{ex} = 365 nm.

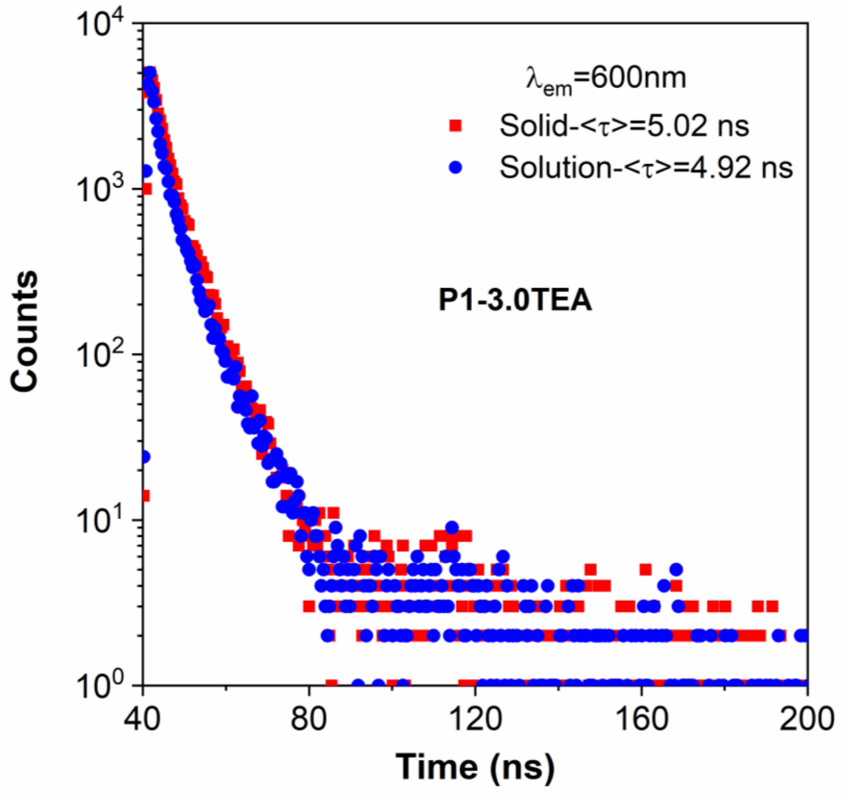

Supplementary Figure 106. Time-resolved PL decay curves of **P1-3.0TEA** measured at emission maximum of 600 nm in solid and DCM solution (c = 10^{-3} M), λ_{ex} = 365 nm.

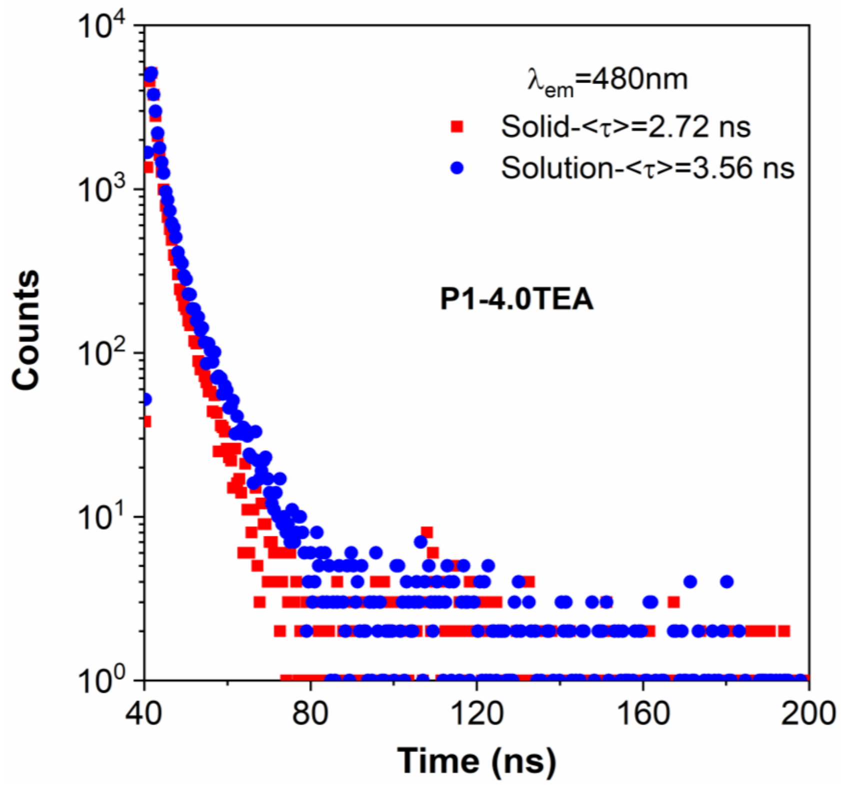

Supplementary Figure 107. Time-resolved PL decay curves of **P1-4.0TEA** measured at emission maximum of 480 nm in solid and DCM solution (c = 10^{-3} M), λ_{ex} = 365 nm.

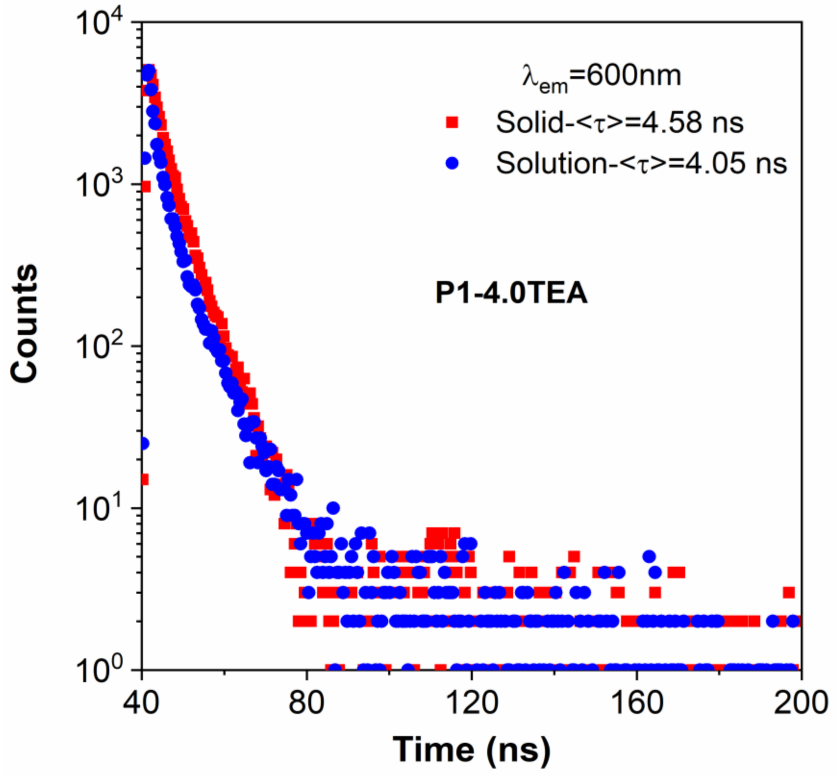

Supplementary Figure 108. Time-resolved PL decay curves of **P1-4.0TEA** measured at emission maximum of 600 nm in solid and DCM solution (c = 10^{-3} M), λ_{ex} = 365 nm.

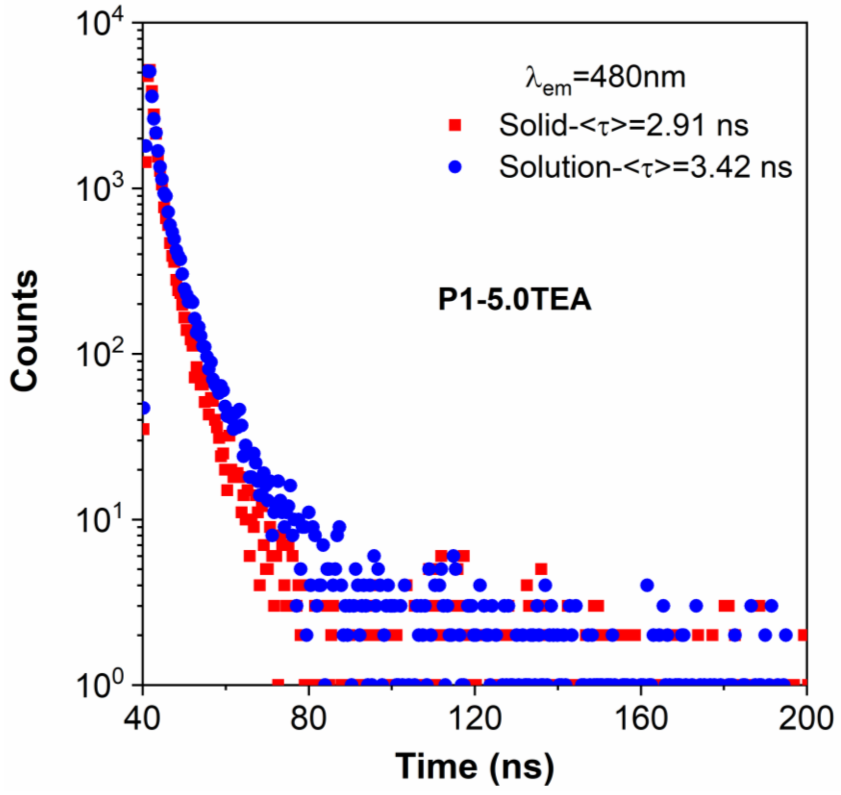

Supplementary Figure 109. Time-resolved PL decay curves of **P1-5.0TEA** measured at emission maximum of 480 nm in solid and DCM solution (c = 10^{-3} M), λ_{ex} = 365 nm.

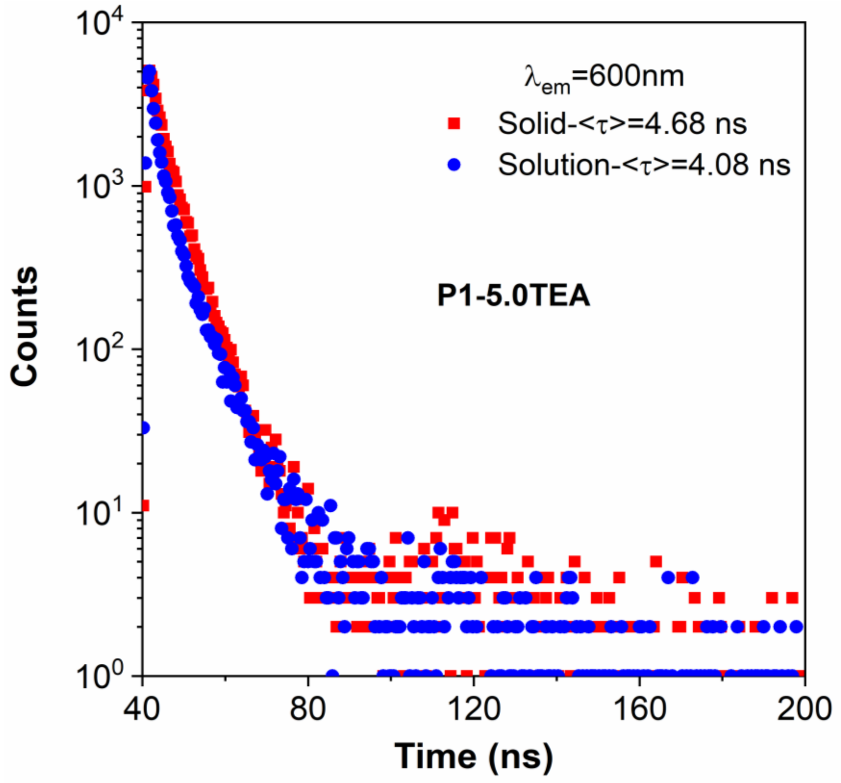

Supplementary Figure 110. Time-resolved PL decay curves of **P1-5.0TEA** measured at emission maximum of 600 nm in solid and DCM solution (c = 10^{-3} M), λ_{ex} = 365 nm.

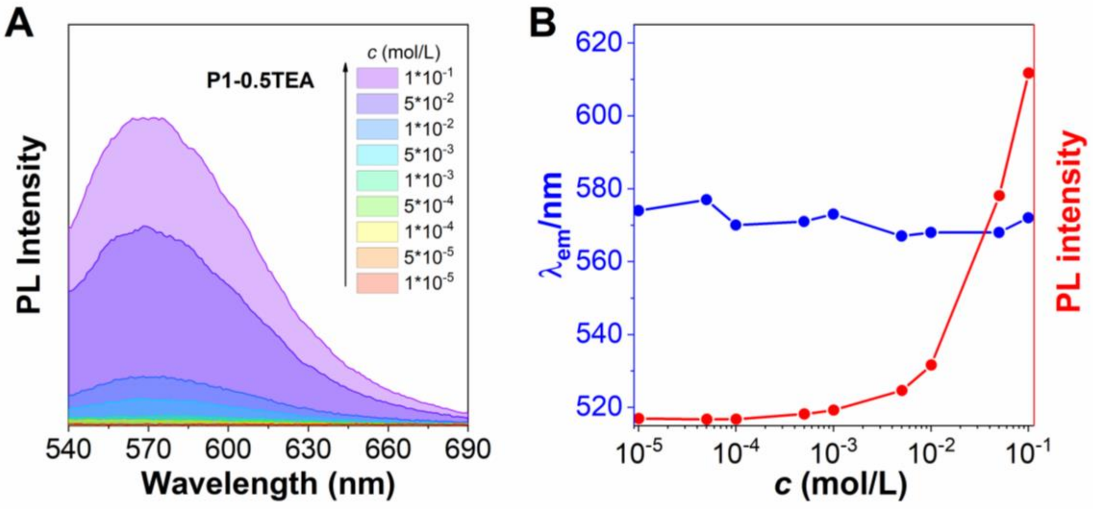

Supplementary Figure 111. (A) Concentration-dependent PL spectra of **P1-0.5TEA** in DCM solution, λ_{ex} = 520 nm.(B) Plots of emission wavelength and PL intensity versusversus concentration. Concentration: from 10^{-5} to 10^{-1} M.

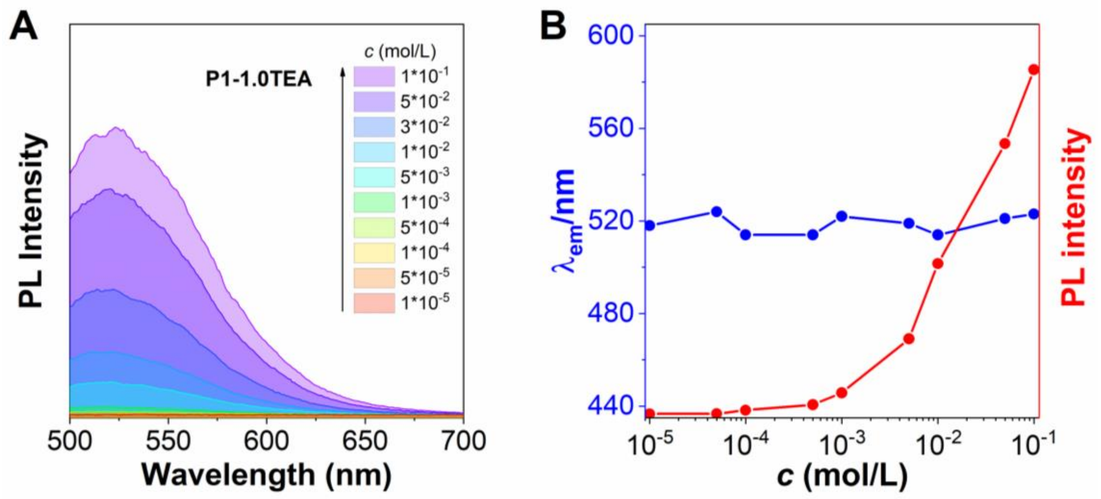

Supplementary Figure 112. (A) Concentration-dependent spectra of **P1-1.0TEA** in DCM solution, λ_{ex} = 480 nm. (B) Plots of emission wavelength and PL intensity versus concentration.concentration Concentration: from 10^{-5} to 10^{-1} M.

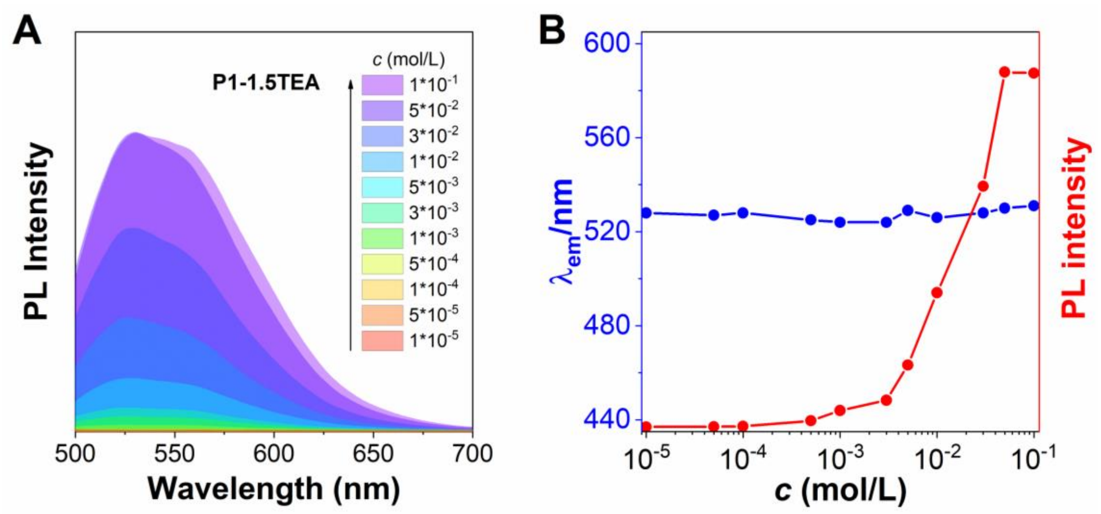

Supplementary Figure 113. (A) Concentration-dependent spectra of **P1-1.5TEA** in DCM solution, λ_{ex} = 480 nm. (B) Plots of emission wavelength and PL intensity and emission wavelength plots versus concentration.concentration Concentration: from 10^{-5} to 10^{-1} M.

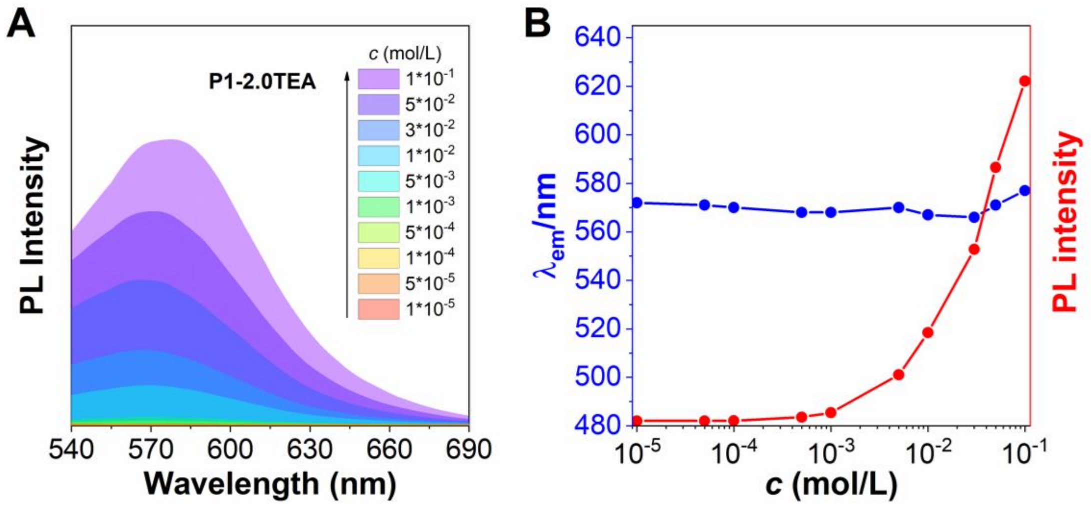

Supplementary Figure 114. (A) Concentration-dependent spectra of **P1-2.0TEA** in DCM solution, λ_{ex} = 520 nm. (B) Plots of emission wavelength and PL intensity and emission wavelength plots versus concentration.concentration Concentration: from 10^{-5} to 10^{-1} M.

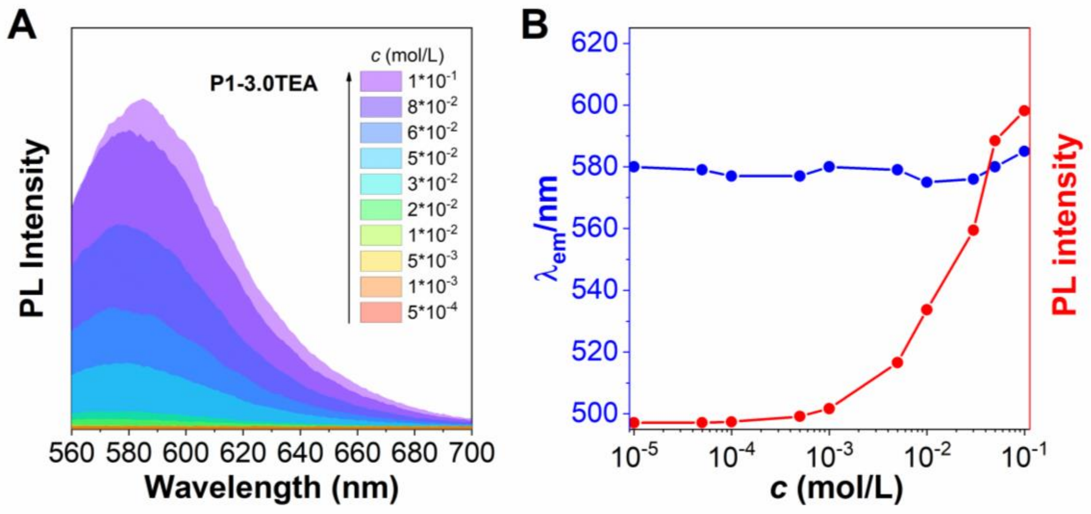

Supplementary Figure 115. (A) Concentration-dependent spectra of **P1-3.0TEA** in DCM solution, λ_{ex} = 540 nm. (B) Plots of emission wavelength and PL intensity and emission wavelength plots versus concentration.concentration Concentration: from 10^{-5} to 10^{-1} M.

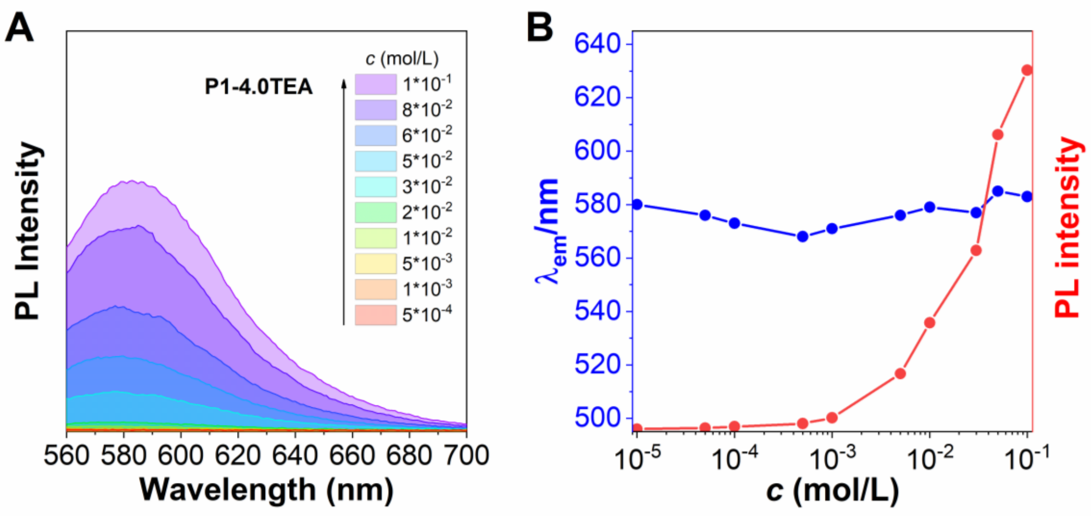

Supplementary Figure 116. (A) Concentration-dependent spectra of **P1-4.0TEA** in DCM solution, λ_{ex} = 540 nm. (B) Plots of emission wavelength and PL intensity and emission wavelength plots versus concentration.concentration Concentration: from 10^{-5} to 10^{-1} M.

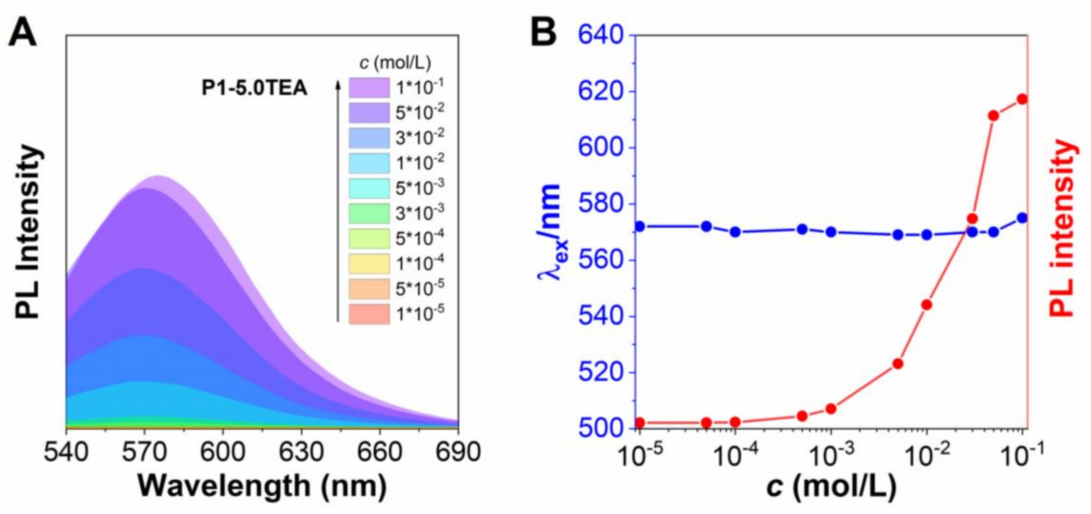

Supplementary Figure 117. (A) Concentration-dependent spectra of **P1-5.0TEA** in DCM solution, λ_{ex} = 520 nm. (B) Plots of emission wavelength and PL intensity and emission wavelength plots versus concentration.concentration Concentration: from 10^{-5} to 10^{-1} M.

Photophysical characterization of P2-aTEA (a% is from 0.5% to 5.0%)

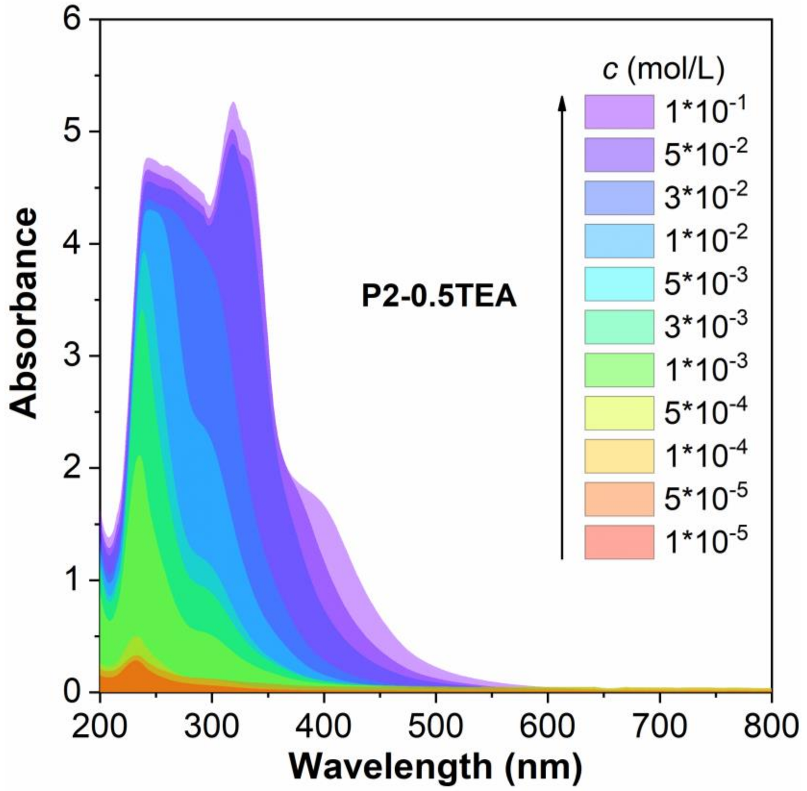

Supplementary Figure 118. Concentration-dependent UV-Vis absorption spectra of **P2-0.5TEA** in DCM solution. Concentration: from 10^{-5} to 10^{-1} M.

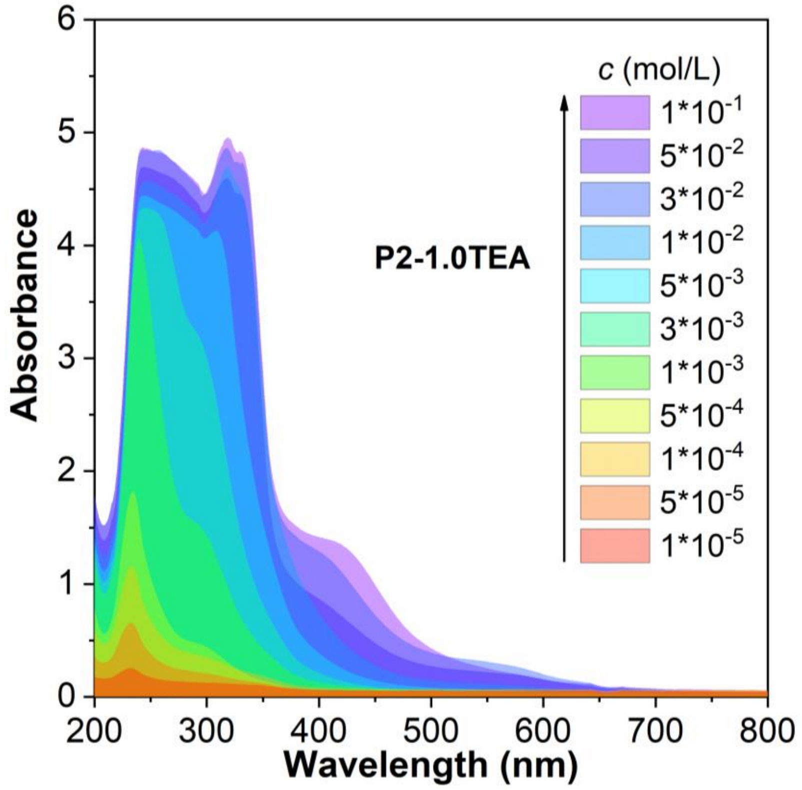

Supplementary Figure 119. Concentration-dependent UV-Vis absorption spectra of **P2-1.0TEA in DCM** solution. Concentration: from 10^{-5} to 10^{-1} M.

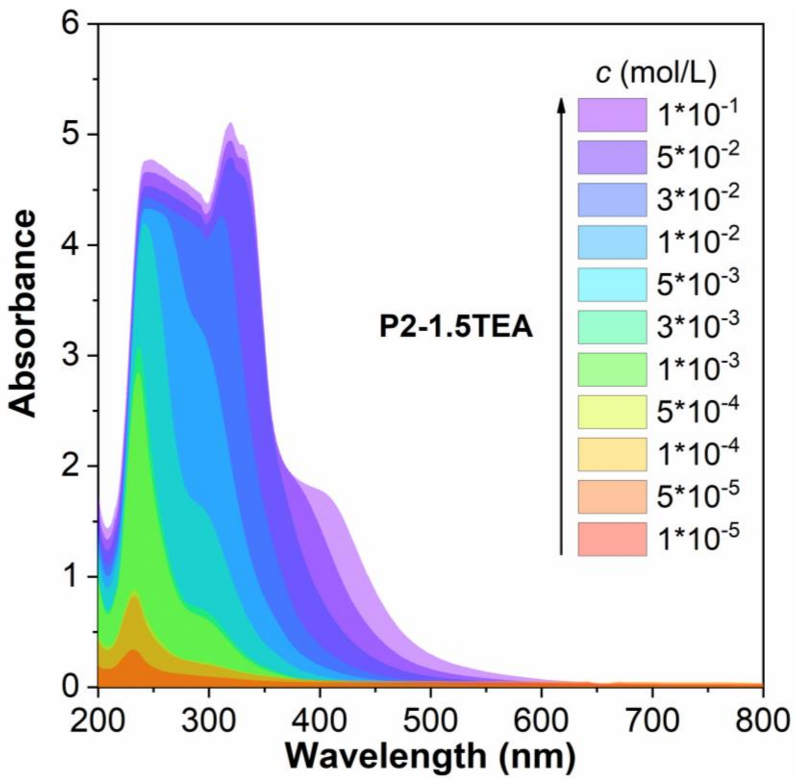

Supplementary Figure 120. Concentration-dependent UV-Vis absorption spectra of **P2-1.5TEA in DCM** solution. Concentration: from 10^{-5} to 10^{-1} M.

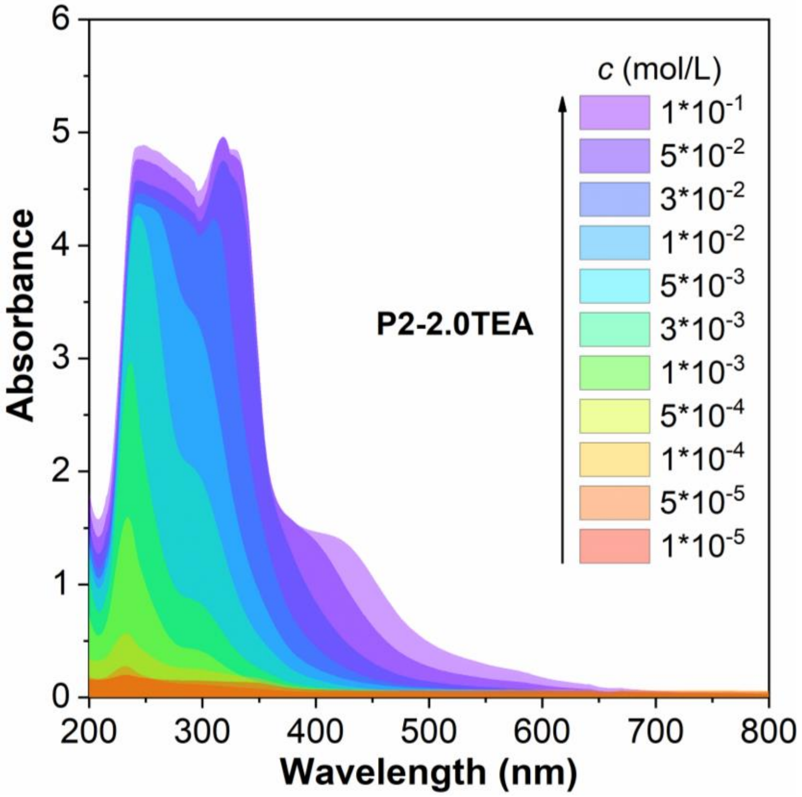

Supplementary Figure 121. Concentration-dependent UV-Vis absorption spectra of **P2-2.0TEA in DCM** solution. Concentration: from 10^{-5} to 10^{-1} M.

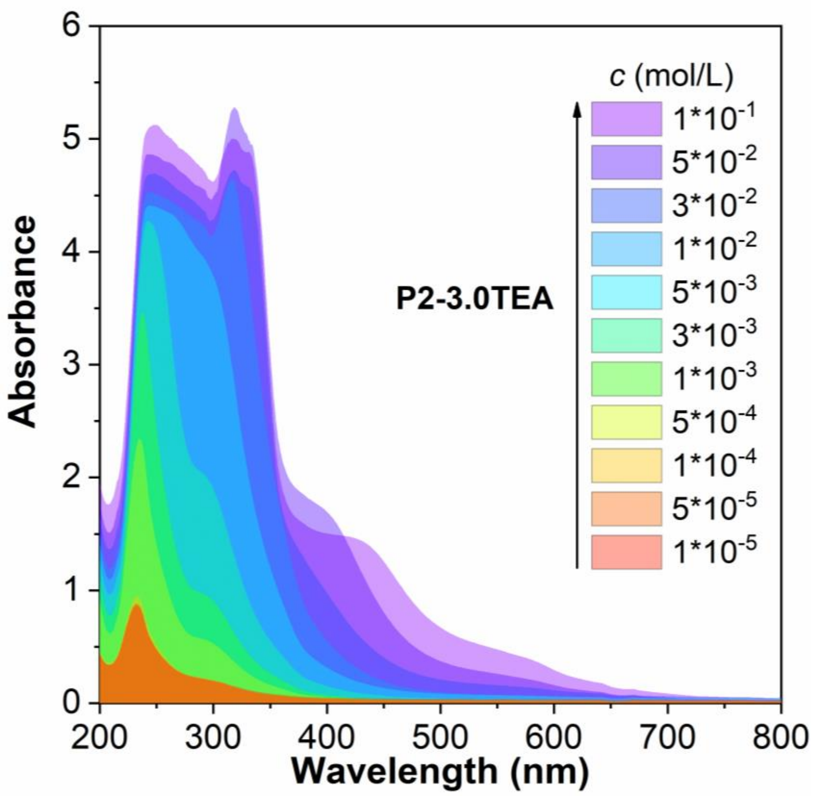

Supplementary Figure 122. Concentration-dependent UV-Vis absorption spectra of **P2-3.0TEA in DCM** solution. Concentration: from 10^{-5} to 10^{-1} M.

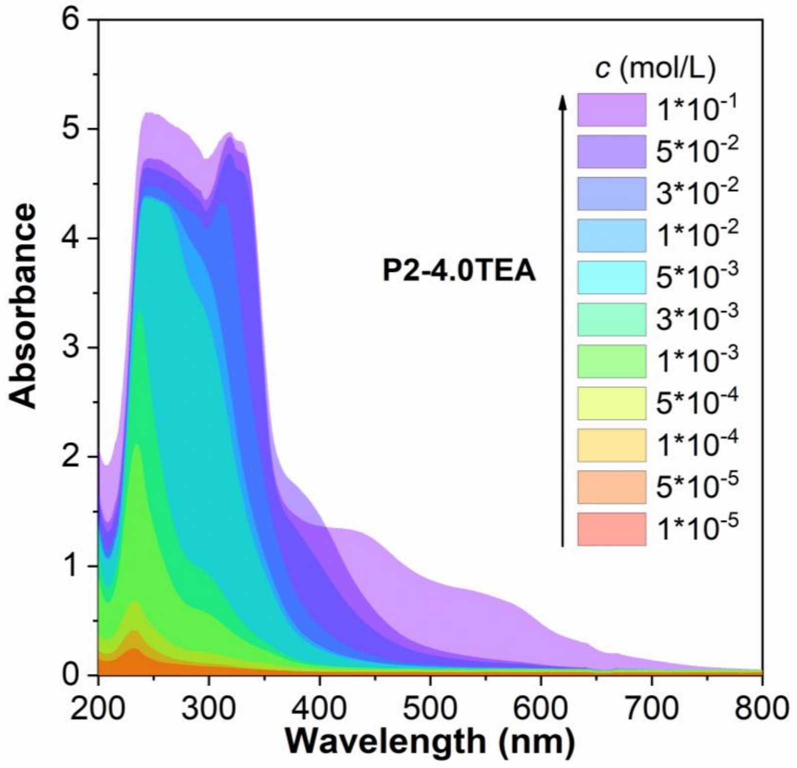

Supplementary Figure 123. Concentration-dependent UV-Vis absorption spectra of **P2-4.0TEA in DCM** solution. Concentration: from 10^{-5} to 10^{-1} M.

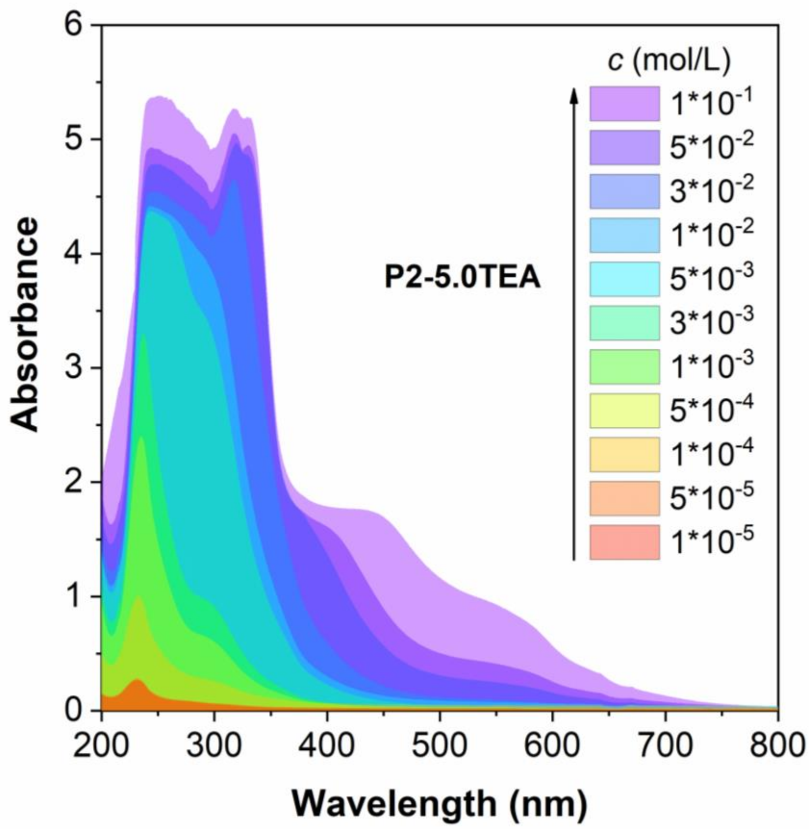

Supplementary Figure 124. Concentration-dependent UV-Vis absorption spectra of **P2-5.0TEA in DCM** solution. Concentration: from 10^{-5} to 10^{-1} M.

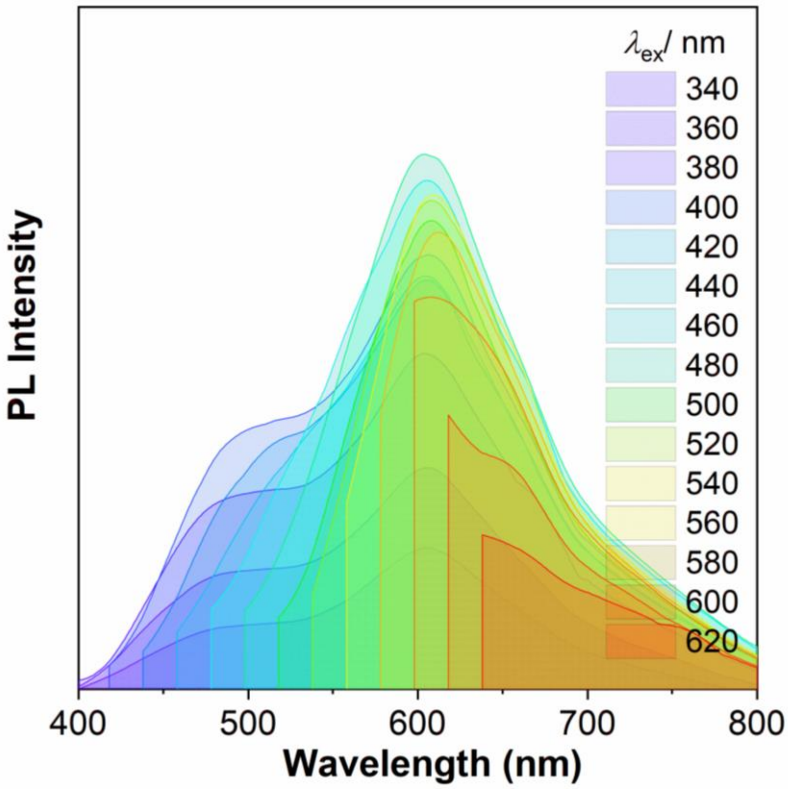

Supplementary Figure 125. PL spectra of **P2-0.5TEA** in solid under different excitation wavelengths.

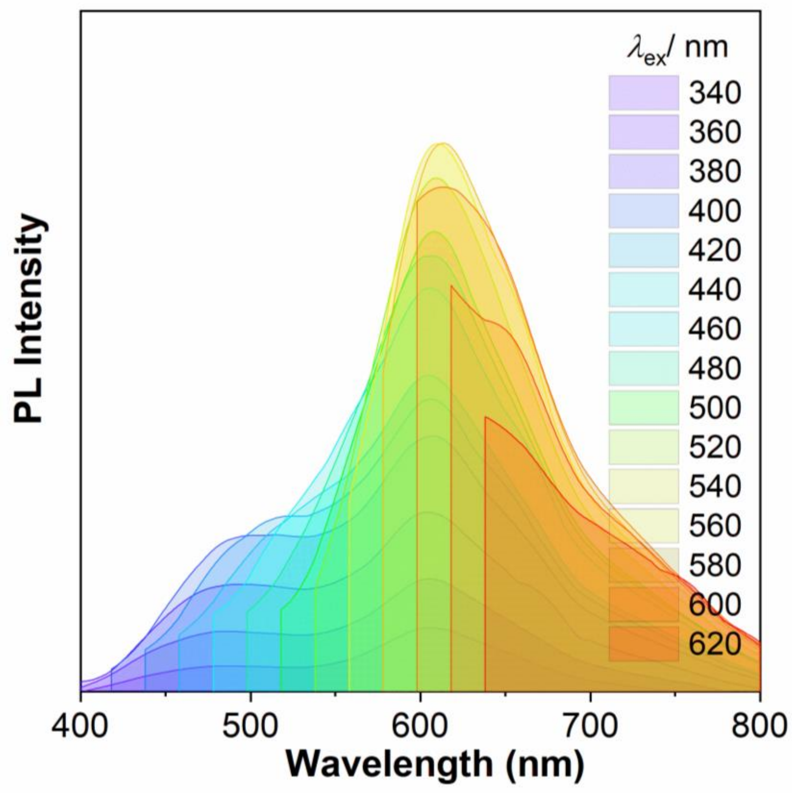

Supplementary Figure 126. PL spectra of **P2-1.0TEA** in solid under different excitation wavelengths.

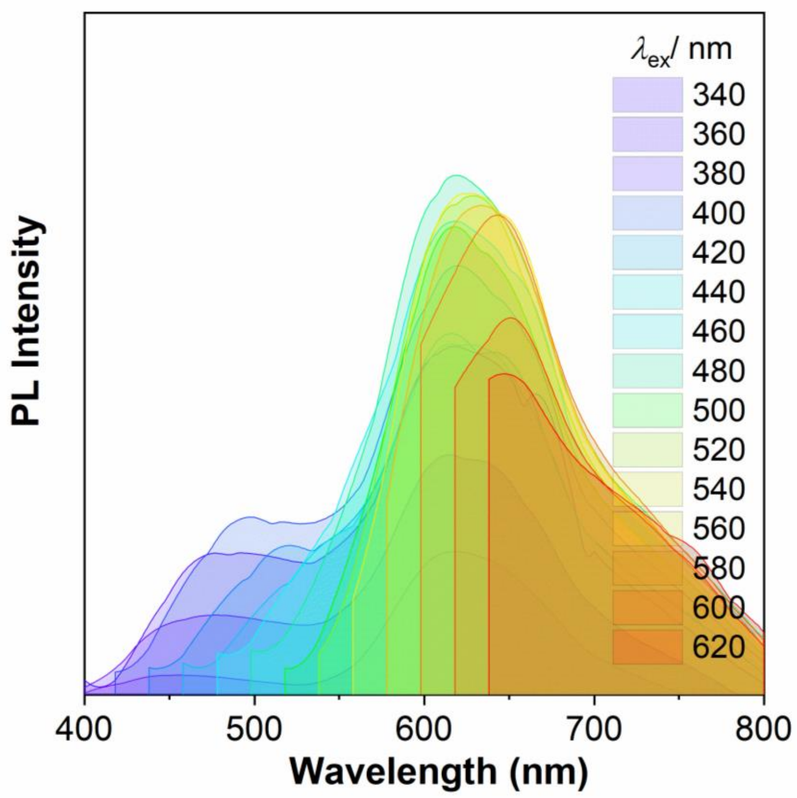

Supplementary Figure 127. PL spectra of **P2-1.5TEA** in solid under different excitation wavelengths.

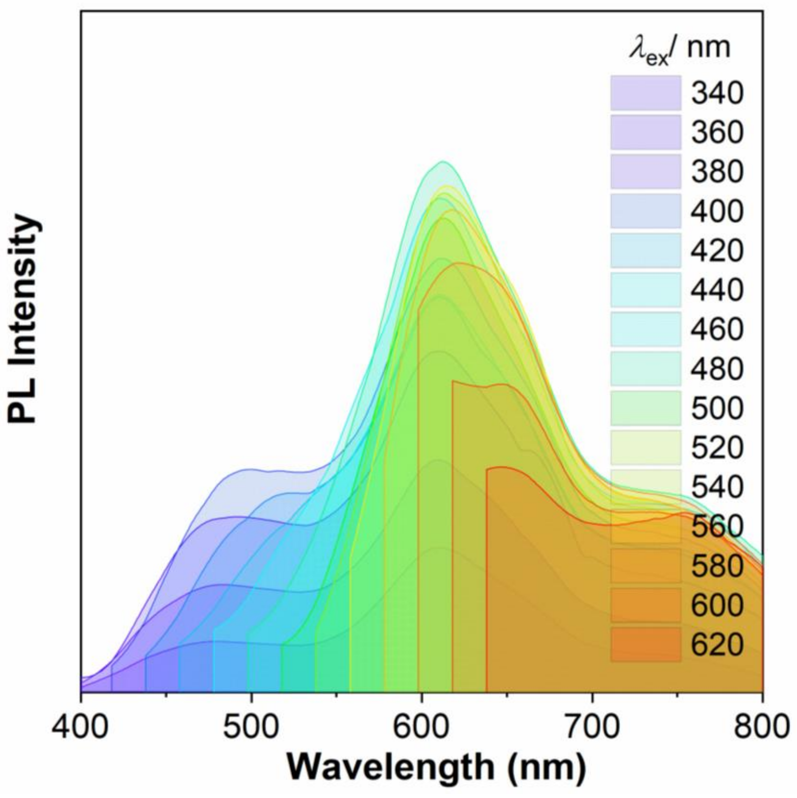

Supplementary Figure 128. PL spectra of **P2-2.0TEA** in solid under different excitation wavelengths.

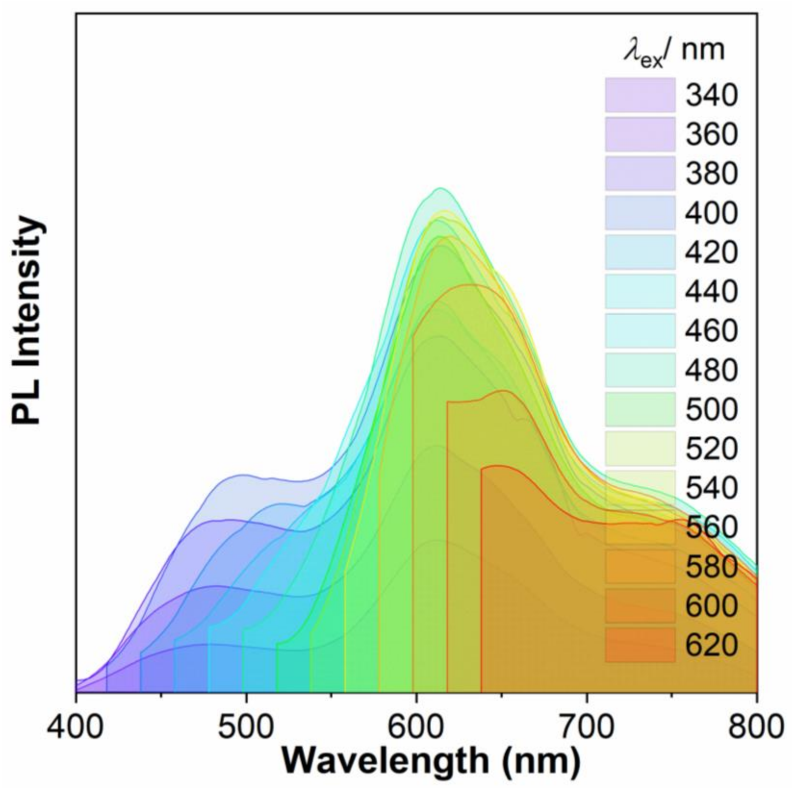

Supplementary Figure 129. PL spectra of **P2-3.0TEA** in solid under different excitation wavelengths.

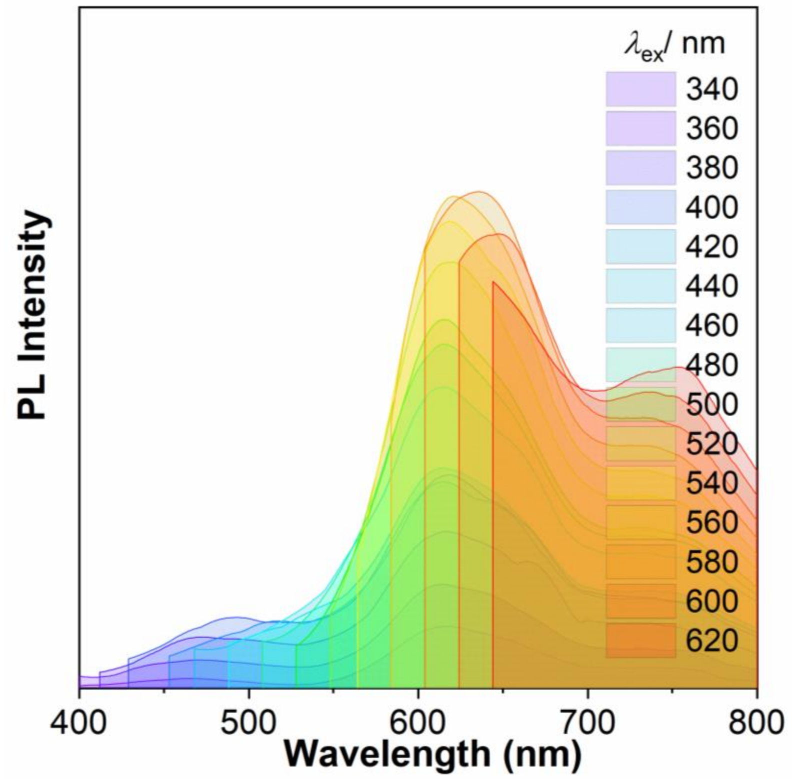

Supplementary Figure 130. PL spectra of **P2-4.0TEA** in solid under different excitation wavelengths.

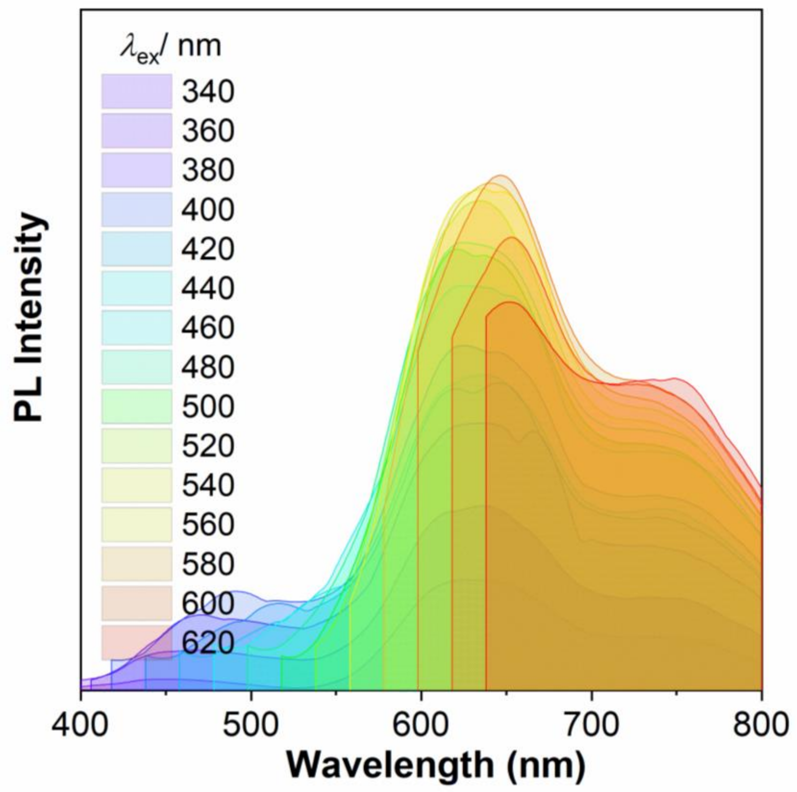

Supplementary Figure 131. PL spectra of **P2-5.0TEA** in solid under different excitation wavelengths.

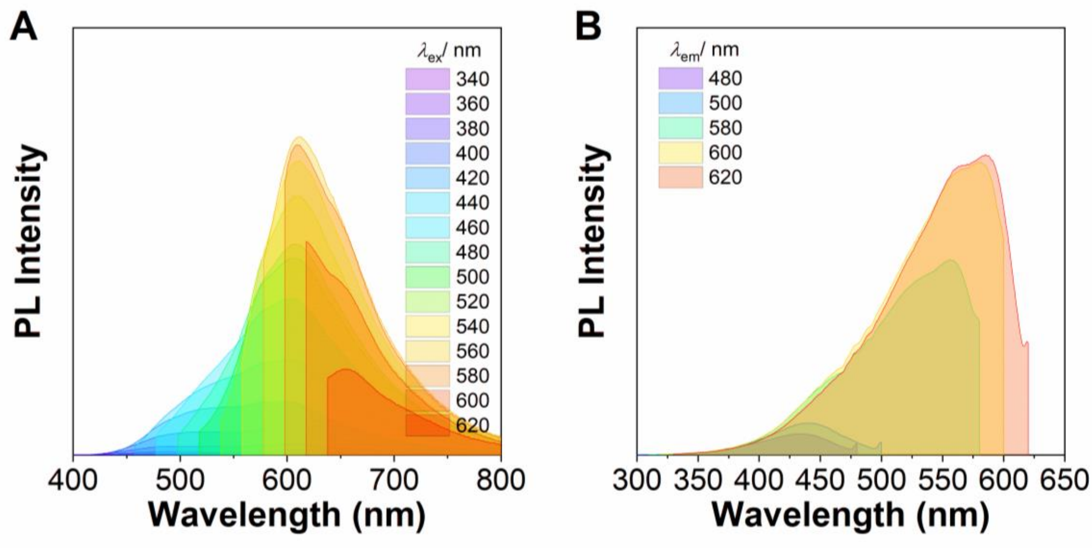

Supplementary Figure 132. PL spectra (A) under different excitation wavelengths and excitation spectra (B) under different emission wavelengths of **P2-0.5TEA** in DCM solution of 10^{-1} M.

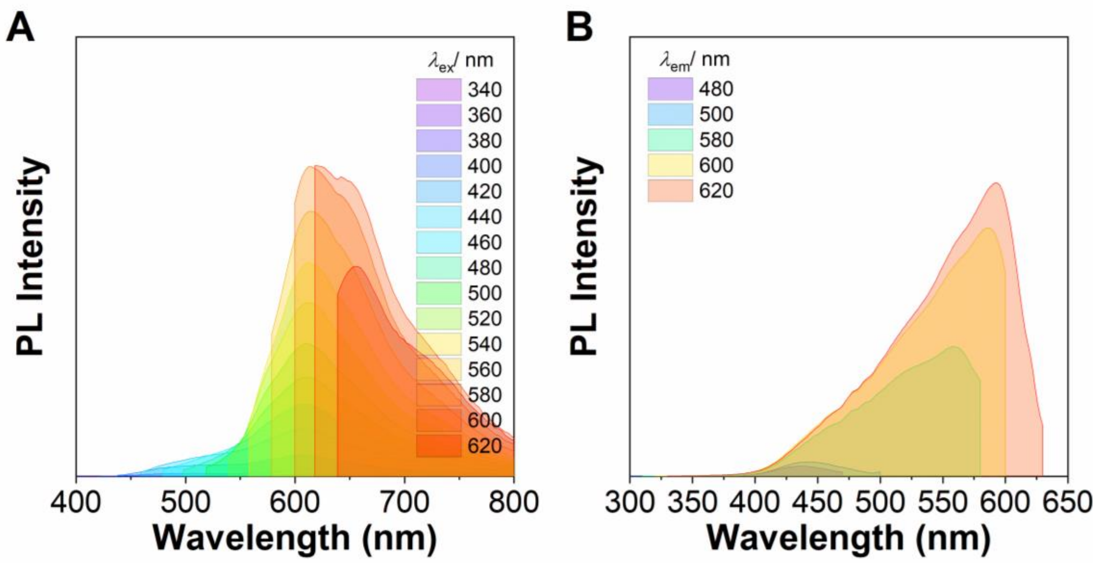

Supplementary Figure 133. PL spectra (A) under different excitation wavelengths and excitation spectra (B) under different emission wavelengths of **P2-1.0TEA** in DCM solution of 10^{-1} M.

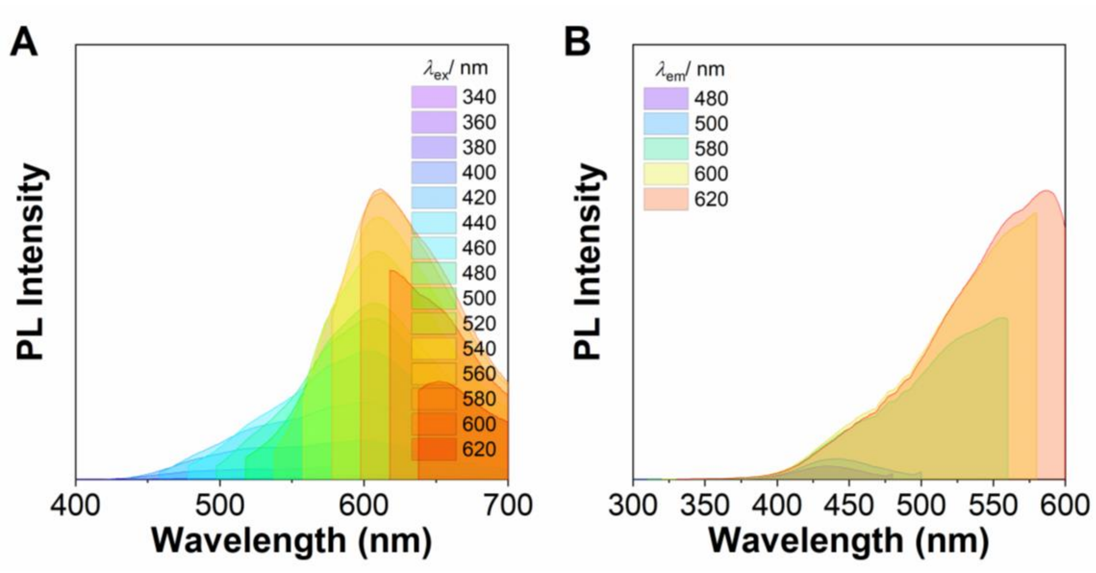

Supplementary Figure 134. PL spectra (A) under different excitation wavelengths and excitation spectra (B) under different emission wavelengths of **P2-1.5TEA** in DCM solution of 10^{-1} M.

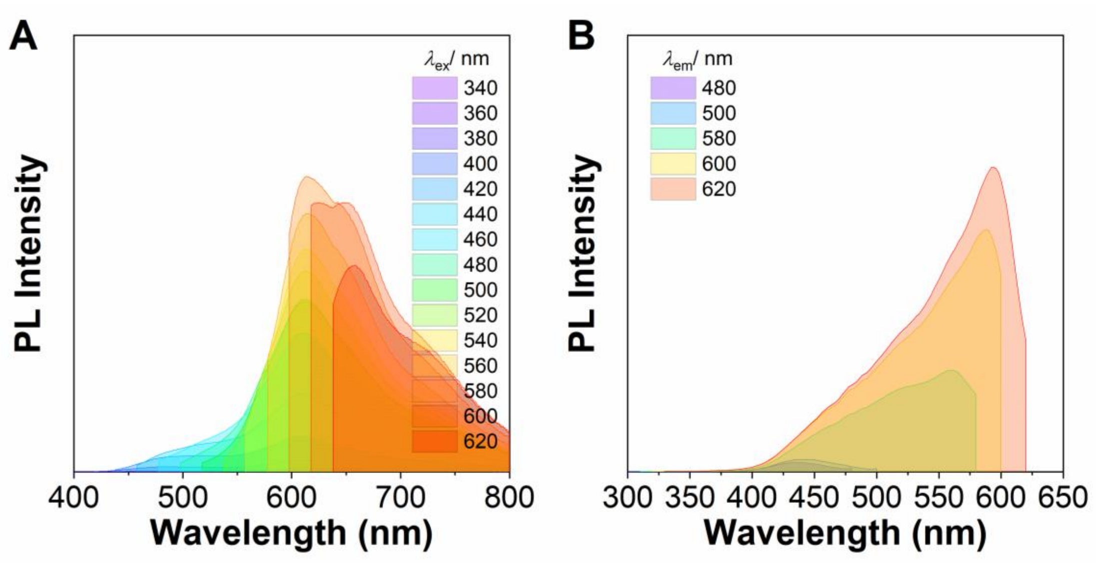

Supplementary Figure 135. PL spectra (A) under different excitation wavelengths and excitation spectra (B) under different emission wavelengths of **P2-2.0TEA** in DCM solution of 10^{-1} M.

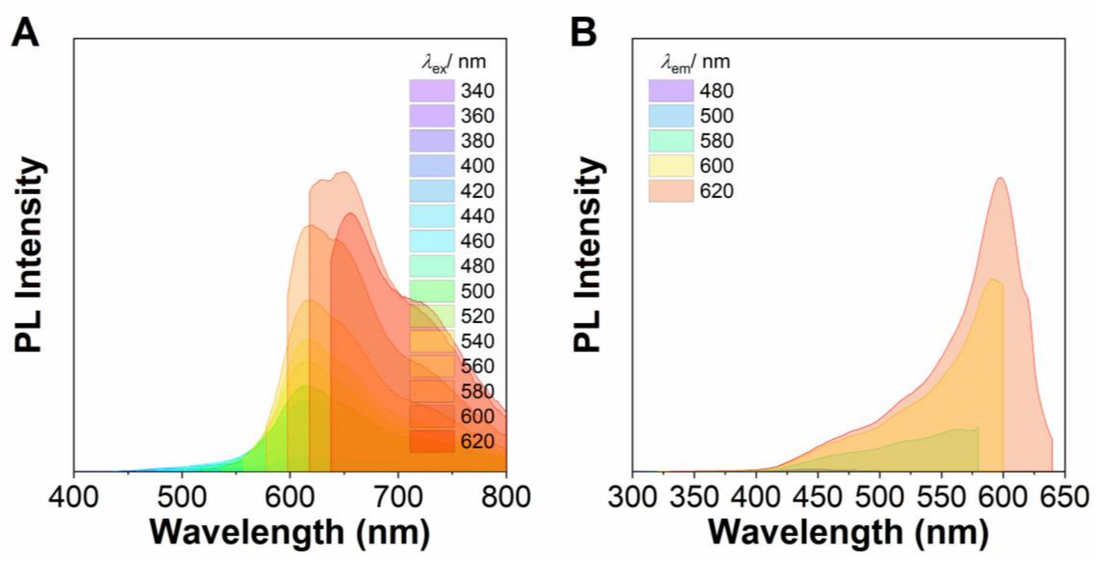

Supplementary Figure 136. PL spectra (A) under different excitation wavelengths and excitation spectra (B) under different emission wavelengths of **P2-3.0TEA** in DCM solution of 10^{-1} M.

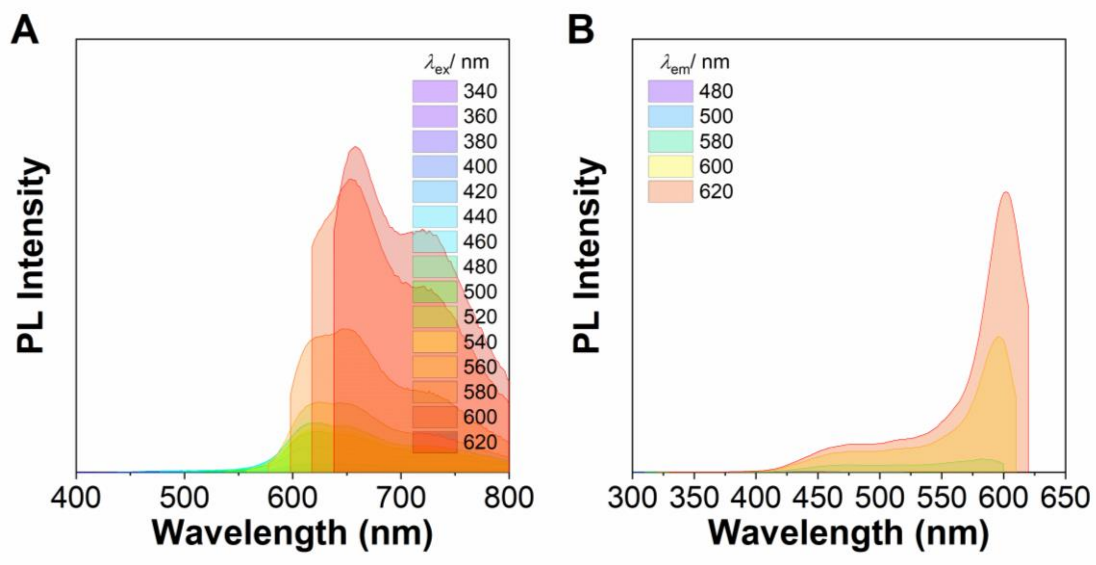

Supplementary Figure 137. PL spectra (A) under different excitation wavelengths and excitation spectra (B) under different emission wavelengths of **P2-4.0TEA** in DCM solution of 10^{-1} M.

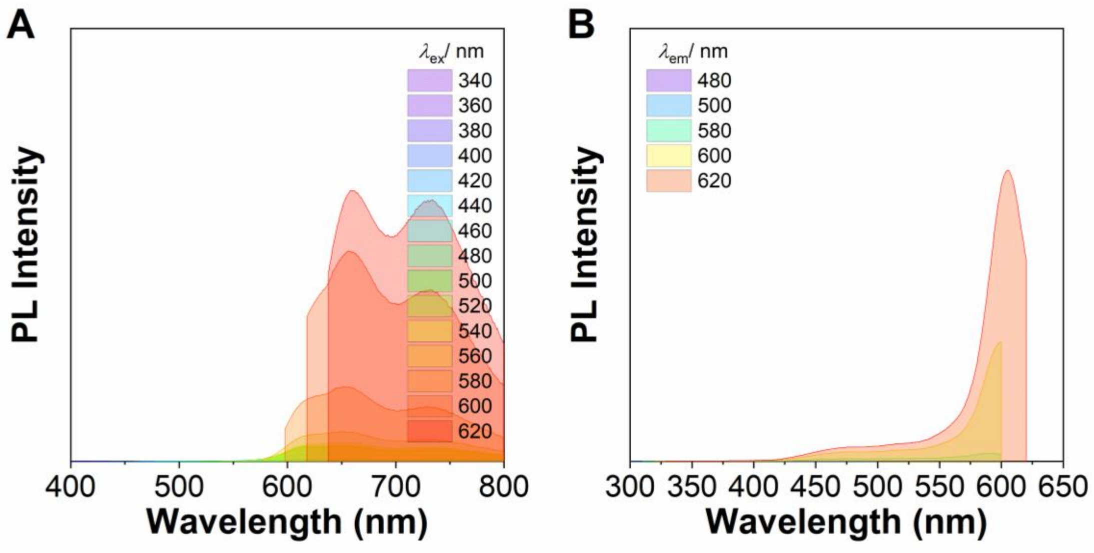

Supplementary Figure 138. PL spectra (A) under different excitation wavelengths and excitation spectra (B) under different emission wavelengths of **P2-5.0TEA** in DCM solution of 10^{-1} M.

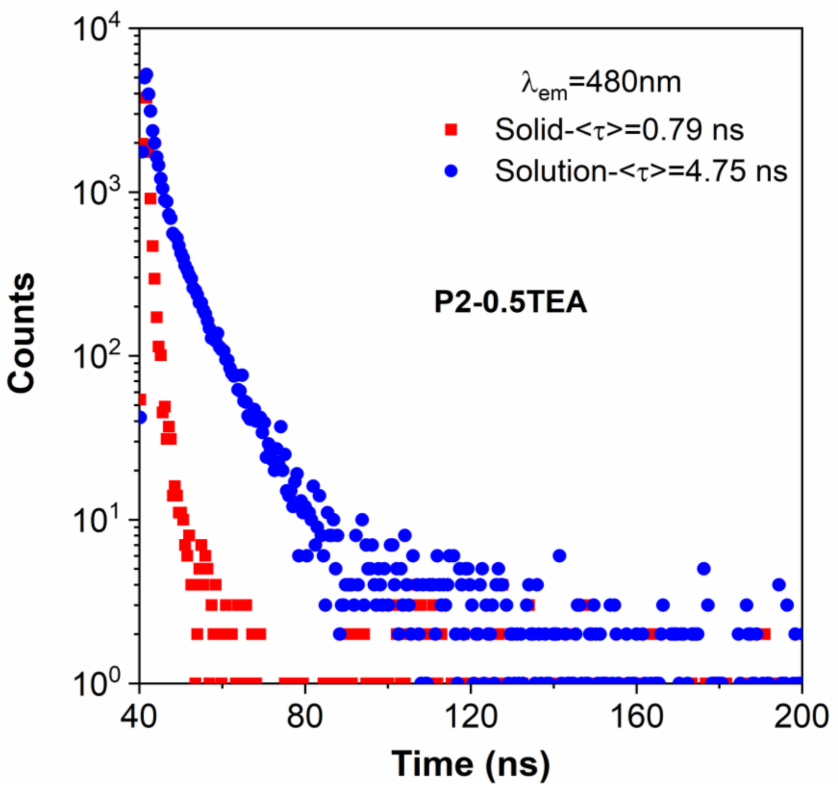

Supplementary Figure 139. Time-resolved PL decay curves of **P2-0.5TEA** measured at emission maximum of 480 nm in solid and DCM solution (c = 10^{-3} M), λ_{ex} = 365 nm.

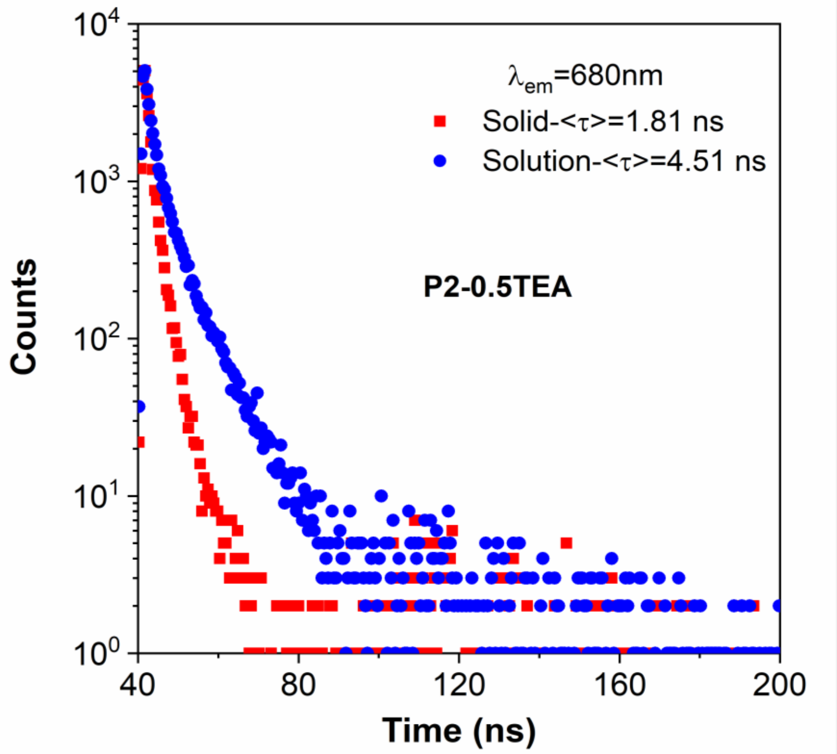

Supplementary Figure 140. Time-resolved PL decay curves of **P2-0.5TEA** measured at emission maximum of 680 nm in solid and DCM solution (c = 10^{-3} M), λ_{ex} = 470 nm.

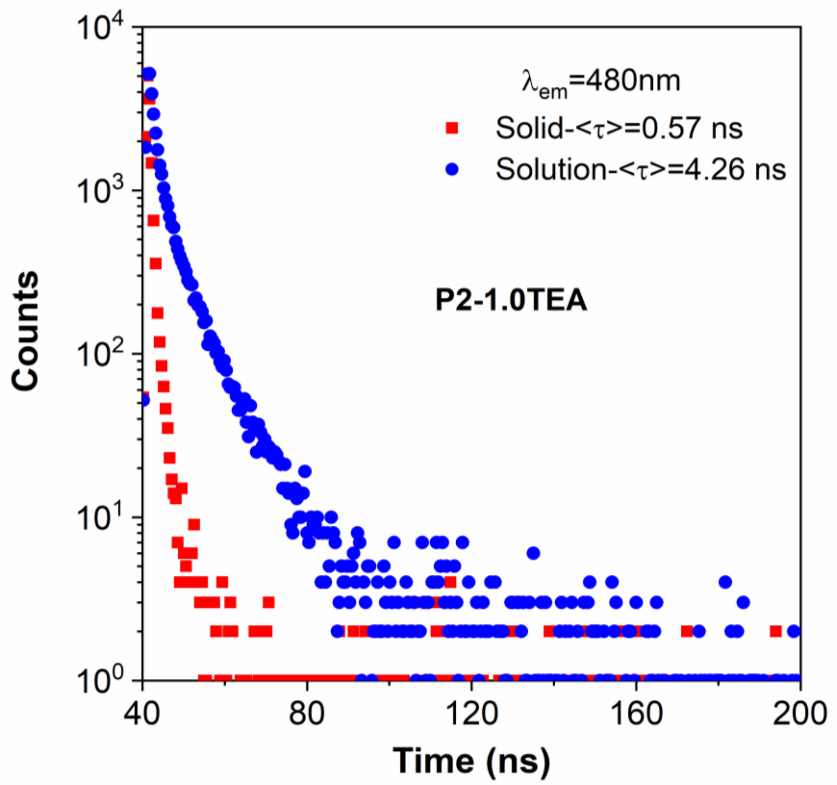

Supplementary Figure 141. Time-resolved PL decay curves of **P2-1.0TEA** measured at emission maximum of 480 nm in solid and DCM solution (c = 10^{-3} M), λ_{ex} = 365 nm.

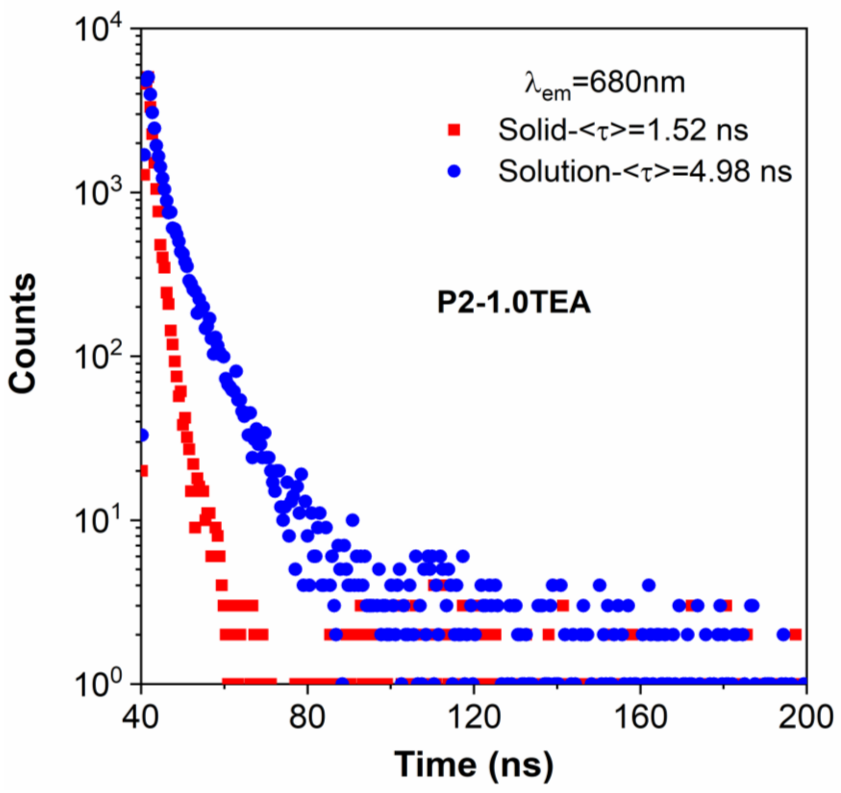

Supplementary Figure 142. Time-resolved PL decay curves of **P2-1.0TEA** measured at emission maximum of 680 nm in solid and DCM solution (c = 10^{-3} M), λ_{ex} = 470 nm.

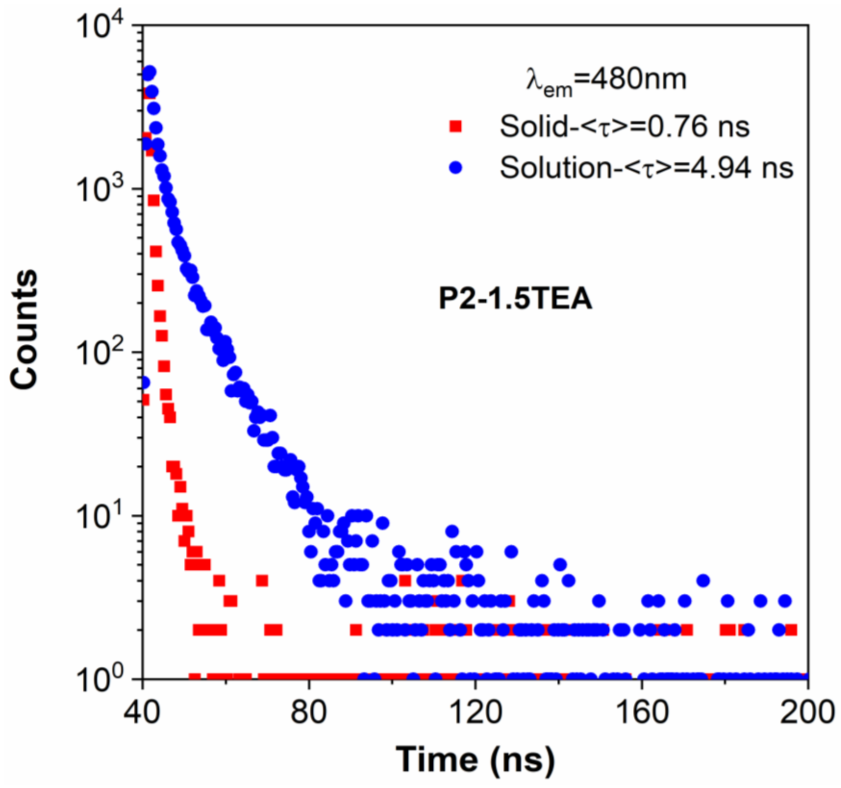

Supplementary Figure 143. Time-resolved PL decay curves of **P2-1.5TEA** measured at emission maximum of 480 nm in solid and DCM solution (c = 10^{-3} M), λ_{ex} = 365 nm.

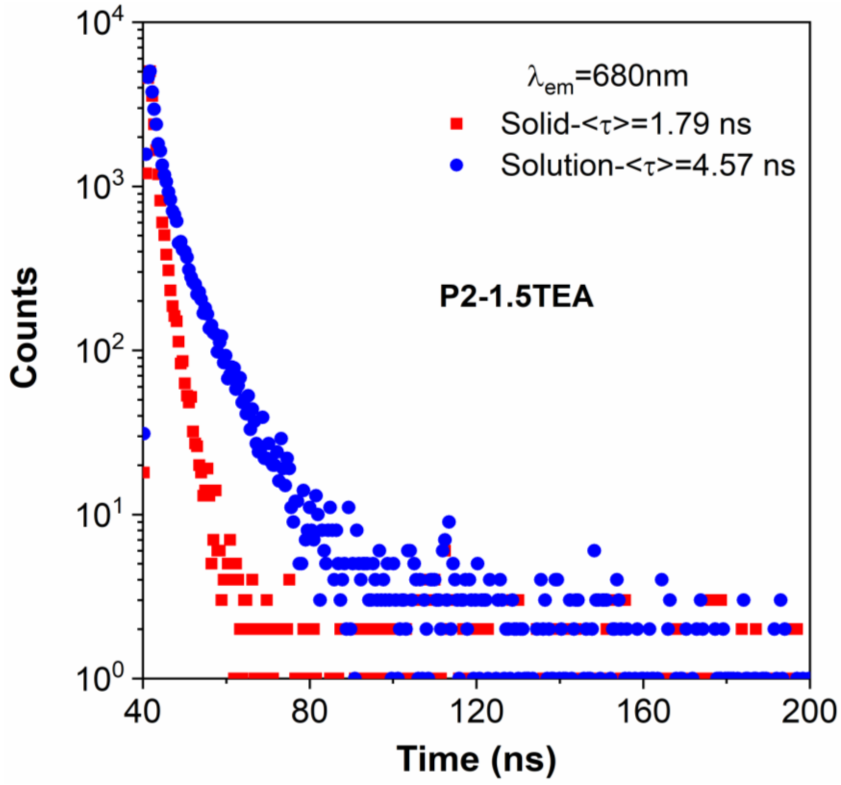

Supplementary Figure 144. Time-resolved PL decay curves of **P2-1.5TEA** measured at emission maximum of 680 nm in solid and DCM solution (c = 10^{-3} M), λ_{ex} = 470 nm.

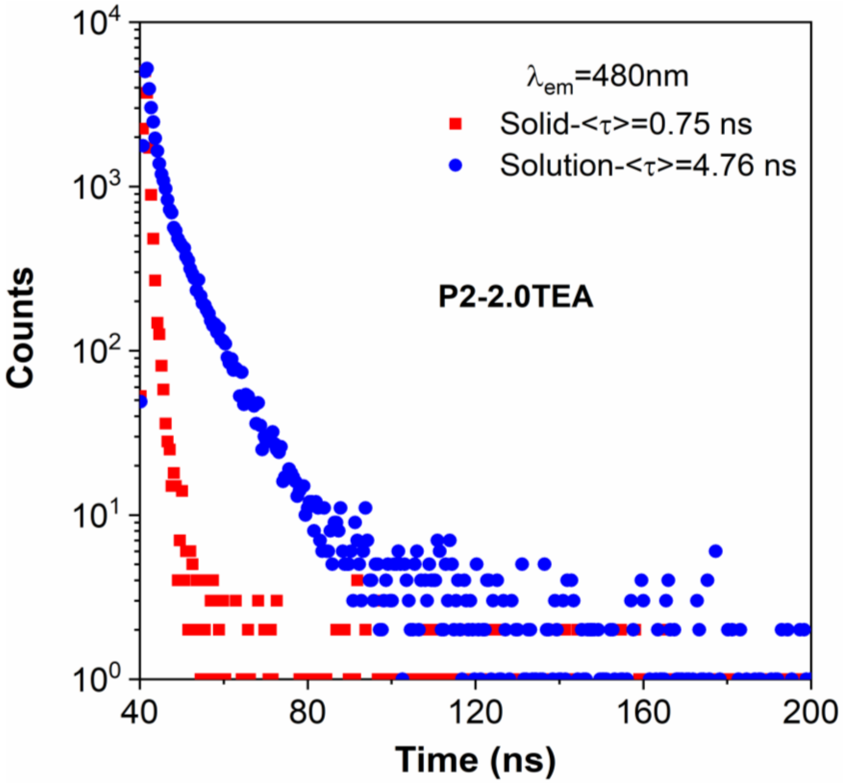

Supplementary Figure 145. Time-resolved PL decay curves of **P2-2.0TEA** measured at emission maximum of 480 nm in solid and DCM solution (c = 10^{-3} M), λ_{ex} = 365 nm.

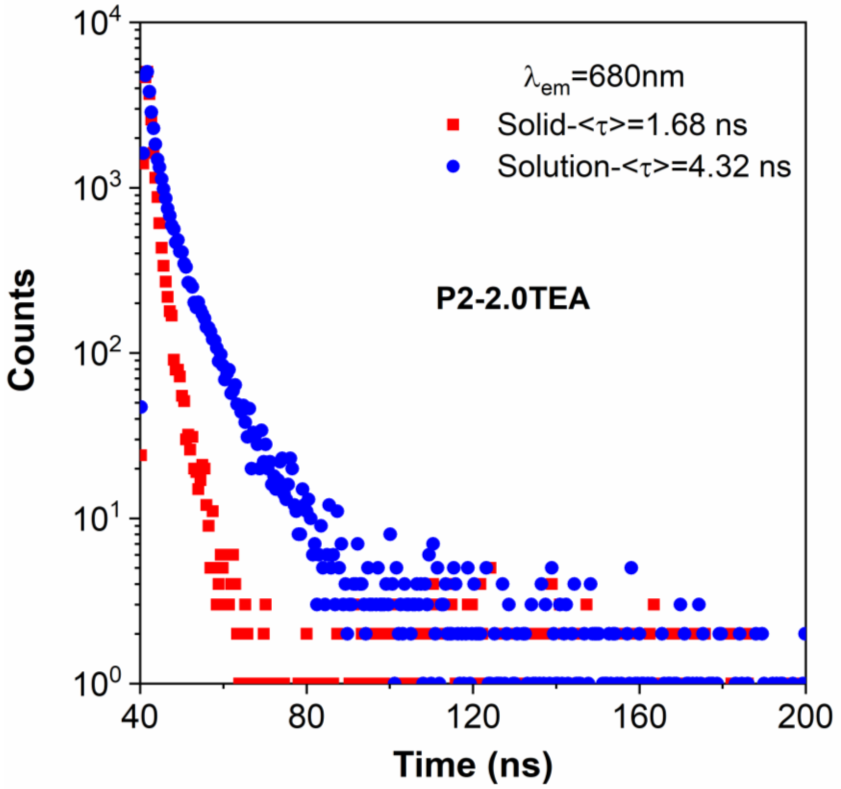

Supplementary Figure 146. Time-resolved PL decay curves of **P2-2.0TEA** measured at emission maximum of 680 nm in solid and DCM solution (c = 10^{-3} M), λ_{ex} = 470 nm.

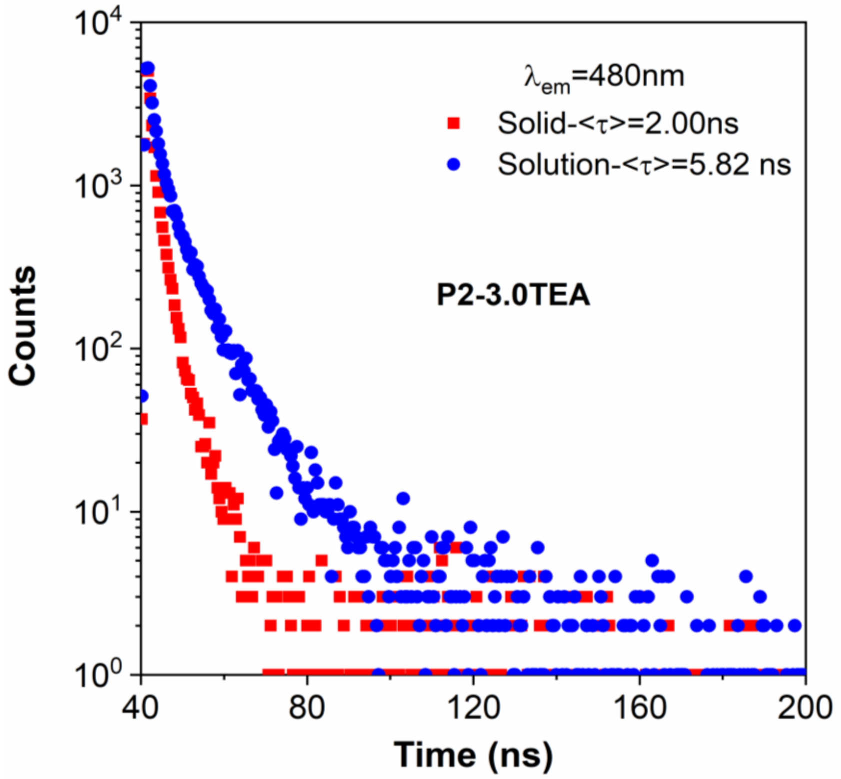

Supplementary Figure 147. Time-resolved PL decay curves of **P2-3.0TEA** measured at emission maximum of 480 nm in solid and DCM solution (c = 10^{-3} M), λ_{ex} = 365 nm.

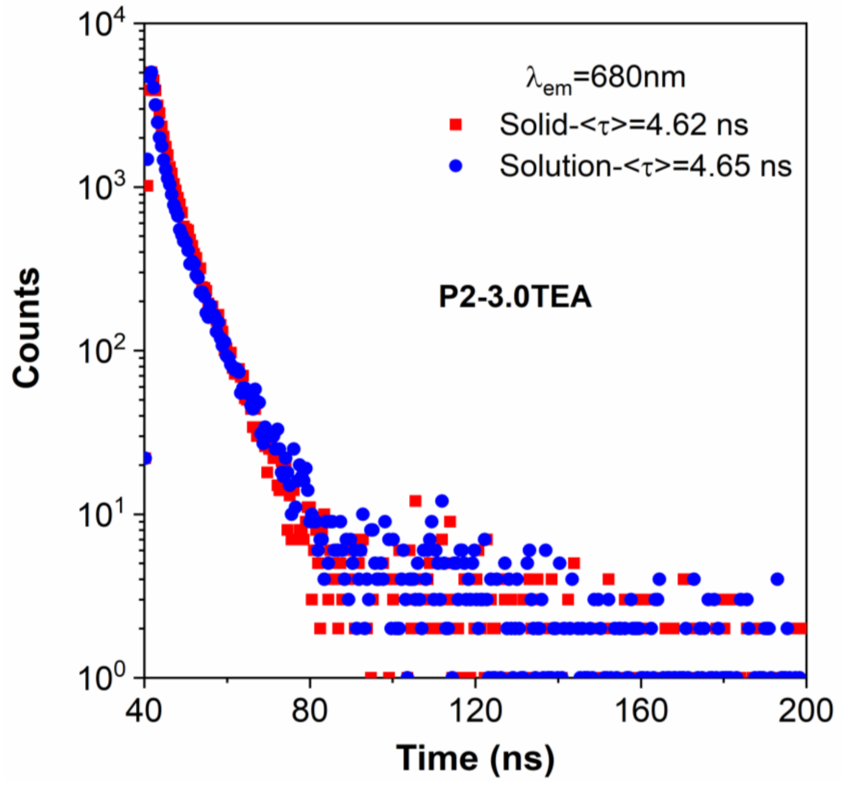

Supplementary Figure 148. Time-resolved PL decay curves of **P2-3.0TEA** measured at emission maximum of 680 nm in solid and DCM solution (c = 10^{-3} M), λ_{ex} = 470 nm.

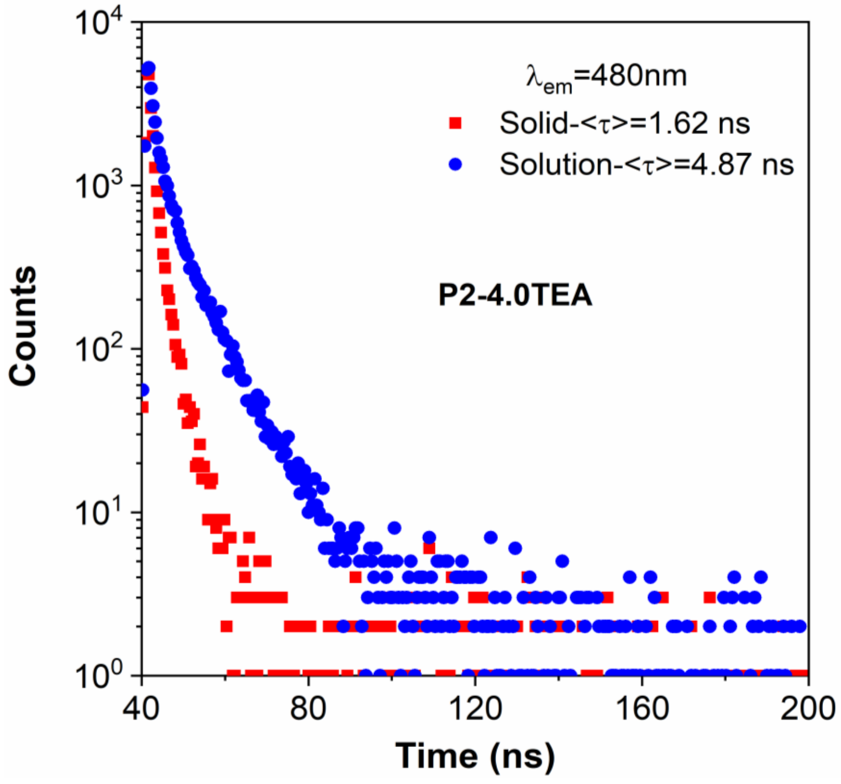

Supplementary Figure 149. Time-resolved PL decay curves of **P2-4.0TEA** measured at emission maximum of 480 nm in solid and DCM solution (c = 10^{-3} M), λ_{ex} = 365 nm.

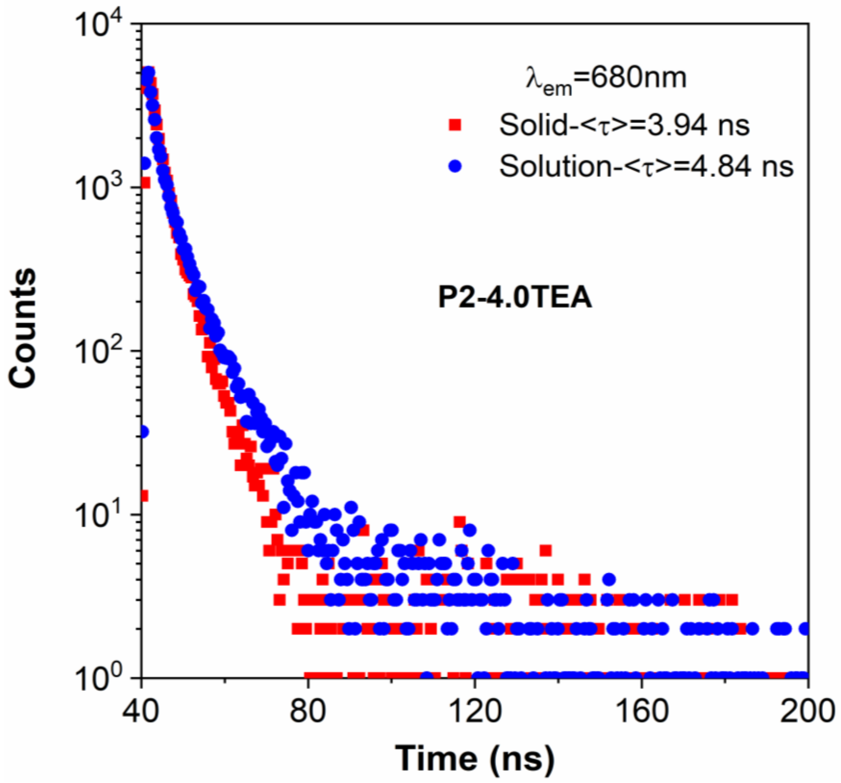

Supplementary Figure 150. Time-resolved PL decay curves of **P2-4.0TEA** measured at emission maximum of 680 nm in solid and DCM solution (c = 10^{-3} M), λ_{ex} = 470 nm.

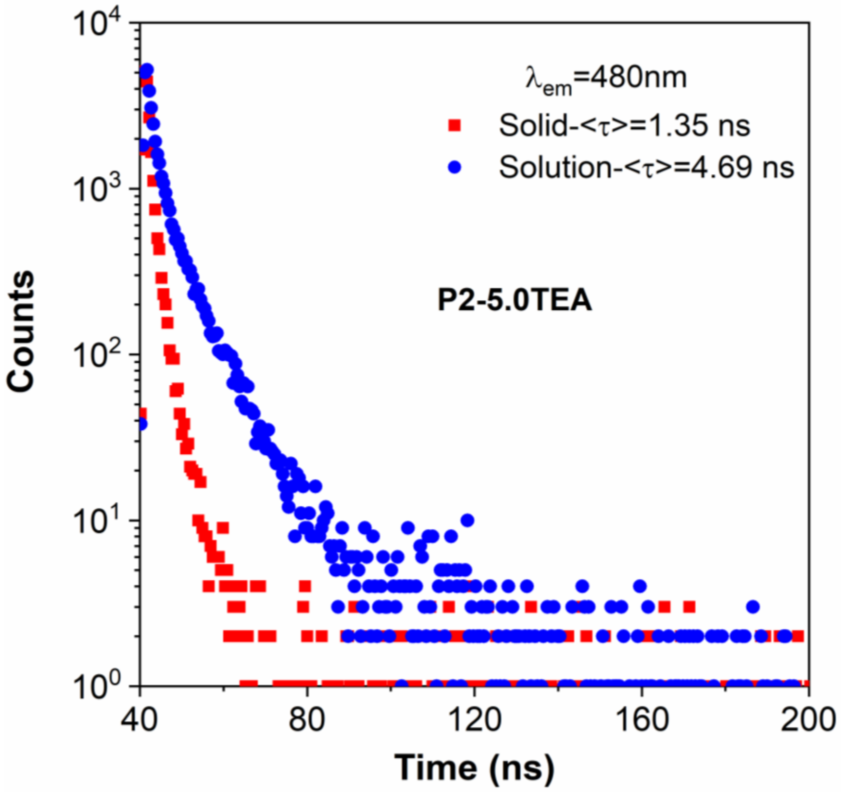

Supplementary Figure 151. Time-resolved PL decay curves of **P2-5.0TEA** measured at emission maximum of 480 nm in solid and DCM solution (c = 10^{-3} M), λ_{ex} = 365 nm.

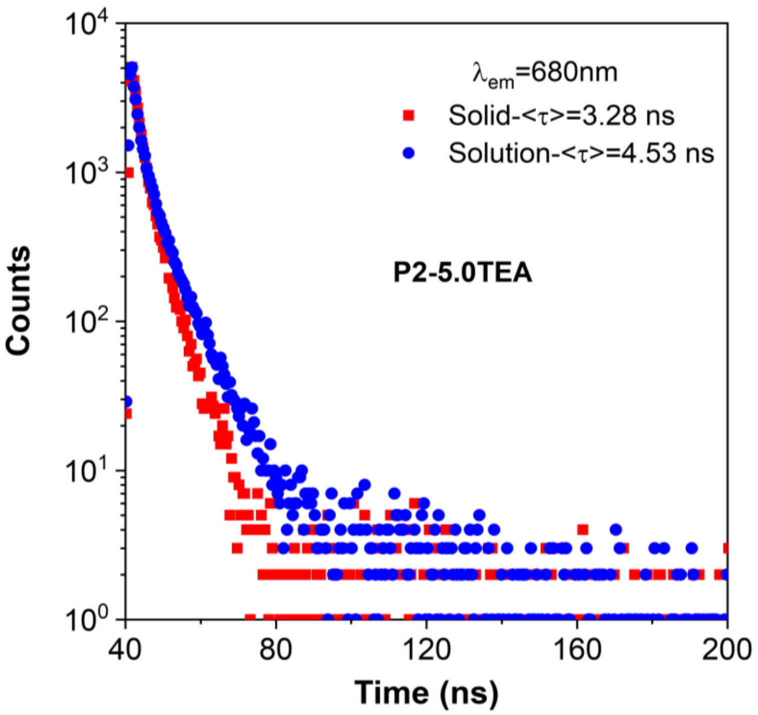

Supplementary Figure 152. Time-resolved PL decay curves of **P2-5.0TEA** measured at emission maximum of 680 nm in solid and DCM solution (c = 10^{-3} M), λ_{ex} = 470 nm.

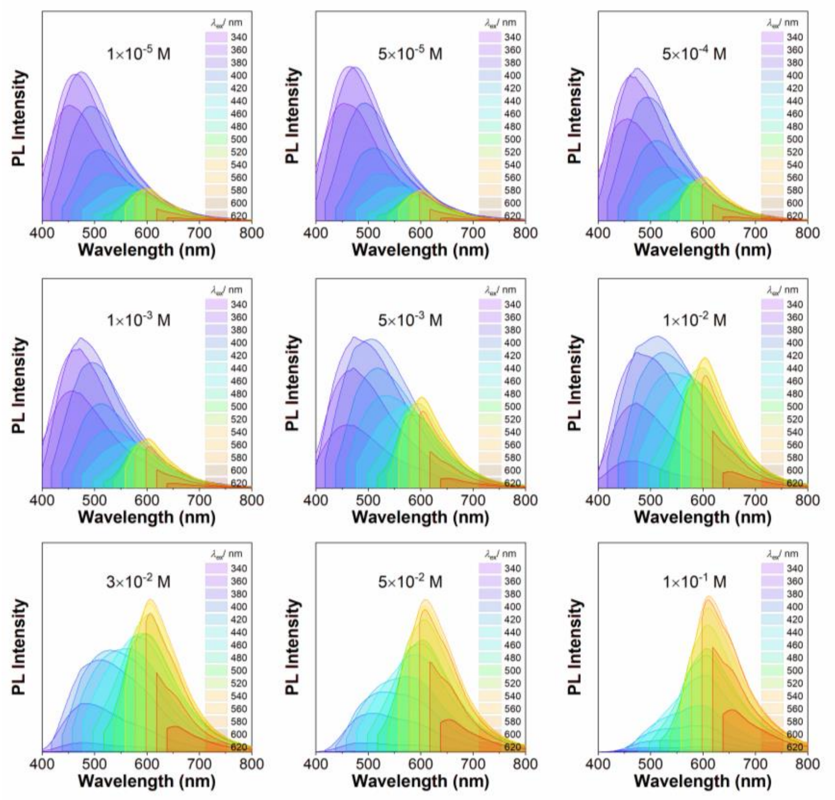

Supplementary Figure 153. Concentration-dependent PL spectra (A)~(I) of **P2-0.5TEA** in DCM solution under different excitation wavelengths. Concentration: from 10^{-5} to 10^{-1} M.

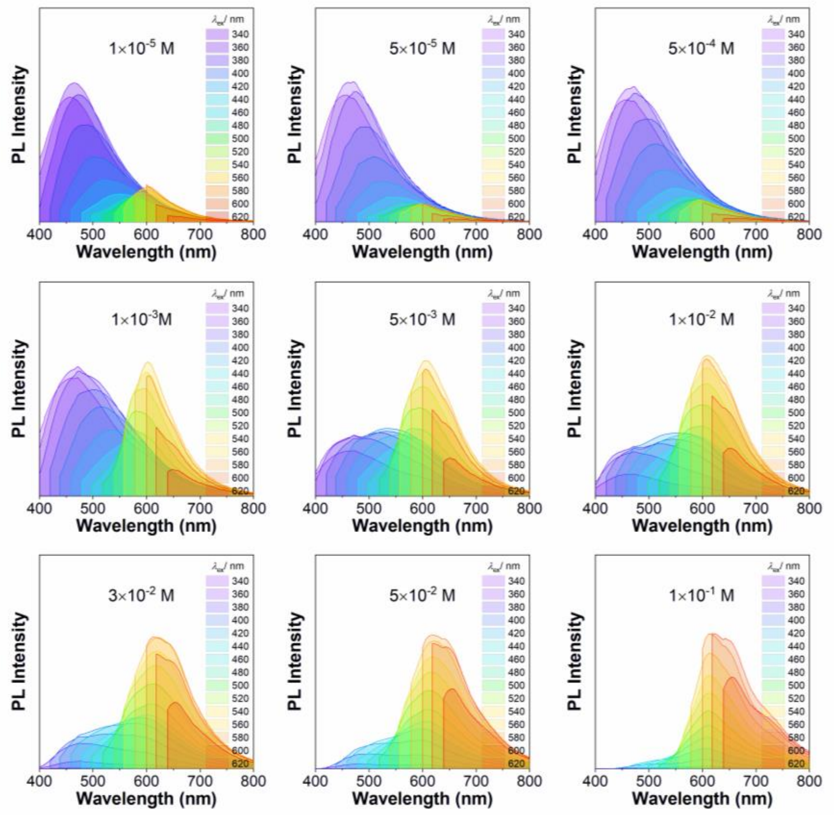

Supplementary Figure 154. Concentration-dependent PL spectra (A)~(I) of **P2-1.0TEA** in DCM solution under different excitation wavelengths. Concentration: from 10^{-5} to 10^{-1} M.

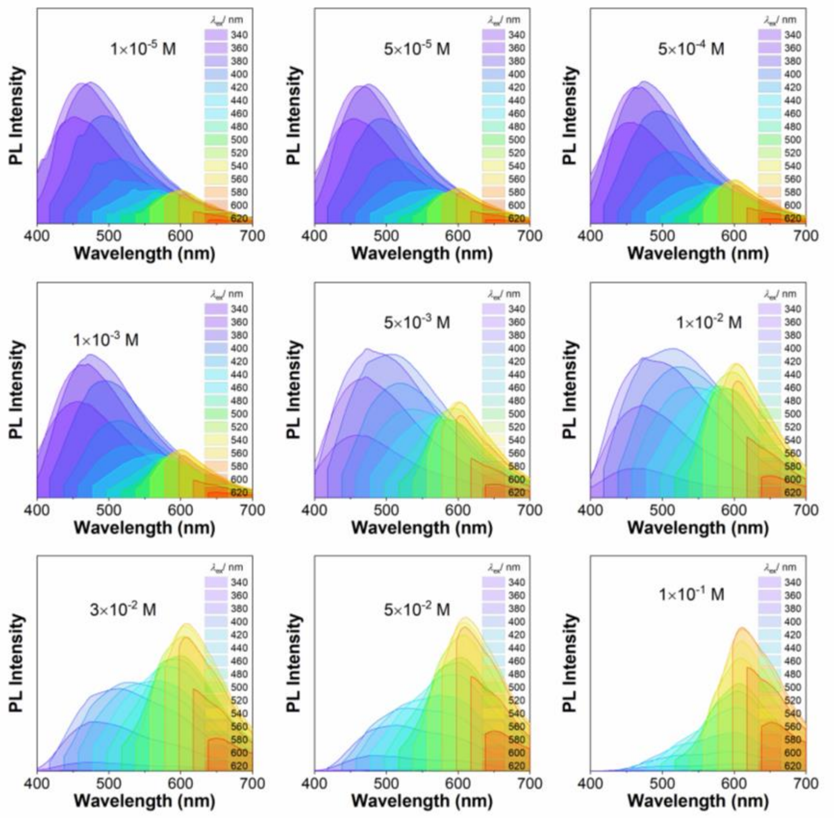

Supplementary Figure 155. Concentration-dependent PL spectra (A)~(I) of **P2-1.5TEA** in DCM solution under different excitation wavelengths. Concentration: from 10^{-5} to 10^{-1} M.

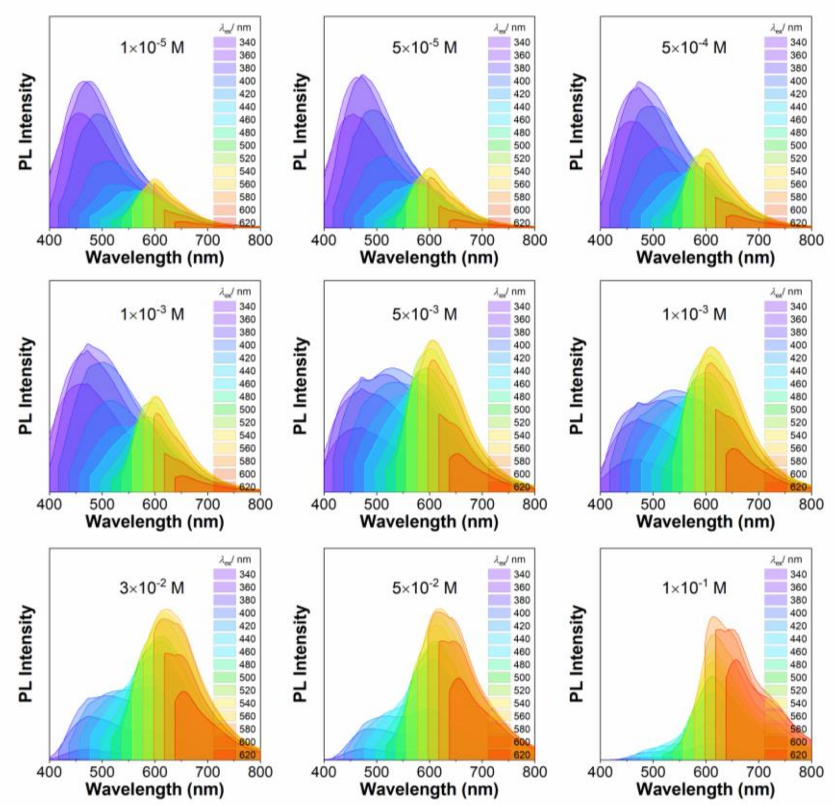

Supplementary Figure 156. Concentration-dependent PL spectra (A)~(I) of **P2-2.0TEA** in DCM solution under different excitation wavelengths. Concentration: from 10^{-5} to 10^{-1} M.

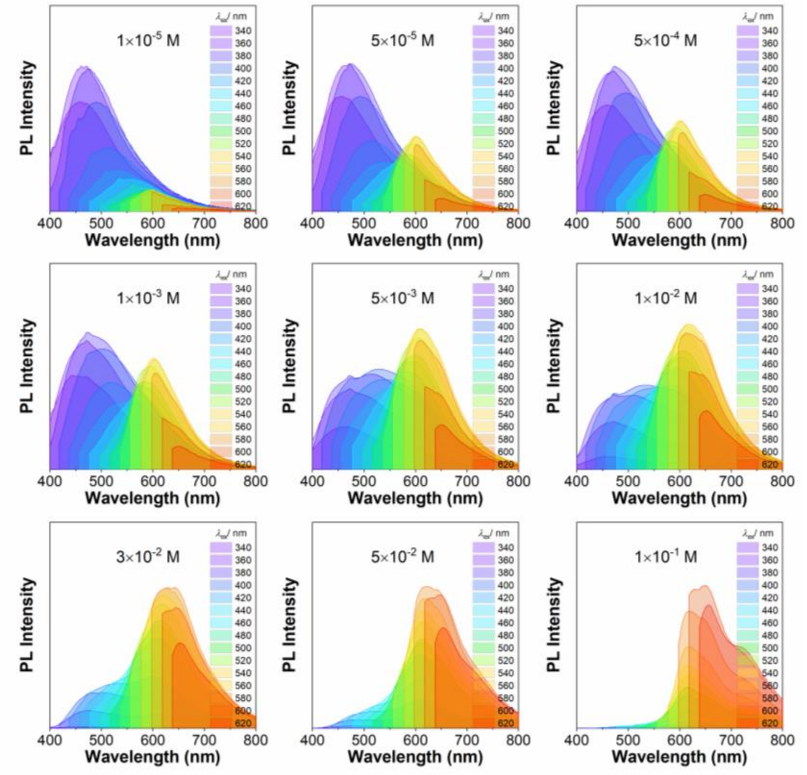

Supplementary Figure 157. Concentration-dependent PL spectra (A)~(I) of **P2-3.0TEA** in DCM solution under different excitation wavelengths. Concentration: from 10^{-5} to 10^{-1} M.

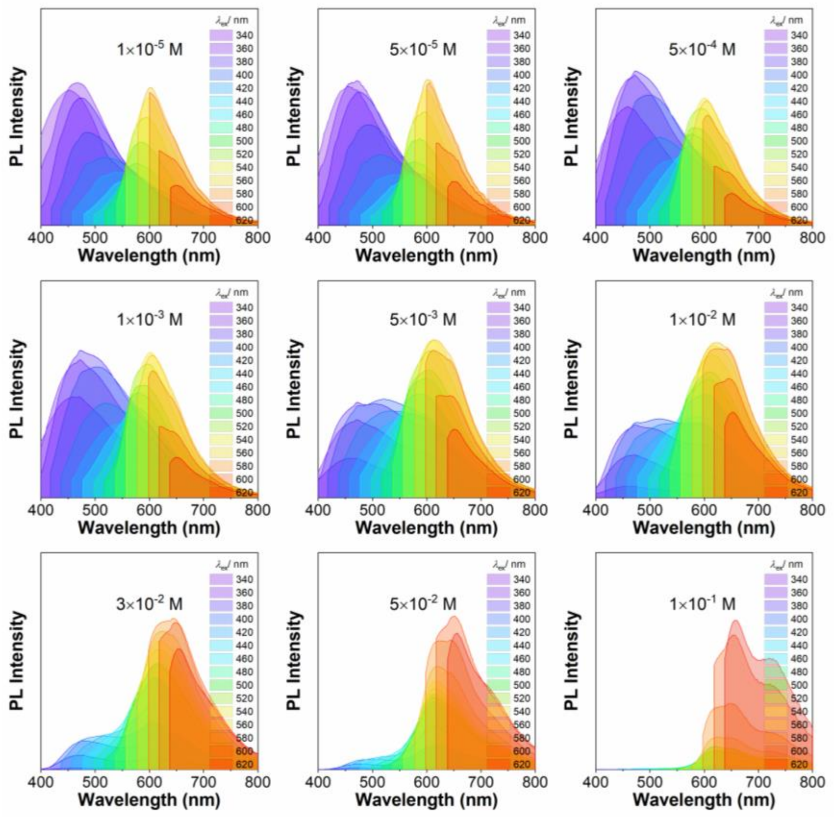

Supplementary Figure 158. Concentration-dependent PL spectra (A)~(I) of **P2-4.0TEA** in DCM solution under different excitation wavelengths. Concentration: from 10^{-5} to 10^{-1} M.

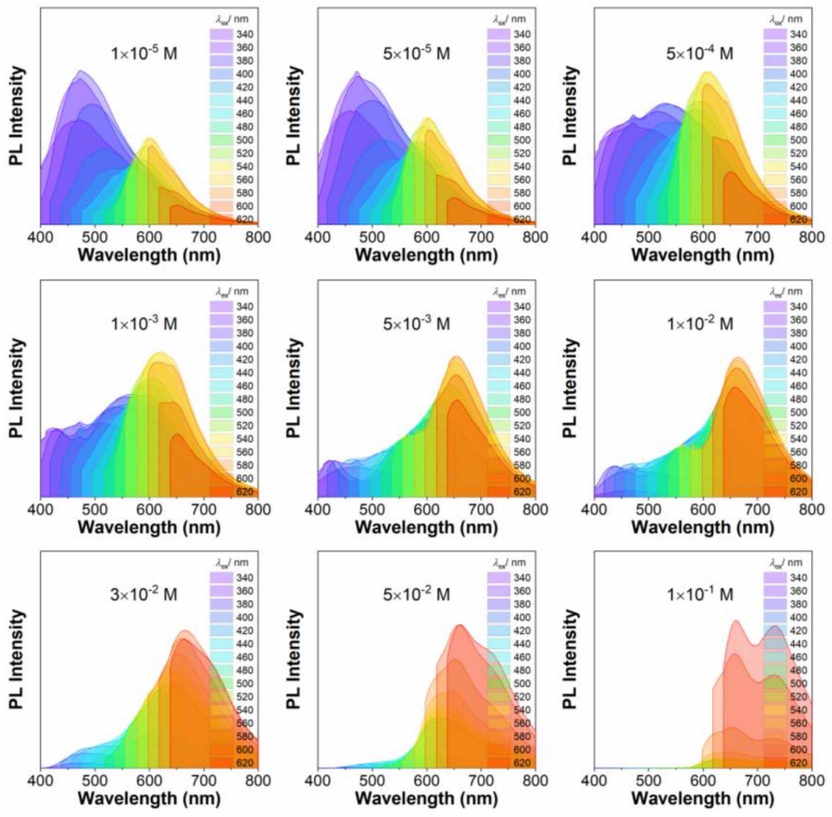

Supplementary Figure 159. Concentration-dependent PL spectra (A)~(I) of **P2-5.0TEA** in DCM solution under different excitation wavelengths. Concentration: from 10^{-5} to 10^{-1} M.

Photophysical characterization of P1-2.0amine and P2-2.0amine

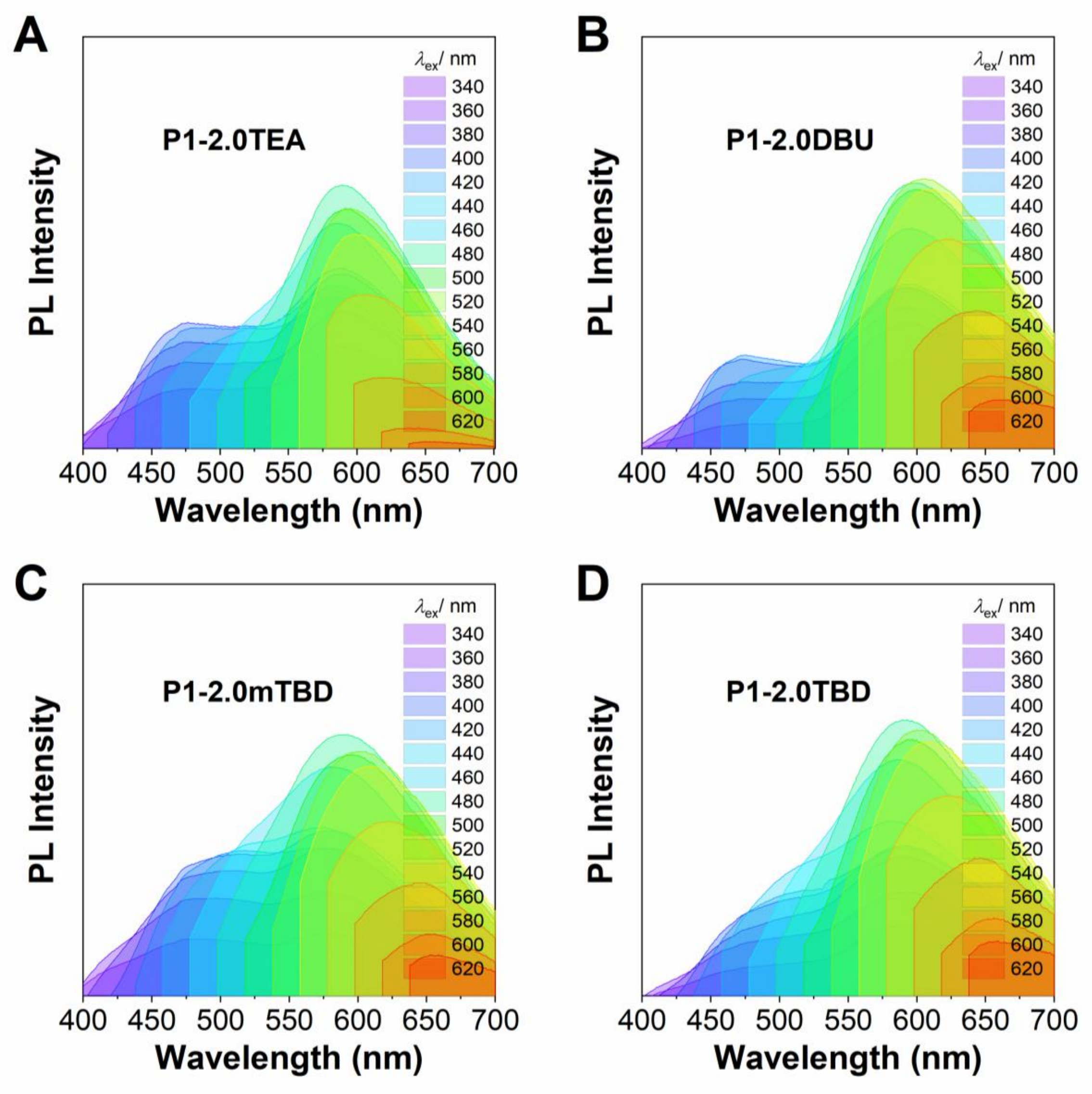

Supplementary Figure 160. PL spectra of (A)**P1-2.0TEA**, (B) **P1-2.0DBU**, (C) **P1-2.0mTBD** and (D) **P1-2.0TBD** in solid under different excitation wavelengths.

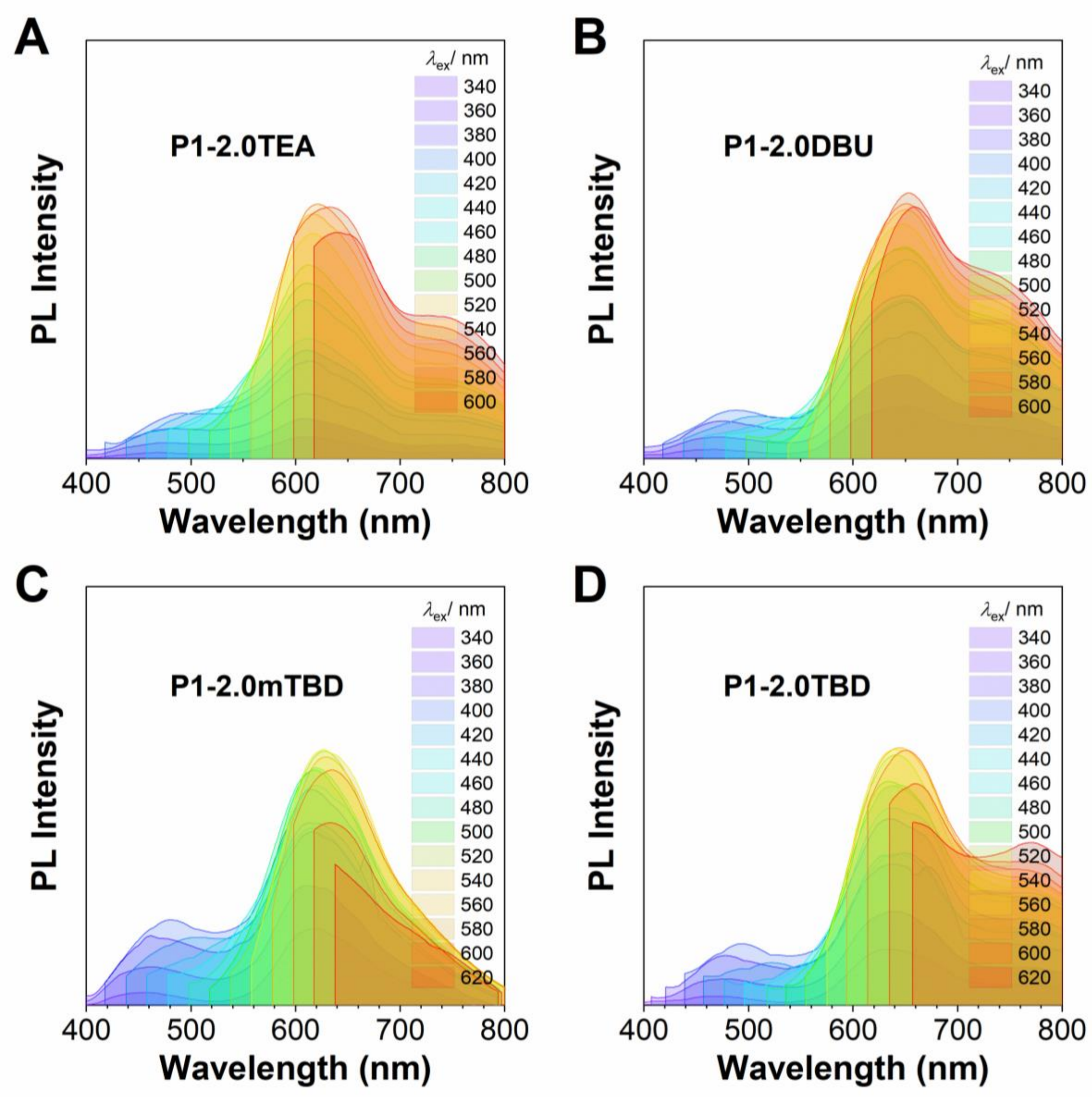

Supplementary Figure 161. PL spectra of (A)**P2-2.0TEA**, (B) **P2-2.0DBU**, (C) **P2-2.0mTBD** and (D) **P2-2.0TBD** in solid under different excitation wavelengths.

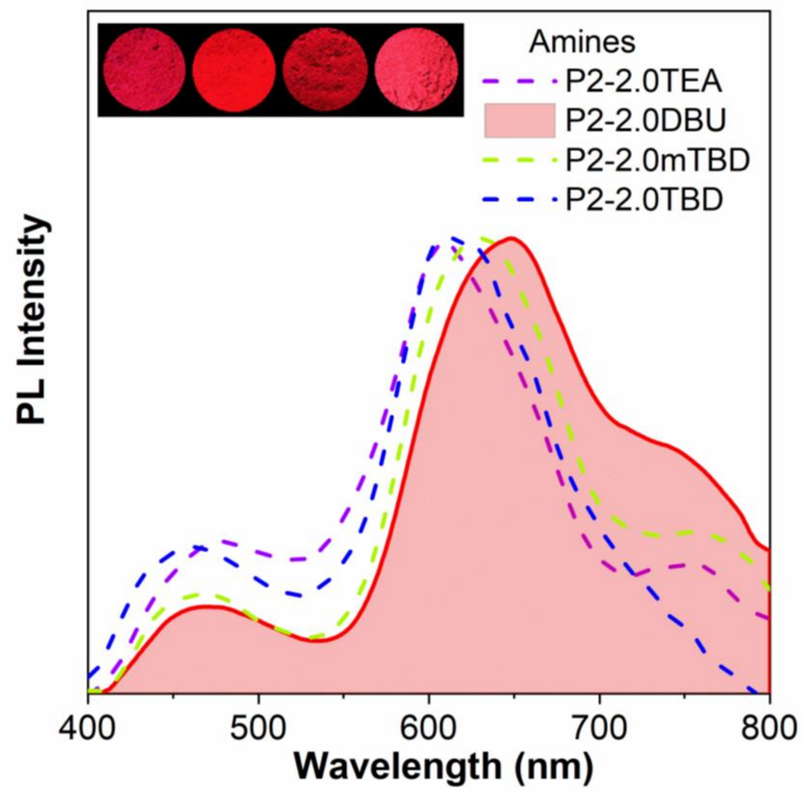

Supplementary Figure 162. Normalzied PL spectra of **P2-2.0TEA**, **P2-2.0DBU**, **P2-2.0mTBD** and **P2-2.0TBD** in solid at λ_{ex} = 360 nm. Inset: photographs taken under 365 nm UV light.

Dynamic NMR spectra of complexation

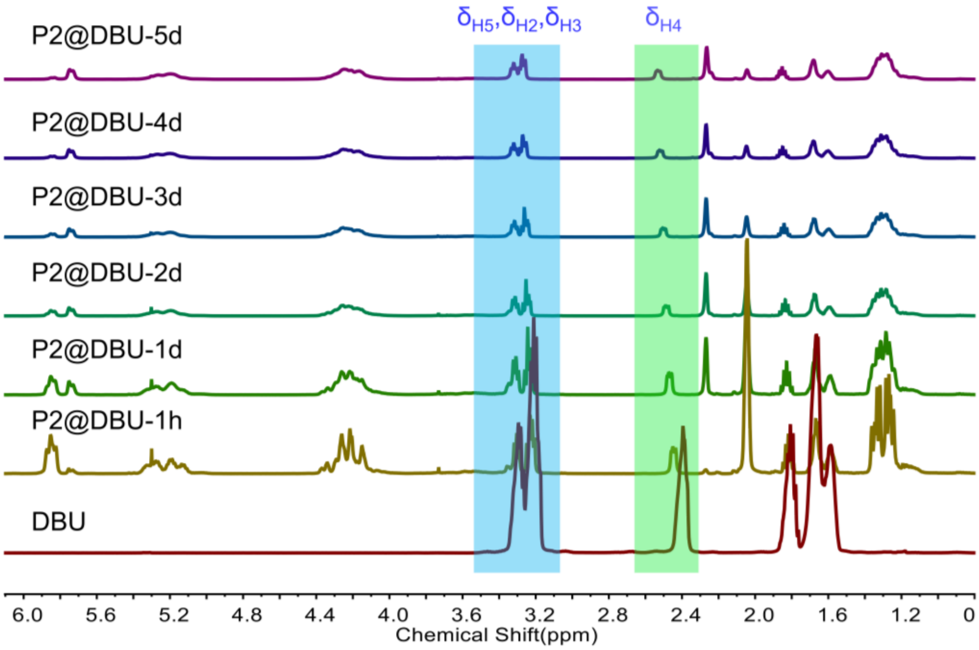

Supplementary Figure 163. Time-dependent ^{1}H-NMR spectra of **P2@DBU** (400 MHz, $CDCl_3$). The molar ratio of structural units of **P2** to DBU is 5:1. The h and d respectively represent hours and days.

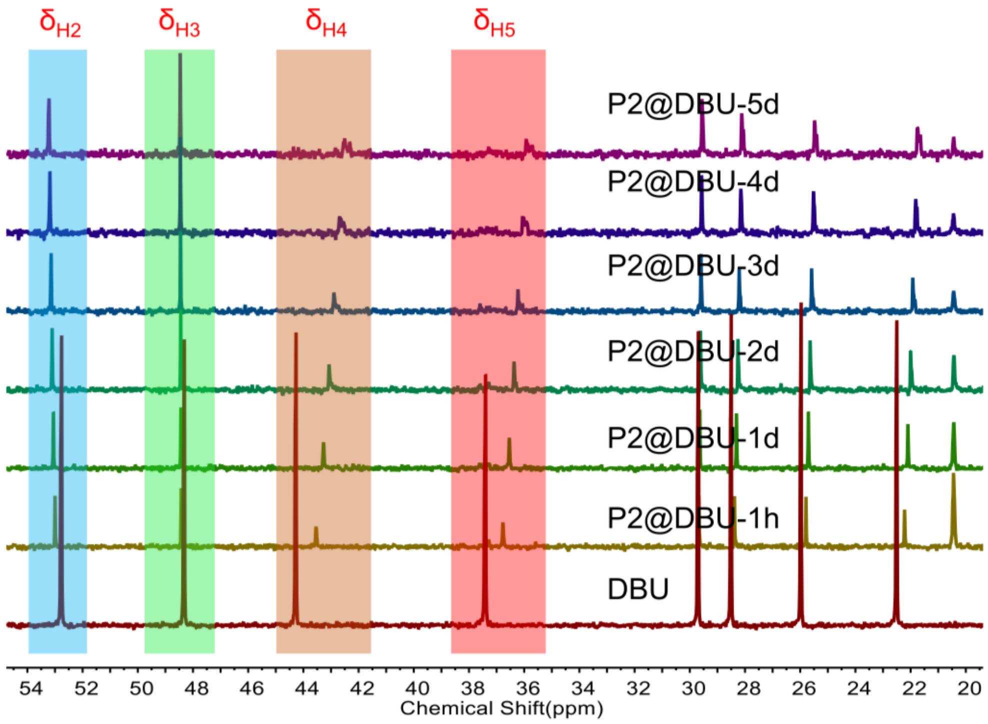

Supplementary Figure 164. Time-dependent ^{13}C-NMR spectra of **P2@DBU** (100 MHz, $CDCl_3$). The molar ratio of structural units of **P2** to DBU is 5:1. The h and d respectively represent hours and days.

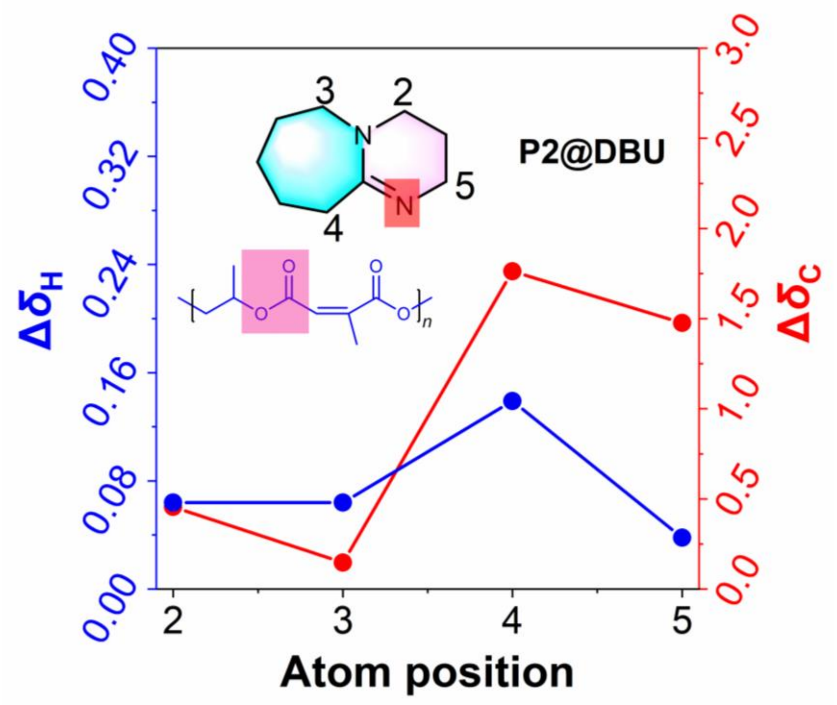

Supplementary Figure 165. Plots of absolute value of chemical shift changes ($\Delta\delta\Delta\delta_H$ for hydrogen atoms, $\Delta\delta\Delta\delta_C$ for carbon atoms) versus atom positions on DBU for **P2@DBU**.

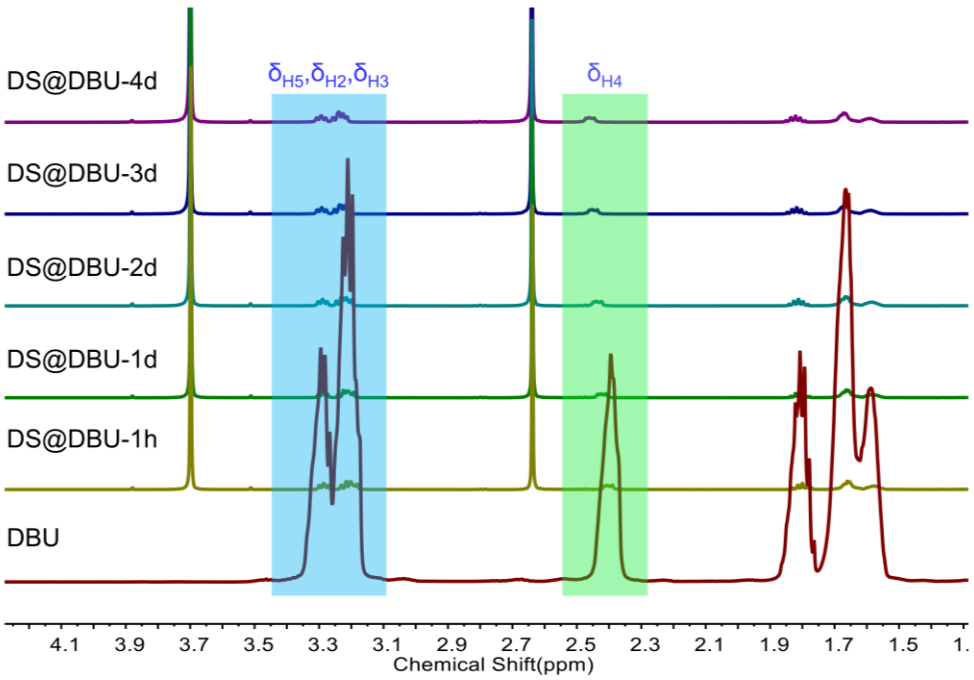

Supplementary Figure 166. Time-dependent ^{1}H-NMR spectra of **DS@DBU** (400 MHz, $CDCl_3$). The molar ratio of structural units of DS to DBU is 5:1. The h and d respectively represent hours and days.

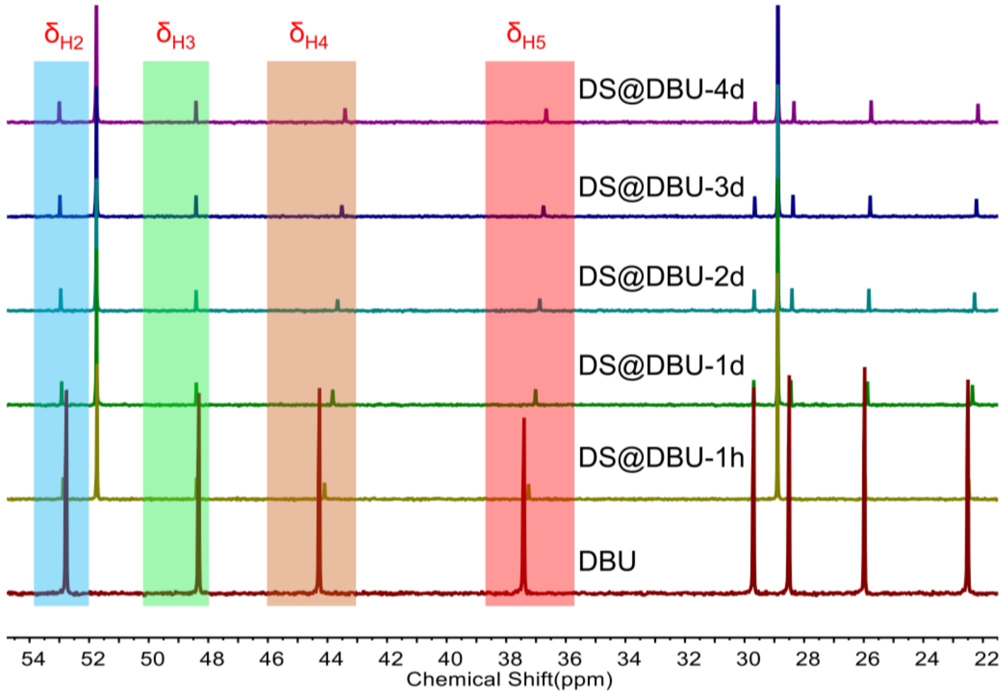

Supplementary Figure 167. Time-dependent ^{13}C-NMR spectra of **DS@DBU** (100 MHz, $CDCl_3$). The molar ratio of structural units of DS to DBU is 5:1. The h and d respectively represent hours and days.

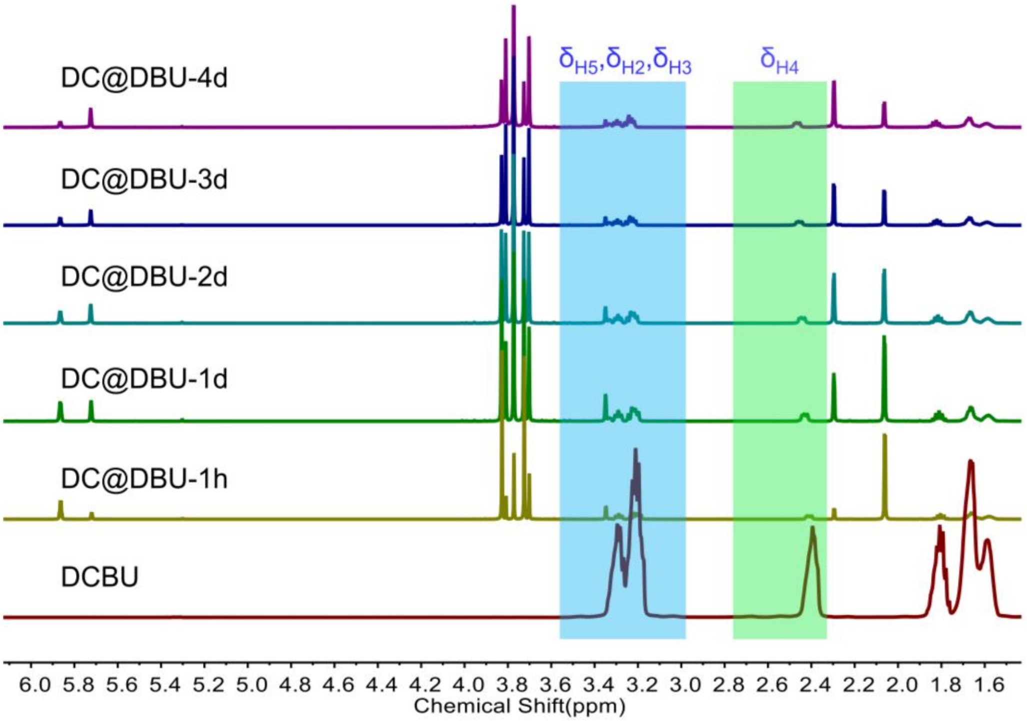

Supplementary Figure 168. Time-dependent ^{1}H-NMR spectra of **DC@DBU** (400 MHz, $CDCl_3$).. The molar ratio of structural units of DC to DBU is 5:1. The h and d respectively represent hours and days.

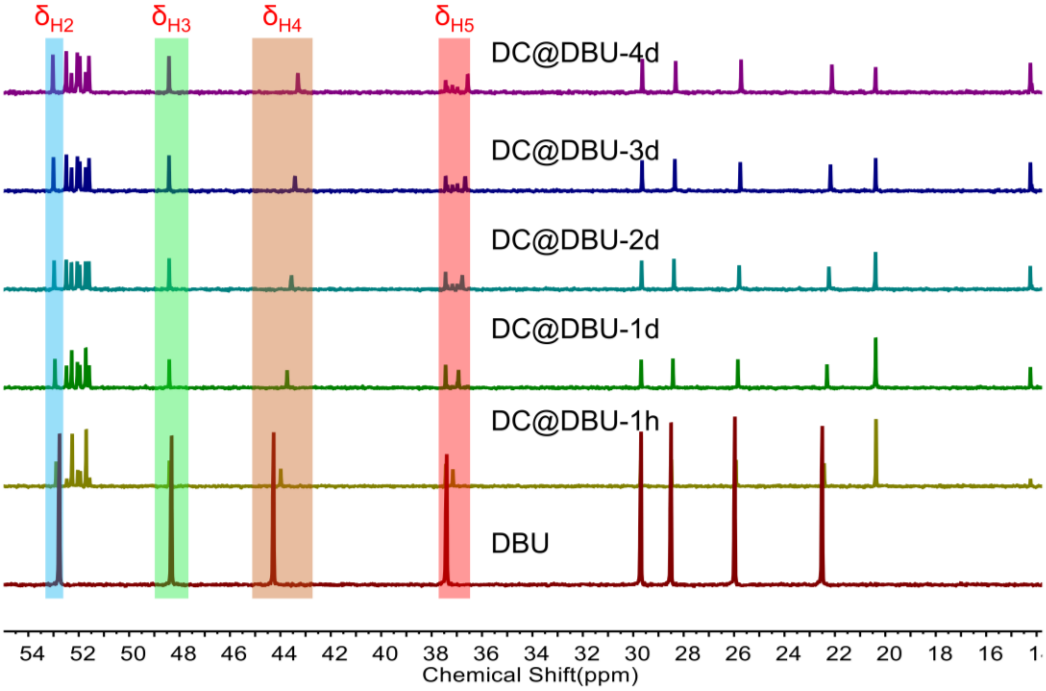

Supplementary Figure 169. Time-dependent ^{13}C-NMR spectra of **DC@DBU** (100 MHz, $CDCl_3$).. The molar ratio of structural units of DC to DBU is 5:1. The h and d respectively represent hours and days.

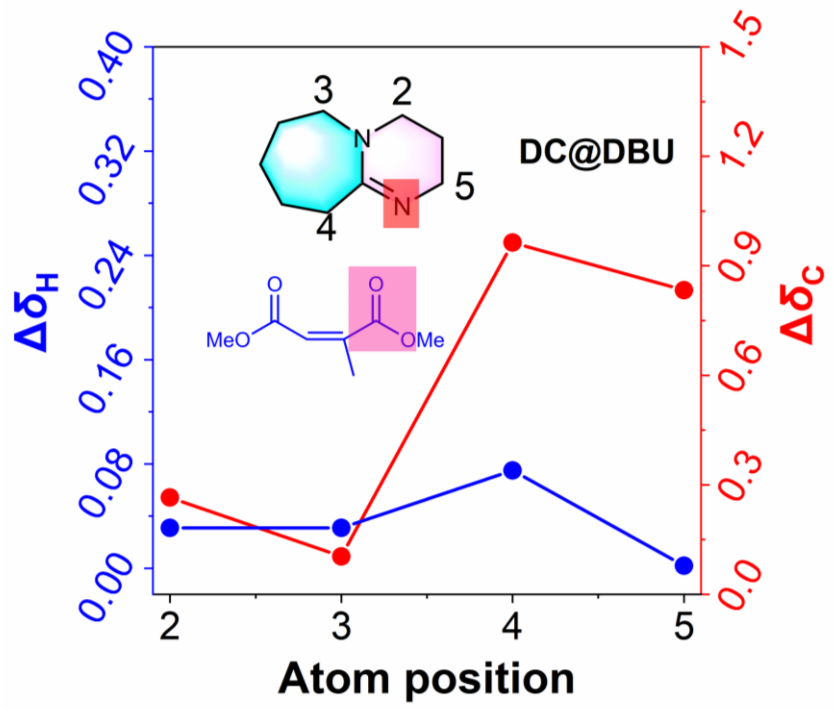

Supplementary Figure 170. Plots of absolute value of chemical shift changes ($\Delta\delta_H$ for hydrogen atoms, $\Delta\delta_C$ for carbon atoms) versus atom positions on DBU for **DC@DBU**.

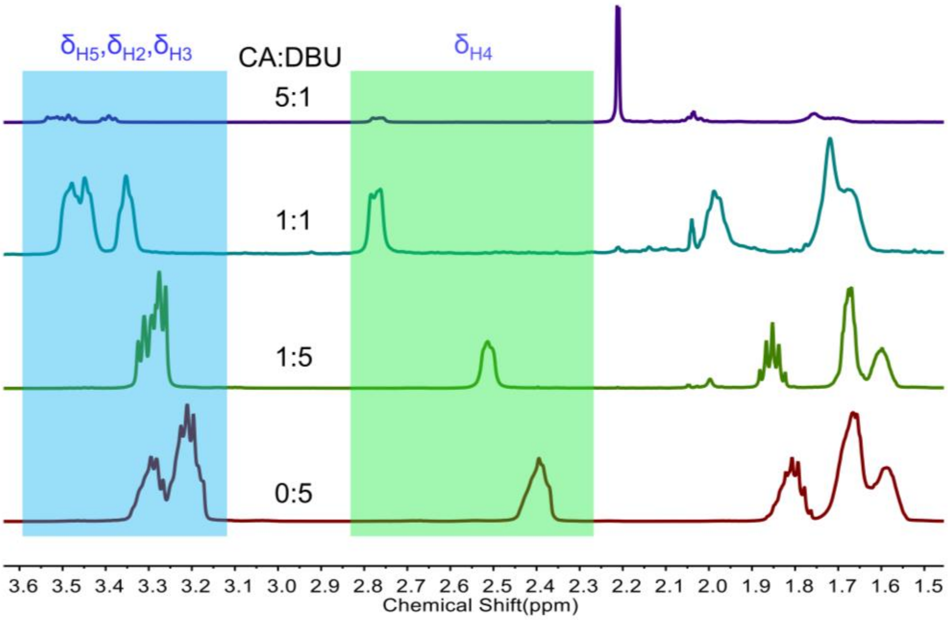

Supplementary Figure 171. Stacked ^{1}H-NMR spectra of **CA@DBU** with molar ratio of structural units of CA to DBU is from 5:0 to 0:5 (400 MHz, $CDCl_3$).

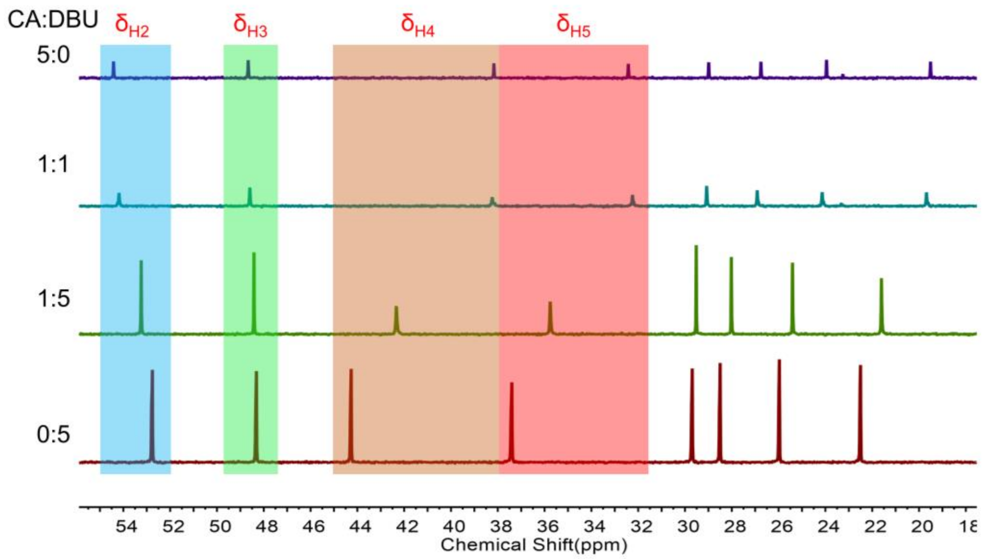

Supplementary Figure 172. Stacked ^{13}C-NMR spectra of **CA@DBU** with molar ratio of structural units of CA to DBU is from 5:0 to 0:5 (100 MHz, $CDCl_3$).

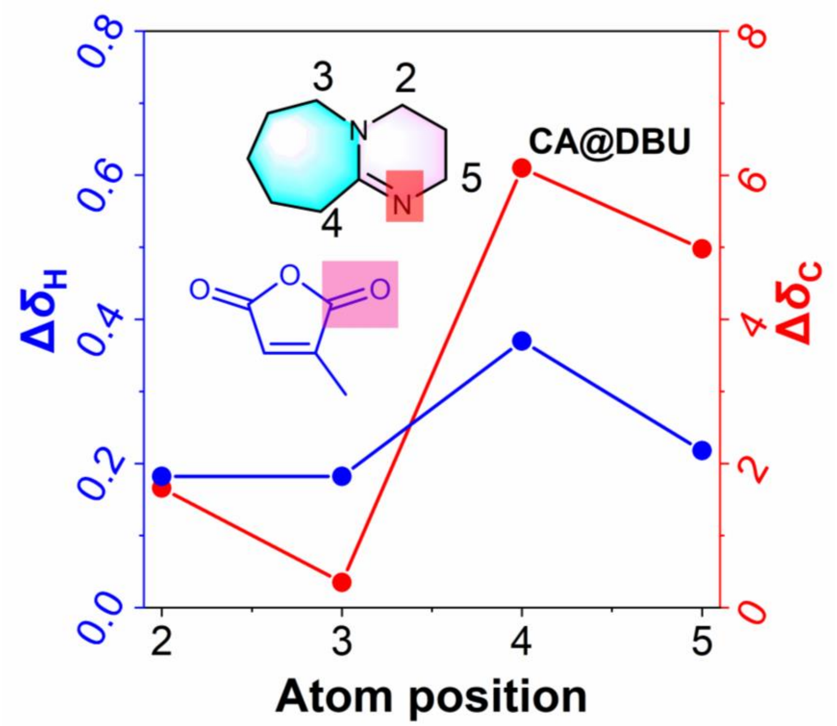

Supplementary Figure 173. Plots of absolute value of chemical shift changes ($\Delta\delta_H$ for hydrogen atoms, $\Delta\delta_C$ for carbon atoms) versus atom positions on DBU for **CA@DBU**.

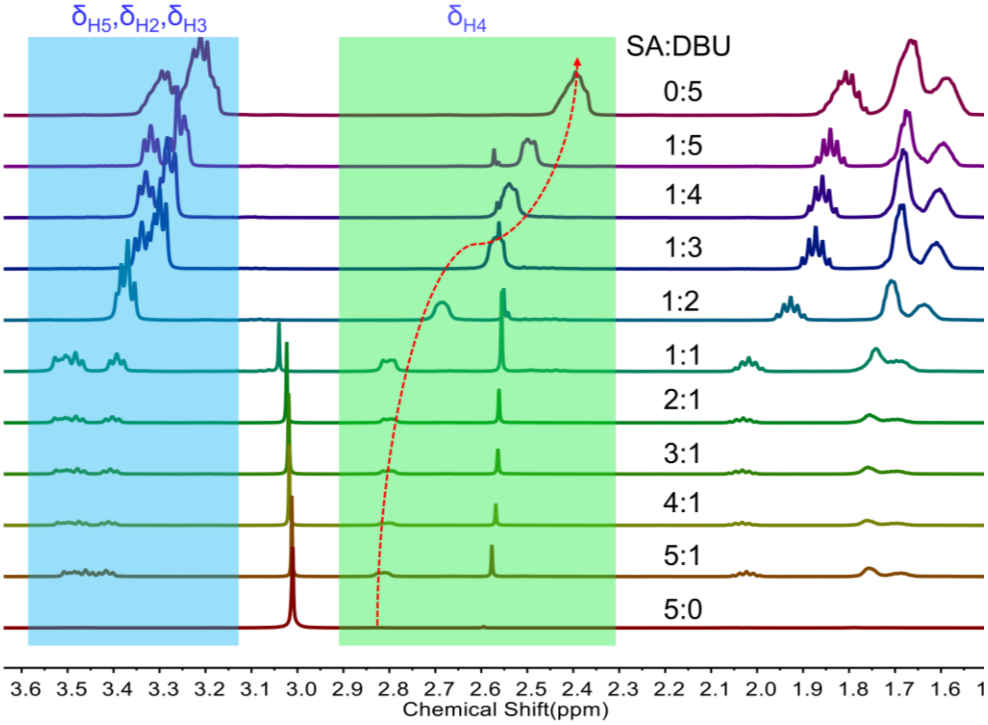

Supplementary Figure 174. Stacked ^{1}H-NMR spectra of **SA@DBU** with molar ratio of structural units of SA to DBU is from 5:0 to 0:5 (400 MHz, $CDCl_3$).

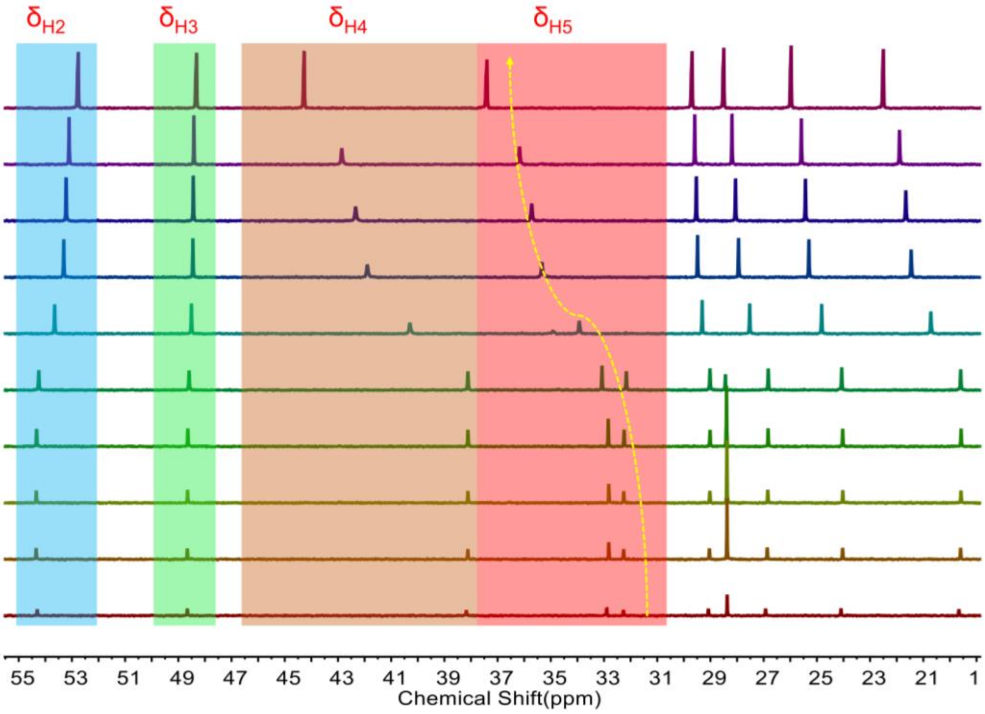

Supplementary Figure 175. Stacked ^{13}C-NMR spectra of **SA@DBU** with molar ratio of structural units of SA to DBU is from 5:0 to 0:5 (100 MHz, $CDCl_3$).

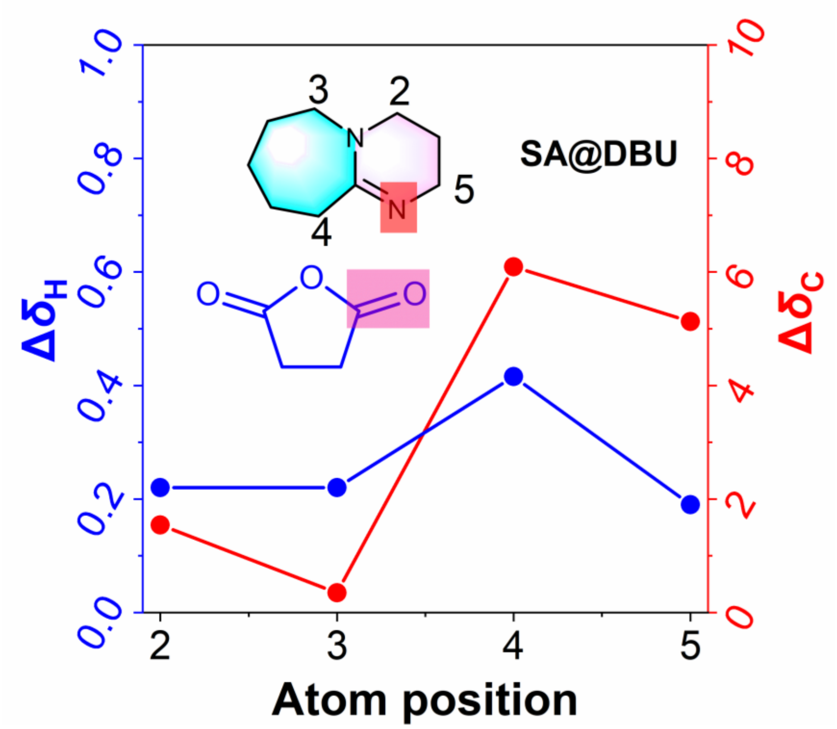

Supplementary Figure 176. Plots of absolute value of chemical shift changes ($\Delta\delta_H$ for hydrogen atoms, $\Delta\delta_C$ for carbon atoms) versus atom positions on DBU for **SA@DBU**.

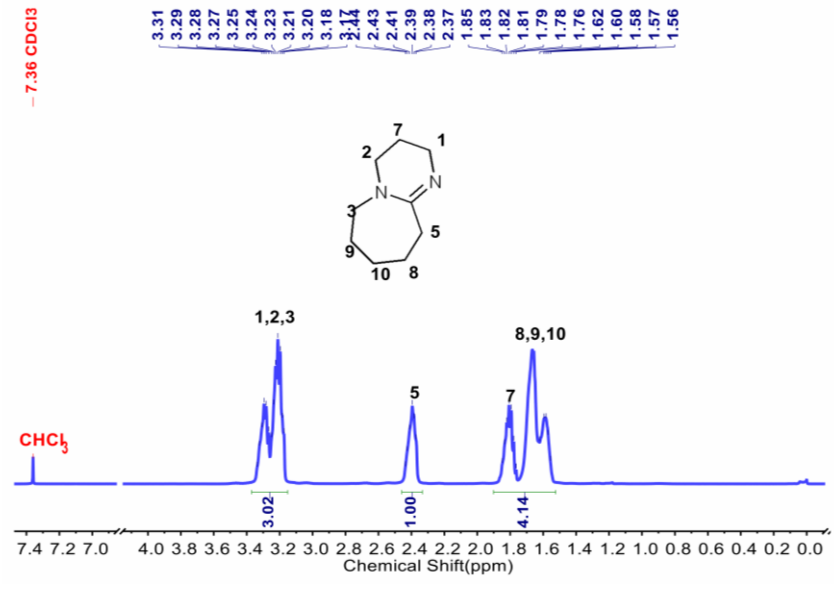

Supplementary Figure 177. ^{1}H-NMR spectra of DBU (400 MHz, $CDCl_3$).

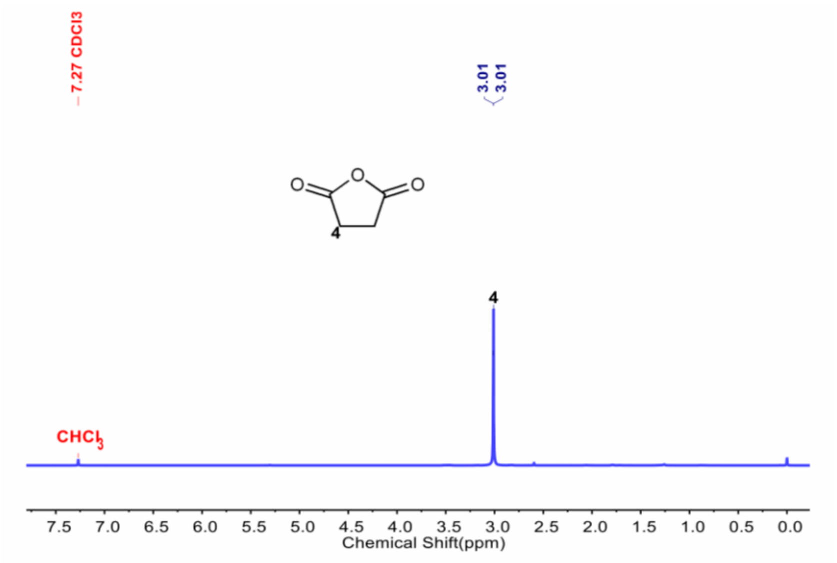

Supplementary Figure 178. ^{1}H-NMR spectra of SA (400 MHz, $CDCl_3$).

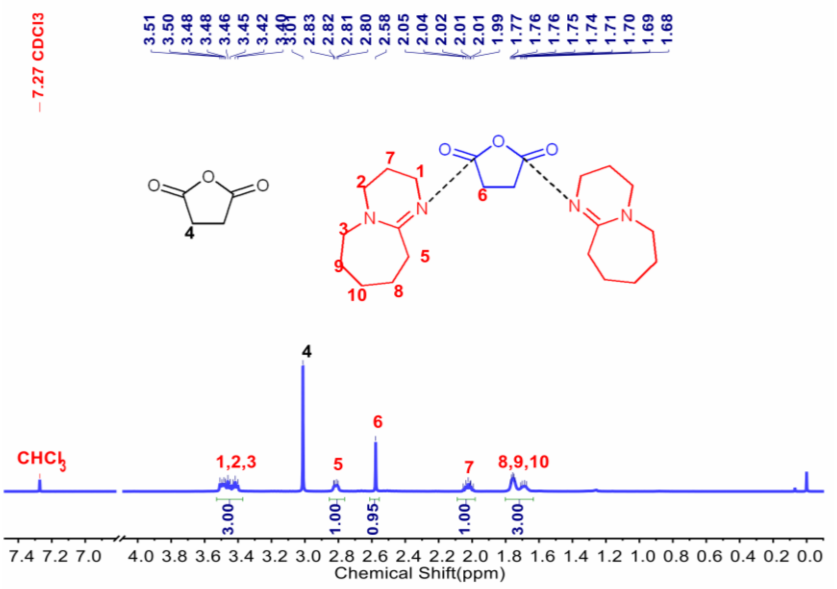

Supplementary Figure 179. ^{1}H-NMR spectra of **SA@DBU** with molar ratio of structural units of SA to DBU is 5:1 (400 MHz, $CDCl_3$).

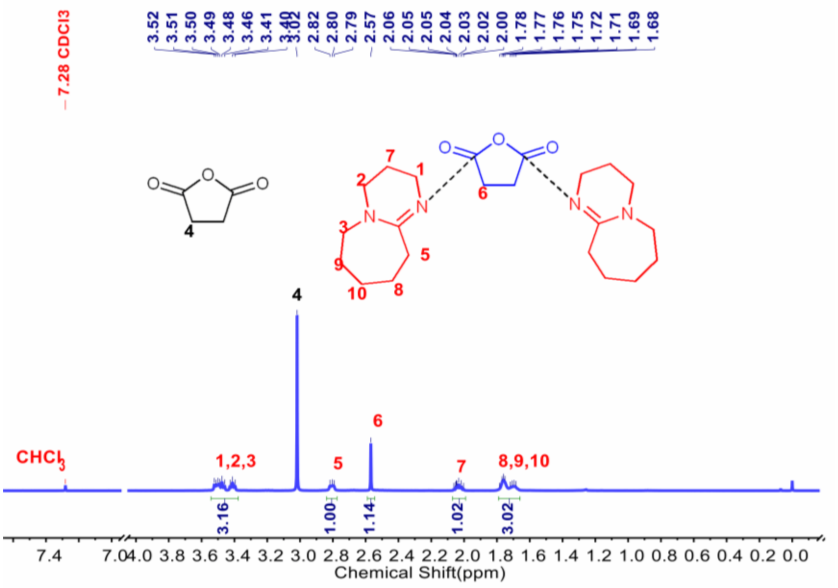

Supplementary Figure 180. ^{1}H-NMR spectra of **SA@DBU** with molar ratio of structural units of SA to DBU is 4:1 (400 MHz, $CDCl_3$).

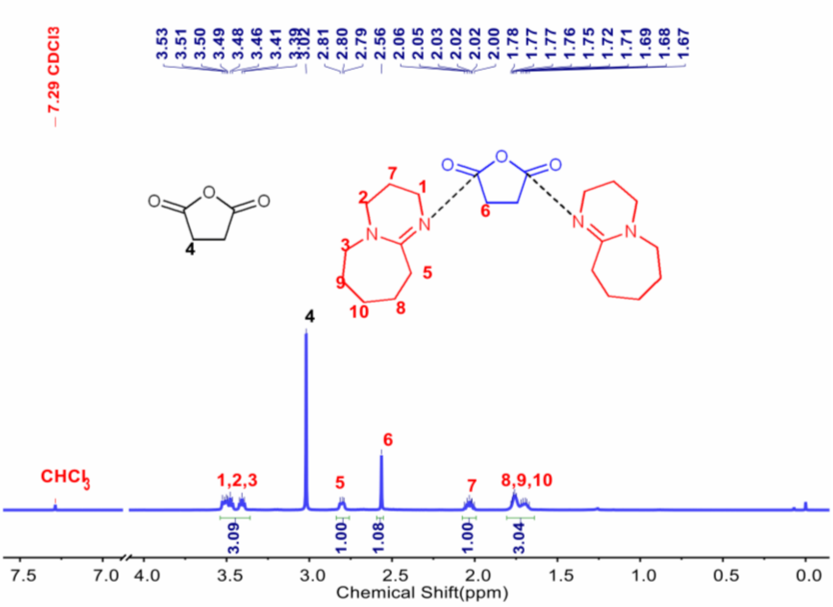

Supplementary Figure 181. ^{1}H-NMR spectra of **SA@DBU** with molar ratio of structural units of SA to DBU is 3:1 (400 MHz, $CDCl_3$).

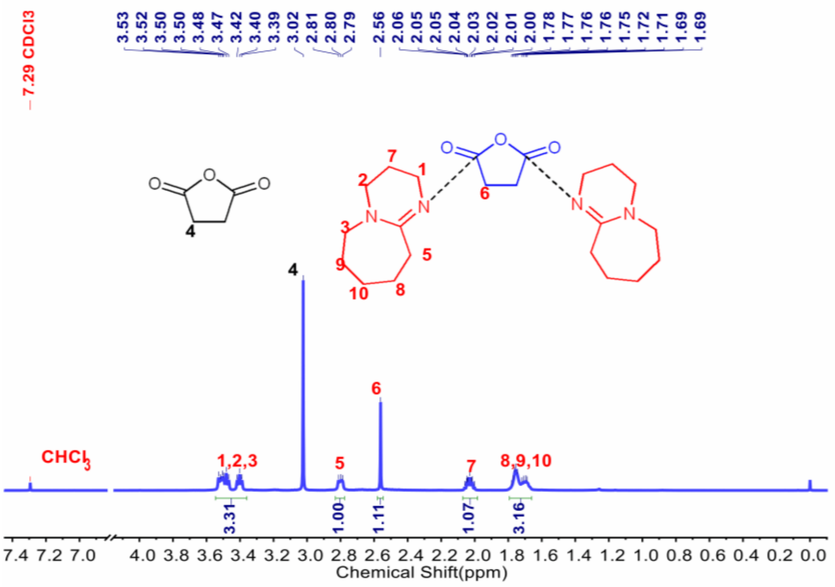

Supplementary Figure 182. ^{1}H-NMR spectra of **SA@DBU** with molar ratio of structural units of SA to DBU is 2:1 (400 MHz, $CDCl_3$).

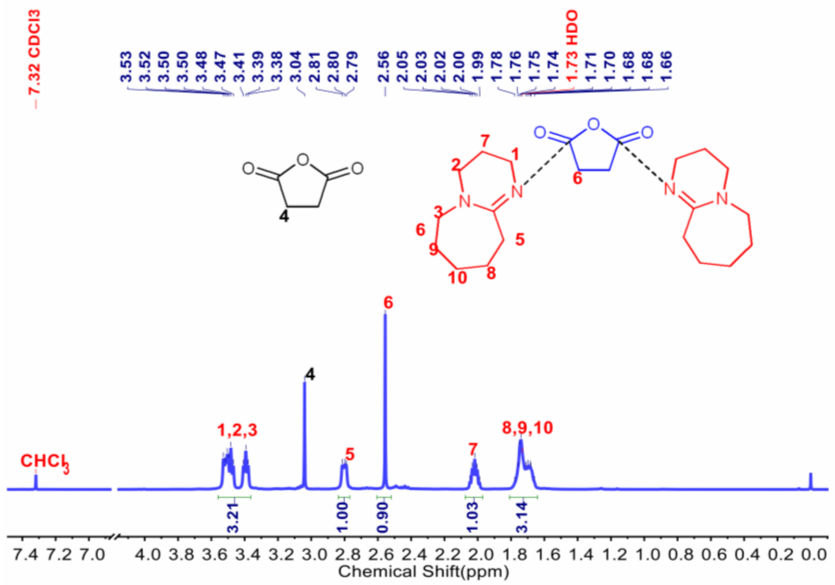

Supplementary Figure 183. ^{1}H-NMR spectra of **SA@DBU** with molar ratio of structural units of SA to DBU is 1:1 (400 MHz, $CDCl_3$).

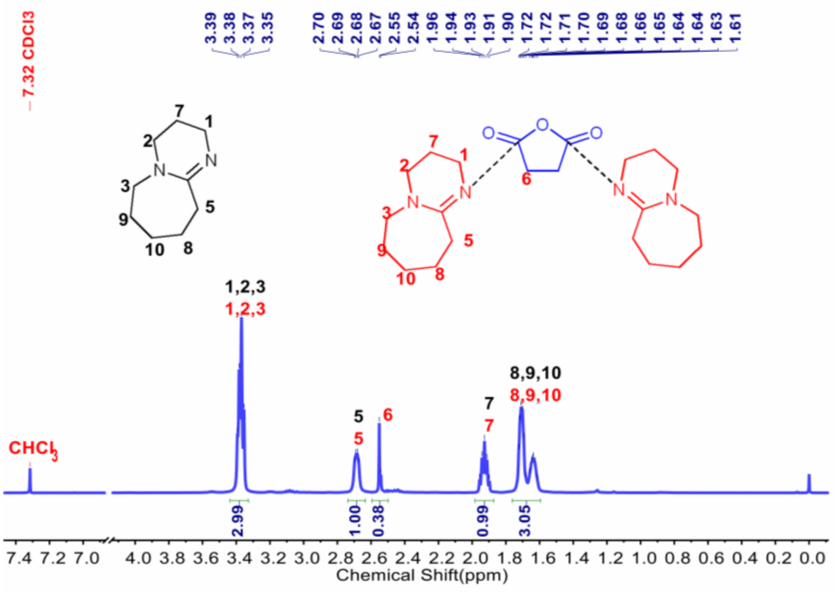

Supplementary Figure 184. ^{1}H-NMR spectra of **SA@DBU** with molar ratio of structural units of SA to DBU is 1:2 (400 MHz, $CDCl_3$).

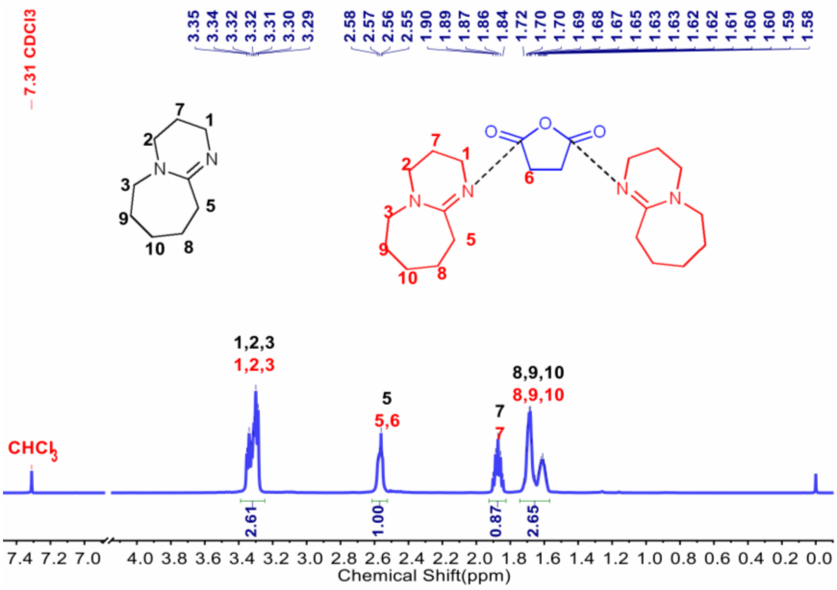

Supplementary Figure 185. ^{1}H-NMR spectra of **SA@DBU** with molar ratio of structural units of SA to DBU is 1:3 (400 MHz, $CDCl_3$).

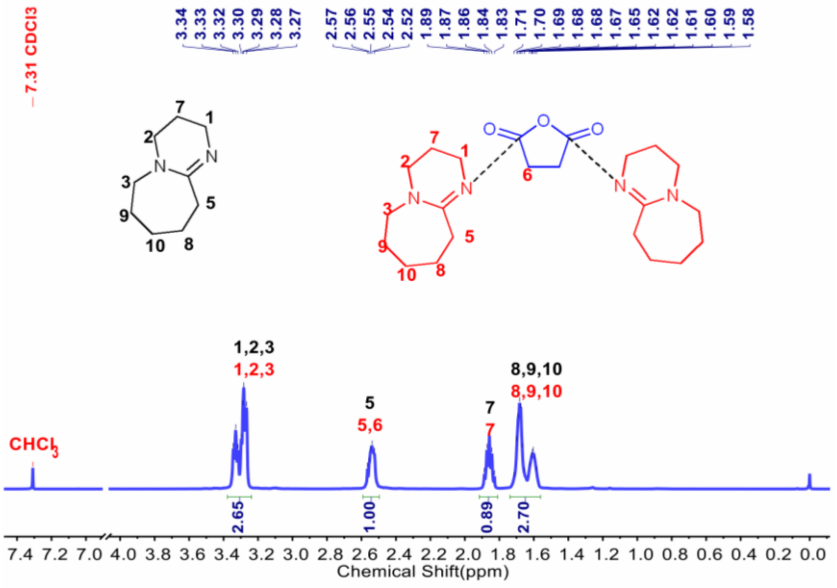

Supplementary Figure 186. ^{1}H-NMR spectra of **SA@DBU** with molar ratio of structural units of SA to DBU is 1:4 (400 MHz, $CDCl_3$).

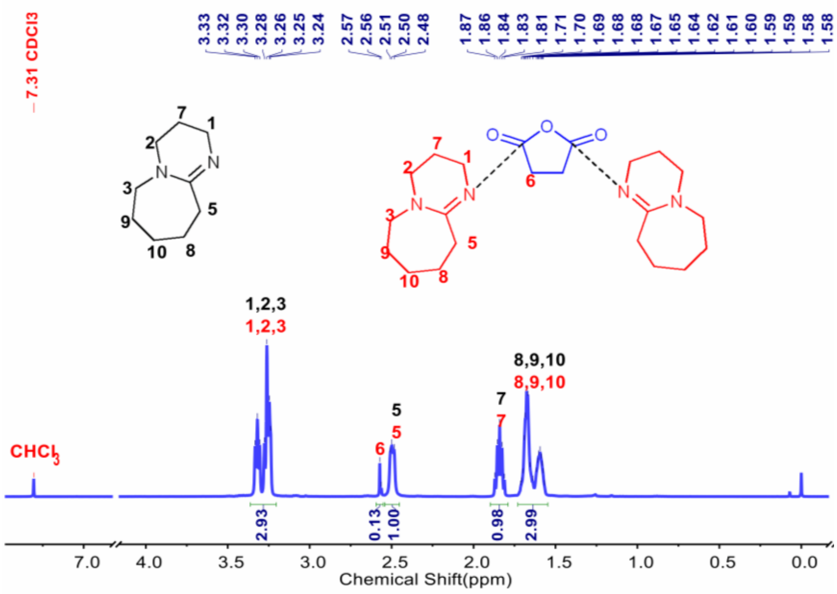

Supplementary Figure 187. ^{1}H-NMR spectra of **SA@DBU** with molar ratio of structural units of SA to DBU is 1:5 (400 MHz, $CDCl_3$).

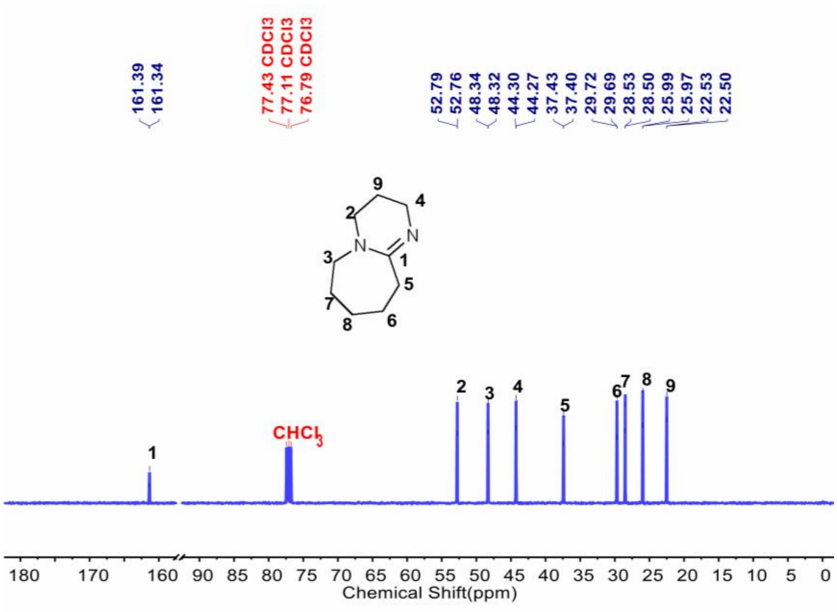

Supplementary Figure 188. ^{13}C-NMR spectra of DBU (100 MHz, $CDCl_3$).

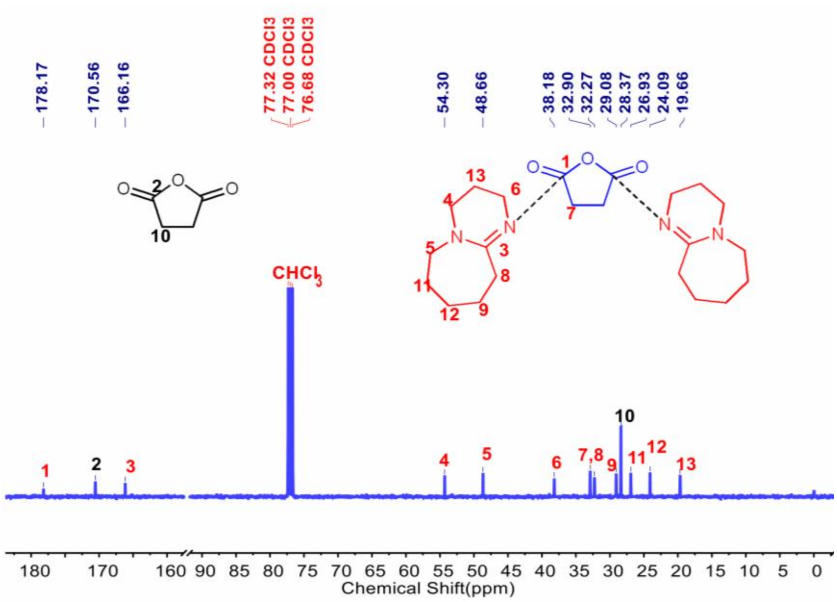

Supplementary Figure 189. ^{13}C-NMR spectra of **SA@DBU** with molar ratio of structural units of SAS to DBU is 5:1 (100 MHz, $CDCl_3$).

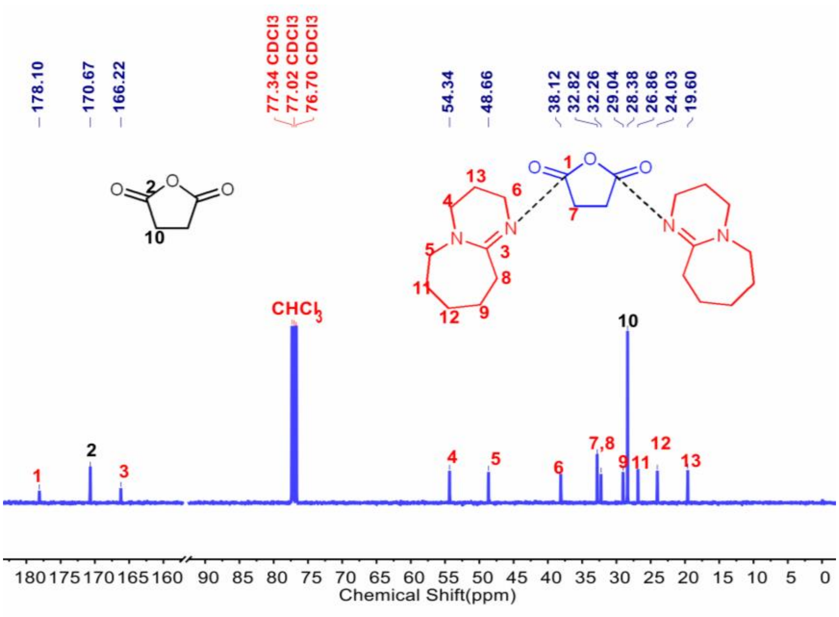

Supplementary Figure 190. ^{13}C-NMR spectra of **SA@DBU** with molar ratio of structural units of SA to DBU is 4:1 (100 MHz, $CDCl_3$).

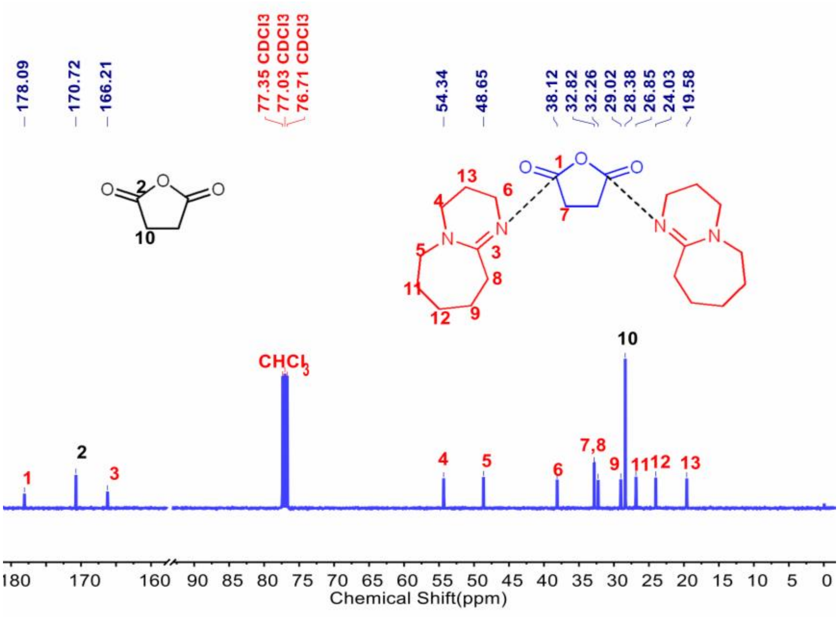

Supplementary Figure 191. ^{13}C-NMR spectra of **SA@DBU** with molar ratio of structural units of SA to DBU is 3:1 (100 MHz, $CDCl_3$).

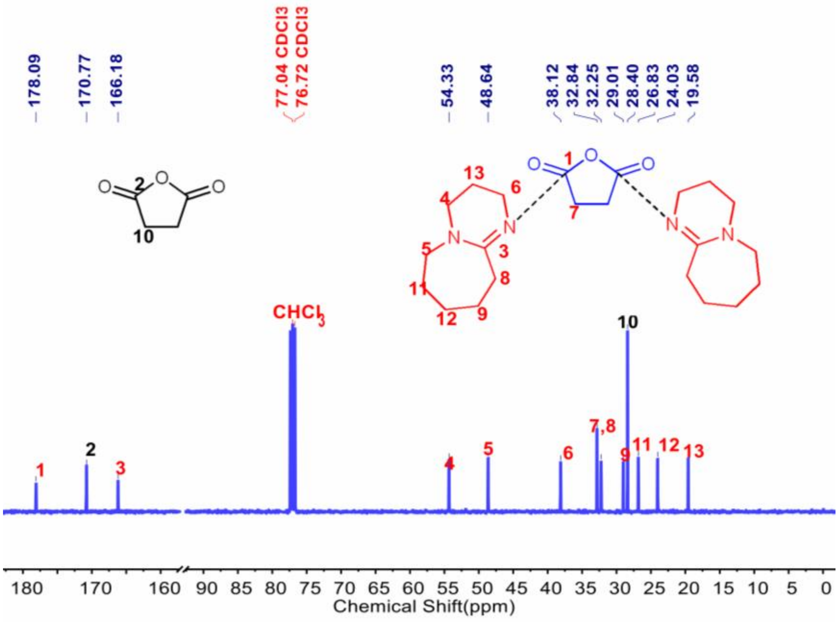

Supplementary Figure 192. ^{13}C-NMR spectra of **SA@DBU** with molar ratio of structural units of SA to DBU is 2:1 (100 MHz, $CDCl_3$).

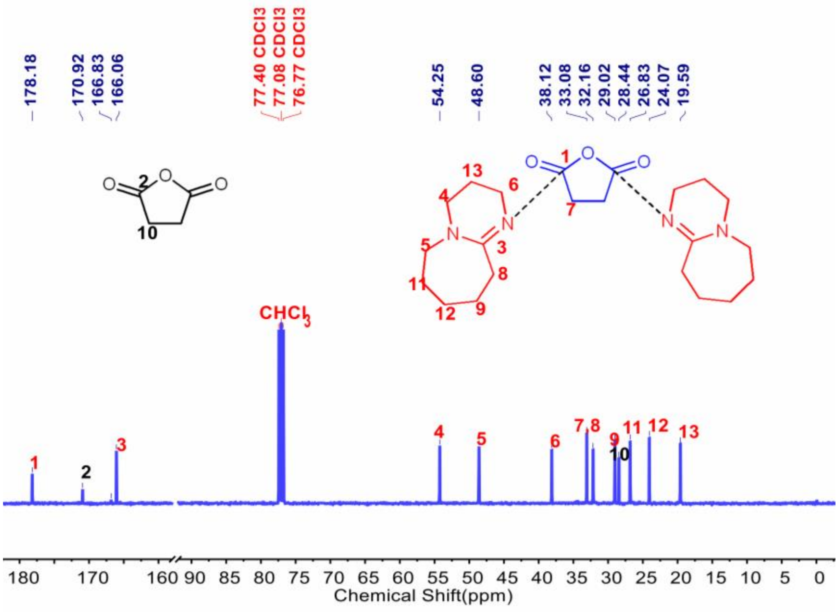

Supplementary Figure 193. ^{13}C-NMR spectra of **SA@DBU** with molar ratio of structural units of SA to DBU is 1:1 (100 MHz, $CDCl_3$).

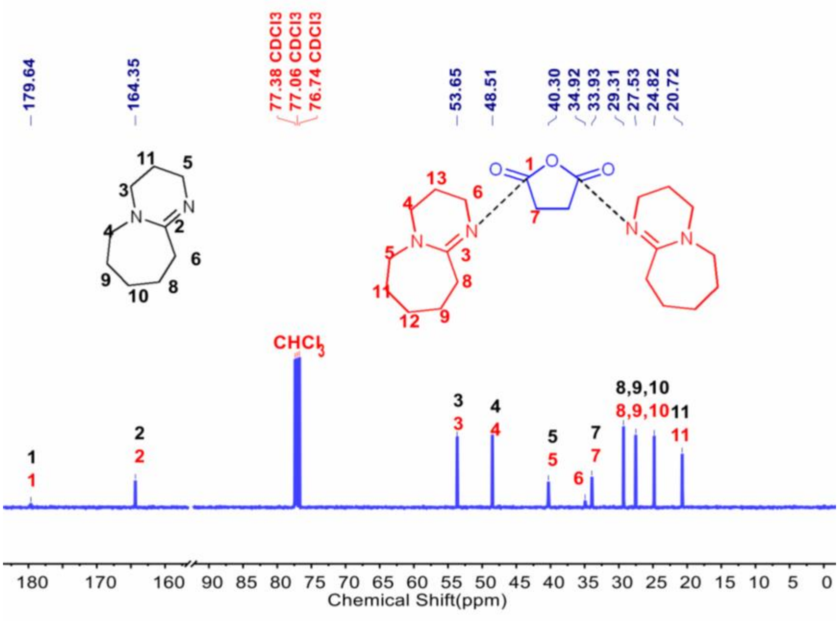

Supplementary Figure 194. ^{13}C-NMR spectra of **SA@DBU** with molar ratio of structural units of SA to DBU is 1:2 (100 MHz, $CDCl_3$).

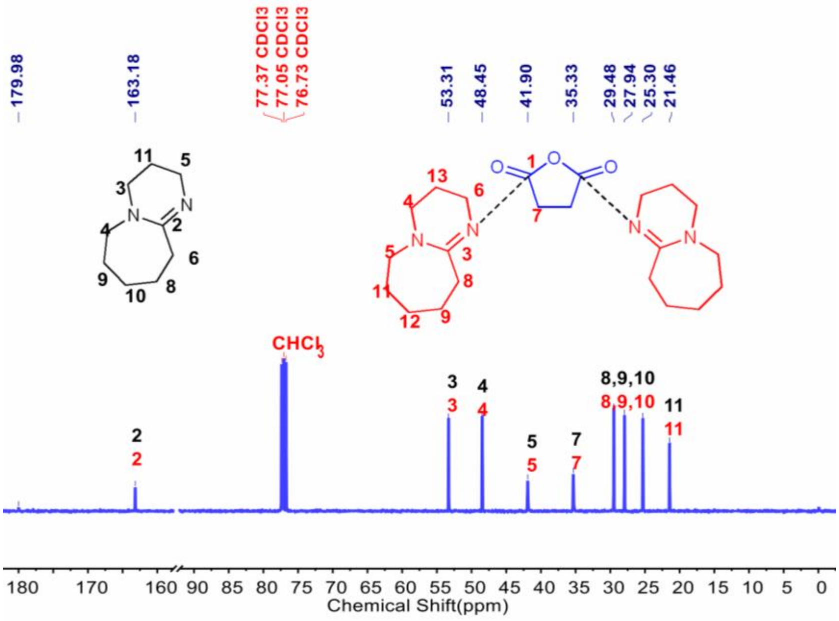

Supplementary Figure 195. ^{13}C-NMR spectra of **SA@DBU** with molar ratio of structural units of SA to DBU is 1:3 (100 MHz, $CDCl_3$).

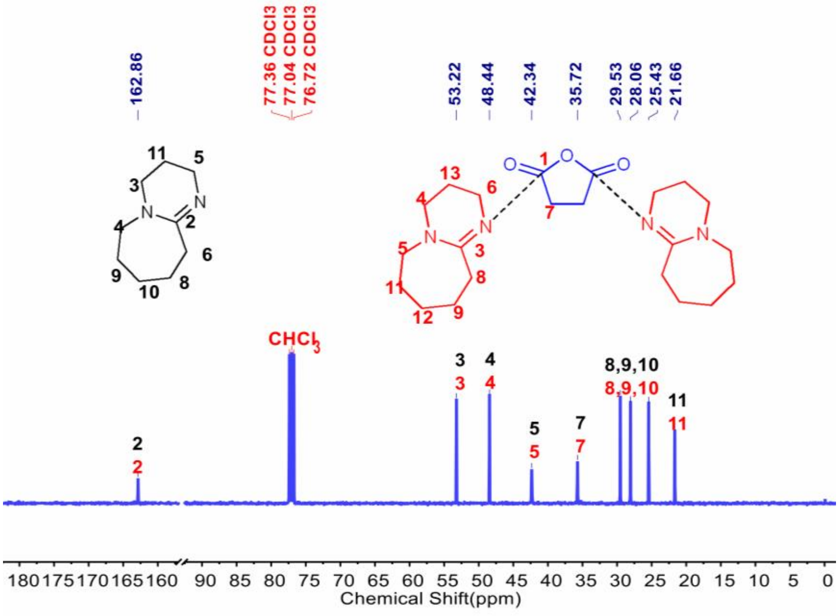

Supplementary Figure 196. ^{13}C-NMR spectra of **SA@DBU** with molar ratio of structural units of SA to DBU is 1:4 (100 MHz, $CDCl_3$).

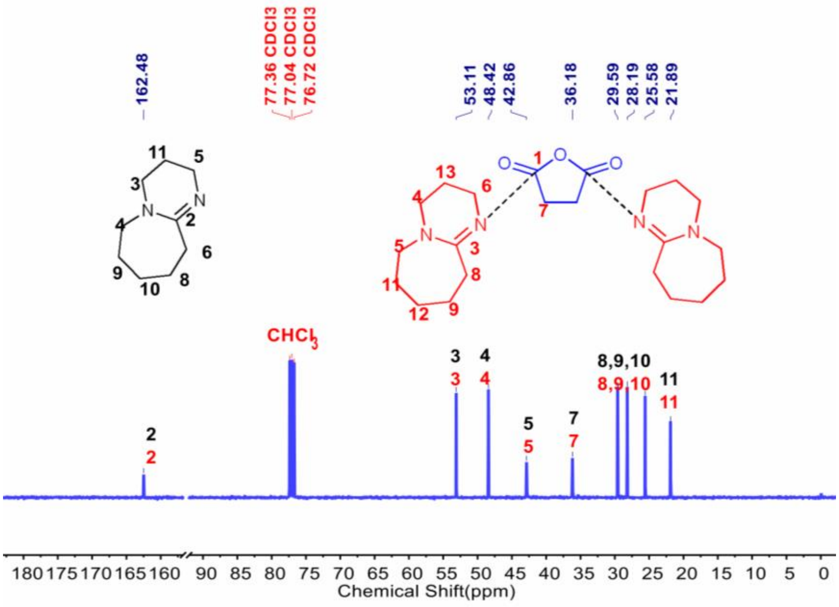

Supplementary Figure 197. ^{13}C-NMR spectra of **SA@DBU** with molar ratio of structural units of SA to DBU is 1:5 (100 MHz, $CDCl_3$).

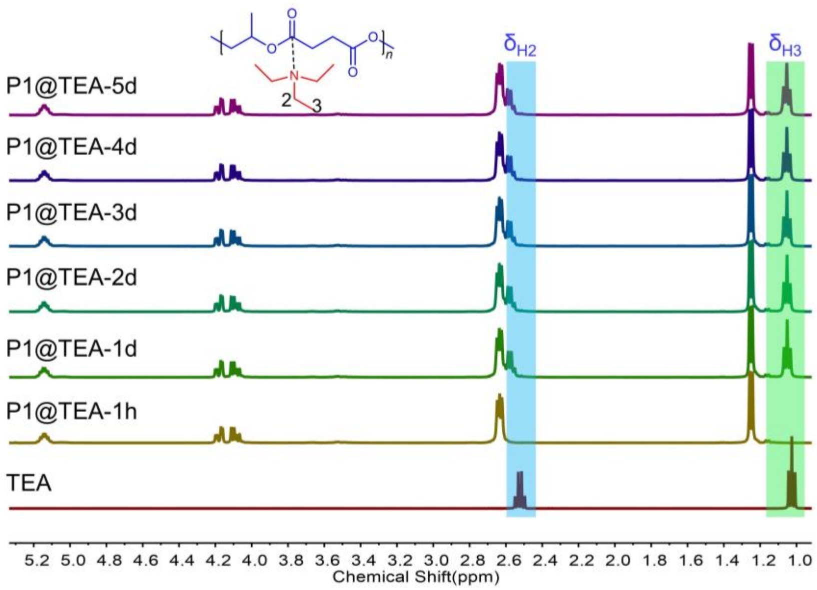

Supplementary Figure 198. Time-dependent ^{1}H-NMR spectra of **P1@TEA** (400 MHz, $CDCl_3$). The molar ratio of structural units of **P1** to TEA is 5:1. The h and d respectively represent hours and days.

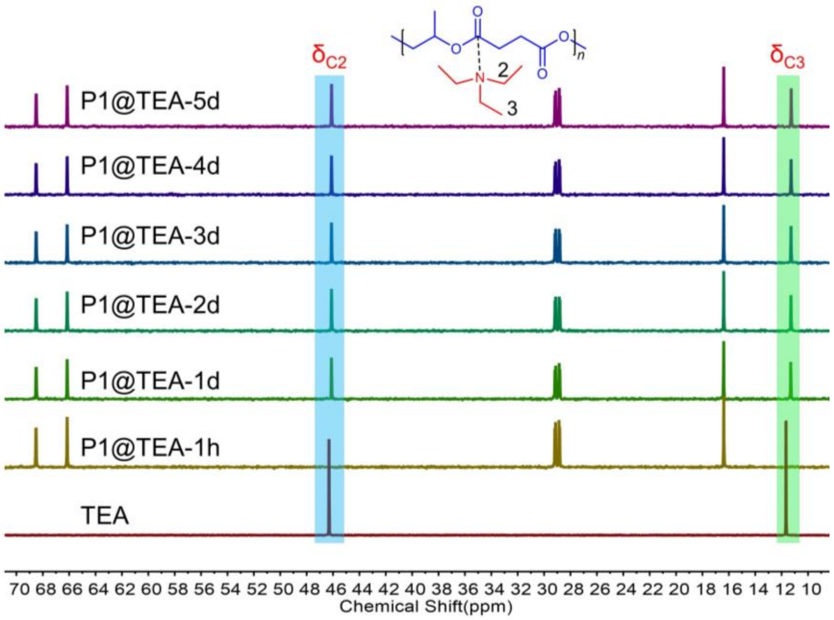

Supplementary Figure 199. Time-dependent ^{13}C-NMR spectra of **P1@TEA** (100 MHz, $CDCl_3$). The molar ratio of structural units of **P1** to TEA is 5:1. The h and d respectively represent hours and days.

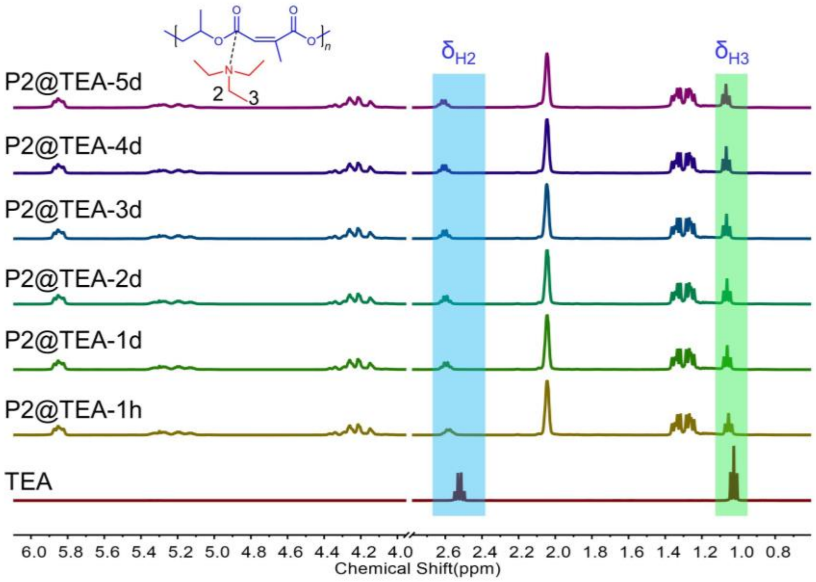

Supplementary Figure 200. Time-dependent ^{1}H-NMR spectra of **P2@TEA** (400 MHz, $CDCl_3$). The molar ratio of structural units of **P2** to TEA is 5:1. The h and d respectively represent hours and days.

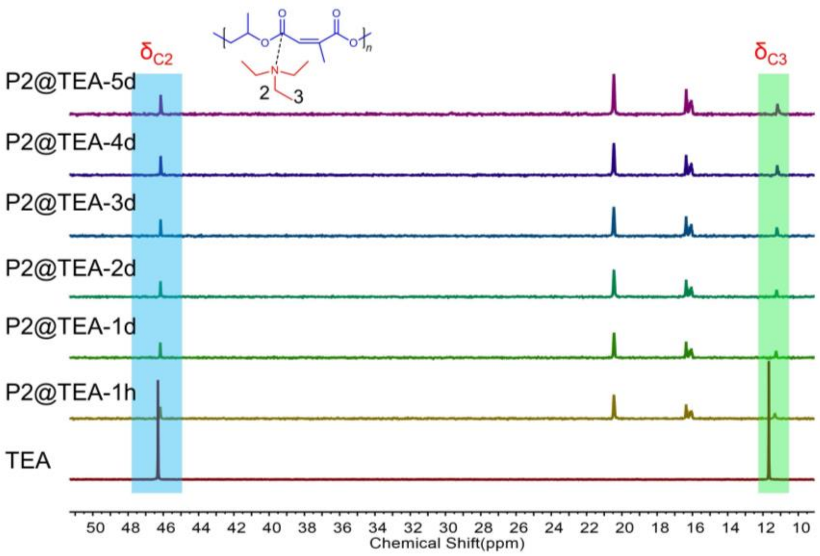

Supplementary Figure 201. Time-dependent ^{13}C-NMR spectra of **P2@TEA** (100 MHz, $CDCl_3$). The molar ratio of structural units of **P2** to TEA is 5:1. The h and d respectively represent hours and days.

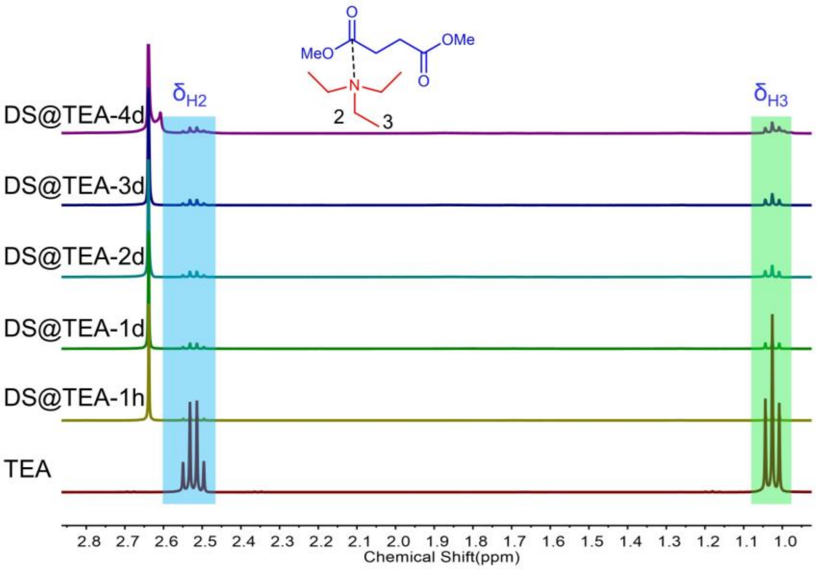

Supplementary Figure 202. Time-dependent ^{1}H-NMR spectra of **DS@TEA** (400 MHz, $CDCl_3$). The molar ratio of structural units of DS to TEA is 5:1. The h and d respectively represent hours and days.

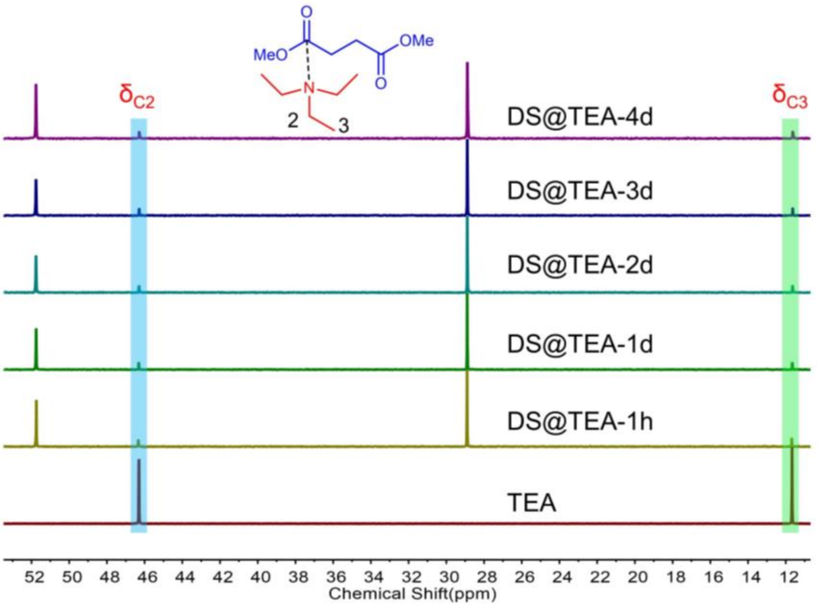

Supplementary Figure 203. Time-dependent ^{13}C-NMR spectra of **DS@TEA** (100 MHz, $CDCl_3$). The molar ratio of structural units of DS to TEA is 5:1. The h and d respectively represent hours and days.

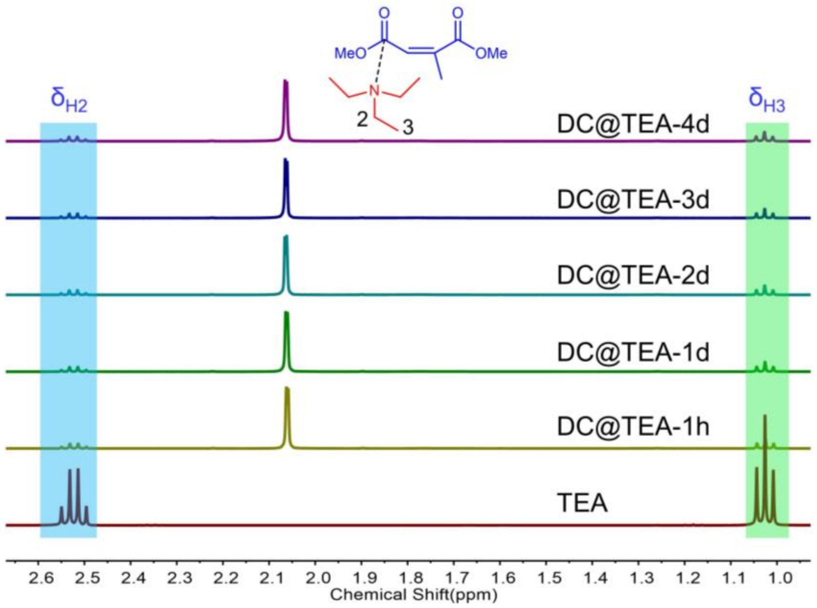

Supplementary Figure 204. Time-dependent ^{1}H-NMR spectra of **DC@TEA** (400 MHz, $CDCl_3$). The molar ratio of structural units of DC to TEA is 5:1. The h and d respectively represent hours and days.

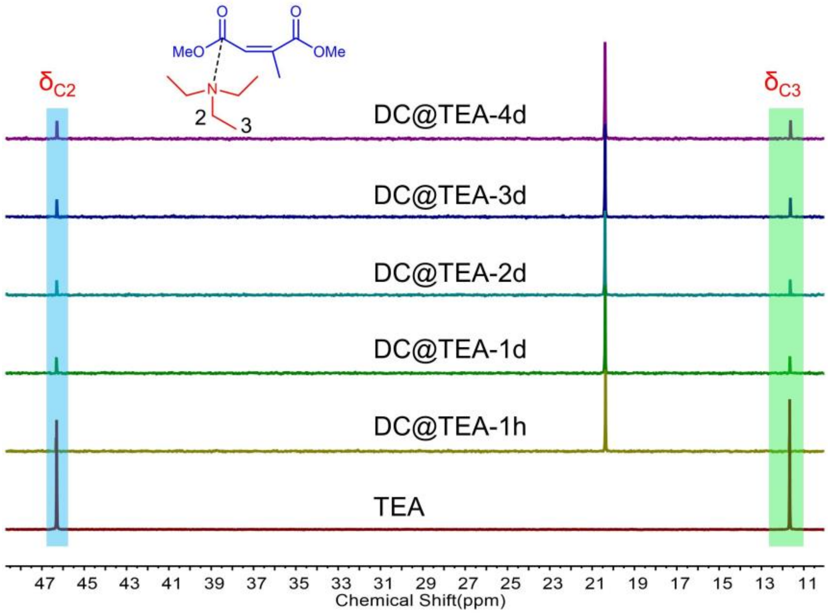

Supplementary Figure 205. Time-dependent ^{13}C-NMR spectra of **DC@TEA** (100 MHz, $CDCl_3$). The molar ratio of structural units of DC to TEA is 5:1. The h and d respectively represent hours and days.

Theoretical calculation on P2@DBU and P2-DBU

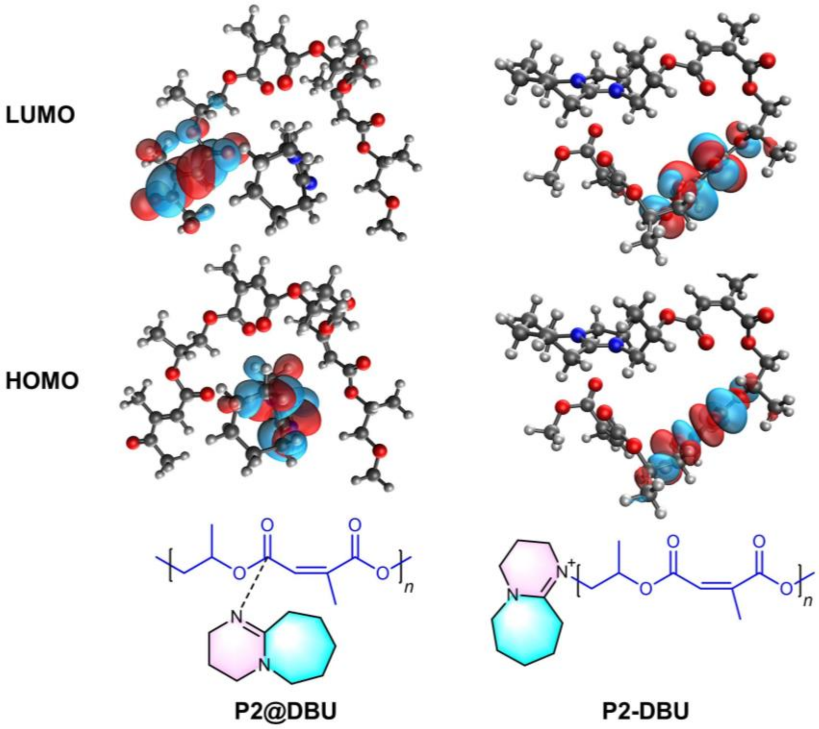

Supplementary Figure 206. Frontier molecular orbitals of optimized excited-state geometries of **P2@DBU** and **P2-DBU** calculated by TD-DFT method at B3LYP-D3/6-31G(d,p) level, Gaussian 09 program. HOMO: the highest occupied molecular orbital, LUMO: the lowest unoccupied molecular orbital.

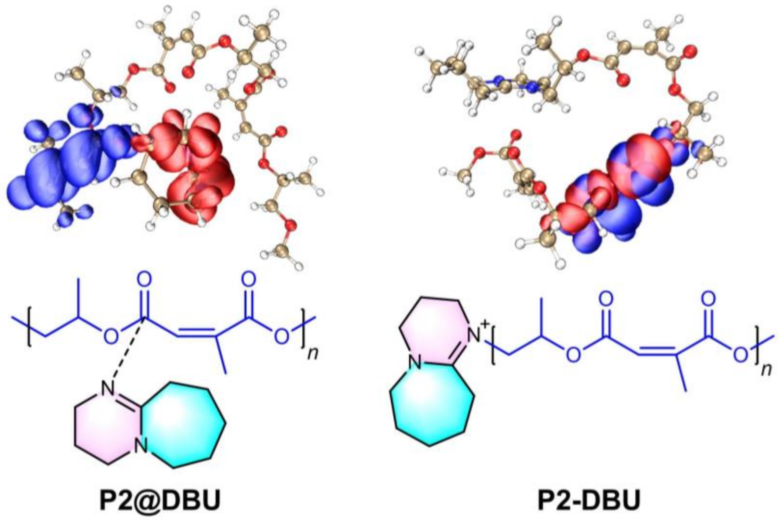

Supplementary Figure 207. Hole (red color) and electron (blue color) of optimized excited-state geometries of **P2@DBU** and **P2-DBU** calculated by TD-DFT method at B3LYP-D3/6-31G(d,p) level, Gaussian 09 program.

Photophysical characterization of amine-ester complexes

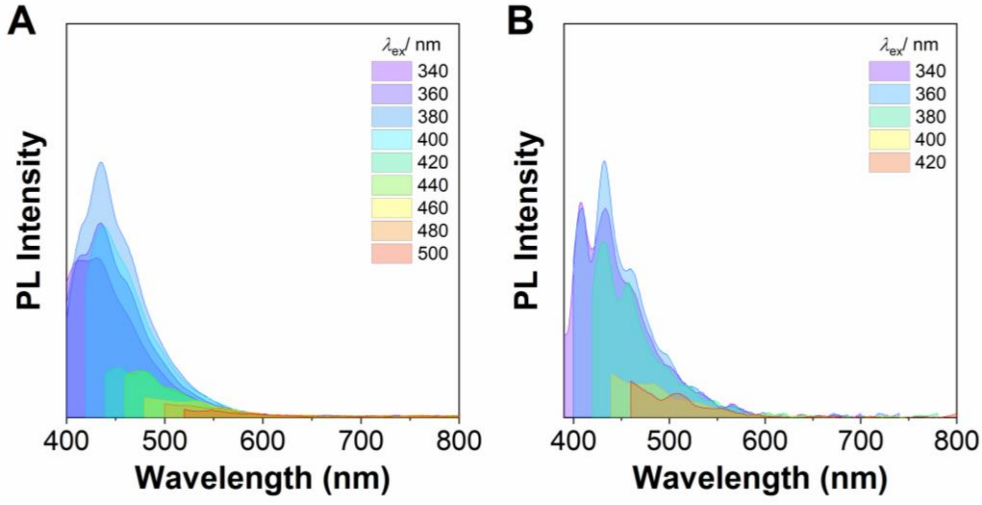

Supplementary Figure 208. PL spectra of (A) DS and (B) DC in bulk under different excitation wavelengths.

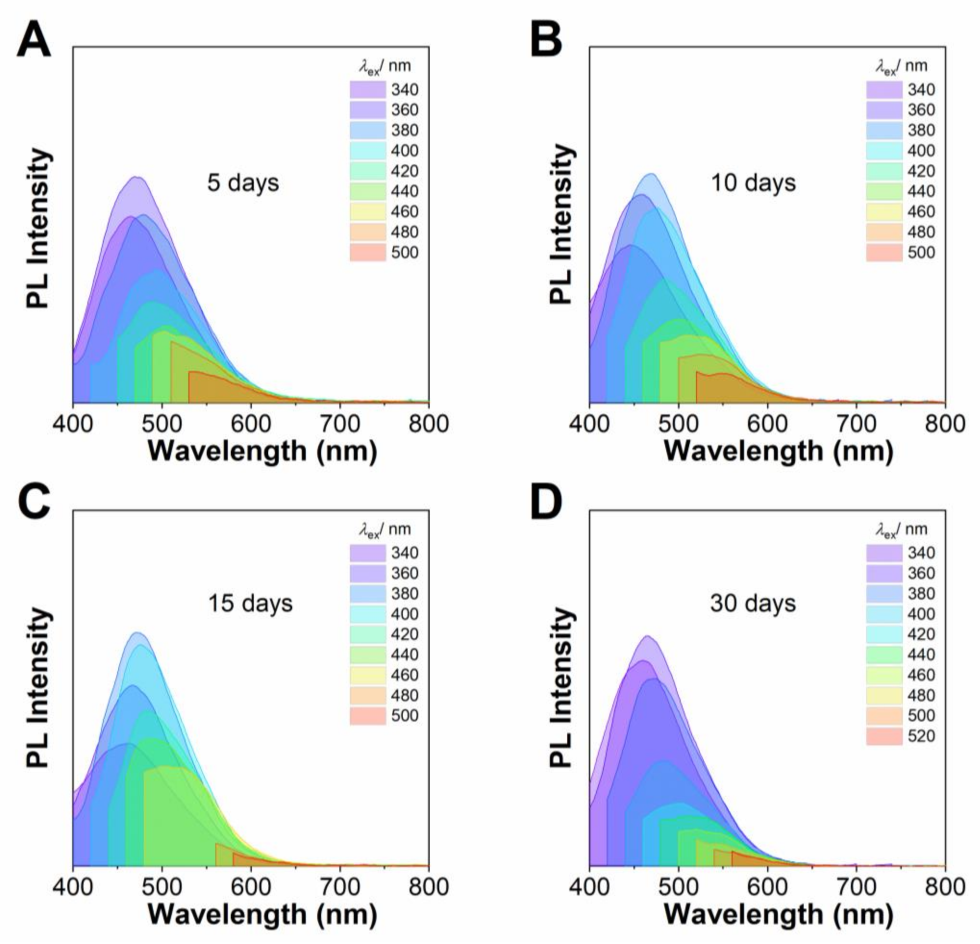

Supplementary Figure 209. (A)~(D) Time-dependent PL spectra of **DS@TEA** in bulk under different excitation wavelengths. The molar ratio of structural units of DS to TEA is 640:1.

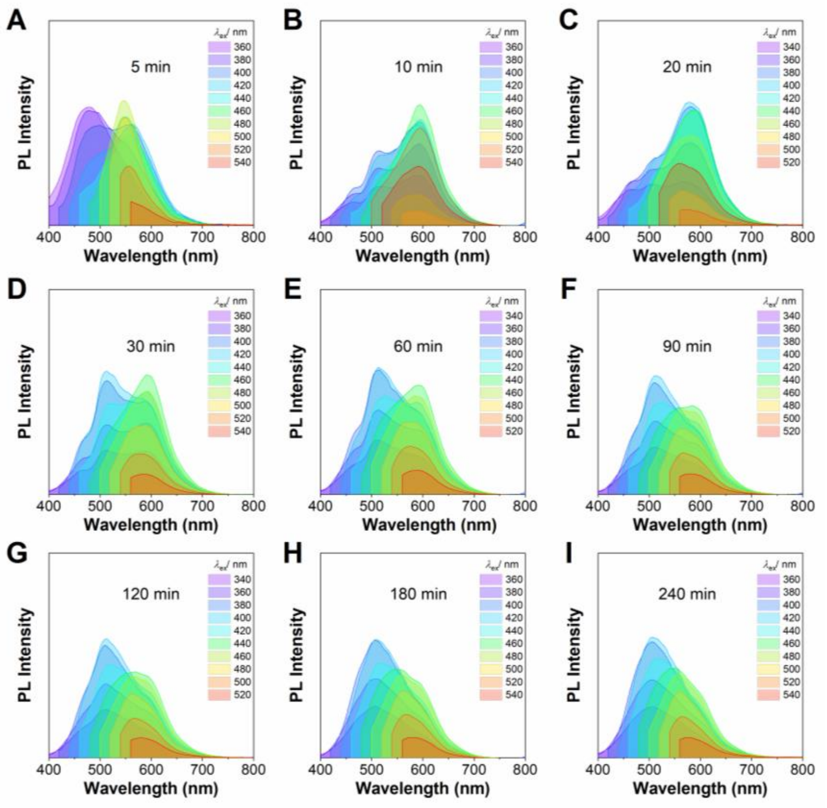

Supplementary Figure 210. (A)~(I) Time-dependent PL spectra of **DS@DBU** in bulk under different excitation wavelengths. The molar ratio of structural units of DS to DBU is 640:1.

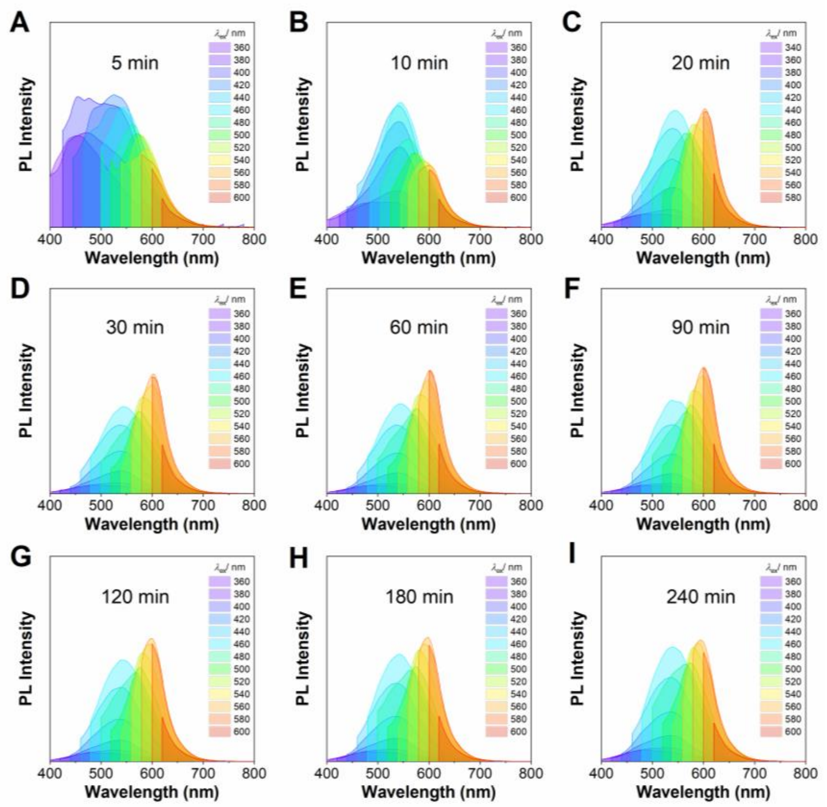

Supplementary Figure 211. (A)~(I) Time-dependent PL spectra of **DC@TEA** in bulk under different excitation wavelengths. The molar ratio of structural units of DC to TEA is 640:1.

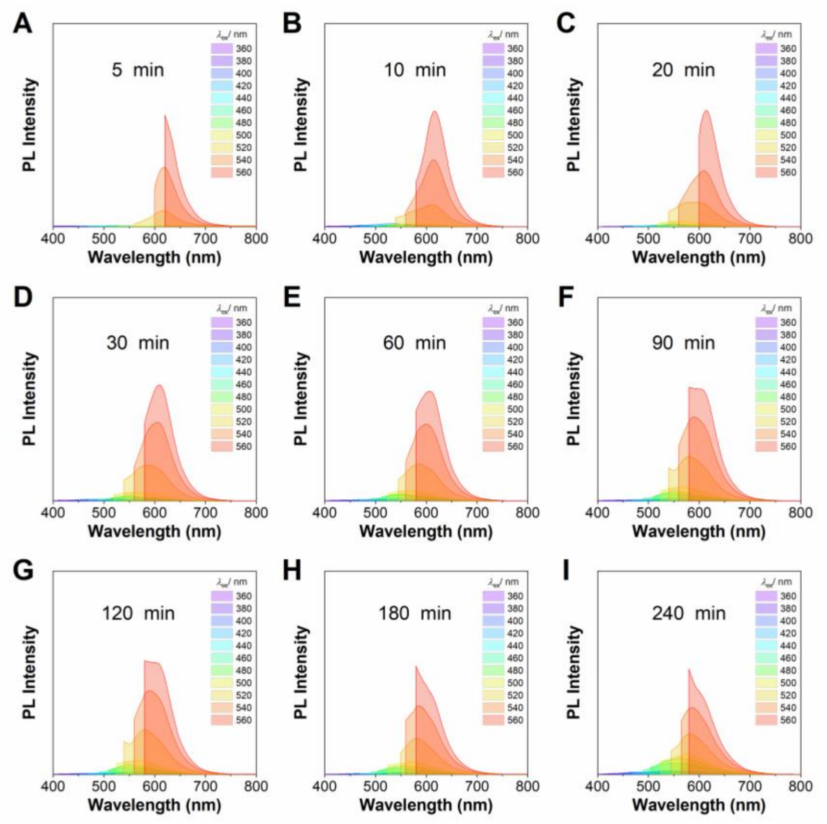

Supplementary Figure 212. (A)~(I) Time-dependent PL spectra of **DC@DBU** in bulk under different excitation wavelengths. The molar ratio of structural units of DC to DBU is 640:1.

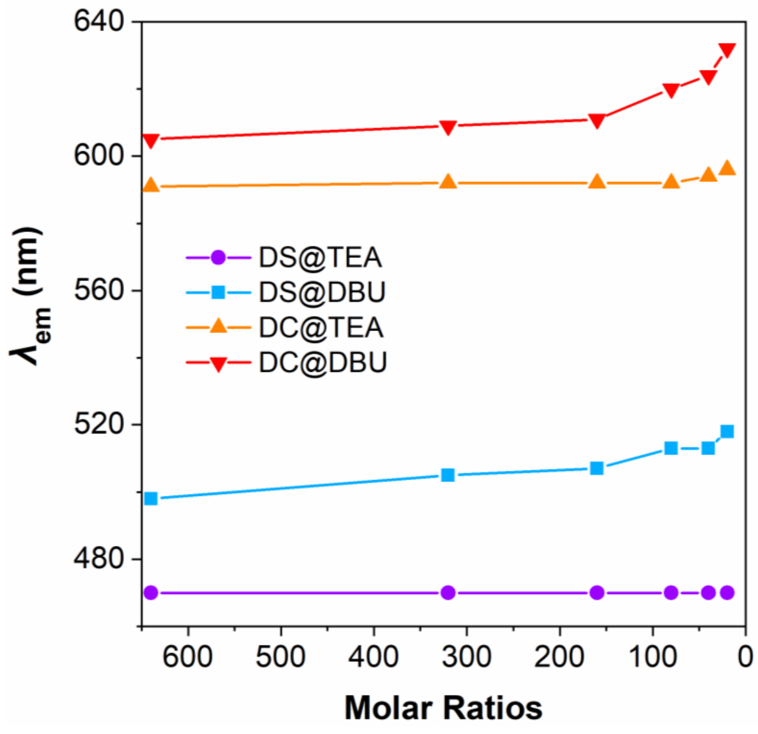

Supplementary Figure 213. Plots of optimal emission wavelength versus molar ratios of DS or DC to TEA or DBU for **DS@TEA**, **DS@DBU**, **DC@TEA** and **DC@DBU**.

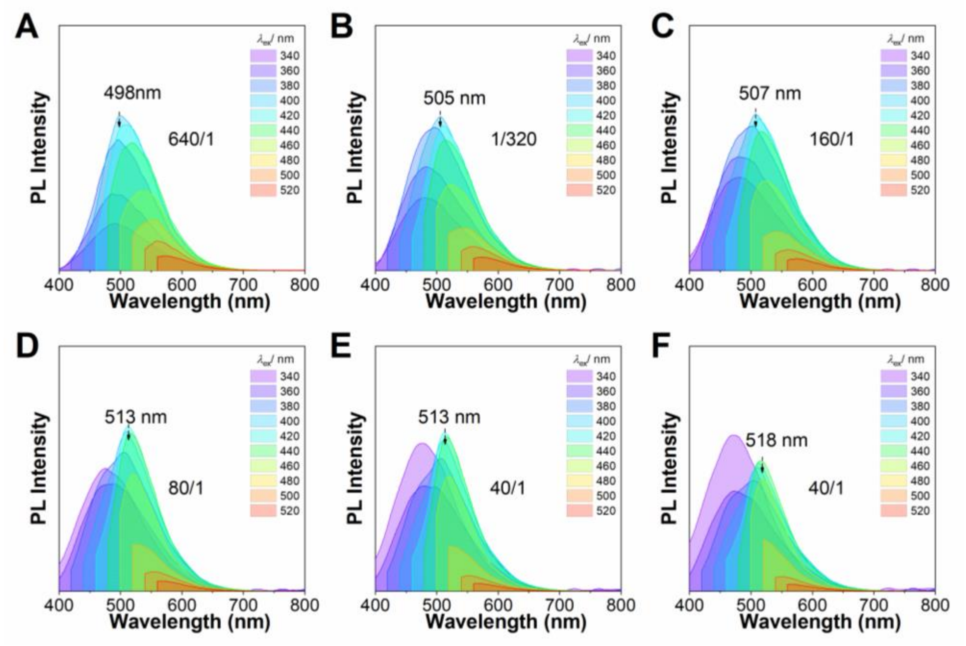

Supplementary Figure 214. (A)~(F) Molar ratios-dependent PL spectra of **DS@DBU** in bulk under different excitation wavelengths. The molar ratios of DS to DBU are from 640/1 to 20/1.

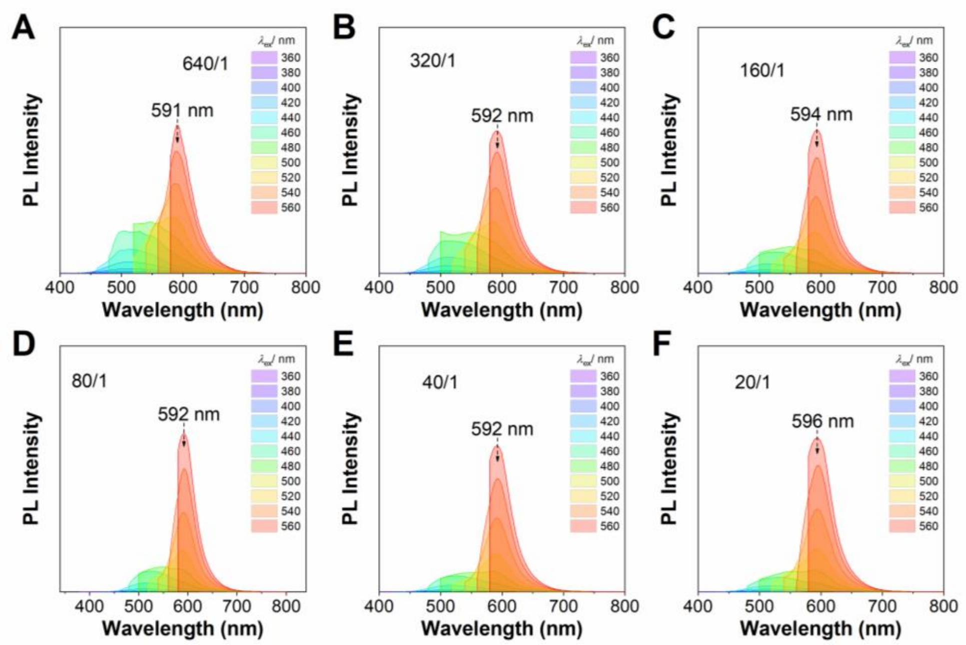

Supplementary Figure 215. (A)~(F) Molar ratios-dependent PL spectra of **DC@TEA** in bulk under different excitation wavelengths. The molar ratios of DC to TEA are from 640/1 to 20/1.

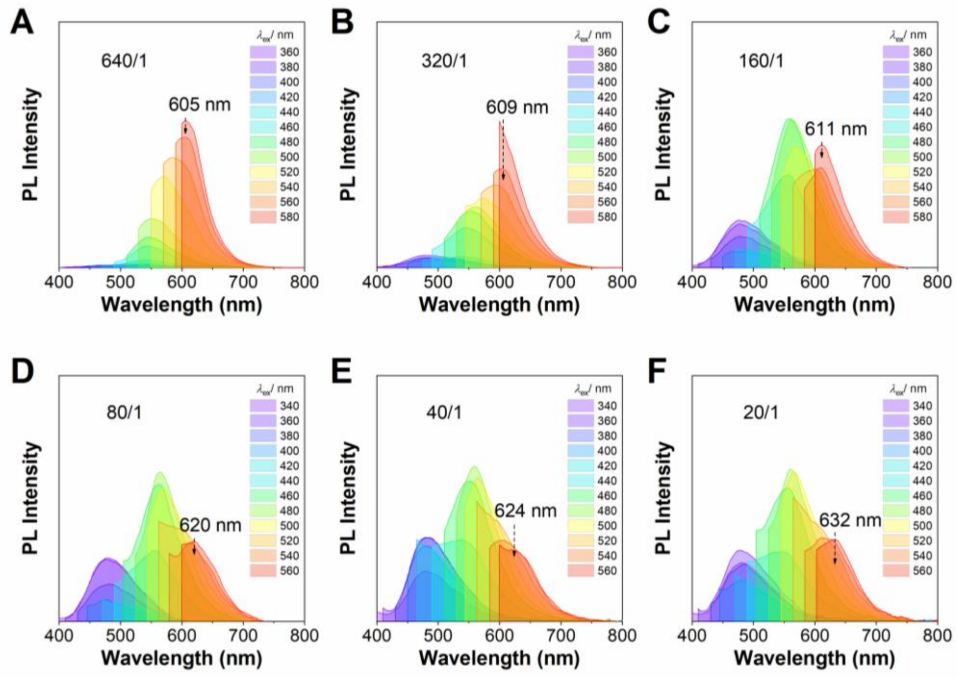

Supplementary Figure 216. (A)~(F) Molar ratios-dependent PL spectra of **DC@DBU** in bulk under different excitation wavelengths. The molar ratios of DC to DBU are from 640/1 to 20/1.

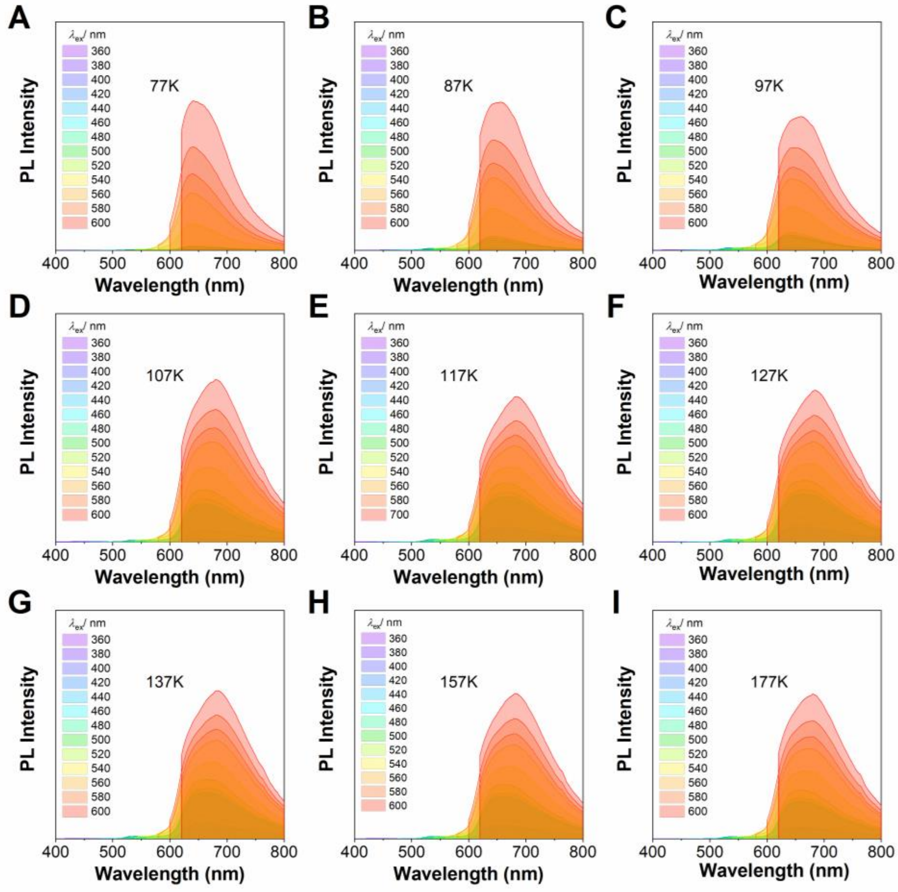

Supplementary Figure 217. (A)~(I) Temperature-dependent PL spectra of **DC@DBU** with temperature from 77K to 177K in bulk under different excitation wavelengths. The molar ratio of DC to DBU is 20/1.

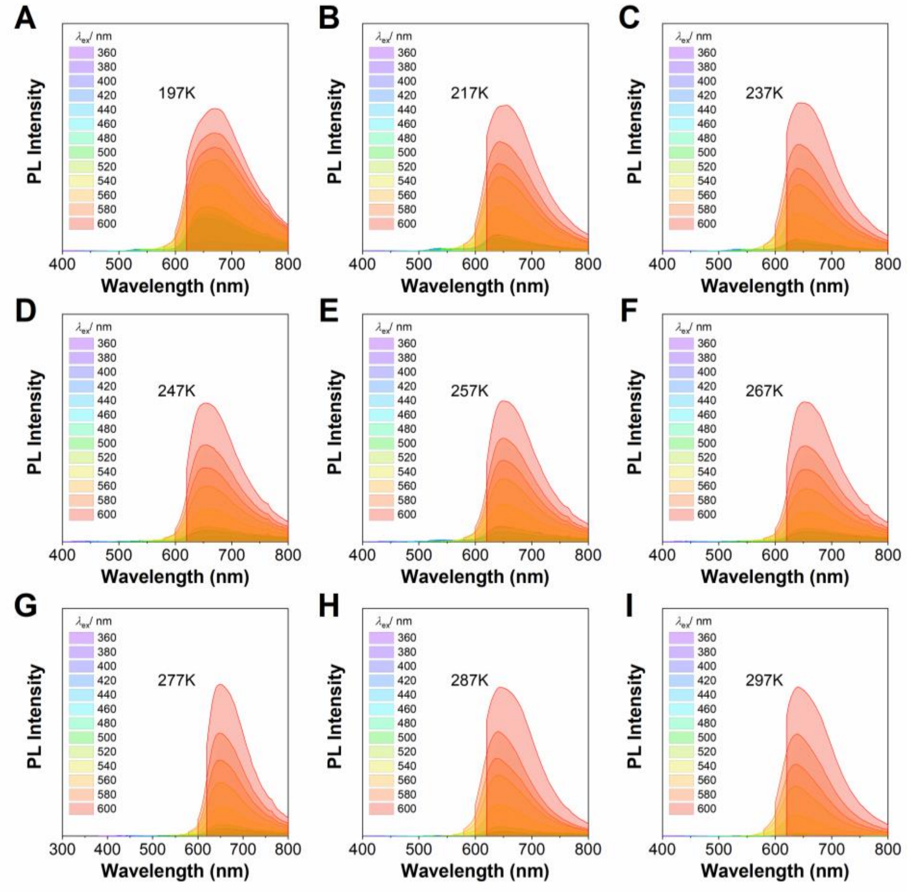

Supplementary Figure 218. (A)~(I) Temperature-dependent PL spectra of **DC@DBU** with temperature from 197K to 297K in bulk under different excitation wavelengths. The molar ratio of DC to DBU is 20/1.

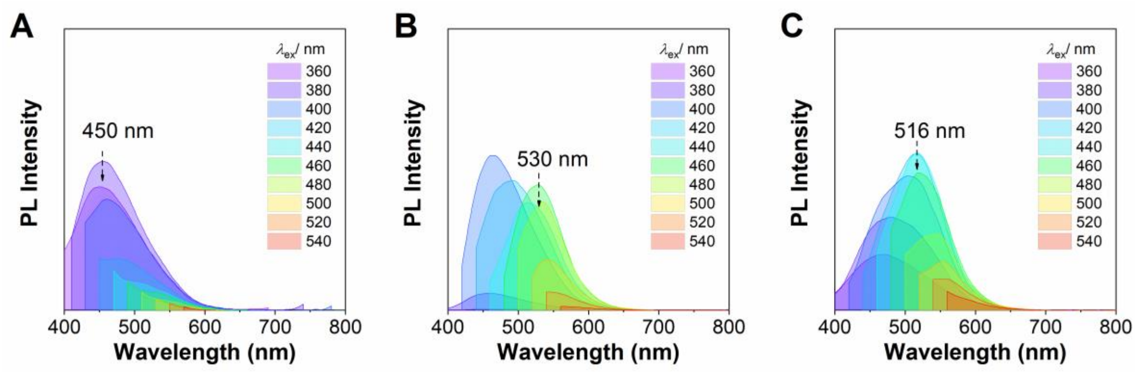

Supplementary Figure 219. PL spectra of (A) **P1**, (B) **P1@TEA** and (C) **P1@DBU** in DCM solution of 10^{-1} M with molar ratios of ester units to amines are 1/2 under different excitation wavelengths.

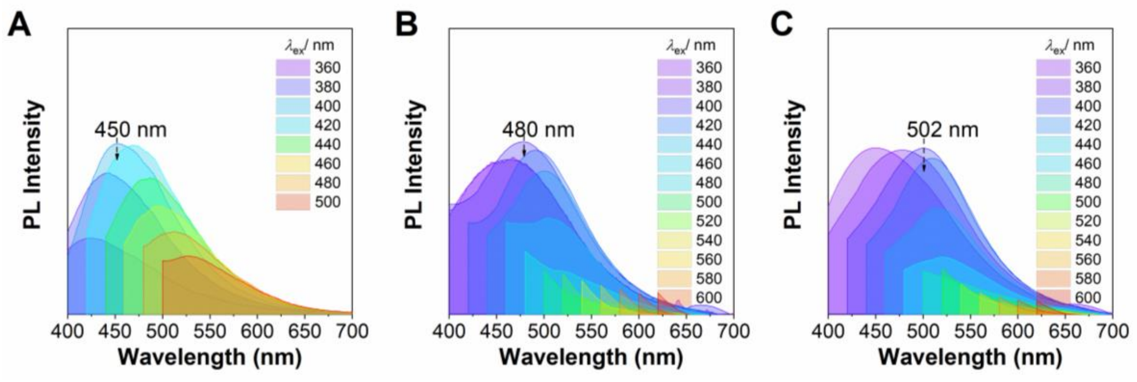

Supplementary Figure 220. PL spectra of (A) **P1**, (B) **P1@TEA** and (C) **P1@DBU** in solid with molar ratios of ester units to amines are 1/2 under different excitation wavelengths.

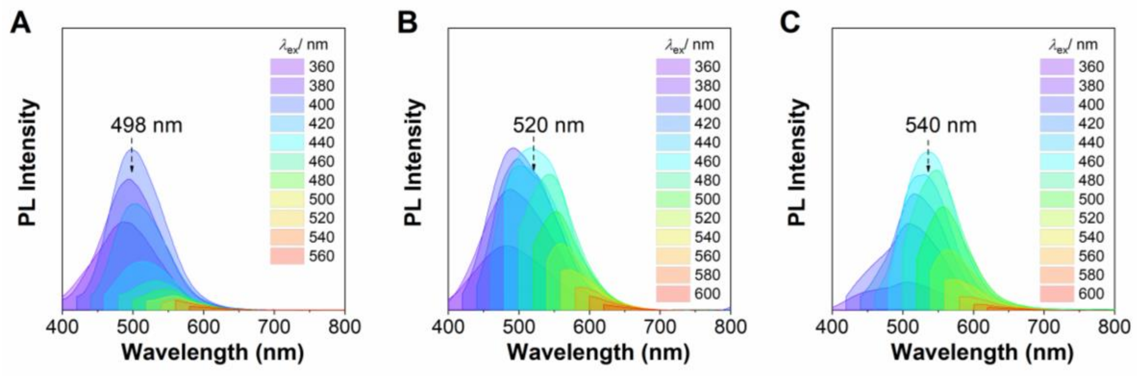

Supplementary Figure 221. PL spectra of (A) **P2**, (B) **P2@TEA** and (C) **P2@DBU** in DCM solution of 10^{-1} M with molar ratios of ester units to amines are 1/2 under different excitation wavelengths.

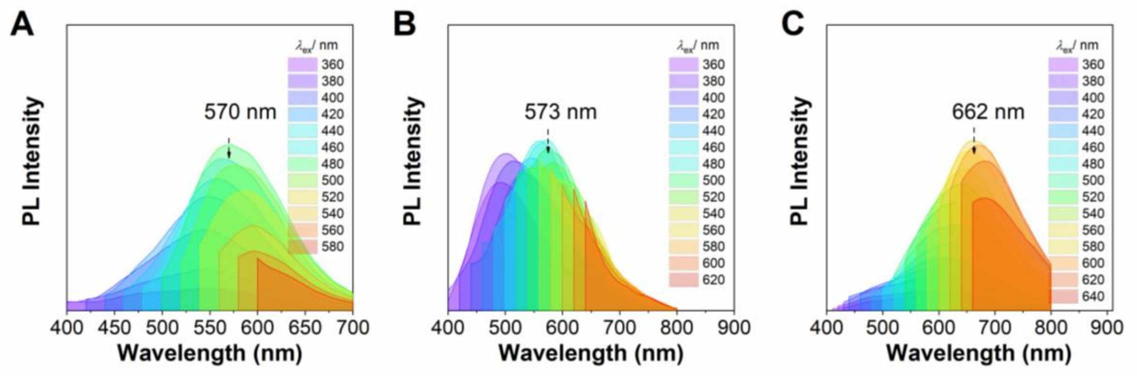

Supplementary Figure 222. PL spectra of (A) **P2**, (B) **P2@TEA** and (C) **P2@DBU** in solid with molar ratios of ester units to amines are 1/2 under different excitation wavelengths.

Photophysical characterizaion of amine-initiated polyesters after eluting terminal amines

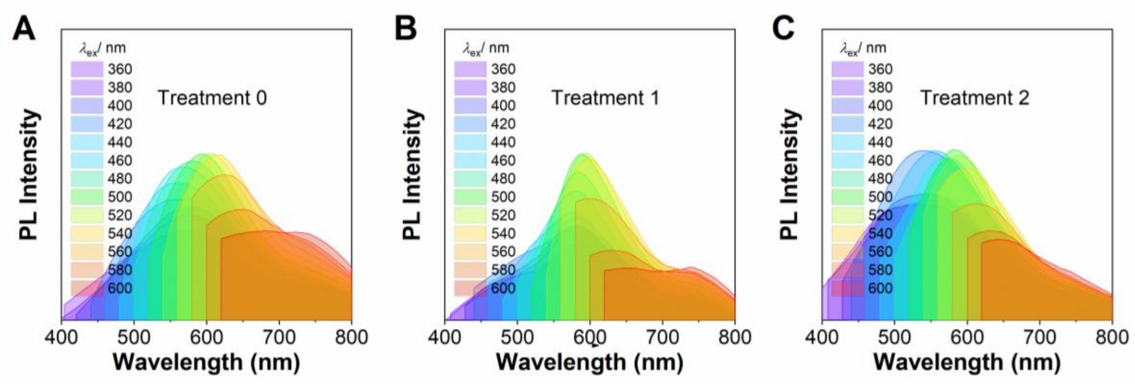

Supplementary Figure 223. PL spectra of **P1-5.0DBU** in solid precipitated in ethanol under different excitation wavelengths. Treatment 0, 1, 2 respectively represent once precipitated treatment without hydrochloric acid, once and twice precipitated treatment under hydrochloric acid of 37 *wt*% (5 drops of hydrochloric acid for per precipitation).

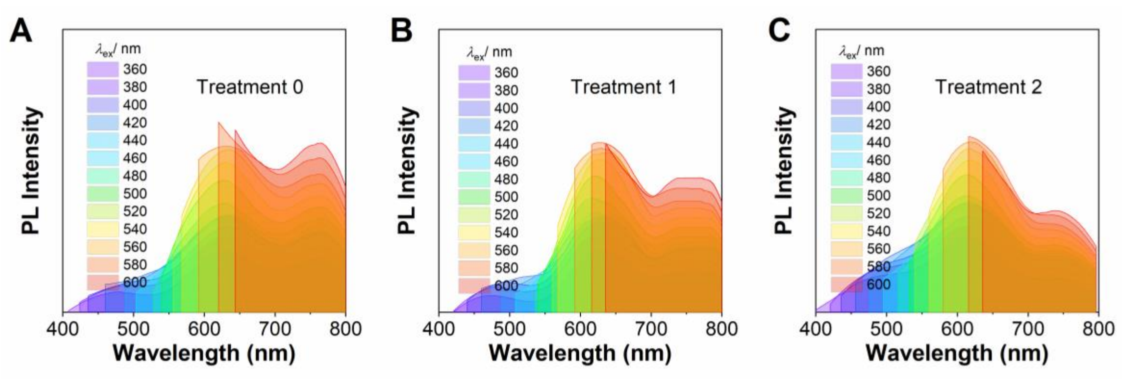

Supplementary Figure 224. PL spectra of **P2-5.0DBU** in solid precipitated in ethanol under different excitation wavelengths. Treatment 0, 1, 2 respectively represent once precipitated treatment without hydrochloric acid, once and twice precipitated treatment under hydrochloric acid of 37 *wt*% (5 drops of hydrochloric acid for per precipitation).

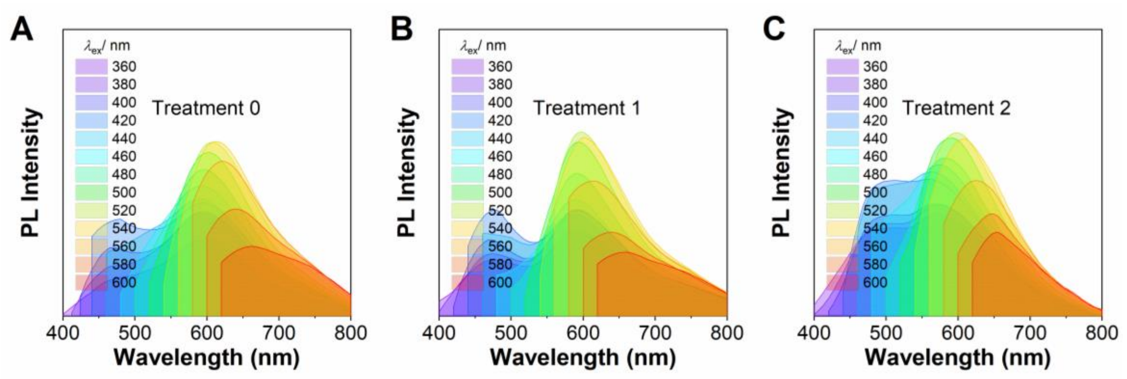

Supplementary Figure 225. PL spectra of **P1-5.0TEA** in solid precipitated in ethanol under different excitation wavelengths. TreatmentTreatment 0, 1, 2 represent precipitated treatment without hydrochloric acid, once and twice precipitated treatment under hydrochloric acid of 37 *wt*% (5 drops of hydrochloric acid for per precipitation).

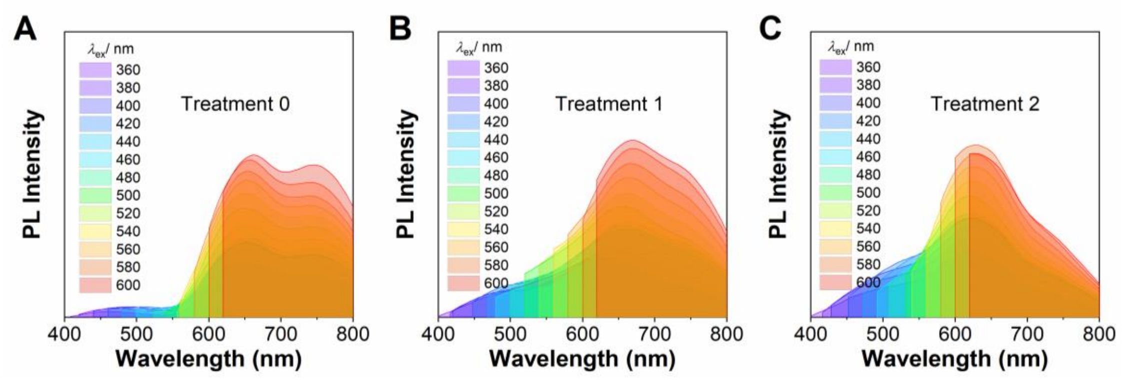

Supplementary Figure 226. PL spectra of **P2-5.0TEA** in solid precipitated in ethanol under different excitation wavelengths. Treatment 0, 1, 2 respectively represent once precipitated treatment without hydrochloric acid, once and twice precipitated treatment under hydrochloric acid of 37 wt% (5 drops of hydrochloric acid for per precipitation).

Supplementary Table 1. Summary of physical properties of P1 and P1-amines

Sample	Ester[a] (%)	M_n[b] (kg/mol)	PDI[b]	λ_{abs}[c] (DCM)(nm)	λ_{em}[d] (DCM)(nm)	λ_{em}[e] (solid)(nm)	Φ[d] (DCM, %)	Φ[e] (solid, %)
**P1**	>99	10.8	1.38	253, 306	460	450	7.6	9.20
**P1-0.5TEA**	>99	5.2	1.38	247,310	437, 520	473, 587	4.9	10.4
**P-1.0TEA**	>99	6.9	1.69	250, 317, 364	441, 512	475, 584	4.0	13.0
**P1-1.5TEA**	97	2.9	1.90	253, 306, 376	467, 560	474, 598	4.6	12.2
**P1-2.0TEA**	93	6.9	1.58	252, 306, 374	485, 570	472, 595	5.2	11.6
**P1-3.0TEA**	85	6.7	1.51	353, 307, 387, 441	485, 575	467, 595	3.8	11.7
**P1-4.0TEA**	64	7.4	1.60	252, 305, 388,433	483, 582	475, 593	3.4	12.8
**P1-5.0TEA**	>99	18.2	1.55	253, 300, 386,438	489, 582	471, 602	4.0	15.8
**P1-2.0DBU**	>99	4.2	1.84		465, 595	468, 604	3.8	19.4
**P1-2.0mTBD**	>99	6.1	2.21		491,583	475, 580	3.9	12.8
**P1-2.0TBD**	>99	4.1	2.10		474, 580	481, 593	3.3	8.24

a: Ester contents calculated by ^{1}H NMR spectra. b: number-average molecular weight and polydispersity measured by GPC using THF as the eluent. c: UV-Vis absorption spectra in DCM (10^{-3} M). d: PL spectra and QYs in DCM (10^{-1} M). e: PL spectra and QYs in solid. QYs were measured λ_{ex} =360 nm.

Supplementary Table 2. Summary of physical properties of P2 and P2-amines

Sample	Ester[a] (%)	M_n[b] (kg/mol)	PDI[b]	λ_{abs}[c] (DCM)(nm)	λ_{em}[d] (DCM)(nm)	λ_{em}[e] (solid)(nm)	Φ[d] (DCM, %)	Φ[e] (solid, %)
**P2**	>99	10.4	1.62	242, 300	490	477, 572	4.2	4.4
**P2-0.5TEA**	>99	3.9	2.91	294, 353	500, 613	496, 605, 717	9.6	5.0
**P2-1.0TEA**	>99	3.2	2.00	296, 347	500, 612	499, 614, 740	6.1	3.8
**P2-1.5TEA**	>99	2.7	2.78	296, 356	498, 611	494, 616, 738	7.2	2.1
**P2-2.0TEA**	>99	2.9	2.95	292, 353	501, 612, 722	493, 614, 745	6.5	1.9
**P2-3.0TEA**	>99	2.4	2.79	292, 332	649, 722	495, 617, 748	4.8	1.2
**P2-4.0TEA**	>99	1.7	2.78	290, 338	659, 725	497, 640, 759	2.9	1.3
**P2-5.0TEA**	>99	1.6	3.90	298, 350	660, 740	490, 640, 750	3.7	1.4
**P2-2.0DBU**	>99	4.8	2.40	290, 347	668, 730	487, 656, 747	3.7	1.0
**P2-2.0mTBD**	>99	8.4	1.53	294, 349	654, 730	484, 636, 727	2.9	1.2
**P2-2.0TBD**	>99	6.9	1.74	295, 334	648, 738	491, 650, 769	4.6	0.6

a: Ester contents calculated by ^1H NMR spectra. b: number-average molecular weight and polydispersity measured by GPC using THF as the eluent. c: UV-Vis absorption spectra in DCM (10^{-3} M). d: PL spectra and QYs in DCM (10^{-1} M). e: PL spectra and QYs in solid. QYs were measured at λ_{ex} =500 nm.

Supplementary Reference

1 Tirado-Rives, J. & Jorgensen, W. L. Performance of B3LYP Density Functional Methods for a Large Set of Organic Molecules. *J. Chem. Theory Comput.* **4**, 297-306 (2008).

2 Lu, T. & Chen, F. Multiwfn: A multifunctional wavefunction analyzer. *J. Comput. Chem.* **33**, 580-592 (2012).

3 Lu, T. Multiwfn. *Software manual. Version* **3** (2014).

4 Humphrey, W., Dalke, A. & Schulten, K. VMD: Visual molecular dynamics. *Journal of Molecular Graphics* **14**, 33-38 (1996).
